# Integrated Proteomics Identified Up-Regulated Focal Adhesion-Mediated Proteins in Human Squamous Cell Carcinoma in an Orthotopic Murine Model

**DOI:** 10.1371/journal.pone.0098208

**Published:** 2014-05-23

**Authors:** Daniela C. Granato, Mariana R. Zanetti, Rebeca Kawahara, Sami Yokoo, Romênia R. Domingues, Annelize Z. Aragão, Michelle Agostini, Marcelo F. Carazzolle, Ramon O. Vidal, Isadora L. Flores, Johanna Korvala, Nilva K. Cervigne, Alan R. S. Silva, Ricardo D. Coletta, Edgard Graner, Nicholas E. Sherman, Adriana F. Paes Leme

**Affiliations:** 1 Laboratório de Espectrometria de Massas, Laboratório Nacional de Biociências, LNBio, CNPEM, Campinas, Brazil; 2 Faculdade de Odontologia de Piracicaba, Universidade Estadual de Campinas, UNICAMP, Piracicaba, Brazil; 3 Faculdade de Odontologia, Universidade Federal do Rio de Janeiro, UFRJ, Rio de Janeiro, Brazil; 4 W. M. Keck Biomedical Mass Spectrometry Lab. University of Virginia, Charlottesville, Virginia, United States of America; 5 Institute of Dentistry, University of Oulu, Oulu, Finland; Ottawa Hospital Research Institute, Canada

## Abstract

Understanding the molecular mechanisms of oral carcinogenesis will yield important advances in diagnostics, prognostics, effective treatment, and outcome of oral cancer. Hence, in this study we have investigated the proteomic and peptidomic profiles by combining an orthotopic murine model of oral squamous cell carcinoma (OSCC), mass spectrometry-based proteomics and biological network analysis. Our results indicated the up-regulation of proteins involved in actin cytoskeleton organization and cell-cell junction assembly events and their expression was validated in human OSCC tissues. In addition, the functional relevance of talin-1 in OSCC adhesion, migration and invasion was demonstrated. Taken together, this study identified specific processes deregulated in oral cancer and provided novel refined OSCC-targeting molecules.

## Introduction

Oral cancer is one of the most common malignancies worldwide [1], [2] and the third most frequent cancer, with a 5-year survival rate less than 50% [3]. The development of oral squamous cell carcinoma (OSCC) requires the accumulation of several genetic alterations that are affected by genetic predisposition and environmental conditions such as tobacco, alcohol, chronic inflammation and viral infection [4]. Because cancer is a complex and multifactorial disease, exploring the molecular pathways involved in this process is necessary to achieve successful treatment of each specific case and improve the understanding of pathogenesis [4]–[6].

Therefore, in order to investigate the mechanisms for oral cancer development, this study focused on analyzing the differential expression of proteins and peptides in OSCC compared to normal tissue using an orthotopic murine model, which recapitulates the local tumor microenvironment [7], [8]. We used a two-step approach by first injecting SCC-9 cells and the respective control cells in the tongues of immunodeficient mice to induce tumor development. After 20 days, tumor and control tissues were isolated, and extracted proteins and peptides were analyzed using mass spectrometry, followed by validation using human OSCC tissues. We demonstrated that the strategies used here enabled the identification of up-regulated focal adhesion-mediated proteins for OSCC, such as filamins A and B, catenin alpha-1 and talin-1 as potential proteins involved in OSCC development.

## Materials and Methods

### Cell culture

The human OSCC cell line SCC-9 was obtained from American Type Culture Collection (ATCC, Manassas, VA, USA), and cultured as recommended. SCC-9 cells are originated from human squamous carcinoma from the tongue. The HaCaT cells, an immortalized but not transformed epithelial cell line [9], was maintained in DMEM containing 10% fetal bovine serum (FBS) and antibiotics at 37°C in a 5% CO_2_ air atmosphere. HaCaT cells are human keratinocytes originated from skin. Control cells were used to assure that all the animals were subjected to the same procedures. Human Epidermoid Carcinoma A431 (epidermoid carcinoma cell line originated from skin) was grown in Roswell Park Memorial Institute (RPMI) −1640 medium supplemented with 10% FBS and antibiotics at 37°C in a 5% CO_2_ air atmosphere. Metastatic SCC-9 cells were isolated from lymph nodes (LN) originating the cell line SCC-9 LN1 [10]. This cell line was cultured as recommended for SCC-9 cells.

### Tissue sample preparation

HaCaT and SCC-9 cells were grown until 75% confluence and 2.5×10^5^ cells in 20 µl of phosphate-buffered saline were implanted into the right lateral portion of the tongue of 6- to 8-week-old male Balb/c nude mice, using a syringe with a 30 gauge disposable needle (BD Biosciences). This procedure was approved by the Institutional Committee for Ethics in Animal Research of the University of Campinas. Mice were sacrificed 20 days after implantation and the control and tumor tissues were immediately removed and frozen in dry ice. A small piece of each tumor was fixed in formalin and embedded in paraffin for histopathological examination after H&E staining. We performed three independent experiments for the analysis of the protein and peptide expression in control and tumor tissues. Each sample is composed of a pool of three mouse tissues, either from control or tumor tissues. The samples were named as Control 1 (experiment 1, n = 3), Control 2 (experiment 2, n = 3), Control 3 (experiment 3, n = 3) and Tumor 1 (experiment 1, n = 3), Tumor 2 (experiment 2, n = 3) and Tumor 3 (experiment 3, n = 3). The control and tumor tissues were homogenized with liquid nitrogen using mortar and pestle. Tissue protein from each of the three mice were separately resuspended with 50 µl of extraction buffer in urea containing protease inhibitors [11] and incubated at room temperature for 30 min. After centrifugation at 12,000×*g* for 10 min at 4°C, the supernatant was quantified using the Bradford method reagent (BioRad) as previously described [12]. Then the same protein amount was pooled from three mouse samples, either from control tissues or from tumor tissues, to be analyzed by LC-MS/MS. Three independent experiments were performed.

### Sample preparation for LC-MS/MS

The extracted proteins were reduced (5 mM ditiotreitol, 25 min at 56°C), alkylated (14 mM iodoacetamide, 30 min at room temperature in the dark) and digested with trypsin (Promega), the peptides were desalinized using the column Sep-pak C18 cartridge (Waters), dried down in a vacuum concentrator and reconstituted in 0.1% formic acid.

Regarding the identification of endogenous cleavage peptides by LC-MS/MS, 672 µg of extracted protein from tissues as described before were precipitated with the final concentration of 10 mM HCl. After centrifugation, the supernatant was collected, the peptides were desalinized using the column Sep-pak C18 cartridge (Waters) and the peptides were dried down in a vacuum concentrator and resuspended in 20 µl of 0.1% formic acid.

### LC-MS/MS analysis

The protein derived samples (2 µg) and endogenous cleavage peptides were analyzed on an ETD enabled LTQ Velos Orbitrap instrument (Thermo Fisher Scientific) connected to nanoflow liquid chromatography tandem mass spectrometry (LC-MS/MS) on an EASY-nLC system (Proxeon Biosystem) through a Proxeon nanoelectrospray ion source. The resulting peptides were separated by 2–90% acetonitrile gradient in 0.1% formic acid using a pre-column EASY-Column (2 cm× ID100 µm, 5 µm particle size, Thermo Fisher Scientific) and the a PicoFrit Column (20 cm× ID75 µm, 5 µm particle size, New Objective), at a flow rate 300 nl/min over 135 min. The nanoelectrospray voltage was set to 2.5 kV and the source temperature was 200°C. All instrument methods for the Orbitrap Velos were set up in the data dependent acquisition mode. The full scan MS spectra (from m/z 300–1600) were acquired in the Orbitrap analyzer after accumulation to a target value of 1e^6^ in the linear ion trap. Resolution in the Orbitrap system was set to *r* = 60,000 and the 20 most intense peptide ions with charge states ≥2 were sequentially isolated to a target value of 10,000 and fragmented in high-pressure linear ion trap by low-energy CID (collision-induced dissociation) normalized collision energy of 35%. The signal threshold for triggering a MS/MS event was set to 1000 counts. Dynamic exclusion was enabled with exclusion size list of 200 and exclusion duration of 60 s. An activation q of 0.25 and activation time of 10 ms were used [13].

For the identification of endogenous cleavage peptides by LC-MS/MS, the samples (4.5 µl) were analyzed on an ETD enabled Orbitrap Velos instrument as described before, except for gradient run that was performed over 45 min. All instrument methods for the LTQ Velos Orbitrap were set up in the data dependent acquisition mode in ETD (electron transfer dissociation), HCD (higher-energy collisional dissociation) and CID fragmentations. For CID fragmentation mode, the same method used for digested proteins was performed. For HCD mode, resolution in the Orbitrap system was set to *r* = 60,000 and the 5 most intense peptide ions with charge states ≥2 were sequentially isolated to a target value of 50,000 and fragmented in HCD with normalized collision energy of 40%, resolution in the Orbitrap system was set to *r* = 7,500. The signal threshold for triggering a MS/MS event was set to 100,000 counts. Dynamic exclusion was enabled with exclusion size list of 200 and exclusion duration of 20 s and activation time of 10 ms was used. For ETD, resolution in the Orbitrap system was set to *r* = 60,000 and the 5 most intense peptide ions with charge states ≥2 were sequentially isolated to a target value of 50,000 and fragmented in high-pressure linear ion trap and readout in the Orbitrap system with *r* = 7,500 for MS/MS. The signal threshold for triggering an MS/MS event was set to 500,000 counts. Dynamic exclusion was enabled with exclusion size list of 200 and exclusion duration of 20 s. An activation q of 0.25 and activation time of 100 ms were used, with supplemental activation.

All mass spectrometric raw files associated with this study may be available for downloading via FTP from the PeptideAtlas data repository by accessing the following link: http://www.peptideatlas.org/PASS/PASS00365.

### Data analysis, bioinformatic analysis and statistical analysis

Peak lists (msf) were generated from the raw data files using Proteome Discoverer version 1.3 (Thermo Fisher Scientific) with Sequest search engine and searched against Human and Mouse International Protein Databases (IPI) v. 3.86 (IPI Human: 91,522 sequences; 36,630,302 residues, release July 2011 and IPI Mouse: 58,667 sequences, 26,399,545 residues, release July 2011) with carbamidomethylation as fixed modification, oxidation of methionine as variable modifications, one trypsin missed cleavage and a tolerance of 10 ppm for precursor and 1 Da for fragment ions. The data were analyzed against Human and Mouse databases, considering the orthotopic model, in which the tumor developed in mouse tongue is originated from human cells and the control tissue is originated from mouse tissues.

Regarding the analysis of endogenous cleavage peptides by LC-MS/MS, they were performed as described above, except for the parameters: no enzyme was specified for cleavage and a tolerance of 10 ppm for precursor and 1 Da for fragment ions for top 20 CID (collision-induced dissociation); and for top 5 HCD (higher-energy collisional dissociation) and top 5 ETD (electron-transfer dissociation) fragmentations, a tolerance of 10 ppm for precursor and 0.02 Da for fragment ions were used. All datasets of proteins and endogenous cleavage peptides were processed using the workflow feature in Proteome Discoverer software and the msf files were analyzed in ScaffoldQ+v.3.3.2 (Proteome Software), filtered using xcorr cutoffs (+1>1.8, +2>2.2, +3>2.5 and +4>3.5). The scoring parameters in ScaffoldQ**+** were set to obtain a false discovery rate less than 1%.

For the analysis of protein and peptide expression, the average number of unique peptides from tumor and control samples was compared directly to obtain the fold-change ratio (FC). To avoid division by zero caused by samples with no unique peptides we added 1 on both averages. The statistical significance of the peptides for each protein was assessed by a two-tailed Fisher's exact test calculated by R [14], [15]. Proteins and peptides with FC>2.0 and p-value <0.05 were selected for the Tables A and B.

Heat map of differential expressed proteins given by Fisher's exact test was performed in Perseus software [16] using Z-score applied on spectral counts.

To explore in-depth the biological significances of up-regulated and down-regulated proteins, the biological processes of GO terms and KEGG (Kyoto Encyclopedia of Genes and Genomes) [17] pathway annotation were analyzed simultaneously using DAVID Gene Functional Classification Tool [18] with the p-value set at 0.05.

Differentially expressed proteins were uploaded into the Ingenuity Pathways (IPA; Ingenuity Systems, Redwood City, CA) Knowledge Base as a tab-delimited text file of IPI accession numbers. Biological networks were generated using their Knowledge Base for interactions between mapped Focus Genes (user's list) and all other gene objects stored in the knowledge base. In addition, functional analysis of the networks was performed to identify the biological functions and/or canonical pathway that were most significant to the genes in the network. The significance of functional enrichment was computed by a Fisher's exact test (p<0.05). A detailed description of IPA can be found on the Ingenuity Systems website.

### Immunoblotting

To validate talin-1 expression, 30 µg of proteins extracted from control and tumor tissues were separated by 4–15% SDS-PAGE and transferred onto nitrocellulose membrane (GE Healthcare). The nitrocellulose membrane was incubated with anti-talin-1 (1∶1000, Abcam), and anti-GAPDH (1∶5000, Bioethyl) specific antibodies for 2 h. After incubation with secondary antibodies, visualization of talin-1 and GAPDH was achieved by chemiluminescence with the ECL kit (Amersham Biosciences).

### Human tissue sample preparation

This study analyzed 12 pairs of fresh samples, each pair from the same patient, of OSCC and adjacent histologically normal oral mucosa. Fresh samples were divided into two parts: one was fixed in formalin and embedded in paraffin for hematoxylin and eosin staining and immunohistochemistry, while the other was immediately stored at −80°C for real-time quantitative PCR experiment. Before conducting the experiments, the frozen sections were stained with hematoxylin/eosin and evaluated by a pathologist. All of the tissue samples were collected from patients who had signed informed consent forms prior to participation in the study, which was approved by the Research Ethics Committee of the Piracicaba Dental School, University of Campinas, Brazil. After the diagnosis, all patients were referred to head and neck surgeons for treatment.

### Immunohistochemistry

Briefly, slides of normal oral mucosa and oral squamous cell carcinoma (n = 10) were incubated with monoclonal mouse anti-talin-1 (Abcam, Cambridge, MA, USA) diluted 1∶500 followed by the Advance detection system (Dako). The control reactions were performed by the exclusion of the primary antibodies. Talin-1 expression was assessed with the aid of the Aperio ScanScope CS and the ImageScope software (Aperio Technologies Inc., Vista, CA).

### Real-time quantitative PCR

Total RNA from 24 fresh tissues samples, 12 from OSCC and 12 from normal oral mucosa, was isolated with TRIzol reagent according to the manufacturer's protocol (Invitrogen). Following DNase I treatment, in order to eliminate genomic DNA contamination, 2 µg of total RNA per sample were used to generate cDNA using Oligo-dT (Invitrogen) and a superscript enzyme (Superscript II RT enzyme, Invitrogen). The resulting cDNAs were subjected to qRT-PCR using SYBR Green PCR Master Mix (Applied Biosystems) in the StepOnePlus Real Time PCR System (Applied Biosystems). Gene expressions were determined by the standard curve method with normalization to the housekeeping gene GAPDH. Primer sequences were to GAPDH 5′ GAAGGTGAAGGTCGGAGTC 3′ (forward) and 5′ GAAGATGGTGATGGGATTTC 3′ (reverse), to Talin-1 5′ CTGTATGTGCAGGCACGAGATGAC-3′ (forward) and 5′-AGCGGACCTTGGCCTCAATCTCA-3′ (reverse), to filamin A 5′-GATCACGGATCCCGAAGGCAAG-3′ (forward) and 5′- AATCTGAATGGTGGGGCCGATG-3′ (reverse), to catenin alpha-1 5′-GCCCAGCTAGCCGCAGAAATGA-3′ (forward) and 5′-TGCAGCCAAAACATGGGCCTTC-3′ (reverse), to filamin B 5′-AGCAGACGCCAAAGCAGAGG -3′ (forward) and 5′- TCAGGAGTGATGACCTGTGGGAC-3′ (reverse).

### Small interfering RNA transfection

For silencing of talin-1, SCC-9, A431 and SCC-9 LN1 cells were grown to a confluence of 40–50% and transfected with 50 nM Small Interfering RNA (siRNA) duplex (sc-36610, Santa Cruz) using Lipofectamine 2000. Random Stealth siRNA duplexes coding for nonfunctional RNAs served as control (sc-37007, Santa Cruz) and submitted to cell adhesion, migration and invasion assays. The SCC-9 cells were processed for immunoblotting and real-time quantitative PCR (three independent experiments were performed with three replicates) to confirm talin-1 knockdown. A431 cells and SCC-9 LN1 cells were also processed for real-time quantitative PCR (one independent experiment was performed with three replicates) to confirm talin-1 knockdown in these cells.

### Cell adhesion assay

SCC-9, A431 and SCC-9 LN1 cells transfected with control (scrambled) and siRNA against talin-1 were submitted to adhesion assay. Briefly, 3×10^5^ cells were plated in 6 well-plate and after 24 h the oligos (Scramble and against TLN-1, Santa Cruz) were transfected with lipofectamine 2000 according to the manufacturer instructions (Invitrogen). After 48 h of transfection, cells were trypsinised and seeded in a Matrigel (2 µg per well; BD Biosciences) coated 96-well plate, previously washed three times with PBS and blocked with 3% BSA (bovine serum albumin) during 2 h. The adhesion was evaluated during 1 h in serum-free media supplemented with 3% BSA, the wells were washed 3 times and cells were fixed with 10% formaldehyde. Cells were stained with 1% toluidine blue containing 1% borax for 5 min. The dye was eluted using 100 µl 1% SDS and the absorbance was measured at 620 nm. Three independent experiments were performed with triplicates for SCC-9, A431 and SCC-9 LN1 cells.

### Transwell migration assay

SCC-9, A431 and SCC-9 LN1 cells transfected with control siRNA and siRNA against talin-1 were plated in the upper chambers of 8 mm pore transwells (HTS Transwell-96 Well Plate, Corning). After 48 h of transfection, the cells were submitted to a starvation period of 4 h. The cells were allowed to migrate towards the lower chamber containing 1% FBS supplemented media. After 24 h, cells at the top chamber were removed with a cotton swab and the cells at the bottom of the insert filter were fixed with 10% formaldehyde for 10 min, washed with PBS and stained with 1% toluidine blue solution in 1% borax for 5 min. The dye was eluted using 1% SDS and the absorbance was measured at 620 nm. Three independent experiments were performed with triplicates.

### Cell invasion assay

For the invasion assay, SCC-9, A431 and SCC-9 LN1 cells transfected with control siRNA and siRNA against talin-1. After 48 h of transfection, the cells were submitted to a starvation period of 24 h. The cells were plated in the top chamber of the transwell (HTS Transwell-96 Well Plate, Corning) with a matrigel-coated polycarbonate membrane (BD matrigel, Basement Membrane Matrix) and the medium with 10% FBS was added to the lower chamber as a chemoattractant. After 72 h, cells on the lower surface of the membrane were fixed with 10% formalin and stained with 1% toluidine blue solution in 1% borax for 15 min. A cotton swab mechanically removed cells that did not migrate through the pores. The dye was eluted using 1% SDS and the absorbance was measured at 620 nm. Two independent experiments were performed with duplicates.

### Statistical analysis for validation experiments

For immunohistochemistry, qRT-PCR, cell adhesion and cell migration assays, statistical analyses were performed using Prism Statistics Software (GraphPad, La Jolla, CA) and the p-values<0.05 were set as statistically significant. Student's *t*-test was used after the assumptions for normality data verified by Kolmogorov–Smirnov test.

## Results

### An in vivo model for human squamous cell carcinoma development

**Figure 1:**
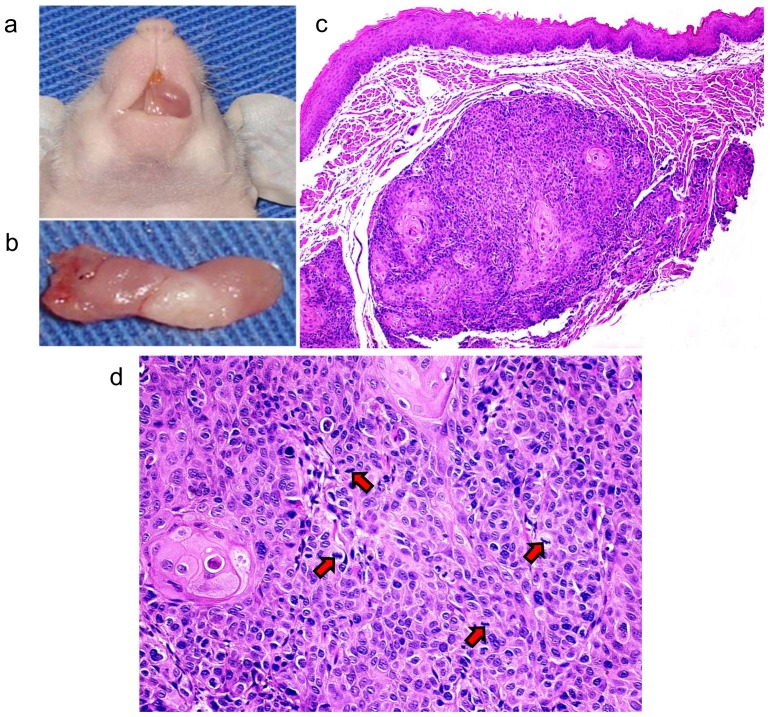
Development of OSCC in the tongue of an immunodeficient mouse. (a) Shows the tumor developed in the oral cavity of BALBc/nude mouse by SCC-9 cells injection. (b) Shows the tumor dissected from the oral cavity of the BALBc/nude mouse. (c) Microscopical features of the OSCC included pleomorphic epithelial cells invading the muscle fibers of the tongue (50X, H&E). (d) Mitotic figures (arrows) were also observed (200X, H&E).

Before characterizing the proteome difference between normal and tumor tissues, we confirmed the tumorigenicity of cells. As expected, SCC-9 cells were able to develop tumors in immunocompromised mice after 20 days (Figures 1a and 1b). Microscopically, the tumors were located in the connective tissue with no contact to the surface epithelium, invading the surrounding muscle fibers of the tongue (Figure 1c). Tumors were composed by pleomorphic epithelial cells, which exhibited mitotic figures (Figure 1d).

### MS-based proteomic and peptidomic analyses in control and tumor tissues obtained from the orthotopic murine model

We carried out a label-free proteomic and peptidomic analysis to quantify the proteins and peptides present in control and tumor tissues (Figure A in File S1). Briefly, the control and tumor tissues isolated from mouse tongue were extracted and for the proteome analysis, the extractions were digested by trypsin followed by peptide analysis on a high-resolution mass spectrometer. Since the tumor tissue is originated from human cells and the control tissue is originated from mouse tissues, only unique peptides that were present in both mouse and human databases were considered for the comparison of protein and peptide expression between control and tumor tissues (the only exception allowed was an exchange of isoleucine to leucine residues and vice-versa). The Fisher's exact test was used with a significance level at 5% (Tables C-G in File S1). The proteins and endogenous peptides with a fold-change of 2.0 that reached statistical significance (Fisher's exact test, p<0.05) were considered to be differentially expressed between control and tumor tissues (Table A in File S1 for identified proteins, and Table B in File S1 for identified endogenous peptides).

**Figure 2:**
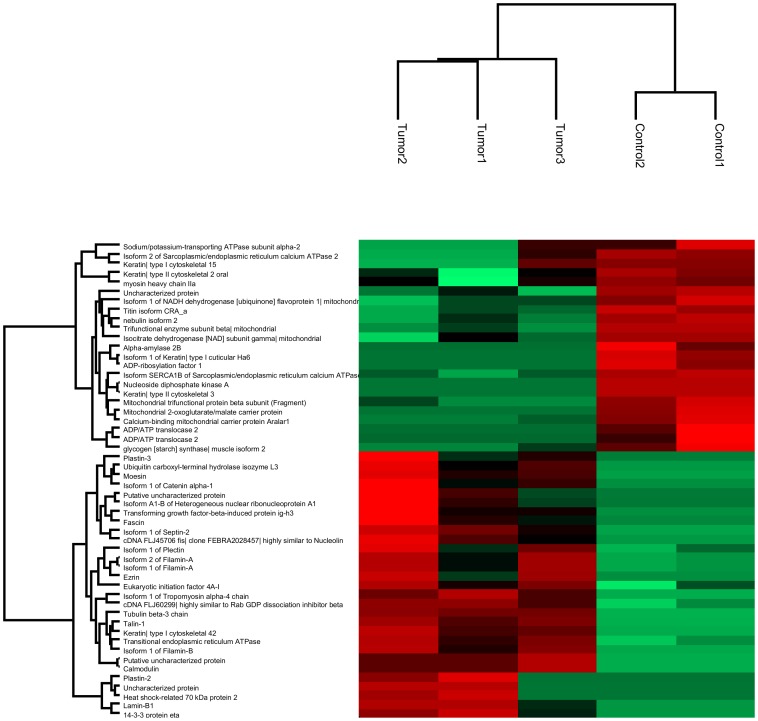
Bioinformatics analysis of differential expressed proteins in OSCC and control tissues identified by MS. Clustering of significantly up- and down-regulated proteins (Fisher's exact test, p<0.05) in OSCC tissues compared to control tissues is shown as a Heat map, applying the Euclidian distance method and average linkage.

After considering the unique peptides that were present in both databases, we identified 734 and 743 proteins against human and mouse databases, respectively (Tables C and D in File S1). We identified 29 up-regulated and 23 down-regulated proteins that were differentially expressed in tumor tissues compared with control tissues using a 2.0-fold-change cutoff (Table A in File S1, Fisher's exact test, p<0.05). Hierarchical clustering of significantly changing proteins was performed using the Z-score calculation on spectral counting values and represented as a heat map (Figure 2).

The endogenous cleaved peptides were evaluated in control and tumor tissues using CID, HCD and ETD fragmentation methods. As shown in Table B in File S1, CID and HCD fragmentation methods identified 14 and 6 down-regulated proteins, respectively, based on identification of endogenous peptides that were differentially expressed in tumor tissues compared with control tissues (Fisher's exact test, p<0.05). The endogenous protein fragments identified using ETD fragmentation did not show statistically significant differences between control and tumor tissues (Fisher's exact test, p>0.05). The fragments identified by CID and HCD fragmentation originated mainly from ribosomal proteins, histones, actin, transgelin-2, myosin and vimentin.

### Validation of the protein signature in OSCC tumor tissues using immunoblotting, qRT-PCR and immunohistochemistry

**Figure 3:**
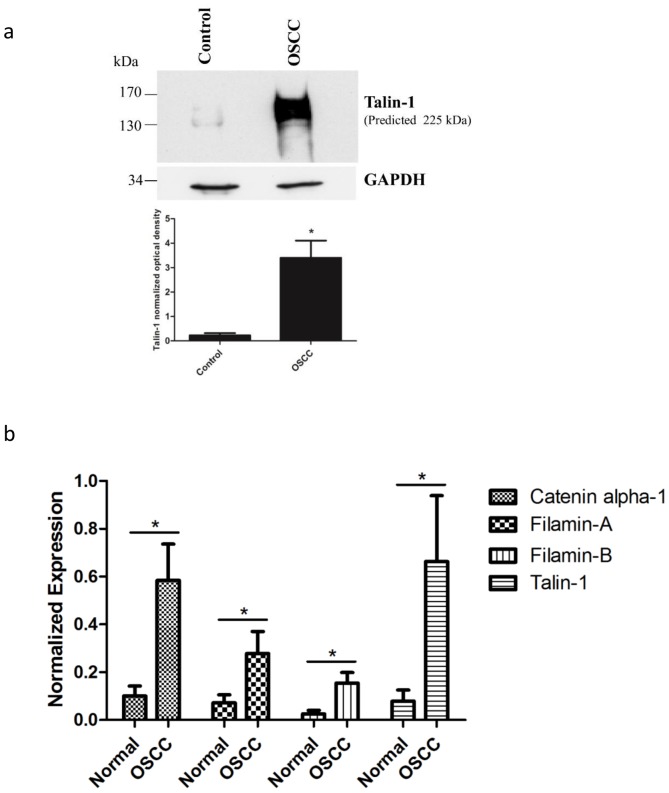
Talin-1 showed higher protein expression in OSCC tissues compared to control tissues in orthotopic murine model by immunoblotting (a). The proteins (30 µg) were submitted to 1-D electrophoresis on 4–15% SDS-polyacrylamide gels, they were transferred onto nitrocellulose membrane and incubated with anti-talin-1 antibody. Anti-GAPDH antibody was used as loading control. The graph represents the normalized optical density of the average data of three immunoblotted samples (n = 3, * indicates p<0.05, Student's *t*-test). Talin-1, filamins A and B and catenin alpha-1 showed higher mRNA expression levels in human OSCC tumor tissues compared to control tissues by qRT-PCR (n = 12) (b). Relative mRNA expression levels were measured by the real-time quantitative PCR. The data were normalized with glyceraldehyde-3- phosphate dehydrogenase gene. Columns represent mean and SD (n = 12; Student's *t*-test, * indicates p<0.05).

**Figure 4:**
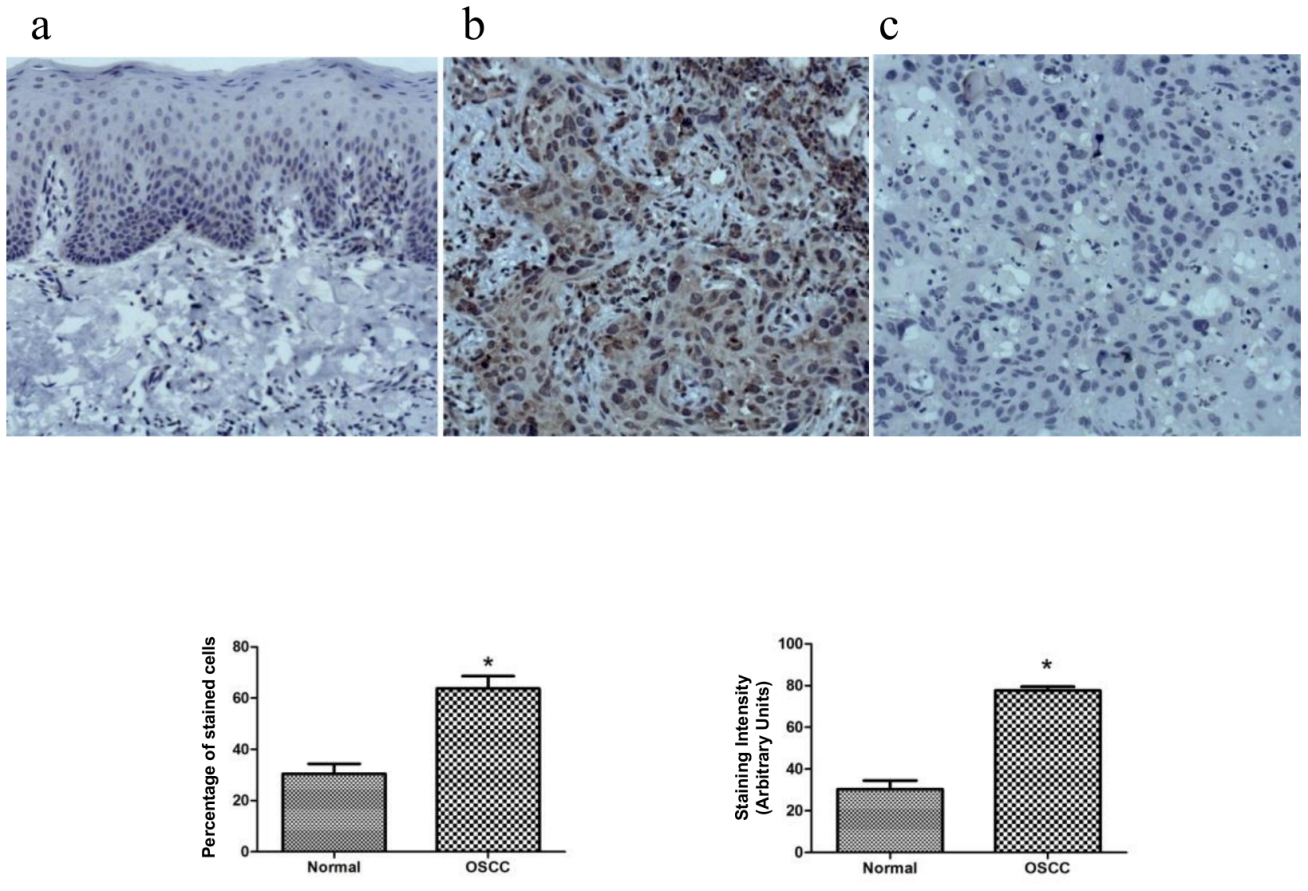
Expression of talin-1 in the human normal oral mucosa and OSCC tissues by immunohistochemistry (n = 10). Talin-1 demonstrated a weak and restrict cytoplasmic immunoreactivity in the basal and suprabasal layers of the normal oral tissue (a), whereas broad positivity with variable intensity was found in the neoplastic cells (b). As expected, some immune and inflammatory cells expressed talin-1. Panel c represents the negative control. The graphics represent the % of positive expression cells and expression intensity between the normal and OSCC tumor tissues (n = 10, Student's *t*-test, * indicates p<0.05).

Among the identified proteins, we first validated by immunoblotting the higher expression of talin-1 in tumor tissues compared with control tissues obtained from the orthotopic murine model (Figure 3a, Student's *t*-test, p<0.05). We also validated the higher expression of talin-1, catenin alpha-1, filamin A and filamin B in human OSCC by qRT-PCR (Figure 3b, Student's *t*-test, p<0.05). By immunohistochemistry, we validated the higher expression of talin-1 (Students' *t*-test, p<0.0001) in human OSCC compared with human normal oral mucosa (Figure 4). The clinicopathological variables of OSCC patients are shown in Table J in File S1.

### Biological network analysis reveals different biological processes for up-regulated and down-regulated proteins in tumor tissues

To further characterize the identified proteins, we mapped the pathway annotations and the functional relationships of the differentially expressed proteins identified by MS. The enrichment GO terms revealed significant biological processes involved in actin cytoskeleton organization and cell-cell junction assembly for up-regulated proteins (p<0.05, Table 1) and energetic metabolism and cellular respiration for down-regulated proteins (p<0.05, Table 1). The complete results are shown in the Tables H and I in File S1.

**Table 1:** List of the significant Gene Ontology annotation enriched in the differentially expressed proteins using DAVID Gene Functional Classification Tool.

Functional Annotation	p-value	Proteins (gene name)
**Up-regulated proteins**		
GO:0030036: actin cytoskeleton organization	8.93E-06	Ezrin (EZR), Plastin-3 (PLS3), Talin-1 (TLN1), Fascin (FSCN1), Isoform 1 of Filamin-B (FLNB) Isoform 2 of Filamin-A (FLNA)
GO:0008104: protein localization	7.10E-04	Rab GDP dissociation inhibitor beta (GDI2), Ezrin (EZR), Transitional endoplasmic reticulum ATPase (VCP), Talin-1 (TLN1), 14-3-3 protein eta (YWHAH), Isoform 1 of Filamin-B (FLNB) Isoform 2 of Filamin-A (FLNA)
GO:0022614: membrane to membrane docking	0.006	Ezrin (EZR), Moesin (MSN)
GO:0007043: cell-cell junction assembly	0.023	Isoform 1 of Catenin alpha-1 (CTNNA1), Talin-1 (TLN1)
GO:0007159: leukocyte adhesion	0.036	Ezrin (EZR), Moesin (MSN)
**Down-regulated proteins**		
GO:0015980: energy derivation by oxidation of organic compounds	2.42E-04	Isocitrate dehydrogenase [NAD] (IDH3G), Isoform 1 of NADH dehydrogenase [ubiquinone] flavoprotein 1| mitochondrial (NDUFV1), glycogen [starch] synthase| muscle isoform 2 (GYS1), Calcium-binding mitochondrial carrier protein Aralar1 (SLC25A12)
GO:0045333: cellular respiration	0.003	Isocitrate dehydrogenase [NAD] (IDH3G), Isoform 1 of NADH dehydrogenase [ubiquinone] flavoprotein 1| mitochondrial (NDUFV1), Calcium-binding mitochondrial carrier protein Aralar1 (SLC25A12)
GO:0055085: transmembrane transport	0.012	Isoform SERCA1B of Sarcoplasmic/endoplasmic reticulum calcium ATPase 1 (ATP2A1), ADP/ATP translocase 2 (SLC25A5) Mitochondrial 2-oxoglutarate/malate carrier protein (SLC25A11), Calcium-binding mitochondrial carrier protein Aralar1 (SLC25A12)

**Figure 5:**
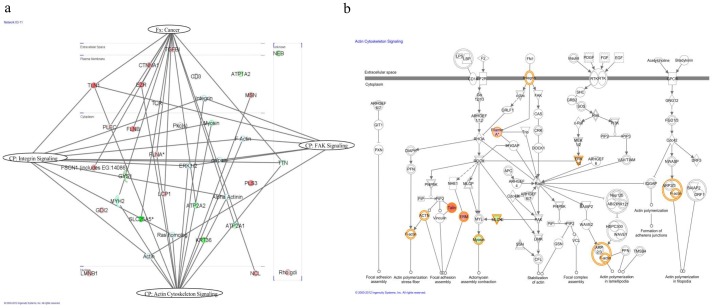
Pathway analysis of differentially expressed proteins. (a) The highest score network generated by IPA comprised 23 differentially expressed proteins (up-regulated proteins are displayed in red, whereas the down-regulated **proteins** are in green) plus additional interacting molecules that were not identified in this study (white). The network revealed 15 proteins in the context of cancer. Actin cytoskeleton signaling, integrin signaling and FAK signaling represent canonical pathways with 13, 10 and 6 identified proteins of the network, respectively. The proteins were grouped according to the canonical pathway or function. Fx: function and Cp: canonical pathway. (b) Representation of the top canonical pathway of actin cytoskeleton signaling.

We also examined functional pathway enrichment in the differentially expressed proteins by using Ingenuity Pathway Analysis (IPA). Of the 52 query molecules, 43 were eligible for network analysis (focus molecule) based on the IPA Knowledge Base criteria. The top network was selected and included 23 of the 43 focus molecules (score 61). The network revealed 15 proteins in the context of cancer (gene names: FSCN1, MYH2, SLC24A5, LCP1, NCL, ATP2A2, ATP2A1, PLEC, FLNA, FLNB, TTN, TLN1, EZR, CTNNA1, TGFB1) at p = 3.09×10^−5^. Pathways derived from the network are shown in the Figure 5a, such as actin cytoskeleton signaling (p = 2.27×10^−5^), integrin signaling (p = 1.62×10^−2^) and FAK signaling (p = 2.1×10^−1^). The top canonical signaling pathway (p = 2.27×10^−5^), actin cytoskeleton signaling, is displayed in Figure 5b.

### Talin-1 knockdown decreased cell adhesion, migration and invasion

**Figure 6:**
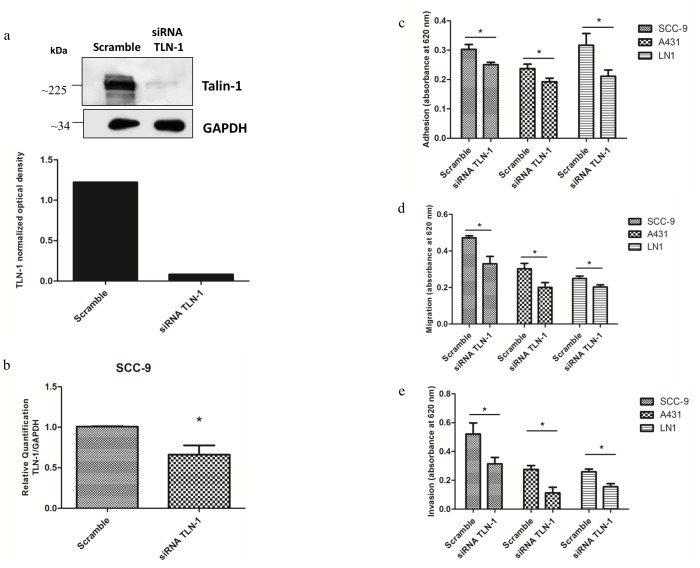
Talin-1 knockdown decreased cell adhesion, migration and invasion of SCC-9, A431 and SCC-9 LN1 cells. (a) Talin-1 showed lower protein expression in SCC-9/siRNA TLN-1 cells compared to SCC-9/control (scrambled) by immunoblotting. The total proteins (30 µg) were submitted to 1-D electrophoresis on 12% SDS-polyacrylamide gels, they were transferred onto nitrocellulose membrane and incubated with anti-talin-1 antibody. Anti-GAPDH antibody was used as loading control. The graph represents the normalized optical density. (b) Talin-1 mRNA expression levels in SCC-9/siRNA TLN-1 cells compared to SCC-9/control (scrambled) by qRT-PCR (n = 3, Student's *t*-test, p<0.05). The data were normalized with GAPDH gene. (c) SCC-9/control (scrambled) and SCC-9/siRNA TLN-1 cells, A431/control (scrambled) and A431/siRNA TLN-1 cells, SCC-9 LN1/control (scrambled) and SCC-9 LN1/siRNA TLN-1 cells were seeded in Matrigel coated 96-well plates. After 1 h, cells were stained and the cell adhesion was measured (**n = 3**, * indicates p<0.05, Student's *t*-test for each comparison). (d) SCC-9/control (scrambled) and SCC-9/siRNA TLN-1 cells, A431/control (scrambled) and A431/siRNA TLN-1 cells, SCC-9 LN1/control (scrambled) and SCC-9 LN1/siRNA TLN-1 cells were seeded in serum-free media in the upper chamber of transwell plates and were allowed to migrate towards the lower chamber containing 1% FBS supplemented media (n = 3, * indicates p<0.05, Student's *t*-test). (e) SCC-9/control (scrambled) and SCC-9/siRNA TLN-1 cells, A431/control (scrambled) and A431/siRNA TLN-1 cells, SCC-9 LN1/control (scrambled) and SCC-9 LN1/siRNA TLN-1 cells were seeded in serum-free media in the upper chamber of matrigel-coated transwell plates and were allowed to invade towards the lower chamber containing 10% FBS supplemented media (n = 2, * indicates p<0.05, Student's *t*-test).

SCC-9, A431, and SCC-9 LN1 cells treated with control siRNA and siRNA against talin-1 were evaluated in cell adhesion assay in matrigel and in migration assay in 96-well transwell plates. Knockdown of talin-1 is confirmed by immunoblotting in SCC-9 cells (Figure 6a) and by qRT-PCR in SCC-9 (Figure 6b), A431 and SCC-9 LN1 cells (Figure B in File S1). As observed in Figure 6c, talin-1 knockdown decreased adhesion of SCC-9, A431, SCC-9 LN1 cells compared to control siRNA (n = 3, Student's *t*-test, p<0.05). In migration, SCC-9 cells treated with control siRNA and siRNA against talin-1 were seeded in 96-well transwell plates in the upper chambers containing serum-free media. The cells were allowed to migrate towards the lower chamber containing 1% FBS supplemented media. After 24 h, migration to the lower chamber was measured by colorimetric assay. SCC-9, A431 and SCC-9 LN1 cells treated with control siRNA against talin-1 showed a decrease in migration (Figure 6d, n = 3, Student's *t*-test, p<0.05). In invasion assay, SCC-9, A431 and SCC-9 LN1 cells treated with control siRNA and siRNA against talin-1 were seeded in transwell plates in the upper chambers containing serum-free media. The cells were allowed to invade towards the lower chamber containing 10% FBS supplemented media. After 72 h, invasion to the lower chamber was measured by colorimetric assay. SCC-9, A431 and SCC-9 LN1 cells treated with siRNA against talin-1 showed a decrease in invasion (Figure 6e, n = 2, Student's *t*-test, p<0.05).

## Discussion

OSCC is the most common malignant tumor of the oral cavity [19], but the molecular mechanisms and factors that lead to the cancerous transformation of normal oral mucosa are not well understood. Proteomic analysis allows us to evaluate the dynamic changes of protein patterns that occur in the tumor to better understand the pathogenesis and contribute to the discovery of oral cancer associated proteins. We applied proteomic label-free approaches in OSCC using an orthotopic murine model of tongue squamous cell carcinoma, which mimics both local tumor growth and invasion and the process of metastatic spread to the cervical lymph nodes [20]–[22].

### Focal adhesion-mediated proteins were found to be up-regulated in OSCC tissues

Recently, it has been reported that multiple cancer cell lines display degradation of the extracellular matrix (ECM) at focal adhesion sites, thereby contributing to two essential steps in the metastatic process: tumor cell migration and the concomitant degradation of the ECM [23]. In fact, we found several proteins possibly involved in actin cytoskeleton organization and cell-cell junction assembly, including talin-1, ezrin, plastin-3, fascin, catenin alpha-1, filamins A and B to be up-regulated in tumor tissues compared to control tissues (Table 1, Figure 5 and Table A in File S1). Among the proteins involved in statistically significant biological processes and functional pathways (Table 1 and Figure 5, p<0.05) a set of proteins that mediate focal adhesion and were concomitantly identified up-regulated in OSCC, such as talin-1, catenin alpha-1, filamins A and B were chosen for further validation.

Talin-1 demonstrated the highest fold-change in tumor tissues (Table A in File S1, Fisher's exact test, p<0.05) and its expression was validated by immunoblotting (Figure 3a, Student's *t*-test, p<0.05), qRT-PCR (Figure 3b, Student's *t*-test, p<0.05) and immunohistochemistry in OSCC human tissues (Figure 4, Students' *t*-test, p<0.0001). This protein is considered a key adaptor protein able to regulate integrin conformation and cell migration [24]. Cell junction proteins such as catenin alpha-1 and filamins A and B were also found to be up-regulated in our MS study (Table A in File S1, Fisher's exact test, p<0.05). These results were validated by qRT-PCR in OSCC human tissues (Figure 3b, Students' *t*-test, p<0.0001).

GO enrichment analysis showed that catenin alpha-1 and filamins A and B are involved in cell-cell junction assembly and actin cytoskeleton organization, respectively (Table 1). As expected, the significant pathways related to actin cytoskeleton signaling and integrin signaling are linked to cancer, and the network wiring proteins recapitulate the dynamic protein signaling that can be involved in cancer development (Figure 5a). Actin cytoskeleton signaling as the top canonical pathway (p-value = 2.57×10^−5^) reflects the major event underscored by this study (Figure 5b). Catenin alpha-1 binds not only to E-cadherin-beta-catenin but also to actin filaments. Thus, it may play a role in local regulation of actin assembly and organization at sites of cadherin-mediated cell-cell adhesion [25].

Interestingly, filamin A and talin-1 have also been shown to play roles in actin-membrane assembly as well as cell-cell junction maintenance. Filamins are able to connect several transmembrane and signaling proteins to actin, allowing the assembly of complex networks. Talin-1 and filamins bind to integrin adhesion receptor in the same position and affect integrin activation by competing for binding to integrin [26]. In fact, the network of actin cytoskeleton signaling revealed proteins named filamin, focal adhesion kinase (FAK) and paxilin (Figure 5b). Interestingly, these proteins play a role in cell motility, proliferation and survival [23], all essential pathways for cancer development. Filamin A, for example, has been reported as a target for DNA damage-based cancer therapy [27]. The fact that filamin A acts in DNA damage repair suggests that the lack of filamin A confers cancer cells more sensitive to DNA damage treatment and allows better prognosis [27].

The adhesion proteins organize the epithelial cell-cell junction and the actin cytoskeleton, which is considered to be a stable structure that maintains the structural integrity of tissues, a critical feature that is affected during cancer development [23]. In order to investigate the functional relevance of talin-1 in OSCC adhesion, migration and invasion, we induced a knockdown of talin-1 in SCC-9 cells (Figure 6a and b). In fact, we observed a decrease in cell adhesion and migration (Figure 6c and 6d), which strengthen the role talin-1 might play in oral cancer development. We also investigated the importance of talin-1 for invasion, and we observed a decrease in the invasion in talin-1 knockdown cells (Figure 6e). To demonstrate that this role is not cell specific but rather independent of the carcinoma cell type, we also induced knockdown of talin-1 in A431 and SCC-9 LN1 cell lines (Figure B in File S1) and observed a decrease in cell adhesion, migration and invasion (Figure 6c, 6d and 6e).

A previous study demonstrated that the genetic gain and overexpression of talin-1 in OSCC correlated with a poor clinical outcome [24]. They further evaluated the effect of dominant-negative mutant, which is able to decrease integrin activation, in low and high talin-1 expressing cells. Interestingly, a significant reduction of cell growth and invasiveness was observed only in high talin-1 expressing cells. Therefore, together with our data, we can suggest that talin-1 might have a role in OSCC development and it can be considered a potential therapeutic target in OSCC.

### Down-regulated proteins in OSCC reflect metabolic changes

Another characteristic of cancer was evidenced in our study; we observed a clear down-regulation of key enzymes involved in energy metabolism (Table 1 and Table A in File S1). The rearrangement in energy metabolism largely towards glycolysis is known to be one of the adjustments cancerous cells undergo to grow and divide [28]. Even in the presence of oxygen, cancer cells can reprogram their metabolism towards glycolysis, as opposed to mitochondrial oxidative phosphorylation, by regulating specific metabolic enzymes and allowing a greater intake of glucose by the cell. This aerobic glycolysis is considered an emerging hallmark of cancer associated with activated oncogenes such as Ras [29]–[31]. Our results identified the lower expression of mitochondrial proteins involved in energy metabolism in the tumor compared to the control tissues. This deregulation is possibly due to the accentuated use of glycolysis by oral cancer cells. The effect observed in the metabolic regulation pathway is a selective advantage for tumor cells because it allows cancer cells to proliferate and survive in an environment that fluctuates in nutrients and oxygen [30], [32].

### Lower abundance of protein fragments in OSCC orthotopic murine model is revealed by peptidomics-degradomics

To further explore the OSCC tissues, the endogenous cleavage peptides resulted from proteolysis and/or degradation were identified using complementary MS approaches to improve identification rates for peptidomic-degradomic analyses [33]. Interestingly, we observed a decrease in endogenous cleavage peptides in tumor tissues when the same amount of extracted proteins from the tissues was precipitated (Table B in File S1). Among the proteins, we found fragments of proteins originated from actin, transgelin-2, myosin regulatory light chain 2 and vimentin. Not only their fragments were identified in a similar fashion but also the proteins have a close relationship with actin cytoskeleton and focal adhesion dynamics [34], [35]. However, the primary complex mechanisms that direct the proteolysis and/or protein degradation in tumor tissues are still not clear, and there are several new frontiers to be overcome in this field to explain the balance between synthesis and degradation [36]–[38].

In summary, our study captures specific processes that are altered upon oral tumorigenesis and highlights the parallel up-regulation of cell junction proteins. Identifying underlying mechanisms that regulate the cell adhesion proteins may lead to more comprehensive understanding of oral cancer development and open novel avenues for OSCC-targeting approaches.

## Supporting Information

File S1 Contains the following Supporting Information files: **Table A**: Up-regulated and down-regulated proteins identified by LC-MS/MS according to the number of unique peptides Only statistically significant proteins are shown in this table (Fisher's exact test, p<0.05). **Table B**: Endogenous peptides identified by LC-MS/MS according to the number of unique peptides. Only statistically significant proteins are shown in this table (Fisher's exact test, p<0.05). **Table C**: List of total proteins with the number of unique peptides found in both Human and Mouse Databases after searching against Human database. For the comparison of the expression of proteins and peptides between control and tumor tissues, the Fisher's exact test was applied at significance level at 5%. **Table D**: List of total proteins with the number of unique peptides found in both Human and Mouse Databases after searching against Mouse database. For the comparison of the expression of proteins and peptides between control and tumor tissues, the Fisher's exact test was applied at significance level at 5%. **Table E**: List of total proteins with the number of unique endogenous peptides obtained from CID fragmentation method. For the comparison of the expression of proteins and peptides between control and tumor tissues, the Fisher's exact test was applied at significance level at 5%. **Table F**: List of total proteins with the number of unique endogenous peptides obtained from HCD fragmentation method. For the comparison of the expression of proteins and peptides between control and tumor tissues, the Fisher's exact test was applied at significance level at 5%. **Table G**: List of total proteins with the number of unique endogenous peptides obtained from ETD fragmentation method. For the comparison of the expression of proteins and peptides between control and tumor tissues, the Fisher's exact test was applied at significance level at 5%. **Table H**: Functional Annotation Chart for the Up-regulated Proteins using DAVID Gene Functional Classification Tool. **Table I**: Functional Annotation Chart for the Down-regulated Proteins using DAVID Gene Functional Classification Tool. **Table J**: Clinicopathological variables of OSCC patients. **Figure A**: Experimental workflow of the proteomic, peptidomic and bioinformatic analyses, and validation performed in this study. **Figure B**: Talin-1 mRNA expression levels in (a) A431/siRNA TLN-1 cells compared to A431/control (scrambled) and (b) in SCC-9 LN1/siRNA TLN-1 cells compared to SCC-9 LN1/control (scrambled) (n = 1, with three replicates). The data were normalized with GAPDH gene.
(DOC)
