# Supplementary material for: Integrated Proteomics Identified Up-Regulated Focal Adhesion-Mediated Proteins in Human Squamous Cell Carcinoma in an Orthotopic Murine Model
Source: PLoS One. 2014 May 23;9(5):e98208. doi: 10.1371/journal.pone.0098208 (PMC4032327; doi:10.1371/journal.pone.0098208)
Supplement: File S1 — Contains the following Supporting Information files: Table A: Up-regulated and down-regulated proteins identified by LC-MS/MS according to the number of unique peptides Only statistically significant proteins are shown in this table (Fisher's exact test, p<0.05). Table B: Endogenous peptides identified by LC-MS/MS according to the number of unique peptides. Only statistically significant proteins are shown in this table (Fisher's exact test, p<0.05). Table C: List of total proteins with the number of unique peptides found in both Human and Mouse Databases after searching against Human database. For the comparison of the expression of proteins and peptides between control and tumor tissues, the Fisher's exact test was applied at significance level at 5%. Table D: List of total proteins with the number of unique peptides found in both Human and Mouse Databases after searching against Mouse database. For the comparison of the expression of proteins and peptides between control and tumor tissues, the Fisher's exact test was applied at significance level at 5%. Table E: List of total proteins with the number of unique endogenous peptides obtained from CID fragmentation method. For the comparison of the expression of proteins and peptides between control and tumor tissues, the Fisher's exact test was applied at significance level at 5%. Table F: List of total proteins with the number of unique endogenous peptides obtained from HCD fragmentation method. For the comparison of the expression of proteins and peptides between control and tumor tissues, the Fisher's exact test was applied at significance level at 5%. Table G: List of total proteins with the number of unique endogenous peptides obtained from ETD fragmentation method. For the comparison of the expression of proteins and peptides between control and tumor tissues, the Fisher's exact test was applied at significance level at 5%. Table H: Functional Annotation Chart for the Up-regulated Proteins using DAVID Gene Fun [file pone.0098208.s001.doc]

| ***Supporting Information: TABLES***  **Table A** | | | | | | | | |
| --- | --- | --- | --- | --- | --- | --- | --- | --- |
| **Protein accession number** | **Protein name** | **Control 1** | **Control 2** | **Tumor 1** | **Tumor 2** | **Tumor 3** | **Fold-change** | **Fisher’s exact test** |
| **Up-regulated proteins** | | | | | | | | |
| IPI00216319, IPI00227392 | 14-3-3 protein eta | 0 | 0 | 6 | 5 | 2 | 5.33 | 2.17E-03 |
| IPI00075248, IPI00916600 | Calmodulin | 0 | 0 | 3 | 3 | 4 | 4.33 | 5.00E-02 |
| IPI00444262, IPI00604620  IPI00317794 | cDNA FLJ45706 fis| clone FEBRA2028457| highly similar to Nucleolin | 0 | 0 | 3 | 5 | 2 | 4.33 | 1.79E-02 |
| IPI00031461,IPI00940148  IPI00323179 | cDNA FLJ60299| highly similar to Rab GDP dissociation inhibitor beta | 2 | 1 | 6 | 6 | 5 | 2.67 | 3.17E-02 |
| IPI00025491, IPI00118676 | Eukaryotic initiation factor 4A-I | 5 | 2 | 7 | 10 | 9 | 2.15 | 4.47E-02 |
| IPI00843975, IPI00330862 | Ezrin | 0 | 0 | 2 | 8 | 7 | 6.67 | 2.22E-02 |
| IPI00163187, IPI00353563 | Fascin | 0 | 0 | 3 | 7 | 2 | 5.00 | 8.33E-03 |
| IPI00331546 | Heat shock-related 70 kDa protein 2 | 0 | 0 | 8 | 7 | 0 | 6.00 | 1.55E-04 |
| IPI00215948,IPI00980391,IPI00981271, IPI00112963 | Isoform 1 of Catenin alpha-1 | 0 | 0 | 2 | 6 | 3 | 4.67 | 3.57E-02 |
| IPI00131138, IPI00875567 | Isoform 1 of Filamin-A | 6 | 5 | 13 | 23 | 22 | 3.13 | 6.39E-04 |
| IPI00289334,IPI00382697,IPI00477536,IPI00900293,IPI00943563,IPI00953109,IPI00663627 | Isoform 1 of Filamin-B | 0 | 0 | 4 | 7 | 6 | 6.67 | 3.03E-03 |
| IPI00014898,IPI00186711,IPI00398002,IPI00398775,IPI00398776,IPI00398777,IPI00398778,IPI00398779, IPI00420096 | Isoform 1 of Plectin | 13 | 9 | 16 | 30 | 24 | 2.03 | 6.87E-03 |
| IPI00014177,IPI00871851,  IPI00114945 | Isoform 1 of Septin-2 | 0 | 0 | 3 | 4 | 2 | 4.00 | 2.86E-02 |
| IPI0001079 | Isoform 1 of Tropomyosin alpha-4 chain | 0 | 0 | 10 | 8 | 7 | 9.33 | 2.29E-05 |
| IPI00302592,IPI00333541,  IPI00131138,IPI00875567 | Isoform 2 of Filamin-A | 6 | 5 | 13 | 23 | 22 | 3.13 | 6.39E-04 |
| IPI00215965,IPI00465365,IPI00797148,IPI01021093,IPI01021324,IPI01022801 | Isoform A1-B of Heterogeneous nuclear ribonucleoprotein A1 | 1 | 1 | 4 | 8 | 2 | 2.83 | 2.30E-02 |
| IPI00468696 | Keratin| type I cytoskeletal 42 | 0 | 0 | 8 | 12 | 9 | 10.67 | 7.94E-06 |
| IPI00217975 | Lamin-B1 | 0 | 0 | 3 | 3 | 1 | 3.33 | 5.00E-02 |
| IPI00219365, IPI00110588 | Moesin | 1 | 1 | 6 | 11 | 7 | 4.50 | 1.69E-03 |
| IPI00118892 | Plastin-2 | 0 | 0 | 4 | 3 | 0 | 3.33 | 2.86E-02 |
| IPI00216694,IPI00947227,IPI01011191, IPI00115528,IPI00776023 | Plastin-3 | 0 | 0 | 2 | 10 | 4 | 6.33 | 1.52E-02 |
| IPI00467841,IPI00761696 | Putative uncharacterized protein | 0 | 0 | 3 | 3 | 4 | 4.33 | 5.00E-02 |
| IPI00553777,IPI00817004 | Putative uncharacterized protein | 1 | 1 | 5 | 9 | 2 | 3.17 | 7.62E-03 |
| IPI00298994,IPI00465786 | Talin-1 | 4 | 4 | 33 | 42 | 38 | 7.73 | 2.09E-14 |
| IPI00018219,IPI00940091,IPI00965868, IPI00122528,IPI00991018 | Transforming growth factor-beta-induced protein ig-h3 | 0 | 0 | 2 | 5 | 2 | 4.00 | 4.76E-02 |
| IPI00022774,IPI00622235 | Transitional endoplasmic reticulum ATPase | 14 | 11 | 24 | 31 | 28 | 2.12 | 8.76E-04 |
| IPI00013683 | Tubulin beta-3 chain | 0 | 0 | 12 | 13 | 12 | 13.33 | 1.92E-07 |
| IPI00011250 | Ubiquitin carboxyl-terminal hydrolase isozyme L3 | 0 | 0 | 2 | 5 | 3 | 4.33 | 4.76E-02 |
| IPI00122312,IPI00990997 | Uncharacterized protein | 0 | 0 | 3 | 3 | 0 | 3.00 | 5.00E-02 |
| **Down-regulated proteins** | | | | | | | | |
| IPI00007188 | ADP/ATP translocase 2 | 9 | 5 | 2 | 2 | 2 | 0.38 | 3.87E-02 |
| IPI00127841 | ADP/ATP translocase 2 | 11 | 6 | 0 | 0 | 0 | 0.11 | 8.08E-05 |
| IPI00215914,IPI00215917  IPI00221613,IPI00221614 | ADP-ribosylation factor 1 | 3 | 4 | 0 | 0 | 0 | 0.22 | 2.86E-02 |
| IPI00021447,IPI00025476,IPI00300786,IPI01010056,IPI00315893 | Alpha-amylase 2B | 3 | 5 | 0 | 0 | 0 | 0.20 | 1.79E-02 |
| IPI00386271,IPI00308162 | Calcium-binding mitochondrial carrier protein Aralar1 | 12 | 9 | 1 | 1 | 2 | 0.20 | 1.15E-04 |
| IPI00157144,IPI00303868  IPI00130127,IPI00856819 | glycogen [starch] synthase| muscle isoform 2 | 5 | 3 | 0 | 0 | 1 | 0.27 | 1.79E-02 |
| IPI00220150,IPI01011396  IPI00109169,IPI00988842 | Isocitrate dehydrogenase [NAD] subunit gamma| mitochondrial | 8 | 8 | 5 | 1 | 3 | 0.44 | 2.47E-02 |
| IPI00008692,IPI00676959 | Isoform 1 of Keratin| type I cuticular Ha6 | 4 | 5 | 0 | 0 | 0 | 0.18 | 7.94E-03 |
| IPI00028520,IPI00221298,IPI00978187,IPI00130460,IPI00928416 | Isoform 1 of NADH dehydrogenase [ubiquinone] flavoprotein 1| mitochondrial | 11 | 9 | 4 | 1 | 4 | 0.36 | 2.71E-03 |
| IPI00177817,IPI00219078,IPI00747443,IPI00969023 | Isoform 2 of Sarcoplasmic/endoplasmic reticulum calcium ATPase 2 | 13 | 14 | 0 | 0 | 9 | 0.28 | 4.99E-08 |
| IPI00024804,IPI00311654 | Isoform SERCA1B of Sarcoplasmic/endoplasmic reticulum calcium ATPase 1 | 26 | 25 | 7 | 11 | 11 | 0.40 | 6.08E-05 |
| IPI00290077 | Keratin| type I cytoskeletal 15 | 8 | 8 | 0 | 0 | 7 | 0.37 | 7.77E-05 |
| IPI00346834 | Keratin| type II cytoskeletal 2 oral | 9 | 10 | 0 | 5 | 6 | 0.44 | 1.53E-03 |
| IPI00290857,IPI00973210 | Keratin| type II cytoskeletal 3 | 8 | 8 | 0 | 0 | 0 | 0.11 | 7.77E-05 |
| IPI00219729,IPI00945233  IPI00230754 | Mitochondrial 2-oxoglutarate/malate carrier protein | 4 | 3 | 0 | 0 | 0 | 0.22 | 2.86E-02 |
| IPI00383695 | Mitochondrial trifunctional protein beta subunit (Fragment) | 5 | 4 | 0 | 1 | 0 | 0.24 | 2.38E-02 |
| IPI00553454 | myosin heavy chain IIa | 86 | 93 | 0 | 61 | 66 | 0.48 | 2.58E-25 |
| IPI00917728 | nebulin isoform 2 | 50 | 47 | 24 | 10 | 15 | 0.35 | 7.00E-09 |
| IPI00131459 | Nucleoside diphosphate kinase A | 3 | 3 | 0 | 0 | 0 | 0.25 | 5.00E-02 |
| IPI00420569,IPI00762871 | Sodium/potassium-transporting ATPase subunit alpha-2 | 7 | 4 | 0 | 0 | 4 | 0.36 | 3.03E-03 |
| IPI00940872,IPI00756257 | Titin isoform CRA_a | 150 | 166 | 69 | 33 | 57 | 0.34 | 5.66E-28 |
| IPI00022793,IPI01018954  IPI00115607 | Trifunctional enzyme subunit beta| mitochondrial | 4 | 4 | 1 | 0 | 0 | 0.27 | 3.97E-02 |
| IPI00379424 | Uncharacterized protein | 32 | 30 | 14 | 6 | 0 | 0.24 | 1.49E-06 |

Non-underlined and underlined IPI accession numbers are originated from Human and Mouse databases, respectively. Only statistically significant proteins are shown in this table (Fisher’s exact test, p<0.05).

| **Table B** | | | | | | | | |
| --- | --- | --- | --- | --- | --- | --- | --- | --- |
| **Protein accession number** | **Protein name** | **Control 1** | **Control 2** | **Control 3** | **Tumor 1** | **Tumor 2** | **Fold-change** | **Fisher’s exact test** |
| **CID fragmentation method** | | | | | | | | |
| IPI00012750, IPI00137735 | 40S ribosomal protein S25 | 3 | 3 | 3 | 0 | 0 | 0.25 | 5.00E-02 |
| IPI00013415  IPI00136984,IPI00850934 | 40S ribosomal protein S7 | 4 | 3 | 2 | 0 | 0 | 0.25 | 2.86E-02 |
| IPI00000494  IPI00308706,IPI00988023 | 60S ribosomal protein L5 | 3 | 4 | 2 | 0 | 0 | 0.25 | 2.86E-02 |
| IPI00021439,IPI00021440  IPI00110850,IPI00874482 | Actin| cytoplasmic 1 | 4 | 3 | 0 | 0 | 0 | 0.30 | 2.86E-02 |
| IPI00217469 | Histone H1.1 | 12 | 10 | 6 | 5 | 0 | 0.34 | 3.56E-04 |
| IPI00217465,IPI00223713 | Histone H1.2 | 10 | 15 | 10 | 4 | 4 | 0.39 | 5.19E-03 |
| IPI00331597 | Histone H1.3 | 14 | 17 | 11 | 6 | 4 | 0.40 | 1.25E-03 |
| IPI00255316 | Histone H2A type 1-D | 6 | 4 | 3 | 1 | 0 | 0.28 | 1.52E-02 |
| IPI00216457  IPI00330000,IPI00974916,IPI00989397 | Histone H2A type 2-A | 4 | 8 | 1 | 1 | 0 | 0.28 | 6.99E-03 |
| IPI00020101,IPI00282266 | Histone H2B type 1-C/E/F/G/I | 11 | 8 | 8 | 4 | 1 | 0.35 | 4.71E-03 |
| IPI00453473,IPI00407339 | Histone H4 | 10 | 7 | 10 | 2 | 1 | 0.25 | 3.22E-03 |
| IPI00002352,IPI00224549 | Myosin regulatory light chain 2| skeletal muscle isoform | 9 | 7 | 1 | 2 | 1 | 0.38 | 4.88E-03 |
| IPI00550363,IPI00647915  IPI00125778 | Transgelin-2 | 9 | 2 | 6 | 0 | 0 | 0.15 | 1.82E-02 |
| IPI00418471,IPI00227299 | Vimentin | 9 | 7 | 6 | 0 | 2 | 0.24 | 1.13E-03 |
| **HCD fragmentation method** | | | | | | | | |
| IPI00003918,IPI00795303  IPI00111412 | 60S ribosomal protein L4 | 4 | 6 | 2 | 0 | 0 | 0.20 | 4.76E-03 |
| IPI00217469 | Histone H1.1 | 7 | 10 | 7 | 2 | 1 | 0.28 | 3.22E-03 |
| IPI00217465,IPI00217466,IPI00217467  IPI00223714,IPI00331597 | Histone H1.2 | 11 | 17 | 10 | 1 | 1 | 0.15 | 2.51E-06 |
| IPI00217468 | Histone H1.5 | 4 | 5 | 0 | 0 | 0 | 0.25 | 7.94E-03 |
| IPI00419833 | Histone H2B type 1-K | 5 | 7 | 1 | 2 | 1 | 0.47 | 3.50E-02 |
| IPI00453473,IPI00407339 | Histone H4 | 12 | 11 | 6 | 2 | 2 | 0.28 | 3.63E-04 |

Non-underlined and underlined IPI accession numbers are originated from Human and Mouse databases, respectively. Only statistically significant proteins are shown in this table (Fisher’s exact test, p<0.05). CID: collision-induced dissociation, HCD: higher-energy collisional dissociation.

| **Table C** | | | | | | | | |
| --- | --- | --- | --- | --- | --- | --- | --- | --- |
| **Protein accession numbers** | **Protein name** | **Control 1** | **Control 2** | **Tumor 1** | **Tumor 2** | **Tumor 3** | **Fold Change** | **Fisher** |
| IPI00216308 | Voltage-dependent anion-selective channel protein 1 | 8 | 10 | 7 | 5 | 5 | 0.67 | 2.31E-01 |
| IPI00101968|IPI00396437|IPI00456925 | Isoform 3 of Drebrin-like protein | 0 | 0 | 1 | 2 | 1 | 2.33 | 3.33E-01 |
| IPI00413108|IPI00553164|IPI00927101 | 33 kDa protein | 3 | 3 | 5 | 4 | 4 | 1.33 | 4.05E-01 |
| IPI00013860 | 3-hydroxyisobutyrate dehydrogenase| mitochondrial | 1 | 1 | 0 | 0 | 0 | 0.50 | 5.00E-01 |
| IPI00640400|IPI00877658|IPI00921936 | Isoform 2 of Carnitine O-palmitoyltransferase 1| muscle isoform | 1 | 1 | 0 | 0 | 0 | 0.50 | 5.00E-01 |
| IPI00005966 | 13kDa differentiation-associated protein variant (Fragment) | 1 | 1 | 0 | 0 | 0 | 0.50 | 5.00E-01 |
| IPI00025239|IPI00946334 | NADH dehydrogenase [ubiquinone] iron-sulfur protein 2| mitochondrial | 3 | 3 | 0 | 1 | 1 | 0.42 | 1.14E-01 |
| IPI00177817|IPI00219078|IPI00747443|IPI00969023 | Isoform 2 of Sarcoplasmic/endoplasmic reticulum calcium ATPase 2 | 13 | 14 | 0 | 0 | 9 | 0.28 | 4.99E-08 |
| IPI00290077 | Keratin| type I cytoskeletal 15 | 8 | 8 | 0 | 0 | 7 | 0.37 | 7.77E-05 |
| IPI00006091|IPI00304639|IPI00472316|IPI01009672 | Isoform 4 of Dystrophin | 2 | 1 | 0 | 0 | 0 | 0.40 | 3.33E-01 |
| IPI00298994 | Talin-1 | 4 | 4 | 33 | 42 | 38 | 7.73 | 2.09E-14 |
| IPI00216070 | Isoform MLC1 of Myosin light chain 1/3| skeletal muscle isoform | 6 | 6 | 5 | 5 | 5 | 0.86 | 5.00E-01 |
| IPI00027626|IPI01013273 | T-complex protein 1 subunit zeta | 0 | 0 | 1 | 1 | 1 | 2.00 | 5.00E-01 |
| IPI00027834 | Heterogeneous nuclear ribonucleoprotein L | 1 | 2 | 4 | 5 | 3 | 2.00 | 1.21E-01 |
| IPI00009342 | Ras GTPase-activating-like protein IQGAP1 | 0 | 1 | 2 | 6 | 5 | 3.56 | 8.33E-02 |
| IPI00007858 | Myosin-13 | 31 | 37 | 28 | 20 | 24 | 0.71 | 4.37E-02 |
| IPI00033025|IPI00816201|IPI00941534|IPI01009775|IPI01015990 | Uncharacterized protein | 0 | 0 | 1 | 2 | 1 | 2.33 | 3.33E-01 |
| IPI00017855 | Aconitate hydratase| mitochondrial | 15 | 17 | 14 | 9 | 11 | 0.73 | 1.57E-01 |
| IPI00216171|IPI00791564 | Gamma-enolase | 5 | 5 | 4 | 0 | 0 | 0.39 | 6.29E-02 |
| IPI00026516 | Succinyl-CoA:3-ketoacid-coenzyme A transferase 1| mitochondrial | 2 | 2 | 0 | 0 | 0 | 0.33 | 1.67E-01 |
| IPI00007765|IPI00966238 | Stress-70 protein| mitochondrial | 11 | 13 | 5 | 8 | 7 | 0.59 | 6.41E-02 |
| IPI00220642 | 14-3-3 protein gamma | 5 | 5 | 7 | 8 | 6 | 1.33 | 2.77E-01 |
| IPI00296053|IPI00759715 | Isoform Mitochondrial of Fumarate hydratase| mitochondrial | 6 | 5 | 3 | 4 | 4 | 0.72 | 3.19E-01 |
| IPI00006663 | Aldehyde dehydrogenase| mitochondrial | 6 | 4 | 3 | 3 | 1 | 0.56 | 3.29E-01 |
| IPI00220213|IPI01011141 | Isoform 4 of Tenascin | 0 | 0 | 2 | 4 | 2 | 3.67 | 6.67E-02 |
| IPI00023006 | Actin| alpha cardiac muscle 1 | 24 | 23 | 19 | 21 | 20 | 0.86 | 2.95E-01 |
| IPI00744119 | Isoform 3 of Obscurin | 3 | 2 | 0 | 0 | 1 | 0.38 | 1.00E-01 |
| IPI00299000|IPI01022048 | Proliferation-associated protein 2G4 | 1 | 1 | 2 | 4 | 2 | 1.83 | 2.86E-01 |
| IPI00003815 | Rho GDP-dissociation inhibitor 1 | 0 | 0 | 2 | 2 | 1 | 2.67 | 1.67E-01 |
| IPI00444262|IPI00604620 | cDNA FLJ45706 fis| clone FEBRA2028457| highly similar to Nucleolin | 0 | 0 | 3 | 5 | 2 | 4.33 | 1.79E-02 |
| IPI00170914 | Isoform 1 of Adenylosuccinate synthetase isozyme 1 | 5 | 2 | 1 | 0 | 1 | 0.37 | 1.07E-01 |
| IPI00013991 | Isoform 1 of Tropomyosin beta chain | 21 | 24 | 19 | 19 | 19 | 0.85 | 2.96E-01 |
| IPI00465439|IPI00796333 | Fructose-bisphosphate aldolase A | 16 | 17 | 13 | 11 | 11 | 0.72 | 1.75E-01 |
| IPI00027230 | Endoplasmin | 5 | 5 | 8 | 10 | 8 | 1.61 | 1.33E-01 |
| IPI00719622 | 40S ribosomal protein S28 | 0 | 1 | 2 | 2 | 2 | 2.00 | 3.00E-01 |
| IPI00395769|IPI01015376 | Isoform Heart of ATP synthase subunit gamma| mitochondrial | 2 | 2 | 0 | 0 | 0 | 0.33 | 1.67E-01 |
| IPI00917728 | nebulin isoform 2 | 50 | 47 | 24 | 10 | 15 | 0.35 | 7.00E-09 |
| IPI00000494|IPI00640037 | 60S ribosomal protein L5 | 1 | 0 | 2 | 3 | 5 | 2.89 | 2.00E-01 |
| IPI00290857|IPI00973210 | Keratin| type II cytoskeletal 3 | 8 | 8 | 0 | 0 | 0 | 0.11 | 7.77E-05 |
| IPI00658109 | Isoform 1 of Creatine kinase U-type| mitochondrial | 3 | 4 | 0 | 2 | 0 | 0.37 | 1.19E-01 |
| IPI00292496 | Tubulin beta-8 chain | 7 | 7 | 0 | 6 | 6 | 0.63 | 2.21E-02 |
| IPI00217963 | Keratin| type I cytoskeletal 16 | 8 | 8 | 11 | 11 | 11 | 1.33 | 2.59E-01 |
| IPI00018146 | 14-3-3 protein theta | 2 | 4 | 7 | 7 | 7 | 2.00 | 7.99E-02 |
| IPI00013890 | Isoform 1 of 14-3-3 protein sigma | 4 | 6 | 8 | 8 | 7 | 1.44 | 2.07E-01 |
| IPI00302329 | Myosin-8 | 61 | 70 | 51 | 43 | 50 | 0.74 | 9.67E-03 |
| IPI00014898|IPI00186711|IPI00398002|IPI00398775|IPI00398776|IPI00398777|IPI00398778|IPI00398779|IPI00420096 | Isoform 1 of Plectin | 13 | 9 | 16 | 30 | 24 | 2.03 | 6.87E-03 |
| IPI00025880|IPI01015436 | Myosin-7 | 30 | 34 | 27 | 22 | 23 | 0.76 | 1.08E-01 |
| IPI00024320 | Putative RNA-binding protein 3 | 0 | 0 | 1 | 1 | 1 | 2.00 | 5.00E-01 |
| IPI00021428 | Actin| alpha skeletal muscle | 24 | 23 | 19 | 22 | 21 | 0.88 | 3.31E-01 |
| IPI00032140|IPI00910487 | Serpin H1 | 1 | 1 | 3 | 3 | 3 | 2.00 | 2.43E-01 |
| IPI00219684 | Fatty acid-binding protein| heart | 0 | 0 | 1 | 1 | 1 | 2.00 | 5.00E-01 |
| IPI00215914|IPI00215917 | ADP-ribosylation factor 1 | 3 | 4 | 0 | 0 | 0 | 0.22 | 2.86E-02 |
| IPI00008692 | Isoform 1 of Keratin| type I cuticular Ha6 | 4 | 5 | 0 | 0 | 0 | 0.18 | 7.94E-03 |
| IPI00012493|IPI00794659 | 40S ribosomal protein S20 | 0 | 0 | 2 | 3 | 2 | 3.33 | 1.00E-01 |
| IPI00018342|IPI00640817 | Adenylate kinase isoenzyme 1 | 4 | 3 | 2 | 2 | 1 | 0.59 | 3.92E-01 |
| IPI00010951 | Epiplakin | 0 | 0 | 1 | 5 | 2 | 3.67 | 1.67E-01 |
| IPI00029623 | Proteasome subunit alpha type-6 | 0 | 0 | 1 | 2 | 1 | 2.33 | 3.33E-01 |
| IPI00014424 | Elongation factor 1-alpha 2 | 12 | 11 | 7 | 8 | 9 | 0.72 | 1.65E-01 |
| IPI00015141 | Creatine kinase S-type| mitochondrial | 12 | 15 | 9 | 10 | 9 | 0.71 | 1.94E-01 |
| IPI00300376 | Protein-glutamine gamma-glutamyltransferase E | 2 | 2 | 1 | 0 | 0 | 0.44 | 3.00E-01 |
| IPI00163187 | Fascin | 0 | 0 | 3 | 7 | 2 | 5.00 | 8.33E-03 |
| IPI00025796 | NADH dehydrogenase [ubiquinone] iron-sulfur protein 3| mitochondrial | 5 | 4 | 2 | 1 | 2 | 0.48 | 1.21E-01 |
| IPI00013683 | Tubulin beta-3 chain | 0 | 0 | 12 | 13 | 12 | 13.33 | 1.92E-07 |
| IPI00294959|IPI00909817 | Isoform 2 of LIM domain-binding protein 3 | 8 | 9 | 5 | 3 | 2 | 0.46 | 7.49E-02 |
| IPI00017297|IPI00789551 | Matrin-3 | 0 | 1 | 2 | 2 | 3 | 2.22 | 3.00E-01 |
| IPI00215743|IPI00220967|IPI00744135|IPI00856098|IPI01011993 | Isoform 3 of Ribosome-binding protein 1 | 0 | 0 | 2 | 4 | 2 | 3.67 | 6.67E-02 |
| IPI00291467 | ADP/ATP translocase 3 | 8 | 6 | 0 | 0 | 4 | 0.29 | 3.33E-04 |
| IPI00030702 | Isoform 1 of Isocitrate dehydrogenase [NAD] subunit alpha| mitochondrial | 10 | 10 | 9 | 4 | 7 | 0.70 | 1.53E-01 |
| IPI00020210|IPI00793867|IPI00871702|IPI00890768|IPI00890779|IPI00908443|IPI00908579|IPI00908658|IPI00908764|IPI00908826|IPI00908936|IPI00910445|IPI00910624|IPI00911101|IPI00915417 | Isoform 14 of Dysferlin | 1 | 1 | 0 | 0 | 0 | 0.50 | 5.00E-01 |
| IPI00396378|IPI00414696 | Isoform B1 of Heterogeneous nuclear ribonucleoproteins A2/B1 | 4 | 5 | 8 | 14 | 6 | 1.88 | 3.24E-02 |
| IPI00414676 | Heat shock protein HSP 90-beta | 18 | 15 | 19 | 21 | 19 | 1.18 | 2.76E-01 |
| IPI00021447|IPI00025476|IPI00300786|IPI01010056 | Alpha-amylase 2B | 3 | 5 | 0 | 0 | 0 | 0.20 | 1.79E-02 |
| IPI00022498 | Metallothionein-2 | 0 | 0 | 2 | 2 | 2 | 3.00 | 1.67E-01 |
| IPI00479145 | Keratin| type I cytoskeletal 19 | 8 | 7 | 5 | 6 | 5 | 0.75 | 3.48E-01 |
| IPI00434580|IPI00479390|IPI00647020 | Isoform 1 of Myomesin-1 | 5 | 6 | 2 | 2 | 2 | 0.46 | 1.00E-01 |
| IPI00021338 | Dihydrolipoyllysine-residue acetyltransferase component of pyruvate dehydrogenase complex| mitochondrial | 4 | 4 | 2 | 0 | 2 | 0.47 | 7.14E-02 |
| IPI00018219|IPI00940091|IPI00965868 | Transforming growth factor-beta-induced protein ig-h3 | 0 | 0 | 2 | 5 | 2 | 4.00 | 4.76E-02 |
| IPI00843975 | Ezrin | 0 | 0 | 2 | 8 | 7 | 6.67 | 2.22E-02 |
| IPI00298933|IPI00909474 | Calsequestrin-2 | 1 | 2 | 0 | 0 | 0 | 0.40 | 3.33E-01 |
| IPI00216694|IPI00947227|IPI01011191 | Plastin-3 | 0 | 0 | 2 | 10 | 4 | 6.33 | 1.52E-02 |
| IPI00025366|IPI00383539 | Citrate synthase| mitochondrial | 4 | 3 | 2 | 2 | 2 | 0.67 | 3.92E-01 |
| IPI00418471 | Vimentin | 14 | 12 | 18 | 15 | 17 | 1.26 | 2.61E-01 |
| IPI00465084 | Desmin | 21 | 18 | 17 | 11 | 15 | 0.75 | 1.23E-01 |
| IPI00216318|IPI00759832 | Isoform Long of 14-3-3 protein beta/alpha | 5 | 5 | 7 | 7 | 7 | 1.33 | 3.42E-01 |
| IPI00304596 | Non-POU domain-containing octamer-binding protein | 0 | 0 | 2 | 1 | 1 | 2.33 | 3.33E-01 |
| IPI00017596|IPI00910350 | Microtubule-associated protein RP/EB family member 1 | 0 | 0 | 1 | 1 | 1 | 2.00 | 5.00E-01 |
| IPI00027107 | elongation factor Tu| mitochondrial precursor | 6 | 5 | 4 | 3 | 4 | 0.72 | 3.19E-01 |
| IPI00022314|IPI00607577|IPI00847322 | Superoxide dismutase [Mn]| mitochondrial | 2 | 2 | 0 | 1 | 1 | 0.56 | 3.00E-01 |
| IPI00018206 | Aspartate aminotransferase| mitochondrial | 9 | 8 | 5 | 3 | 5 | 0.56 | 7.49E-02 |
| IPI00003482|IPI00980553 | 2|4-dienoyl-CoA reductase| mitochondrial | 1 | 1 | 0 | 0 | 0 | 0.50 | 5.00E-01 |
| IPI00215948|IPI00980391|IPI00981271 | Isoform 1 of Catenin alpha-1 | 0 | 0 | 2 | 6 | 3 | 4.67 | 3.57E-02 |
| IPI00012007 | Adenosylhomocysteinase | 1 | 1 | 3 | 6 | 2 | 2.33 | 8.79E-02 |
| IPI00396485 | Elongation factor 1-alpha 1 | 13 | 12 | 8 | 11 | 10 | 0.79 | 2.64E-01 |
| IPI00014177|IPI00871851 | Isoform 1 of Septin-2 | 0 | 0 | 3 | 4 | 2 | 4.00 | 2.86E-02 |
| IPI00157144|IPI00303868 | glycogen [starch] synthase| muscle isoform 2 | 5 | 3 | 0 | 0 | 1 | 0.27 | 1.79E-02 |
| IPI00299571|IPI00644989|IPI01010189|IPI01011902|IPI01013559 | Isoform 2 of Protein disulfide-isomerase A6 | 1 | 1 | 3 | 2 | 2 | 1.67 | 3.71E-01 |
| IPI00022793|IPI01018954 | Trifunctional enzyme subunit beta| mitochondrial | 4 | 4 | 1 | 0 | 0 | 0.27 | 3.97E-02 |
| IPI00479834|IPI00873889|IPI00940615 | Uncharacterized protein | 0 | 0 | 1 | 1 | 2 | 2.33 | 5.00E-01 |
| IPI00103146|IPI00942043|IPI00964290|IPI01013659 | Isoform 3 of PDZ and LIM domain protein 5 | 1 | 2 | 0 | 0 | 0 | 0.40 | 3.33E-01 |
| IPI00219065|IPI00219066|IPI00514126 | Isoform 5 of Glycogen debranching enzyme | 4 | 3 | 1 | 0 | 0 | 0.30 | 7.14E-02 |
| IPI00019884 | Alpha-actinin-2 | 40 | 39 | 27 | 26 | 30 | 0.71 | 1.81E-02 |
| IPI00301028 | Isoform 1 of Tripartite motif-containing protein 72 | 3 | 3 | 2 | 2 | 2 | 0.75 | 5.00E-01 |
| IPI00011229 | Cathepsin D | 0 | 0 | 1 | 1 | 1 | 2.00 | 5.00E-01 |
| IPI00219207|IPI00873380 | Isoform 3 of Reticulon-4 | 1 | 1 | 0 | 0 | 0 | 0.50 | 5.00E-01 |
| IPI00011250 | Ubiquitin carboxyl-terminal hydrolase isozyme L3 | 0 | 0 | 2 | 5 | 3 | 4.33 | 4.76E-02 |
| IPI00396435 | Putative pre-mRNA-splicing factor ATP-dependent RNA helicase DHX15 | 0 | 0 | 2 | 2 | 1 | 2.67 | 1.67E-01 |
| IPI00003865 | Isoform 1 of Heat shock cognate 71 kDa protein | 18 | 16 | 24 | 23 | 23 | 1.35 | 1.10E-01 |
| IPI00479185 | tropomyosin alpha-3 chain isoform 4 | 9 | 8 | 13 | 13 | 10 | 1.37 | 1.42E-01 |
| IPI00072377|IPI00301311|IPI00844040|IPI00896523 | Isoform 1 of Protein SET | 0 | 0 | 2 | 4 | 3 | 4.00 | 6.67E-02 |
| IPI00022832|IPI00640179 | Brain protein 44 | 1 | 1 | 0 | 0 | 0 | 0.50 | 5.00E-01 |
| IPI00004902|IPI00556451 | Isoform 1 of Electron transfer flavoprotein subunit beta | 4 | 5 | 3 | 2 | 2 | 0.61 | 2.96E-01 |
| IPI00604664|IPI00940744 | NADH-ubiquinone oxidoreductase 75 kDa subunit| mitochondrial isoform 5 | 8 | 9 | 6 | 5 | 4 | 0.63 | 2.25E-01 |
| IPI00641829|IPI00790636|IPI00848161 | Isoform 2 of Spliceosome RNA helicase DDX39B | 0 | 0 | 2 | 4 | 3 | 4.00 | 6.67E-02 |
| IPI00219365 | Moesin | 1 | 1 | 6 | 11 | 7 | 4.50 | 1.69E-03 |
| IPI00218130|IPI01009320 | Glycogen phosphorylase| muscle form | 18 | 17 | 13 | 6 | 11 | 0.59 | 1.88E-02 |
| IPI00289334|IPI00382697|IPI00477536|IPI00900293|IPI00943563|IPI00953109 | Isoform 1 of Filamin-B | 0 | 0 | 4 | 7 | 6 | 6.67 | 3.03E-03 |
| IPI00025252|IPI01012004 | Protein disulfide-isomerase A3 | 3 | 2 | 7 | 4 | 6 | 1.90 | 1.82E-01 |
| IPI00643920|IPI00942979 | cDNA FLJ54957| highly similar to Transketolase | 1 | 0 | 2 | 4 | 4 | 2.89 | 1.43E-01 |
| IPI00002459 | Uncharacterized protein | 6 | 5 | 3 | 2 | 3 | 0.56 | 1.57E-01 |
| IPI00334799 | Isoform 3 of Ryanodine receptor 1 | 2 | 2 | 0 | 0 | 0 | 0.33 | 1.67E-01 |
| IPI00022774 | Transitional endoplasmic reticulum ATPase | 14 | 11 | 24 | 31 | 28 | 2.12 | 8.76E-04 |
| IPI00216319 | 14-3-3 protein eta | 0 | 0 | 6 | 5 | 2 | 5.33 | 2.16E-03 |
| IPI00217975 | Lamin-B1 | 0 | 0 | 3 | 3 | 1 | 3.33 | 5.00E-02 |
| IPI00296441 | Adenosine deaminase | 4 | 5 | 2 | 3 | 2 | 0.61 | 2.96E-01 |
| IPI00022418|IPI00339223|IPI00339225|IPI00339227|IPI00339228|IPI00414283|IPI00479723|IPI00845263|IPI00855777|IPI00855785|IPI00867588 | Isoform 1 of Fibronectin | 0 | 0 | 1 | 4 | 2 | 3.33 | 2.00E-01 |
| IPI00007752 | Tubulin beta-2C chain | 17 | 17 | 16 | 14 | 13 | 0.85 | 3.98E-01 |
| IPI00297779 | T-complex protein 1 subunit beta | 0 | 1 | 2 | 2 | 3 | 2.22 | 3.00E-01 |
| IPI00100980 | EH domain-containing protein 2 | 4 | 3 | 2 | 1 | 2 | 0.59 | 2.62E-01 |
| IPI00215965|IPI00465365|IPI00797148|IPI01021093|IPI01021324|IPI01022801 | Isoform A1-B of Heterogeneous nuclear ribonucleoprotein A1 | 1 | 1 | 4 | 8 | 2 | 2.83 | 2.30E-02 |
| IPI00001753 | Myosin-4 | 76 | 85 | 71 | 56 | 60 | 0.78 | 2.72E-02 |
| IPI00025879 | Myosin-1 | 92 | 102 | 83 | 71 | 78 | 0.80 | 2.02E-02 |
| IPI00021812 | Neuroblast differentiation-associated protein AHNAK | 12 | 12 | 14 | 17 | 18 | 1.33 | 2.57E-01 |
| IPI00024990 | Methylmalonate-semialdehyde dehydrogenase [acylating]| mitochondrial | 2 | 3 | 0 | 0 | 0 | 0.29 | 1.00E-01 |
| IPI00029111|IPI00872788 | dihydropyrimidinase-related protein 3 isoform 1 | 1 | 2 | 0 | 0 | 0 | 0.40 | 3.33E-01 |
| IPI00007052 | Mitochondrial fission 1 protein | 2 | 2 | 0 | 0 | 0 | 0.33 | 1.67E-01 |
| IPI00215884 | Isoform ASF-1 of Serine/arginine-rich splicing factor 1 | 0 | 0 | 1 | 3 | 2 | 3.00 | 2.50E-01 |
| IPI00219729|IPI00945233 | Mitochondrial 2-oxoglutarate/malate carrier protein | 4 | 3 | 0 | 0 | 0 | 0.22 | 2.86E-02 |
| IPI00218474 | Isoform 1 of Beta-enolase | 18 | 17 | 10 | 7 | 13 | 0.59 | 1.18E-02 |
| IPI00020987|IPI00980755 | Prolargin | 4 | 4 | 3 | 0 | 2 | 0.53 | 1.06E-01 |
| IPI00298281 | Laminin subunit gamma-1 | 4 | 3 | 2 | 1 | 2 | 0.59 | 2.62E-01 |
| IPI00304925|IPI00845339 | Heat shock 70 kDa protein 1A/1B | 4 | 3 | 5 | 6 | 5 | 1.41 | 3.19E-01 |
| IPI00290078|IPI00797452|IPI01022175|IPI01022327 | 64 kDa protein | 7 | 6 | 2 | 5 | 4 | 0.62 | 1.57E-01 |
| IPI00020008 | NEDD8 | 1 | 1 | 0 | 0 | 0 | 0.50 | 5.00E-01 |
| IPI00940872 | Titin| isoform CRA_a | 150 | 166 | 69 | 33 | 57 | 0.34 | 5.66E-28 |
| IPI00465070 | Histone H3.1 | 0 | 0 | 3 | 2 | 1 | 3.00 | 1.00E-01 |
| IPI00215777|IPI01021698|IPI01022950 | Isoform B of Phosphate carrier protein| mitochondrial | 2 | 2 | 0 | 1 | 1 | 0.56 | 3.00E-01 |
| IPI00007188 | ADP/ATP translocase 2 | 9 | 5 | 2 | 2 | 2 | 0.38 | 3.87E-02 |
| IPI00010779 | Isoform 1 of Tropomyosin alpha-4 chain | 0 | 0 | 10 | 8 | 7 | 9.33 | 2.29E-05 |
| IPI00302592|IPI00333541 | Isoform 2 of Filamin-A | 6 | 5 | 13 | 23 | 22 | 3.13 | 6.39E-04 |
| IPI00025491 | Eukaryotic initiation factor 4A-I | 5 | 2 | 7 | 10 | 9 | 2.15 | 4.47E-02 |
| IPI00658052 | Isoform 1 of Sarcalumenin | 9 | 7 | 5 | 3 | 6 | 0.63 | 1.07E-01 |
| IPI00011107|IPI01015385 | Isocitrate dehydrogenase [NADP]| mitochondrial | 7 | 10 | 5 | 5 | 5 | 0.63 | 1.82E-01 |
| IPI00011770 | NADH dehydrogenase [ubiquinone] 1 alpha subcomplex subunit 4 | 1 | 1 | 0 | 0 | 0 | 0.50 | 5.00E-01 |
| IPI00453473 | Histone H4 | 5 | 5 | 9 | 8 | 8 | 1.56 | 1.70E-01 |
| IPI00219525|IPI01012504 | 6-phosphogluconate dehydrogenase| decarboxylating | 1 | 0 | 2 | 4 | 2 | 2.44 | 1.43E-01 |
| IPI00075248|IPI00916600 | Calmodulin | 0 | 0 | 3 | 3 | 4 | 4.33 | 5.00E-02 |
| IPI00021369|IPI00981730 | Alpha-crystallin B chain | 3 | 4 | 1 | 1 | 2 | 0.52 | 1.67E-01 |
| IPI00028091 | Actin-related protein 3 | 1 | 2 | 4 | 7 | 7 | 2.80 | 6.29E-02 |
| IPI00013895 | Protein S100-A11 | 0 | 0 | 2 | 2 | 2 | 3.00 | 1.67E-01 |
| IPI00220150|IPI01011396 | Isocitrate dehydrogenase [NAD] subunit gamma| mitochondrial | 8 | 8 | 5 | 1 | 3 | 0.44 | 2.47E-02 |
| IPI00465179|IPI00743142 | 6-phosphofructokinase| muscle type isoform 1 | 9 | 10 | 5 | 3 | 5 | 0.51 | 3.77E-02 |
| IPI00009866|IPI00171196 | Isoform 1 of Keratin| type I cytoskeletal 13 | 11 | 12 | 9 | 8 | 10 | 0.80 | 2.64E-01 |
| IPI00014581 | Isoform 1 of Tropomyosin alpha-1 chain | 20 | 19 | 17 | 18 | 16 | 0.88 | 4.08E-01 |
| IPI00291922 | Proteasome subunit alpha type-5 | 0 | 0 | 3 | 2 | 3 | 3.67 | 1.00E-01 |
| IPI00419237 | Isoform 1 of Cytosol aminopeptidase | 2 | 3 | 0 | 0 | 1 | 0.38 | 1.00E-01 |
| IPI00465361 | 60S ribosomal protein L13 | 0 | 0 | 2 | 2 | 3 | 3.33 | 1.67E-01 |
| IPI00007856 | Myosin-2 | 73 | 83 | 61 | 52 | 57 | 0.73 | 6.11E-03 |
| IPI00030781 | Isoform Alpha of Signal transducer and activator of transcription 1-alpha/beta | 0 | 0 | 3 | 2 | 1 | 3.00 | 1.00E-01 |
| IPI00220740|IPI00549248 | Isoform 2 of Nucleophosmin | 0 | 0 | 1 | 4 | 1 | 3.00 | 2.00E-01 |
| IPI00031522 | Trifunctional enzyme subunit alpha| mitochondrial | 2 | 3 | 1 | 1 | 1 | 0.57 | 3.71E-01 |
| IPI00005587 | Myomesin-2 | 3 | 1 | 0 | 0 | 0 | 0.33 | 2.50E-01 |
| IPI00028520|IPI00221298|IPI00978187 | Isoform 1 of NADH dehydrogenase [ubiquinone] flavoprotein 1| mitochondrial | 11 | 9 | 4 | 1 | 4 | 0.36 | 2.71E-03 |
| IPI00179964|IPI00183626|IPI00334175 | Isoform 1 of Polypyrimidine tract-binding protein 1 | 0 | 2 | 3 | 3 | 5 | 2.33 | 1.79E-01 |
| IPI00031461|IPI00940148 | cDNA FLJ60299| highly similar to Rab GDP dissociation inhibitor beta | 2 | 1 | 6 | 6 | 5 | 2.67 | 3.17E-02 |
| IPI00010810 | Electron transfer flavoprotein subunit alpha| mitochondrial | 4 | 5 | 2 | 3 | 2 | 0.61 | 2.96E-01 |
| IPI00413641 | Aldose reductase | 1 | 2 | 0 | 0 | 0 | 0.40 | 3.33E-01 |
| IPI00008994 | Isoform 1 of Protein NDRG2 | 2 | 3 | 0 | 0 | 0 | 0.29 | 1.00E-01 |
| IPI00021907 | Isoform 1 of Myelin basic protein | 0 | 0 | 2 | 2 | 1 | 2.67 | 1.67E-01 |
| IPI00257508|IPI00883655|IPI00984387 | Dihydropyrimidinase-related protein 2 | 4 | 5 | 2 | 3 | 3 | 0.67 | 2.96E-01 |
| IPI00021263 | 14-3-3 protein zeta/delta | 7 | 10 | 12 | 13 | 13 | 1.44 | 1.69E-01 |
| IPI00014399 | Four and a half LIM domains protein 3 | 2 | 2 | 1 | 0 | 0 | 0.44 | 3.00E-01 |
| IPI00019502 | Isoform 1 of Myosin-9 | 14 | 14 | 20 | 32 | 30 | 1.89 | 1.03E-02 |
| IPI00742682 | Nucleoprotein TPR | 0 | 0 | 1 | 2 | 1 | 2.33 | 3.33E-01 |
| IPI00436518 | Benzodiazepine receptor ligand | 3 | 3 | 2 | 2 | 2 | 0.75 | 5.00E-01 |
| IPI00105598 | Proteasome 26S non-ATPase subunit 11 variant (Fragment) | 0 | 0 | 1 | 3 | 1 | 2.67 | 2.50E-01 |
| IPI00008524 | Isoform 1 of Polyadenylate-binding protein 1 | 2 | 0 | 5 | 3 | 4 | 2.50 | 8.33E-02 |
| IPI00383695 | Mitochondrial trifunctional protein beta subunit (Fragment) | 5 | 4 | 0 | 1 | 0 | 0.24 | 2.38E-02 |
| IPI00024804 | Isoform SERCA1B of Sarcoplasmic/endoplasmic reticulum calcium ATPase 1 | 26 | 25 | 7 | 11 | 11 | 0.40 | 6.08E-05 |
| IPI00032164 | Cysteine and glycine-rich protein 3 | 2 | 2 | 3 | 3 | 4 | 1.44 | 5.00E-01 |
| IPI00913991 | pyruvate dehydrogenase protein X component| mitochondrial isoform 2 | 2 | 2 | 0 | 1 | 0 | 0.44 | 3.00E-01 |
| IPI00018398 | 26S protease regulatory subunit 6A | 3 | 2 | 5 | 6 | 4 | 1.71 | 1.57E-01 |
| IPI00386271 | Calcium-binding mitochondrial carrier protein Aralar1 | 12 | 9 | 1 | 1 | 2 | 0.20 | 1.15E-04 |
| IPI00027701 | Short-chain specific acyl-CoA dehydrogenase| mitochondrial | 4 | 4 | 2 | 2 | 2 | 0.60 | 2.84E-01 |
| IPI00008438 | 40S ribosomal protein S10 | 1 | 0 | 2 | 1 | 2 | 1.78 | 1.00E+00 |
| IPI00026230 | Heterogeneous nuclear ribonucleoprotein H2 | 0 | 2 | 3 | 3 | 2 | 1.83 | 1.79E-01 |
| IPI00290566 | T-complex protein 1 subunit alpha | 1 | 2 | 4 | 4 | 2 | 1.73 | 1.75E-01 |
| IPI00329331|IPI00395676|IPI00873223 | Isoform 1 of UTP--glucose-1-phosphate uridylyltransferase | 5 | 4 | 4 | 2 | 2 | 0.67 | 6.57E-01 |
| IPI00003925|IPI00549885 | Isoform 1 of Pyruvate dehydrogenase E1 component subunit beta| mitochondrial | 4 | 5 | 4 | 3 | 3 | 0.79 | 7.04E-01 |
| IPI00003021|IPI00640401 | Sodium/potassium-transporting ATPase subunit alpha-2 | 6 | 4 | 0 | 0 | 4 | 0.39 | 4.76E-03 |
| IPI00294911 | Succinate dehydrogenase [ubiquinone] iron-sulfur subunit| mitochondrial | 2 | 3 | 1 | 2 | 1 | 0.67 | 8.00E-01 |
| IPI00030986|IPI00220297 | Isoform Long of Kelch repeat and BTB domain-containing protein 10 | 2 | 1 | 1 | 0 | 0 | 0.53 | 1.00E+00 |
| IPI01015522 | cDNA FLJ55253| highly similar to Actin| cytoplasmic 1 | 17 | 16 | 0 | 0 | 16 | 0.36 | 8.57E-10 |
| IPI00554723|IPI00853161 | 60S ribosomal protein L10 | 1 | 2 | 1 | 0 | 0 | 0.53 | 1.00E+00 |
| IPI00017617|IPI00947363|IPI00984405 | Probable ATP-dependent RNA helicase DDX5 | 0 | 1 | 1 | 3 | 3 | 2.22 | 5.71E-01 |
| IPI00219217 | L-lactate dehydrogenase B chain | 8 | 4 | 3 | 3 | 4 | 0.62 | 2.20E-01 |
| IPI00014398|IPI00643324|IPI00647207|IPI00902610|IPI00922730|IPI00930706|IPI01018104 | four and a half LIM domains protein 1 isoform 5 | 7 | 6 | 6 | 4 | 4 | 0.76 | 6.08E-01 |
| IPI00217966|IPI00947127 | Isoform 1 of L-lactate dehydrogenase A chain | 5 | 4 | 2 | 4 | 2 | 0.67 | 6.57E-01 |
| IPI00029264 | Cytochrome c1| heme protein| mitochondrial | 2 | 1 | 0 | 0 | 1 | 0.53 | 3.33E-01 |
| IPI00221093 | 40S ribosomal protein S17 | 2 | 1 | 3 | 3 | 2 | 1.47 | 5.00E-01 |
| IPI00220683|IPI00220685|IPI00903278|IPI01014602 | Isoform 2 of Heterogeneous nuclear ribonucleoprotein D0 | 0 | 2 | 3 | 3 | 2 | 1.83 | 1.79E-01 |
| IPI00186966|IPI00220586|IPI00220587|IPI00220996|IPI00220997|IPI00220998|IPI00220999|IPI00221000|IPI00221001|IPI00329733|IPI00395680 | Isoform IIA of Myc box-dependent-interacting protein 1 | 5 | 4 | 4 | 3 | 3 | 0.79 | 6.22E-01 |
| IPI00029133|IPI00456747 | ATP synthase subunit b| mitochondrial | 2 | 1 | 0 | 0 | 1 | 0.53 | 3.33E-01 |
| IPI00024913|IPI00218482|IPI00793677 | Isoform Long of ES1 protein homolog| mitochondrial | 1 | 0 | 2 | 1 | 2 | 1.78 | 1.00E+00 |
| IPI00306301 | pyruvate dehydrogenase E1 component subunit alpha| somatic form| mitochondrial isoform 2 precursor | 9 | 7 | 6 | 7 | 6 | 0.81 | 5.21E-01 |
| IPI00028031|IPI00178744|IPI00937735|IPI01013108 | cDNA FLJ56425| highly similar to Very-long-chain specific acyl-CoAdehydrogenase| mitochondrial | 3 | 2 | 2 | 0 | 0 | 0.48 | 1.00E+00 |
| IPI00470528|IPI00927715 | 60S ribosomal protein L15 | 1 | 3 | 0 | 1 | 0 | 0.44 | 1.00E+00 |
| IPI00018140|IPI00402182|IPI00402183|IPI00402184|IPI00930205|IPI00983581 | Isoform 1 of Heterogeneous nuclear ribonucleoprotein Q | 0 | 2 | 2 | 6 | 5 | 2.67 | 2.69E-01 |
| IPI00022200|IPI00072917|IPI00072918|IPI00220701|IPI00946286 | Isoform 1 of Collagen alpha-3(VI) chain | 0 | 1 | 4 | 1 | 3 | 2.44 | 5.00E-01 |
| IPI00025512 | Heat shock protein beta-1 | 0 | 1 | 1 | 3 | 2 | 2.00 | 5.00E-01 |
| IPI01014861 | Heterogeneous nuclear ribonucleoprotein M isoform a variant (Fragment) | 1 | 0 | 1 | 3 | 2 | 2.00 | 1.00E+00 |
| IPI00033494|IPI00220573 | Myosin regulatory light chain 12B | 0 | 1 | 2 | 3 | 1 | 2.00 | 2.00E-01 |
| IPI00553185 | T-complex protein 1 subunit gamma | 1 | 0 | 1 | 3 | 5 | 2.67 | 1.00E+00 |
| IPI00221092 | 40S ribosomal protein S16 | 1 | 0 | 3 | 2 | 1 | 2.00 | 4.86E-01 |
| IPI00025086 | Cytochrome c oxidase subunit 5A| mitochondrial | 4 | 3 | 3 | 2 | 1 | 0.67 | 6.67E-01 |
| IPI00001539 | 3-ketoacyl-CoA thiolase| mitochondrial | 4 | 3 | 2 | 0 | 3 | 0.59 | 1.19E-01 |
| IPI00008529 | 60S acidic ribosomal protein P2 | 2 | 0 | 2 | 4 | 3 | 2.00 | 1.00E+00 |
| IPI00013415 | 40S ribosomal protein S7 | 1 | 0 | 1 | 3 | 2 | 2.00 | 1.00E+00 |
| IPI00010740 | Isoform Long of Splicing factor| proline- and glutamine-rich | 1 | 0 | 4 | 3 | 1 | 2.44 | 2.06E-01 |
| IPI00024284 | Basement membrane-specific heparan sulfate proteoglycan core protein | 9 | 4 | 3 | 4 | 1 | 0.49 | 6.70E-01 |
| IPI00003362 | 78 kDa glucose-regulated protein | 12 | 13 | 13 | 19 | 16 | 1.26 | 4.14E-01 |
| IPI00646304 | Peptidyl-prolyl cis-trans isomerase B | 2 | 0 | 2 | 5 | 4 | 2.33 | 1.00E+00 |
| IPI00382470|IPI00784295 | Isoform 2 of Heat shock protein HSP 90-alpha | 8 | 7 | 8 | 13 | 11 | 1.37 | 5.25E-01 |
| IPI00002352 | Myosin regulatory light chain 2| skeletal muscle isoform | 12 | 14 | 12 | 10 | 11 | 0.86 | 5.55E-01 |
| IPI00023860|IPI00797545|IPI01013371|IPI01020731|IPI01021323|IPI01021493|IPI01021659|IPI01021819|IPI01022332|IPI01022822|IPI01022994 | Nucleosome assembly protein 1-like 1 | 0 | 0 | 0 | 1 | 1 | 1.67 | 1.00E+00 |
| IPI00910701 | cDNA FLJ61339| highly similar to Alanyl-tRNA synthetase | 0 | 0 | 0 | 2 | 2 | 2.33 | 1.00E+00 |
| IPI00219029 | Aspartate aminotransferase| cytoplasmic | 4 | 4 | 4 | 3 | 3 | 0.87 | 7.04E-01 |
| IPI00025039 | rRNA 2'-O-methyltransferase fibrillarin | 0 | 0 | 0 | 2 | 2 | 2.33 | 1.00E+00 |
| IPI00102069|IPI00979853 | Eukaryotic translation initiation factor 3 subunit M | 0 | 0 | 0 | 1 | 1 | 1.67 | 1.00E+00 |
| IPI00220916|IPI00375714|IPI00641046|IPI00902998|IPI00944996 | Isoform 2 of Heat shock protein beta-7 | 0 | 0 | 1 | 1 | 0 | 1.67 | 1.00E+00 |
| IPI00010414 | PDZ and LIM domain protein 1 | 0 | 0 | 1 | 1 | 0 | 1.67 | 1.00E+00 |
| IPI00009771 | Lamin-B2 | 0 | 0 | 0 | 1 | 1 | 1.67 | 1.00E+00 |
| IPI00020042|IPI00216770|IPI00738042 | Isoform 1 of 26S protease regulatory subunit 6B | 0 | 0 | 0 | 2 | 2 | 2.33 | 1.00E+00 |
| IPI00024145|IPI00216026|IPI00455531|IPI00902560|IPI00917420 | Isoform 2 of Voltage-dependent anion-selective channel protein 2 | 3 | 3 | 1 | 1 | 3 | 0.67 | 2.43E-01 |
| IPI00100160|IPI00604431 | Isoform 1 of Cullin-associated NEDD8-dissociated protein 1 | 0 | 0 | 0 | 1 | 1 | 1.67 | 1.00E+00 |
| IPI00921080 | Isoform 7 of Integrin alpha-6 | 0 | 0 | 0 | 2 | 2 | 2.33 | 1.00E+00 |
| IPI00000861|IPI00386803|IPI00883946 | Isoform 1 of LIM and SH3 domain protein 1 | 0 | 0 | 0 | 2 | 2 | 2.33 | 1.00E+00 |
| IPI00293464|IPI00909008|IPI00976686 | DNA damage-binding protein 1 | 0 | 0 | 1 | 1 | 0 | 1.67 | 1.00E+00 |
| IPI00028055 | Transmembrane emp24 domain-containing protein 10 | 0 | 0 | 0 | 2 | 2 | 2.33 | 1.00E+00 |
| IPI00291175|IPI00307162 | Isoform 1 of Vinculin | 7 | 7 | 7 | 6 | 6 | 0.92 | 6.52E-01 |
| IPI00009841|IPI00065554|IPI00293254|IPI00872855|IPI00879242|IPI00879259|IPI00953325 | RNA-binding protein EWS isoform 1 | 2 | 2 | 0 | 2 | 0 | 0.56 | 1.00E+00 |
| IPI00015148|IPI00019345|IPI00877120|IPI00908754|IPI01014435|IPI01015962 | Ras-related protein Rap-1b | 0 | 0 | 0 | 2 | 2 | 2.33 | 1.00E+00 |
| IPI00005160|IPI00737530 | Actin-related protein 2/3 complex subunit 1B | 0 | 0 | 1 | 1 | 0 | 1.67 | 1.00E+00 |
| IPI00021891|IPI00219713|IPI00877703|IPI00877792 | Isoform Gamma-B of Fibrinogen gamma chain | 0 | 0 | 3 | 3 | 0 | 3.00 | 1.00E-01 |
| IPI00005969 | F-actin-capping protein subunit alpha-1 | 0 | 0 | 1 | 1 | 0 | 1.67 | 1.00E+00 |
| IPI00844578 | ATP-dependent RNA helicase A | 0 | 0 | 1 | 1 | 0 | 1.67 | 1.00E+00 |
| IPI00479786|IPI00855957 | Isoform 1 of Far upstream element-binding protein 2 | 0 | 0 | 0 | 3 | 3 | 3.00 | 1.00E+00 |
| IPI00019599|IPI00019600|IPI00030962|IPI00447356|IPI00472498|IPI00514724|IPI00965965|IPI01012867|IPI01015529|IPI01021809 | 42 kDa protein | 0 | 0 | 0 | 2 | 2 | 2.33 | 1.00E+00 |
| IPI00013847 | Cytochrome b-c1 complex subunit 1| mitochondrial | 4 | 4 | 3 | 3 | 4 | 0.87 | 5.00E-01 |
| IPI00030179 | 60S ribosomal protein L7 | 0 | 0 | 0 | 1 | 1 | 1.67 | 1.00E+00 |
| IPI00299608|IPI00456695 | Isoform 1 of 26S proteasome non-ATPase regulatory subunit 1 | 0 | 0 | 0 | 1 | 1 | 1.67 | 1.00E+00 |
| IPI00176469 | Isoform 1 of Chaperone activity of bc1 complex-like| mitochondrial | 2 | 2 | 2 | 0 | 0 | 0.56 | 1.00E+00 |
| IPI00027341|IPI00908511 | Macrophage-capping protein | 0 | 0 | 0 | 2 | 2 | 2.33 | 1.00E+00 |
| IPI00296183|IPI00654709 | Aldehyde dehydrogenase| dimeric NADP-preferring | 1 | 1 | 1 | 0 | 0 | 0.67 | 1.00E+00 |
| IPI00001960|IPI01010396 | Chloride intracellular channel protein 4 | 0 | 0 | 0 | 2 | 2 | 2.33 | 1.00E+00 |
| IPI00549343 | Vesicle-associated membrane protein 3 | 0 | 0 | 0 | 2 | 2 | 2.33 | 1.00E+00 |
| IPI00010845|IPI00982019|IPI00984633 | NADH dehydrogenase [ubiquinone] iron-sulfur protein 8| mitochondrial | 2 | 2 | 2 | 0 | 0 | 0.56 | 1.00E+00 |
| IPI00005161 | Actin-related protein 2/3 complex subunit 2 | 0 | 0 | 1 | 1 | 0 | 1.67 | 1.00E+00 |
| IPI00305692 | Thioredoxin-like protein 1 | 0 | 0 | 1 | 1 | 0 | 1.67 | 1.00E+00 |
| IPI00008274|IPI01009563 | Isoform 1 of Adenylyl cyclase-associated protein 1 | 2 | 2 | 2 | 3 | 3 | 1.22 | 7.38E-01 |
| IPI00743696|IPI00873684 | Isoform 1 of Collagen alpha-1(IV) chain | 0 | 0 | 1 | 1 | 0 | 1.67 | 1.00E+00 |
| IPI00012795 | Eukaryotic translation initiation factor 3 subunit I | 0 | 0 | 0 | 1 | 1 | 1.67 | 1.00E+00 |
| IPI00291006 | Malate dehydrogenase| mitochondrial | 11 | 11 | 11 | 10 | 10 | 0.94 | 6.21E-01 |
| IPI00455510|IPI00479214|IPI00827535|IPI00916480|IPI00916962|IPI00917228|IPI00917401 | Isoform 2 of Prothymosin alpha | 2 | 2 | 0 | 2 | 0 | 0.56 | 1.00E+00 |
| IPI00478921 | Myelin protein zero | 0 | 0 | 1 | 0 | 1 | 1.67 | 1.00E+00 |
| IPI00026216|IPI00978387|IPI01015230 | Puromycin-sensitive aminopeptidase | 0 | 0 | 0 | 3 | 3 | 3.00 | 1.00E+00 |
| IPI00294398|IPI00298406|IPI00902588 | Isoform 1 of Hydroxyacyl-coenzyme A dehydrogenase| mitochondrial | 2 | 2 | 2 | 1 | 0 | 0.67 | 6.00E-01 |
| IPI00848226|IPI00966111|IPI00967792 | Guanine nucleotide-binding protein subunit beta-2-like 1 | 6 | 6 | 2 | 6 | 5 | 0.76 | 3.37E-01 |
| IPI00007702 | Heat shock-related 70 kDa protein 2 | 0 | 0 | 9 | 8 | 0 | 6.67 | 4.11E-05 |
| IPI00021700 | Proliferating cell nuclear antigen | 0 | 0 | 1 | 2 | 0 | 2.00 | 3.33E-01 |
| IPI00021290|IPI00394838|IPI00935456|IPI00983296 | ATP-citrate synthase | 0 | 0 | 0 | 2 | 1 | 2.00 | 1.00E+00 |
| IPI00550689|IPI00910144 | tRNA-splicing ligase RtcB homolog | 0 | 0 | 1 | 2 | 0 | 2.00 | 3.33E-01 |
| IPI00221384|IPI00329573|IPI00964552 | Isoform 2 of Collagen alpha-1(XII) chain | 0 | 0 | 0 | 4 | 2 | 3.00 | 1.00E+00 |
| IPI00479722 | Proteasome activator complex subunit 1 | 0 | 0 | 3 | 0 | 2 | 2.67 | 1.00E+00 |
| IPI00465028|IPI00797270 | triosephosphate isomerase isoform 2 | 8 | 10 | 8 | 6 | 8 | 0.83 | 4.91E-01 |
| IPI00217467 | Histone H1.4 | 4 | 4 | 6 | 5 | 4 | 1.20 | 4.14E-01 |
| IPI00001639 | Importin subunit beta-1 | 0 | 0 | 0 | 2 | 1 | 2.00 | 1.00E+00 |
| IPI00069750|IPI00100716|IPI00788826|IPI00797595|IPI00855912|IPI00856076 | Isoform 6 of Poly(U)-binding-splicing factor PUF60 | 0 | 0 | 0 | 4 | 2 | 3.00 | 1.00E+00 |
| IPI00021347 | Ubiquitin-conjugating enzyme E2 L3 | 0 | 0 | 1 | 2 | 0 | 2.00 | 3.33E-01 |
| IPI00016077 | Protein NipSnap homolog 2 | 3 | 3 | 1 | 2 | 3 | 0.75 | 3.57E-01 |
| IPI00221354|IPI00645208 | Isoform Short of RNA-binding protein FUS | 0 | 0 | 0 | 3 | 1 | 2.33 | 1.00E+00 |
| IPI00647693|IPI01011271 | asporin isoform 2 preproprotein | 2 | 2 | 2 | 1 | 0 | 0.67 | 6.00E-01 |
| IPI00843888|IPI01020694 | Calsequestrin-1 | 2 | 0 | 2 | 2 | 3 | 1.67 | 1.00E+00 |
| IPI00009865 | Keratin| type I cytoskeletal 10 | 5 | 4 | 3 | 4 | 4 | 0.85 | 5.00E-01 |
| IPI00000581|IPI00939174|IPI01012383|IPI01014215 | cDNA FLJ56307| highly similar to Ubiquitin thioesterase protein OTUB1 | 0 | 0 | 0 | 2 | 1 | 2.00 | 1.00E+00 |
| IPI00334190 | Stomatin-like protein 2 | 0 | 0 | 0 | 3 | 1 | 2.33 | 1.00E+00 |
| IPI00473014 | Destrin | 2 | 2 | 2 | 1 | 0 | 0.67 | 6.00E-01 |
| IPI00182469|IPI00182540|IPI00219725|IPI00219726|IPI00219727|IPI00219728|IPI00219730|IPI00219731|IPI00219732|IPI00219733|IPI00219734|IPI00219735|IPI00219737|IPI00219738|IPI00219739|IPI00219741|IPI00219742|IPI00219869|IPI00219870|IPI00219872|IPI00219873|IPI00219875|IPI00419482|IPI00845519|IPI00942869|IPI00977746|IPI00979192|IPI00983022|IPI01019120 | Isoform 1AB of Catenin delta-1 | 0 | 0 | 0 | 2 | 3 | 2.67 | 1.00E+00 |
| IPI00219156 | 60S ribosomal protein L30 | 0 | 0 | 0 | 4 | 3 | 3.33 | 1.00E+00 |
| IPI00024915|IPI00759663 | Isoform Mitochondrial of Peroxiredoxin-5| mitochondrial | 3 | 4 | 3 | 3 | 2 | 0.81 | 6.08E-01 |
| IPI00328753|IPI00337736|IPI00783726|IPI00909030 | Isoform 1 of Kinectin | 0 | 0 | 0 | 1 | 2 | 2.00 | 1.00E+00 |
| IPI00180240|IPI00220828|IPI00719405|IPI00815642|IPI00816288|IPI01011913|IPI01012854|IPI01013265|IPI01014272|IPI01015569 | Thymosin beta-4-like protein 3 | 2 | 1 | 1 | 0 | 1 | 0.67 | 5.00E-01 |
| IPI00006181|IPI00789582|IPI00791086 | Eukaryotic translation initiation factor 3 subunit D | 0 | 0 | 0 | 2 | 1 | 2.00 | 1.00E+00 |
| IPI00017726 | Isoform 1 of 3-hydroxyacyl-CoA dehydrogenase type-2 | 0 | 0 | 0 | 2 | 1 | 2.00 | 1.00E+00 |
| IPI00335168|IPI00789605|IPI00796366|IPI01021801|IPI01022179|IPI01022268|IPI01022653 | Isoform Non-muscle of Myosin light polypeptide 6 | 5 | 5 | 5 | 6 | 7 | 1.17 | 5.93E-01 |
| IPI00013475 | Tubulin beta-2A chain | 15 | 14 | 16 | 15 | 15 | 1.05 | 5.52E-01 |
| IPI00012837 | Kinesin-1 heavy chain | 1 | 4 | 4 | 4 | 5 | 1.52 | 3.78E-01 |
| IPI01018120 | Uncharacterized protein | 2 | 2 | 2 | 0 | 1 | 0.67 | 6.00E-01 |
| IPI00219153 | 60S ribosomal protein L22 | 0 | 0 | 0 | 2 | 1 | 2.00 | 1.00E+00 |
| IPI00012074|IPI00856037|IPI00856038|IPI00910614|IPI00941649 | Isoform 1 of Heterogeneous nuclear ribonucleoprotein R | 0 | 0 | 0 | 2 | 3 | 2.67 | 1.00E+00 |
| IPI00003348 | Guanine nucleotide-binding protein G(I)/G(S)/G(T) subunit beta-2 | 1 | 2 | 2 | 4 | 2 | 1.47 | 4.05E-01 |
| IPI00022977 | Creatine kinase B-type | 2 | 2 | 0 | 2 | 1 | 0.67 | 6.00E-01 |
| IPI00016613|IPI00741317|IPI00744507|IPI00784195 | Uncharacterized protein | 0 | 0 | 0 | 1 | 2 | 2.00 | 1.00E+00 |
| IPI00024976 | Mitochondrial import receptor subunit TOM22 homolog | 0 | 0 | 0 | 2 | 1 | 2.00 | 1.00E+00 |
| IPI00302925|IPI00784090 | 59 kDa protein | 0 | 0 | 0 | 2 | 1 | 2.00 | 1.00E+00 |
| IPI00217468 | Histone H1.5 | 0 | 0 | 2 | 0 | 1 | 2.00 | 1.00E+00 |
| IPI00643041|IPI00795671|IPI00796462 | GTP-binding nuclear protein Ran | 2 | 3 | 1 | 2 | 2 | 0.76 | 6.29E-01 |
| IPI00009328 | Eukaryotic initiation factor 4A-III | 0 | 0 | 0 | 3 | 2 | 2.67 | 1.00E+00 |
| IPI00289758|IPI01010797 | Calpain-2 catalytic subunit | 0 | 0 | 0 | 1 | 2 | 2.00 | 1.00E+00 |
| IPI00438229 | Isoform 1 of Transcription intermediary factor 1-beta | 0 | 0 | 1 | 2 | 0 | 2.00 | 3.33E-01 |
| IPI00016832|IPI00472442 | Isoform Short of Proteasome subunit alpha type-1 | 2 | 1 | 2 | 4 | 2 | 1.47 | 5.48E-01 |
| IPI00300371 | Isoform 1 of Splicing factor 3B subunit 3 | 0 | 0 | 0 | 3 | 4 | 3.33 | 1.00E+00 |
| IPI00220327 | Keratin| type II cytoskeletal 1 | 3 | 2 | 3 | 4 | 3 | 1.24 | 6.17E-01 |
| IPI00783625 | Isoform 1 of Serpin B5 | 5 | 3 | 3 | 3 | 1 | 0.67 | 5.94E-01 |
| IPI00746777 | Alcohol dehydrogenase class-3 | 0 | 0 | 0 | 2 | 1 | 2.00 | 1.00E+00 |
| IPI00179330|IPI00456429|IPI00719280|IPI00784990|IPI00793729|IPI00798155|IPI00969566|IPI01009501|IPI01010419|IPI01011412|IPI01011805|IPI01014205|IPI01014596|IPI01015113|IPI01015565|IPI01015749|IPI01015985|IPI01018966 | Ubiquitin-40S ribosomal protein S27a | 1 | 2 | 3 | 2 | 2 | 1.33 | 5.00E-01 |
| IPI00025084 | Calpain small subunit 1 | 0 | 0 | 0 | 2 | 1 | 2.00 | 1.00E+00 |
| IPI00024933 | Isoform 1 of 60S ribosomal protein L12 | 3 | 3 | 5 | 4 | 3 | 1.25 | 4.05E-01 |
| IPI00013296 | 40S ribosomal protein S18 | 1 | 1 | 2 | 3 | 1 | 1.50 | 3.71E-01 |
| IPI00010154 | Rab GDP dissociation inhibitor alpha | 0 | 0 | 0 | 5 | 4 | 4.00 | 1.00E+00 |
| IPI00872295 | cDNA FLJ56319| highly similar to Annexin A8 | 0 | 0 | 0 | 3 | 2 | 2.67 | 1.00E+00 |
| IPI00420108 | Dihydrolipoyllysine-residue succinyltransferase component of 2-oxoglutarate dehydrogenase complex| mitochondrial | 3 | 2 | 2 | 2 | 0 | 0.67 | 4.76E-01 |
| IPI00010402|IPI00514669|IPI00746352|IPI00955848 | Putative uncharacterized protein | 0 | 0 | 2 | 1 | 0 | 2.00 | 3.33E-01 |
| IPI00926925 | Uncharacterized protein | 10 | 8 | 8 | 4 | 8 | 0.77 | 2.06E-01 |
| IPI00073096|IPI00232492 | Isoform Alpha of Tripartite motif-containing protein 29 | 0 | 0 | 0 | 4 | 3 | 3.33 | 1.00E+00 |
| IPI00337455|IPI00477427|IPI00641544|IPI00658138|IPI00852975|IPI00853392|IPI00872734|IPI00937352|IPI00943329 | Isoform 5 of Troponin T| fast skeletal muscle | 5 | 5 | 5 | 3 | 4 | 0.83 | 5.81E-01 |
| IPI00023598 | Tubulin beta-4 chain | 0 | 0 | 0 | 13 | 12 | 9.33 | 1.00E+00 |
| IPI00017367|IPI00903145 | Uncharacterized protein | 1 | 0 | 0 | 0 | 0 | 0.67 | 1.00E+00 |
| IPI00645805|IPI00789848|IPI00929108 | Isovaleryl-CoA dehydrogenase| mitochondrial | 1 | 0 | 0 | 0 | 0 | 0.67 | 1.00E+00 |
| IPI00177008 | Phosphoglycolate phosphatase | 1 | 0 | 0 | 0 | 0 | 0.67 | 1.00E+00 |
| IPI00294073|IPI00645720 | Isoform 1 of Ubiquinone biosynthesis protein COQ7 homolog | 2 | 0 | 0 | 0 | 0 | 0.50 | 1.00E+00 |
| IPI00154509|IPI00219950|IPI00748423|IPI01009874 | Isoform 1 of Proteasome subunit alpha type-7-like | 0 | 1 | 0 | 0 | 0 | 0.67 | 1.00E+00 |
| IPI00000877|IPI00922838|IPI01011324 | Hypoxia up-regulated protein 1 | 0 | 1 | 0 | 0 | 0 | 0.67 | 1.00E+00 |
| IPI00003933|IPI00745553 | Isoform 1 of Hydroxyacylglutathione hydrolase| mitochondrial | 2 | 0 | 0 | 0 | 0 | 0.50 | 1.00E+00 |
| IPI00795816 | serine hydroxymethyltransferase| mitochondrial isoform 2 precursor | 0 | 2 | 0 | 0 | 0 | 0.50 | 1.00E+00 |
| IPI00005978|IPI00385786|IPI01009595|IPI01011463 | Serine/arginine-rich splicing factor 2 | 1 | 0 | 0 | 0 | 0 | 0.67 | 1.00E+00 |
| IPI00016457|IPI00029140|IPI00514595 | Isoform 1 of Carnitine O-acetyltransferase | 2 | 0 | 0 | 0 | 0 | 0.50 | 1.00E+00 |
| IPI00877934 | Isoform 2 of Histone-binding protein RBBP4 | 1 | 0 | 0 | 0 | 0 | 0.67 | 1.00E+00 |
| IPI00019329 | Dynein light chain 1| cytoplasmic | 0 | 3 | 0 | 0 | 0 | 0.40 | 1.00E+00 |
| IPI00218847|IPI00219861|IPI00410615 | Isoform 2 of Low molecular weight phosphotyrosine protein phosphatase | 0 | 1 | 0 | 0 | 0 | 0.67 | 1.00E+00 |
| IPI00220373 | Insulin-degrading enzyme | 1 | 0 | 0 | 0 | 0 | 0.67 | 1.00E+00 |
| IPI00027350 | Peroxiredoxin-2 | 1 | 0 | 0 | 0 | 0 | 0.67 | 1.00E+00 |
| IPI00295777|IPI01020783 | Glycerol-3-phosphate dehydrogenase [NAD+]| cytoplasmic | 0 | 2 | 0 | 0 | 0 | 0.50 | 1.00E+00 |
| IPI00007797 | Fatty acid-binding protein| epidermal | 0 | 1 | 1 | 1 | 1 | 1.33 | 6.67E-01 |
| IPI00062037 | Dynein light chain 2| cytoplasmic | 0 | 2 | 0 | 0 | 0 | 0.50 | 1.00E+00 |
| IPI00641582 | BAG family molecular chaperone regulator 3 | 1 | 0 | 0 | 0 | 0 | 0.67 | 1.00E+00 |
| IPI00017963|IPI00930678 | Small nuclear ribonucleoprotein Sm D2 | 0 | 1 | 0 | 0 | 0 | 0.67 | 1.00E+00 |
| IPI00096066|IPI00921931|IPI00945507 | Succinyl-CoA ligase [GDP-forming] subunit beta| mitochondrial | 0 | 1 | 0 | 0 | 0 | 0.67 | 1.00E+00 |
| IPI00926977 | 26S protease regulatory subunit 10B | 0 | 1 | 0 | 0 | 0 | 0.67 | 1.00E+00 |
| IPI00219077|IPI00514090|IPI00790203|IPI00793812 | cDNA FLJ52432| highly similar to Leukotriene A-4 hydrolase | 0 | 2 | 0 | 0 | 0 | 0.50 | 1.00E+00 |
| IPI00419373|IPI00455134 | Isoform 1 of Heterogeneous nuclear ribonucleoprotein A3 | 6 | 5 | 6 | 6 | 6 | 1.08 | 6.58E-01 |
| IPI00945379|IPI00969091 | Isoform 1 of E3 ubiquitin-protein ligase NEDD4 | 2 | 0 | 0 | 0 | 0 | 0.50 | 1.00E+00 |
| IPI00292130 | Dermatopontin | 0 | 2 | 0 | 0 | 0 | 0.50 | 1.00E+00 |
| IPI00029631 | Enhancer of rudimentary homolog | 0 | 2 | 0 | 0 | 0 | 0.50 | 1.00E+00 |
| IPI00007427|IPI00893607|IPI00893733|IPI00942032|IPI00952571 | AGR2 | 0 | 1 | 0 | 0 | 0 | 0.67 | 1.00E+00 |
| IPI00295851|IPI00982417 | Coatomer subunit beta | 1 | 0 | 1 | 1 | 1 | 1.33 | 1.00E+00 |
| IPI00022891 | ADP/ATP translocase 1 | 0 | 8 | 0 | 0 | 0 | 0.20 | 1.00E+00 |
| IPI00008359 | Keratin| type II cytoskeletal 2 oral | 0 | 7 | 0 | 0 | 0 | 0.22 | 1.00E+00 |
| IPI00384992 | Myosin light chain 4 | 0 | 3 | 0 | 0 | 0 | 0.40 | 1.00E+00 |
| IPI00239077 | Histidine triad nucleotide-binding protein 1 | 1 | 0 | 0 | 0 | 0 | 0.67 | 1.00E+00 |
| IPI00337541|IPI00964130 | NAD(P) transhydrogenase| mitochondrial | 0 | 1 | 0 | 0 | 0 | 0.67 | 1.00E+00 |
| IPI00218414 | Carbonic anhydrase 2 | 0 | 1 | 0 | 0 | 0 | 0.67 | 1.00E+00 |
| IPI00470829|IPI01012925 | 80 kDa protein | 2 | 3 | 2 | 2 | 2 | 0.86 | 7.57E-01 |
| IPI00295905 | Thioesterase superfamily member 5 | 0 | 1 | 0 | 0 | 0 | 0.67 | 1.00E+00 |
| IPI00747159|IPI00922810 | Isobutyryl-CoA dehydrogenase| mitochondrial | 0 | 2 | 0 | 0 | 0 | 0.50 | 1.00E+00 |
| IPI00395341|IPI00478774|IPI00936836 | Isoform 1 of Myomesin-3 | 1 | 0 | 0 | 0 | 0 | 0.67 | 1.00E+00 |
| IPI00243742 | Myosin light chain 3 | 0 | 1 | 0 | 0 | 0 | 0.67 | 1.00E+00 |
| IPI00179026|IPI00645446|IPI00900380 | Isoform 1 of Malignant T cell-amplified sequence 1 | 0 | 1 | 0 | 0 | 0 | 0.67 | 1.00E+00 |
| IPI00939159 | Adenylyl cyclase-associated protein | 3 | 0 | 0 | 0 | 0 | 0.40 | 1.00E+00 |
| IPI00012303|IPI00942368 | Isoform 1 of Selenium-binding protein 1 | 0 | 2 | 0 | 0 | 0 | 0.50 | 1.00E+00 |
| IPI00218606 | 40S ribosomal protein S23 | 1 | 0 | 0 | 0 | 0 | 0.67 | 1.00E+00 |
| IPI00554788|IPI01022070 | Keratin| type I cytoskeletal 18 | 0 | 2 | 0 | 0 | 0 | 0.50 | 1.00E+00 |
| IPI00003970|IPI00984697 | NADP-dependent malic enzyme| mitochondrial | 0 | 1 | 0 | 0 | 0 | 0.67 | 1.00E+00 |
| IPI00216138 | Transgelin | 0 | 3 | 0 | 0 | 0 | 0.40 | 1.00E+00 |
| IPI00290416|IPI00916847 | Isoform 1 of Obg-like ATPase 1 | 0 | 2 | 2 | 2 | 2 | 1.50 | 4.00E-01 |
| IPI00867533 | 60S ribosomal protein L6 | 0 | 1 | 0 | 0 | 0 | 0.67 | 1.00E+00 |
| IPI00470535|IPI00953206|IPI00953262|IPI00953650|IPI00978064|IPI01015859 | Dihydropyridine receptor alpha 2 subunit | 1 | 0 | 0 | 0 | 0 | 0.67 | 1.00E+00 |
| IPI00012728|IPI00401448|IPI00942534|IPI00965893 | Isoform 1 of Long-chain-fatty-acid--CoA ligase 1 | 0 | 2 | 0 | 0 | 0 | 0.50 | 1.00E+00 |
| IPI00328156|IPI00922603|IPI00976528 | Amine oxidase [flavin-containing] B | 0 | 1 | 0 | 0 | 0 | 0.67 | 1.00E+00 |
| IPI00008433 | 40S ribosomal protein S5 | 0 | 2 | 0 | 0 | 0 | 0.50 | 1.00E+00 |
| IPI00031804|IPI00294779|IPI01010397 | Isoform 1 of Voltage-dependent anion-selective channel protein 3 | 0 | 2 | 0 | 0 | 0 | 0.50 | 1.00E+00 |
| IPI00217872|IPI00219526|IPI01011356 | Isoform 2 of Phosphoglucomutase-1 | 3 | 5 | 1 | 4 | 1 | 0.60 | 7.22E-01 |
| IPI00440493 | ATP synthase subunit alpha| mitochondrial | 21 | 24 | 22 | 16 | 18 | 0.84 | 5.30E-01 |
| IPI00025092|IPI00384170|IPI00410261|IPI00412087|IPI00789615|IPI00797657|IPI00939358|IPI01015161|IPI01020911|IPI01021665|IPI01022367|IPI01022710 | Isoform 1 of Myosin-binding protein C| slow-type | 10 | 14 | 11 | 5 | 8 | 0.69 | 4.35E-01 |
| IPI00382844 | Aconitase (Fragment) | 11 | 14 | 12 | 8 | 9 | 0.79 | 6.07E-01 |
| IPI00220766|IPI01021703 | Isoform 1 of Lactoylglutathione lyase | 2 | 0 | 3 | 3 | 0 | 1.50 | 1.96E-01 |
| IPI00031812 | Nuclease-sensitive element-binding protein 1 | 2 | 3 | 4 | 2 | 4 | 1.24 | 7.27E-01 |
| IPI00215637|IPI00984839|IPI00985384 | ATP-dependent RNA helicase DDX3X | 3 | 2 | 2 | 5 | 5 | 1.43 | 5.73E-01 |
| IPI00291136|IPI00975947|IPI01009421|IPI01010585 | Collagen alpha-1(VI) chain | 1 | 0 | 0 | 2 | 2 | 1.56 | 1.00E+00 |
| IPI00010153 | 60S ribosomal protein L23 | 0 | 1 | 0 | 2 | 2 | 1.56 | 1.00E+00 |
| IPI00027423|IPI00550451 | serine/threonine-protein phosphatase PP1-alpha catalytic subunit isoform 3 | 4 | 2 | 2 | 7 | 7 | 1.58 | 4.26E-01 |
| IPI00465248 | Isoform alpha-enolase of Alpha-enolase | 10 | 9 | 8 | 8 | 10 | 0.92 | 3.70E-01 |
| IPI00007960|IPI00218585|IPI00410241|IPI00641231|IPI00910262|IPI01009588 | Isoform 1 of Periostin | 2 | 0 | 0 | 3 | 3 | 1.50 | 4.64E-01 |
| IPI00178352 | Isoform 1 of Filamin-C | 18 | 14 | 10 | 14 | 16 | 0.84 | 1.39E-01 |
| IPI00965327 | Uncharacterized protein | 5 | 6 | 3 | 4 | 6 | 0.82 | 3.19E-01 |
| IPI00016801|IPI01014382 | Glutamate dehydrogenase 1| mitochondrial | 5 | 4 | 5 | 3 | 2 | 0.79 | 5.73E-01 |
| IPI00007750|IPI00794663 | Tubulin alpha-4A chain | 11 | 13 | 13 | 14 | 12 | 1.08 | 3.89E-01 |
| IPI00217030 | 40S ribosomal protein S4| X isoform | 2 | 3 | 2 | 5 | 4 | 1.33 | 6.57E-01 |
| IPI00219018 | Glyceraldehyde-3-phosphate dehydrogenase | 3 | 5 | 4 | 2 | 3 | 0.80 | 6.89E-01 |
| IPI00012066|IPI00470509|IPI00790627|IPI01009186|IPI01022076 | poly(rC)-binding protein 2 isoform b | 4 | 0 | 3 | 5 | 4 | 1.67 | 1.00E+00 |
| IPI00015911|IPI00909143|IPI01011421 | Dihydrolipoyl dehydrogenase| mitochondrial | 8 | 6 | 5 | 6 | 7 | 0.88 | 3.48E-01 |
| IPI00303476 | ATP synthase subunit beta| mitochondrial | 22 | 24 | 23 | 20 | 22 | 0.94 | 5.38E-01 |
| IPI00002966 | Heat shock 70 kDa protein 4 | 1 | 0 | 0 | 4 | 2 | 2.00 | 1.00E+00 |
| IPI00216691 | Profilin-1 | 0 | 2 | 1 | 3 | 2 | 1.50 | 5.00E-01 |
| IPI00010790|IPI00643384 | Biglycan | 4 | 2 | 4 | 3 | 5 | 1.25 | 5.00E-01 |
| IPI00220637|IPI00514587 | Seryl-tRNA synthetase| cytoplasmic | 0 | 1 | 2 | 3 | 0 | 1.78 | 1.00E-01 |
| IPI00549725 | Phosphoglycerate mutase 1 | 9 | 6 | 9 | 11 | 7 | 1.18 | 3.69E-01 |
| IPI00005159|IPI00470573|IPI01014716 | Actin-related protein 2 | 3 | 0 | 5 | 2 | 3 | 1.73 | 1.00E+00 |
| IPI00384444 | Keratin| type I cytoskeletal 14 | 15 | 14 | 14 | 24 | 17 | 1.25 | 4.21E-01 |
| IPI00554648|IPI01021414 | Keratin| type II cytoskeletal 8 | 0 | 4 | 0 | 6 | 7 | 1.78 | 1.00E+00 |
| IPI00289499|IPI00925601 | Bifunctional purine biosynthesis protein PURH | 0 | 0 | 0 | 1 | 0 | 1.33 | 1.00E+00 |
| IPI00219678 | Eukaryotic translation initiation factor 2 subunit 1 | 0 | 0 | 0 | 2 | 0 | 1.67 | 1.00E+00 |
| IPI00293655 | ATP-dependent RNA helicase DDX1 | 0 | 0 | 0 | 2 | 0 | 1.67 | 1.00E+00 |
| IPI00216237 | 60S ribosomal protein L36 | 0 | 0 | 2 | 0 | 0 | 1.67 | 1.00E+00 |
| IPI00026089 | Splicing factor 3B subunit 1 | 0 | 0 | 0 | 1 | 0 | 1.33 | 1.00E+00 |
| IPI00028004 | Proteasome subunit beta type-3 | 0 | 0 | 0 | 1 | 0 | 1.33 | 1.00E+00 |
| IPI00009104|IPI01014133 | RuvB-like 2 | 0 | 0 | 0 | 1 | 0 | 1.33 | 1.00E+00 |
| IPI00419235|IPI00444582|IPI00642432 | Glutathione S-transferase Mu 5 | 0 | 0 | 1 | 0 | 0 | 1.33 | 1.00E+00 |
| IPI00004839 | Crk-like protein | 0 | 0 | 0 | 0 | 1 | 1.33 | 1.00E+00 |
| IPI00010796 | Protein disulfide-isomerase | 6 | 6 | 7 | 6 | 6 | 1.05 | 5.82E-01 |
| IPI00218830|IPI00329692 | Isoform Short of Glycylpeptide N-tetradecanoyltransferase 1 | 0 | 0 | 0 | 2 | 0 | 1.67 | 1.00E+00 |
| IPI00170796|IPI00184284|IPI01020820|IPI01021500 | Isoform 1 of Vacuolar protein sorting-associated protein 29 | 0 | 0 | 0 | 1 | 0 | 1.33 | 1.00E+00 |
| IPI00219757 | Glutathione S-transferase P | 0 | 0 | 0 | 1 | 0 | 1.33 | 1.00E+00 |
| IPI00550900 | Translationally-controlled tumor protein | 1 | 1 | 1 | 1 | 0 | 0.83 | 6.67E-01 |
| IPI00303207 | ATP-binding cassette sub-family E member 1 | 0 | 0 | 0 | 0 | 1 | 1.33 | 1.00E+00 |
| IPI00910422 | cDNA FLJ52802| highly similar to Eukaryotic translation initiation factor 3subunit 6-interacting protein | 0 | 0 | 0 | 1 | 0 | 1.33 | 1.00E+00 |
| IPI00029079|IPI00945620|IPI01013391 | GMP synthase [glutamine-hydrolyzing] | 0 | 0 | 0 | 0 | 2 | 1.67 | 1.00E+00 |
| IPI00022463|IPI00945626 | Serotransferrin | 0 | 0 | 1 | 0 | 0 | 1.33 | 1.00E+00 |
| IPI00022334 | Ornithine aminotransferase| mitochondrial | 0 | 0 | 0 | 2 | 0 | 1.67 | 1.00E+00 |
| IPI00239789|IPI00894474 | Isoform 2 of Katanin p60 ATPase-containing subunit A-like 2 | 0 | 0 | 0 | 0 | 1 | 1.33 | 1.00E+00 |
| IPI00060715 | BTB/POZ domain-containing protein KCTD12 | 0 | 0 | 3 | 0 | 0 | 2.00 | 1.00E+00 |
| IPI00023591 | Transcriptional activator protein Pur-alpha | 2 | 2 | 2 | 0 | 2 | 0.78 | 4.00E-01 |
| IPI00304417|IPI00304419 | Isocitrate dehydrogenase [NAD] subunit beta| mitochondrial precursor | 2 | 2 | 2 | 2 | 1 | 0.89 | 6.29E-01 |
| IPI00029601|IPI00062884 | Src substrate cortactin | 0 | 0 | 0 | 1 | 0 | 1.33 | 1.00E+00 |
| IPI00908513 | cDNA FLJ60039| highly similar to Proteasome subunit beta type 7 | 0 | 0 | 0 | 0 | 1 | 1.33 | 1.00E+00 |
| IPI00017256|IPI00377066|IPI00847168 | Ras suppressor protein 1 | 0 | 0 | 0 | 1 | 0 | 1.33 | 1.00E+00 |
| IPI00375441|IPI00641948|IPI00983652 | Isoform 1 of Far upstream element-binding protein 1 | 0 | 0 | 0 | 0 | 2 | 1.67 | 1.00E+00 |
| IPI00306332|IPI00791426|IPI00946221 | 60S ribosomal protein L24 | 0 | 0 | 0 | 1 | 0 | 1.33 | 1.00E+00 |
| IPI00420014 | Isoform 1 of U5 small nuclear ribonucleoprotein 200 kDa helicase | 0 | 0 | 0 | 2 | 0 | 1.67 | 1.00E+00 |
| IPI00793199 | annexin A4 | 0 | 0 | 1 | 0 | 0 | 1.33 | 1.00E+00 |
| IPI00654777|IPI00941255|IPI01013988 | cDNA FLJ36192 fis| clone TESTI2027450| highly similar to Eukaryotic translation initiation factor 3 subunit 5 | 0 | 0 | 0 | 4 | 0 | 2.33 | 1.00E+00 |
| IPI00005040|IPI00513827|IPI00895801|IPI01015888 | Isoform 1 of Medium-chain specific acyl-CoA dehydrogenase| mitochondrial | 0 | 0 | 0 | 0 | 1 | 1.33 | 1.00E+00 |
| IPI00306369|IPI00966877 | tRNA (cytosine(34)-C(5))-methyltransferase | 0 | 0 | 0 | 1 | 0 | 1.33 | 1.00E+00 |
| IPI00306960|IPI00647678 | Asparaginyl-tRNA synthetase| cytoplasmic | 0 | 0 | 0 | 2 | 0 | 1.67 | 1.00E+00 |
| IPI00376005|IPI00411704 | Isoform 2 of Eukaryotic translation initiation factor 5A-1 | 3 | 3 | 3 | 5 | 3 | 1.17 | 5.29E-01 |
| IPI00018278|IPI00218448 | Histone H2A.V | 0 | 0 | 0 | 4 | 0 | 2.33 | 1.00E+00 |
| IPI00141318 | Cytoskeleton-associated protein 4 | 0 | 0 | 0 | 1 | 0 | 1.33 | 1.00E+00 |
| IPI00215901|IPI00218988|IPI00922165|IPI01020958 | Isoform 1 of Adenylate kinase 2| mitochondrial | 0 | 0 | 0 | 2 | 0 | 1.67 | 1.00E+00 |
| IPI00784320|IPI00974117 | Protein FAM83H | 0 | 0 | 0 | 2 | 0 | 1.67 | 1.00E+00 |
| IPI00411680|IPI00828189|IPI00922359|IPI00947458 | Isoform 1 of Protein-L-isoaspartate(D-aspartate) O-methyltransferase | 0 | 0 | 1 | 0 | 0 | 1.33 | 1.00E+00 |
| IPI00220487|IPI00456049 | Isoform 1 of ATP synthase subunit d| mitochondrial | 0 | 0 | 0 | 1 | 0 | 1.33 | 1.00E+00 |
| IPI00007402|IPI00981775 | Importin-7 | 0 | 0 | 0 | 1 | 0 | 1.33 | 1.00E+00 |
| IPI00221089|IPI00977844 | 40S ribosomal protein S13 | 0 | 0 | 0 | 1 | 0 | 1.33 | 1.00E+00 |
| IPI00554521|IPI00981633|IPI00982137|IPI01010800|IPI01013419 | Ferritin heavy chain | 0 | 0 | 0 | 1 | 0 | 1.33 | 1.00E+00 |
| IPI00012503|IPI00219825 | Isoform Sap-mu-0 of Proactivator polypeptide | 0 | 0 | 1 | 0 | 0 | 1.33 | 1.00E+00 |
| IPI00550239|IPI00979041 | Histone H1.0 | 0 | 0 | 0 | 0 | 2 | 1.67 | 1.00E+00 |
| IPI00299155 | Proteasome subunit alpha type-4 | 3 | 3 | 3 | 3 | 2 | 0.92 | 6.08E-01 |
| IPI00010471 | Plastin-2 | 0 | 0 | 4 | 0 | 0 | 2.33 | 1.00E+00 |
| IPI00479306 | Isoform 1 of Proteasome subunit beta type-5 | 0 | 0 | 0 | 0 | 1 | 1.33 | 1.00E+00 |
| IPI00220528 | Small nuclear ribonucleoprotein F | 0 | 0 | 0 | 1 | 0 | 1.33 | 1.00E+00 |
| IPI00003519|IPI00917777|IPI01012385 | 116 kDa U5 small nuclear ribonucleoprotein component | 0 | 0 | 0 | 1 | 0 | 1.33 | 1.00E+00 |
| IPI00024993 | Enoyl-CoA hydratase| mitochondrial | 0 | 0 | 0 | 0 | 1 | 1.33 | 1.00E+00 |
| IPI00465128|IPI00640922|IPI00645431|IPI00793299|IPI00829720|IPI00892541|IPI00893932|IPI00894269|IPI00894467 | Isoform 1 of Large proline-rich protein BAG6 | 0 | 0 | 0 | 0 | 1 | 1.33 | 1.00E+00 |
| IPI00304692|IPI00939558 | Heterogeneous nuclear ribonucleoprotein G | 0 | 0 | 0 | 2 | 0 | 1.67 | 1.00E+00 |
| IPI00011603|IPI01014869|IPI01015295 | 26S proteasome non-ATPase regulatory subunit 3 | 0 | 0 | 0 | 0 | 2 | 1.67 | 1.00E+00 |
| IPI00013122 | Hsp90 co-chaperone Cdc37 | 0 | 0 | 0 | 1 | 0 | 1.33 | 1.00E+00 |
| IPI00294536|IPI00941907 | cDNA FLJ51909| highly similar to Serine-threonine kinase receptor-associatedprotein | 0 | 0 | 0 | 0 | 2 | 1.67 | 1.00E+00 |
| IPI00329633|IPI00908949 | Threonyl-tRNA synthetase| cytoplasmic | 0 | 0 | 0 | 3 | 0 | 2.00 | 1.00E+00 |
| IPI00026167|IPI00893746 | NHP2-like protein 1 | 0 | 0 | 2 | 0 | 0 | 1.67 | 1.00E+00 |
| IPI00027422|IPI00220845|IPI00220846|IPI00220847 | Isoform Beta-4C of Integrin beta-4 | 0 | 0 | 0 | 0 | 3 | 2.00 | 1.00E+00 |
| IPI00029012|IPI01014951 | Eukaryotic translation initiation factor 3 subunit A | 0 | 0 | 0 | 0 | 2 | 1.67 | 1.00E+00 |
| IPI00100197|IPI00397571 | Isoform 1 of NSFL1 cofactor p47 | 0 | 0 | 0 | 2 | 0 | 1.67 | 1.00E+00 |
| IPI00783987 | Complement C3 (Fragment) | 0 | 0 | 1 | 0 | 0 | 1.33 | 1.00E+00 |
| IPI00000684|IPI00217816|IPI00607787 | Isoform AGX2 of UDP-N-acetylhexosamine pyrophosphorylase | 0 | 0 | 0 | 0 | 1 | 1.33 | 1.00E+00 |
| IPI00784614|IPI00784808|IPI00784936 | septin-9 isoform a | 0 | 0 | 0 | 2 | 0 | 1.67 | 1.00E+00 |
| IPI00024403 | Copine-3 | 0 | 0 | 0 | 2 | 0 | 1.67 | 1.00E+00 |
| IPI00556173 | Isoform 6 of Interleukin enhancer-binding factor 3 | 0 | 0 | 0 | 4 | 0 | 2.33 | 1.00E+00 |
| IPI00797738 | Cytochrome c oxidase subunit 6B1 | 0 | 0 | 0 | 1 | 0 | 1.33 | 1.00E+00 |
| IPI00414717|IPI00641153|IPI00923461 | Isoform 2 of Golgi apparatus protein 1 | 0 | 0 | 0 | 0 | 2 | 1.67 | 1.00E+00 |
| IPI00017672 | cDNA FLJ25678 fis| clone TST04067| highly similar to PURINE NUCLEOSIDE PHOSPHORYLASE | 0 | 0 | 0 | 1 | 0 | 1.33 | 1.00E+00 |
| IPI00012535 | DnaJ homolog subfamily A member 1 | 0 | 0 | 0 | 0 | 1 | 1.33 | 1.00E+00 |
| IPI00021187|IPI00788942 | Isoform 1 of RuvB-like 1 | 0 | 0 | 0 | 1 | 0 | 1.33 | 1.00E+00 |
| IPI00217561|IPI00217562|IPI00217563|IPI00293305|IPI00549336 | Isoform Beta-1C of Integrin beta-1 | 0 | 0 | 0 | 0 | 1 | 1.33 | 1.00E+00 |
| IPI00017704 | Coactosin-like protein | 0 | 0 | 1 | 0 | 0 | 1.33 | 1.00E+00 |
| IPI00465431 | Galectin-3 | 0 | 0 | 0 | 2 | 0 | 1.67 | 1.00E+00 |
| IPI00171199|IPI00419249 | Isoform 2 of Proteasome subunit alpha type-3 | 0 | 0 | 1 | 0 | 0 | 1.33 | 1.00E+00 |
| IPI00012268|IPI01014808 | 26S proteasome non-ATPase regulatory subunit 2 | 1 | 1 | 2 | 1 | 1 | 1.17 | 7.00E-01 |
| IPI00026105|IPI00479510|IPI00479934|IPI00909685|IPI00943320|IPI00973807|IPI00977865 | Isoform SCPx of Non-specific lipid-transfer protein | 0 | 0 | 1 | 0 | 0 | 1.33 | 1.00E+00 |
| IPI00031556|IPI00830039 | Isoform 1 of Splicing factor U2AF 65 kDa subunit | 0 | 0 | 0 | 2 | 0 | 1.67 | 1.00E+00 |
| IPI00244391 | Xanthine dehydrogenase/oxidase | 0 | 0 | 1 | 0 | 0 | 1.33 | 1.00E+00 |
| IPI00027270|IPI00433834 | 60S ribosomal protein L26 | 0 | 0 | 0 | 1 | 0 | 1.33 | 1.00E+00 |
| IPI00030131|IPI00181409 | Isoform Beta of Lamina-associated polypeptide 2| isoforms beta/gamma | 0 | 0 | 0 | 2 | 0 | 1.67 | 1.00E+00 |
| IPI00430812|IPI00430813|IPI00430814|IPI00895806|IPI00895852|IPI00895911|IPI00967344|IPI00977873 | Isoform 1 of Cellular nucleic acid-binding protein | 0 | 0 | 2 | 0 | 0 | 1.67 | 1.00E+00 |
| IPI00024046|IPI01013272 | cDNA FLJ52398| highly similar to Cadherin-13 | 0 | 0 | 3 | 0 | 0 | 2.00 | 1.00E+00 |
| IPI00025091 | 40S ribosomal protein S11 | 0 | 0 | 0 | 1 | 0 | 1.33 | 1.00E+00 |
| IPI00025874 | Dolichyl-diphosphooligosaccharide--protein glycosyltransferase subunit 1 precursor | 0 | 0 | 0 | 1 | 0 | 1.33 | 1.00E+00 |
| IPI00549467 | Omega-amidase NIT2 | 0 | 0 | 0 | 1 | 0 | 1.33 | 1.00E+00 |
| IPI00549189 | Thimet oligopeptidase | 0 | 0 | 1 | 0 | 0 | 1.33 | 1.00E+00 |
| IPI00006379 | Nucleolar protein 58 | 0 | 0 | 0 | 1 | 0 | 1.33 | 1.00E+00 |
| IPI00156689|IPI00942344|IPI01010637 | Synaptic vesicle membrane protein VAT-1 homolog | 0 | 0 | 0 | 3 | 0 | 2.00 | 1.00E+00 |
| IPI00220194|IPI00909237 | Solute carrier family 2| facilitated glucose transporter member 1 | 0 | 0 | 0 | 3 | 0 | 2.00 | 1.00E+00 |
| IPI00293665 | Keratin| type II cytoskeletal 6B | 0 | 0 | 0 | 16 | 0 | 6.33 | 1.00E+00 |
| IPI00176903 | Isoform 1 of Polymerase I and transcript release factor | 3 | 3 | 4 | 3 | 3 | 1.08 | 6.17E-01 |
| IPI00022256|IPI00619900|IPI00909772|IPI01009864|IPI01015530 | Isoform 1 of AP-2 complex subunit mu | 0 | 0 | 1 | 0 | 0 | 1.33 | 1.00E+00 |
| IPI00298497|IPI00965713 | Fibrinogen beta chain | 0 | 0 | 0 | 6 | 0 | 3.00 | 1.00E+00 |
| IPI00012442 | Ras GTPase-activating protein-binding protein 1 | 0 | 0 | 0 | 2 | 0 | 1.67 | 1.00E+00 |
| IPI00872762 | Succinyl-CoA ligase [GDP-forming] subunit alpha| mitochondrial | 2 | 2 | 2 | 1 | 2 | 0.89 | 6.29E-01 |
| IPI00216951|IPI01009538 | Aspartyl-tRNA synthetase| cytoplasmic | 0 | 0 | 0 | 1 | 0 | 1.33 | 1.00E+00 |
| IPI00026952 | Plakophilin-3 | 0 | 0 | 0 | 5 | 0 | 2.67 | 1.00E+00 |
| IPI00022078|IPI00923597 | Protein NDRG1 | 0 | 0 | 0 | 3 | 0 | 2.00 | 1.00E+00 |
| IPI00008223|IPI00642549 | UV excision repair protein RAD23 homolog B | 0 | 0 | 0 | 1 | 0 | 1.33 | 1.00E+00 |
| IPI00012345|IPI00215879|IPI00556297 | Isoform SRP55-1 of Serine/arginine-rich splicing factor 6 | 0 | 0 | 0 | 1 | 0 | 1.33 | 1.00E+00 |
| IPI00010133 | Coronin-1A | 0 | 0 | 2 | 0 | 0 | 1.67 | 1.00E+00 |
| IPI00025464|IPI00872143 | Metallothionein-1F | 0 | 0 | 0 | 1 | 0 | 1.33 | 1.00E+00 |
| IPI00026546 | Platelet-activating factor acetylhydrolase IB subunit beta | 0 | 0 | 0 | 1 | 0 | 1.33 | 1.00E+00 |
| IPI00009904 | Protein disulfide-isomerase A4 | 0 | 0 | 0 | 0 | 1 | 1.33 | 1.00E+00 |
| IPI00924816 | Myotrophin | 0 | 0 | 0 | 0 | 1 | 1.33 | 1.00E+00 |
| IPI00220301 | Peroxiredoxin-6 | 2 | 2 | 2 | 2 | 1 | 0.89 | 6.29E-01 |
| IPI00007423|IPI00759824 | Isoform 1 of Acidic leucine-rich nuclear phosphoprotein 32 family member B | 0 | 0 | 0 | 1 | 0 | 1.33 | 1.00E+00 |
| IPI00304840 | Isoform 2C2 of Collagen alpha-2(VI) chain | 0 | 0 | 2 | 0 | 0 | 1.67 | 1.00E+00 |
| IPI00026202 | 60S ribosomal protein L18a | 0 | 0 | 0 | 1 | 0 | 1.33 | 1.00E+00 |
| IPI00007280|IPI00550234 | Isoform 2 of Actin-related protein 2/3 complex subunit 5 | 0 | 0 | 1 | 0 | 0 | 1.33 | 1.00E+00 |
| IPI00247583|IPI00845507 | 60S ribosomal protein L21 | 0 | 0 | 0 | 0 | 1 | 1.33 | 1.00E+00 |
| IPI00783872 | Isoform 1 of Caprin-1 | 0 | 0 | 0 | 1 | 0 | 1.33 | 1.00E+00 |
| IPI00031420 | UDP-glucose 6-dehydrogenase | 0 | 0 | 0 | 2 | 0 | 1.67 | 1.00E+00 |
| IPI00020986 | Lumican | 2 | 2 | 2 | 2 | 0 | 0.78 | 4.00E-01 |
| IPI00397358|IPI00746004 | Similar to ribosomal protein S27 | 0 | 0 | 0 | 0 | 1 | 1.33 | 1.00E+00 |
| IPI00216348|IPI00302712|IPI00744015|IPI00827813|IPI00827859|IPI00915993|IPI01015321 | Isoform 2C of Cytoplasmic dynein 1 intermediate chain 2 | 0 | 0 | 0 | 2 | 0 | 1.67 | 1.00E+00 |
| IPI00221091 | 40S ribosomal protein S15a | 0 | 0 | 1 | 0 | 0 | 1.33 | 1.00E+00 |
| IPI00007611 | ATP synthase subunit O| mitochondrial | 2 | 2 | 2 | 1 | 2 | 0.89 | 6.29E-01 |
| IPI00001676|IPI00290543 | Isoform 2 of Nuclear protein localization protein 4 homolog | 0 | 0 | 0 | 1 | 0 | 1.33 | 1.00E+00 |
| IPI00216592|IPI00477313|IPI00759596|IPI01013087 | Isoform C1 of Heterogeneous nuclear ribonucleoproteins C1/C2 | 0 | 0 | 0 | 2 | 0 | 1.67 | 1.00E+00 |
| IPI00304903 | Cornifin-B | 0 | 0 | 0 | 1 | 0 | 1.33 | 1.00E+00 |
| IPI00943181 | Uncharacterized protein | 0 | 0 | 0 | 2 | 0 | 1.67 | 1.00E+00 |
| IPI00976704 | non-histone chromosomal protein HMG-17-like | 0 | 0 | 1 | 0 | 0 | 1.33 | 1.00E+00 |
| IPI00025019 | Proteasome subunit beta type-1 | 0 | 0 | 0 | 3 | 0 | 2.00 | 1.00E+00 |
| IPI00060181 | EF-hand domain-containing protein D2 | 0 | 0 | 2 | 0 | 0 | 1.67 | 1.00E+00 |
| IPI00220416|IPI00790644|IPI01012160 | Cytochrome b-c1 complex subunit 7 | 0 | 0 | 0 | 1 | 0 | 1.33 | 1.00E+00 |
| IPI00017292|IPI00927799 | Isoform 1 of Catenin beta-1 | 0 | 0 | 0 | 2 | 0 | 1.67 | 1.00E+00 |
| IPI00027285|IPI00329512|IPI00395674|IPI01018641 | Isoform SM-B' of Small nuclear ribonucleoprotein-associated proteins B and B' | 0 | 0 | 0 | 1 | 0 | 1.33 | 1.00E+00 |
| IPI00298301 | Myosin-3 | 0 | 0 | 30 | 0 | 0 | 11.00 | 1.00E+00 |
| IPI00032826|IPI00218038|IPI00892521 | Hsc70-interacting protein | 0 | 0 | 0 | 2 | 0 | 1.67 | 1.00E+00 |
| IPI00024919|IPI00374151 | Thioredoxin-dependent peroxide reductase| mitochondrial | 0 | 0 | 0 | 1 | 0 | 1.33 | 1.00E+00 |
| IPI00020101|IPI00152906|IPI00303133|IPI00329665|IPI00419833|IPI00554798|IPI00646240|IPI00794461|IPI00930174|IPI00930570 | Histone H2B type 1-C/E/F/G/I | 3 | 3 | 2 | 3 | 3 | 0.92 | 6.08E-01 |
| IPI00413778|IPI00647507 | FKBP1A protein | 0 | 0 | 2 | 0 | 0 | 1.67 | 1.00E+00 |
| IPI00304589 | Isoform 1 of 182 kDa tankyrase-1-binding protein | 0 | 0 | 0 | 1 | 0 | 1.33 | 1.00E+00 |
| IPI00104050 | Thyroid hormone receptor-associated protein 3 | 0 | 0 | 0 | 1 | 0 | 1.33 | 1.00E+00 |
| IPI00030243|IPI00219445 | Isoform 1 of Proteasome activator complex subunit 3 | 0 | 0 | 0 | 2 | 0 | 1.67 | 1.00E+00 |
| IPI00651653|IPI00651677|IPI00889541|IPI01022339|IPI01022656 | probable ATP-dependent RNA helicase DDX17 isoform 3 | 0 | 0 | 0 | 2 | 0 | 1.67 | 1.00E+00 |
| IPI00021435 | 26S protease regulatory subunit 7 | 0 | 0 | 0 | 2 | 0 | 1.67 | 1.00E+00 |
| IPI00003817 | Rho GDP-dissociation inhibitor 2 | 0 | 0 | 1 | 0 | 0 | 1.33 | 1.00E+00 |
| IPI00010204|IPI00843996 | Serine/arginine-rich splicing factor 3 | 0 | 0 | 0 | 1 | 0 | 1.33 | 1.00E+00 |
| IPI00003949 | Ubiquitin-conjugating enzyme E2 N | 2 | 2 | 2 | 2 | 0 | 0.78 | 4.00E-01 |
| IPI00220362 | 10 kDa heat shock protein| mitochondrial | 1 | 1 | 1 | 0 | 1 | 0.83 | 6.67E-01 |
| IPI00020672|IPI00956400|IPI00956436|IPI00956642|IPI00979514 | Isoform 1 of Dipeptidyl peptidase 3 | 0 | 0 | 0 | 1 | 0 | 1.33 | 1.00E+00 |
| IPI00218993 | Isoform Beta of Heat shock protein 105 kDa | 0 | 0 | 0 | 0 | 1 | 1.33 | 1.00E+00 |
| IPI00014850|IPI00643342 | Astrocytic phosphoprotein PEA-15 | 0 | 0 | 0 | 0 | 1 | 1.33 | 1.00E+00 |
| IPI00646748 | Uncharacterized protein | 0 | 0 | 0 | 0 | 19 | 7.33 | 1.00E+00 |
| IPI00306436|IPI00412752|IPI00784414 | Isoform Del-701 of Signal transducer and activator of transcription 3 | 0 | 0 | 0 | 3 | 0 | 2.00 | 1.00E+00 |
| IPI00008164 | Prolyl endopeptidase | 0 | 0 | 0 | 0 | 2 | 1.67 | 1.00E+00 |
| IPI00017964|IPI00878876|IPI00879750 | Small nuclear ribonucleoprotein Sm D3 | 0 | 0 | 2 | 0 | 0 | 1.67 | 1.00E+00 |
| IPI00550021|IPI00651660|IPI00877999 | 60S ribosomal protein L3 | 0 | 0 | 0 | 3 | 0 | 2.00 | 1.00E+00 |
| IPI00298860|IPI00903112|IPI00925547 | cDNA FLJ78440| highly similar to Human lactoferrin | 0 | 0 | 0 | 1 | 0 | 1.33 | 1.00E+00 |
| IPI00015897|IPI00885057 | Isoform 1 of Cysteine and histidine-rich domain-containing protein 1 | 0 | 0 | 0 | 1 | 0 | 1.33 | 1.00E+00 |
| IPI00216984 | Calmodulin-like protein 3 | 0 | 0 | 1 | 0 | 0 | 1.33 | 1.00E+00 |
| IPI00329801 | Annexin A5 | 5 | 5 | 4 | 5 | 5 | 0.94 | 5.86E-01 |
| IPI00013468|IPI00514701|IPI00644108|IPI01010654 | Isoform 1 of Mitotic checkpoint protein BUB3 | 0 | 0 | 0 | 0 | 1 | 1.33 | 1.00E+00 |
| IPI00026670|IPI00410162|IPI00917694 | Transcription elongation factor B polypeptide 2 | 0 | 0 | 0 | 0 | 1 | 1.33 | 1.00E+00 |
| IPI00015018 | Inorganic pyrophosphatase | 0 | 0 | 0 | 2 | 0 | 1.67 | 1.00E+00 |
| IPI00005719|IPI00334174|IPI00917079 | Isoform 1 of Ras-related protein Rab-1A | 2 | 2 | 0 | 2 | 2 | 0.78 | 4.00E-01 |
| IPI00977359 | HLA class I histocompatibility antigen| A-74 alpha chain-like isoform 10 | 0 | 0 | 1 | 0 | 0 | 1.33 | 1.00E+00 |
| IPI00010105 | Eukaryotic translation initiation factor 6 | 0 | 0 | 0 | 2 | 0 | 1.67 | 1.00E+00 |
| IPI00024664|IPI00375145 | Isoform Long of Ubiquitin carboxyl-terminal hydrolase 5 | 2 | 2 | 1 | 2 | 2 | 0.89 | 6.29E-01 |
| IPI00893431 | cDNA FLJ53410| highly similar to Eukaryotic translation initiation factor 3 subunit 9 | 0 | 0 | 0 | 2 | 0 | 1.67 | 1.00E+00 |
| IPI00006980 | UPF0568 protein C14orf166 | 0 | 0 | 1 | 0 | 0 | 1.33 | 1.00E+00 |
| IPI00000875|IPI00937615 | cDNA FLJ56389| highly similar to Elongation factor 1-gamma | 4 | 6 | 4 | 4 | 5 | 0.89 | 6.01E-01 |
| IPI00013917 | 40S ribosomal protein S12 | 2 | 4 | 2 | 3 | 2 | 0.83 | 6.43E-01 |
| IPI00022744|IPI00219762|IPI00219994 | Isoform 1 of Exportin-2 | 2 | 0 | 0 | 0 | 1 | 0.67 | 3.33E-01 |
| IPI00006482|IPI00646182|IPI00977640 | Isoform Long of Sodium/potassium-transporting ATPase subunit alpha-1 | 4 | 0 | 0 | 0 | 3 | 0.67 | 2.86E-02 |
| IPI00216975 | Isoform 2 of Tropomyosin alpha-4 chain | 14 | 0 | 11 | 0 | 0 | 0.58 | 1.00E+00 |
| IPI00004358 | Glycogen phosphorylase| brain form | 0 | 8 | 0 | 4 | 0 | 0.47 | 1.00E+00 |
| IPI00027223 | Isocitrate dehydrogenase [NADP] cytoplasmic | 2 | 0 | 2 | 2 | 1 | 1.33 | 1.00E+00 |
| IPI00952671 | T-complex protein 1 subunit eta isoform c | 0 | 2 | 1 | 2 | 2 | 1.33 | 6.00E-01 |
| IPI00442073|IPI00978767 | Cysteine and glycine-rich protein 1 | 2 | 0 | 1 | 0 | 0 | 0.67 | 1.00E+00 |
| IPI00025276|IPI00644296|IPI00892681|IPI00895929|IPI00942649 | Isoform XB of Tenascin-X | 2 | 0 | 1 | 0 | 0 | 0.67 | 1.00E+00 |
| IPI00025329 | 60S ribosomal protein L19 | 1 | 0 | 1 | 0 | 2 | 1.33 | 1.00E+00 |
| IPI00021405 | Isoform A of Prelamin-A/C | 18 | 18 | 20 | 17 | 20 | 1.05 | 5.43E-01 |
| IPI00011126|IPI01015427 | 26S protease regulatory subunit 4 | 2 | 0 | 0 | 2 | 3 | 1.33 | 4.29E-01 |
| IPI00022434|IPI00745872|IPI00966829 | Uncharacterized protein | 1 | 2 | 2 | 1 | 0 | 0.80 | 5.00E-01 |
| IPI00465436 | Catalase | 2 | 1 | 0 | 1 | 2 | 0.80 | 3.00E-01 |
| IPI00005614|IPI00328230|IPI00333015 | Isoform Long of Spectrin beta chain| brain 1 | 7 | 5 | 7 | 8 | 5 | 1.10 | 4.15E-01 |
| IPI00296635|IPI00789251|IPI01014616 | 1|4-alpha-glucan-branching enzyme | 1 | 1 | 0 | 2 | 0 | 0.83 | 1.00E+00 |
| IPI00013485|IPI00979595 | 40S ribosomal protein S2 | 2 | 1 | 0 | 2 | 1 | 0.80 | 6.00E-01 |
| IPI00456969 | Cytoplasmic dynein 1 heavy chain 1 | 2 | 1 | 0 | 1 | 2 | 0.80 | 3.00E-01 |
| IPI00000105 | Major vault protein | 0 | 1 | 0 | 2 | 1 | 1.33 | 1.00E+00 |
| IPI00007935|IPI00553102|IPI00966347|IPI00966603 | Isoform 1 of PDZ and LIM domain protein 5 | 0 | 1 | 1 | 2 | 0 | 1.33 | 3.33E-01 |
| IPI00376798|IPI00746438 | Isoform 1 of 60S ribosomal protein L11 | 0 | 1 | 2 | 1 | 0 | 1.33 | 3.33E-01 |
| IPI00479946 | STIP1 protein | 0 | 1 | 0 | 1 | 2 | 1.33 | 1.00E+00 |
| IPI00071509 | Isoform 2 of Plakophilin-1 | 7 | 7 | 5 | 11 | 5 | 1.00 | 4.30E-01 |
| IPI00216457|IPI00339274 | Histone H2A type 2-A | 4 | 5 | 4 | 5 | 7 | 1.15 | 5.35E-01 |
| IPI00021840 | 40S ribosomal protein S6 | 2 | 0 | 0 | 3 | 2 | 1.33 | 1.00E+00 |
| IPI00023919|IPI00555749|IPI00745502 | 26S protease regulatory subunit 8 | 0 | 1 | 0 | 3 | 1 | 1.56 | 1.00E+00 |
| IPI00186290 | Elongation factor 2 | 17 | 19 | 17 | 21 | 19 | 1.05 | 5.07E-01 |
| IPI00465315|IPI00917605 | Cytochrome c | 2 | 2 | 3 | 1 | 1 | 0.89 | 7.14E-01 |
| IPI00478231 | Transforming protein RhoA | 1 | 1 | 2 | 0 | 0 | 0.83 | 1.00E+00 |
| IPI00867509|IPI00943173|IPI01011988|IPI01022161 | Coronin-1C_i3 protein | 1 | 1 | 0 | 2 | 0 | 0.83 | 1.00E+00 |
| IPI00010271|IPI00219675 | Isoform A of Ras-related C3 botulinum toxin substrate 1 | 1 | 0 | 1 | 0 | 2 | 1.33 | 1.00E+00 |
| IPI00646779 | TUBB6 protein | 0 | 9 | 0 | 10 | 9 | 1.33 | 1.00E+00 |
| IPI00215746 | Fatty acid-binding protein| adipocyte | 2 | 1 | 0 | 1 | 2 | 0.80 | 3.00E-01 |
| IPI00296922 | Laminin subunit beta-2 | 1 | 1 | 2 | 0 | 0 | 0.83 | 1.00E+00 |
| IPI00045109 | Histone H2A type 1-A | 5 | 6 | 0 | 6 | 5 | 0.72 | 1.06E-01 |
| IPI00027497|IPI00908881|IPI00910781 | Glucose-6-phosphate isomerase | 2 | 3 | 3 | 1 | 2 | 0.86 | 7.14E-01 |
| IPI00916111|IPI00952583 | Malate dehydrogenase| cytoplasmic | 3 | 4 | 4 | 5 | 3 | 1.11 | 4.05E-01 |
| IPI00221035|IPI00419473 | Isoform 1 of Transcription factor BTF3 | 1 | 0 | 0 | 1 | 2 | 1.33 | 1.00E+00 |
| IPI00550363|IPI00647915 | Transgelin-2 | 4 | 6 | 6 | 7 | 4 | 1.11 | 2.60E-01 |
| IPI00218728 | Isoform 1 of Platelet-activating factor acetylhydrolase IB subunit alpha | 2 | 2 | 3 | 4 | 0 | 1.11 | 1.19E-01 |
| IPI00020501|IPI00024870|IPI00743857|IPI00744256|IPI00873982 | Myosin-11 | 8 | 8 | 0 | 5 | 10 | 0.67 | 2.62E-03 |
| IPI00759776|IPI00909239|IPI00921118 | alpha-actinin-1 isoform a | 14 | 14 | 12 | 22 | 21 | 1.29 | 5.13E-01 |
| IPI00219622 | Proteasome subunit alpha type-2 | 2 | 2 | 3 | 4 | 0 | 1.11 | 1.19E-01 |
| IPI00020599 | Calreticulin | 3 | 5 | 4 | 6 | 4 | 1.13 | 4.19E-01 |
| IPI00784154 | 60 kDa heat shock protein| mitochondrial | 9 | 5 | 7 | 10 | 7 | 1.13 | 5.80E-01 |
| IPI00479186 | Isoform M2 of Pyruvate kinase isozymes M1/M2 | 17 | 17 | 13 | 18 | 19 | 0.98 | 3.42E-01 |
| IPI00419880 | 40S ribosomal protein S3a | 0 | 2 | 1 | 3 | 1 | 1.33 | 4.00E-01 |
| IPI00450768 | Keratin| type I cytoskeletal 17 | 12 | 13 | 0 | 16 | 16 | 0.86 | 1.44E-03 |
| IPI00009867 | Keratin| type II cytoskeletal 5 | 12 | 13 | 11 | 14 | 14 | 1.04 | 5.50E-01 |
| IPI00413344 | Cofilin-2 | 3 | 2 | 0 | 4 | 0 | 0.67 | 1.00E+00 |
| IPI00554711 | Junction plakoglobin | 13 | 13 | 7 | 17 | 9 | 0.86 | 6.34E-01 |
| IPI00418169|IPI00455315 | Isoform 2 of Annexin A2 | 13 | 10 | 11 | 12 | 14 | 1.07 | 3.89E-01 |
| IPI00843765|IPI00844215 | Isoform 3 of Spectrin alpha chain| brain | 12 | 9 | 5 | 16 | 6 | 0.87 | 6.95E-01 |
| IPI00789324 | Uncharacterized protein | 12 | 14 | 10 | 18 | 17 | 1.14 | 5.18E-01 |
| IPI00000816 | Isoform 1 of 14-3-3 protein epsilon | 5 | 7 | 10 | 8 | 4 | 1.19 | 9.06E-02 |
| IPI00219446 | Phosphatidylethanolamine-binding protein 1 | 0 | 1 | 0 | 1 | 1 | 1.11 | 1.00E+00 |
| IPI00026271 | 40S ribosomal protein S14 | 2 | 1 | 2 | 1 | 1 | 0.93 | 8.00E-01 |
| IPI00305383 | Cytochrome b-c1 complex subunit 2| mitochondrial | 2 | 0 | 2 | 0 | 2 | 1.17 | 4.67E-01 |
| IPI00008380|IPI00815784 | Serine/threonine-protein phosphatase 2A catalytic subunit alpha isoform | 2 | 0 | 0 | 2 | 2 | 1.17 | 4.67E-01 |
| IPI00021266 | 60S ribosomal protein L23a | 0 | 1 | 1 | 1 | 0 | 1.11 | 5.00E-01 |
| IPI00790334|IPI00939602|IPI00940894|IPI01012998|IPI01019102 | DNA polymerase | 0 | 1 | 1 | 0 | 0 | 0.89 | 1.00E+00 |
| IPI00140420|IPI00910438|IPI01009513 | Staphylococcal nuclease domain-containing protein 1 | 1 | 0 | 0 | 1 | 1 | 1.11 | 1.00E+00 |
| IPI00029468|IPI01013091|IPI01013552 | Alpha-centractin | 0 | 2 | 0 | 2 | 0 | 0.83 | 1.00E+00 |
| IPI00930688 | Tubulin alpha-1B chain | 13 | 14 | 14 | 14 | 13 | 1.01 | 5.00E-01 |
| IPI00418262 | Fructose-bisphosphate aldolase | 3 | 2 | 3 | 2 | 3 | 1.05 | 6.08E-01 |
| IPI00219796 | Troponin C| skeletal muscle | 3 | 2 | 3 | 2 | 2 | 0.95 | 7.38E-01 |
| IPI00219953|IPI00514049|IPI00514929|IPI00908752 | UMP-CMP kinase isoform a | 0 | 2 | 0 | 2 | 0 | 0.83 | 1.00E+00 |
| IPI00009901 | Nuclear transport factor 2 | 1 | 0 | 0 | 1 | 0 | 0.89 | 1.00E+00 |
| IPI00216008|IPI00289800|IPI00853547 | Isoform Long of Glucose-6-phosphate 1-dehydrogenase | 0 | 1 | 0 | 1 | 0 | 0.89 | 1.00E+00 |
| IPI00908647 | cDNA FLJ59942| highly similar to Prostaglandin E synthase 3 | 0 | 2 | 0 | 0 | 2 | 0.83 | 1.00E+00 |
| IPI00334713 | Isoform 3 of Heterogeneous nuclear ribonucleoprotein A/B | 0 | 2 | 0 | 2 | 0 | 0.83 | 1.00E+00 |
| IPI00555956 | Proteasome subunit beta type-4 | 1 | 0 | 0 | 1 | 0 | 0.89 | 1.00E+00 |
| IPI00328550|IPI00966406 | Thrombospondin-4 | 1 | 2 | 1 | 2 | 2 | 1.07 | 8.00E-01 |
| IPI00985353 | Uncharacterized protein | 0 | 1 | 1 | 0 | 0 | 0.89 | 1.00E+00 |
| IPI00010214 | Protein S100-A14 | 0 | 2 | 0 | 0 | 2 | 0.83 | 1.00E+00 |
| IPI00297982 | Eukaryotic translation initiation factor 2 subunit 3 | 2 | 0 | 0 | 2 | 2 | 1.17 | 4.67E-01 |
| IPI00008530|IPI00556485|IPI01022174 | 60S acidic ribosomal protein P0 | 2 | 4 | 2 | 2 | 4 | 0.92 | 5.48E-01 |
| IPI00021785 | Cytochrome c oxidase subunit 5B| mitochondrial | 1 | 0 | 1 | 0 | 0 | 0.89 | 1.00E+00 |
| IPI00303200|IPI00922546 | Tropomodulin-4 | 0 | 1 | 0 | 0 | 1 | 0.89 | 1.00E+00 |
| IPI00412579 | 60S ribosomal protein L10a | 1 | 0 | 0 | 0 | 1 | 0.89 | 1.00E+00 |
| IPI00221106|IPI00477803|IPI00978402 | Splicing factor 3B subunit 2 | 1 | 0 | 0 | 1 | 0 | 0.89 | 1.00E+00 |
| IPI00032875|IPI00455179|IPI00965641 | Electron transfer flavoprotein-ubiquinone oxidoreductase| mitochondrial | 1 | 0 | 1 | 0 | 0 | 0.89 | 1.00E+00 |
| IPI00032406 | DnaJ homolog subfamily A member 2 | 1 | 0 | 1 | 0 | 0 | 0.89 | 1.00E+00 |
| IPI00022442 | Acyl carrier protein| mitochondrial | 0 | 2 | 2 | 2 | 0 | 1.17 | 1.67E-01 |
| IPI00019755|IPI00642936 | Glutathione S-transferase omega-1 | 1 | 0 | 1 | 0 | 1 | 1.11 | 1.00E+00 |
| IPI00010896 | Chloride intracellular channel protein 1 | 1 | 3 | 2 | 2 | 1 | 0.89 | 5.00E-01 |
| IPI00419585 | Peptidyl-prolyl cis-trans isomerase A | 8 | 4 | 8 | 7 | 7 | 1.19 | 6.42E-01 |
| IPI00217232|IPI00464979|IPI01013225 | Isoform 2 of Succinyl-CoA ligase [ADP-forming] subunit beta| mitochondrial | 3 | 0 | 2 | 0 | 2 | 0.93 | 4.29E-01 |
| IPI00009551|IPI00967845 | Isoform 1 of Myotilin | 2 | 0 | 1 | 0 | 1 | 0.83 | 1.00E+00 |
| IPI00102864|IPI00917193 | Hexokinase-2 | 0 | 5 | 0 | 2 | 2 | 0.67 | 1.00E+00 |
| IPI00744692 | Transaldolase | 2 | 4 | 3 | 3 | 2 | 0.92 | 5.00E-01 |
| IPI00027487 | Creatine kinase M-type | 10 | 11 | 12 | 7 | 10 | 0.93 | 5.24E-01 |
| IPI00027252 | Prohibitin-2 | 3 | 4 | 1 | 5 | 3 | 0.89 | 5.94E-01 |
| IPI00645078 | Ubiquitin-like modifier-activating enzyme 1 | 3 | 2 | 2 | 6 | 1 | 1.14 | 3.64E-01 |
| IPI00291328|IPI00646556 | NADH dehydrogenase [ubiquinone] flavoprotein 2| mitochondrial | 5 | 4 | 5 | 2 | 6 | 0.97 | 2.78E-01 |
| IPI00299145 | Keratin| type II cytoskeletal 6C | 14 | 15 | 10 | 14 | 16 | 0.92 | 2.82E-01 |
| IPI00017334 | Prohibitin | 2 | 3 | 0 | 4 | 3 | 0.95 | 4.17E-01 |
| IPI00013933 | Isoform DPI of Desmoplakin | 23 | 18 | 11 | 27 | 23 | 0.99 | 1.53E-01 |
| IPI00216049|IPI00216746 | Isoform 1 of Heterogeneous nuclear ribonucleoprotein K | 10 | 8 | 9 | 10 | 7 | 0.97 | 4.85E-01 |
| IPI00011253 | 40S ribosomal protein S3 | 5 | 3 | 3 | 7 | 2 | 1.00 | 4.37E-01 |
| IPI00246058|IPI00938079 | Programmed cell death 6-interacting protein | 2 | 1 | 0 | 2 | 3 | 1.07 | 2.86E-01 |
| IPI00479217|IPI00644224|IPI00883857 | Isoform Short of Heterogeneous nuclear ribonucleoprotein U | 2 | 3 | 3 | 5 | 1 | 1.14 | 1.97E-01 |
| IPI00411706|IPI00641040 | S-formylglutathione hydrolase | 2 | 1 | 1 | 3 | 0 | 0.93 | 5.00E-01 |
| IPI00221088 | 40S ribosomal protein S9 | 3 | 2 | 0 | 2 | 4 | 0.86 | 1.19E-01 |
| IPI00419258 | High mobility group protein B1 | 2 | 3 | 4 | 3 | 1 | 1.05 | 2.62E-01 |
| IPI00013808 | Alpha-actinin-4 | 17 | 16 | 10 | 22 | 16 | 0.97 | 4.40E-01 |
| IPI00037283|IPI00146935|IPI00235412|IPI00473085|IPI00555883|IPI00871742|IPI00942042|IPI01010281|IPI01021702|IPI01022698 | Isoform 5 of Dynamin-1-like protein | 1 | 2 | 0 | 2 | 3 | 1.07 | 5.00E-01 |
| IPI00003881|IPI01010852 | Heterogeneous nuclear ribonucleoprotein F | 3 | 2 | 3 | 4 | 1 | 1.05 | 3.48E-01 |
| IPI00016610 | Poly(rC)-binding protein 1 | 2 | 5 | 3 | 2 | 4 | 0.89 | 6.52E-01 |
| IPI00300502 | Myozenin-1 | 1 | 4 | 2 | 3 | 2 | 0.95 | 5.00E-01 |
| IPI00000874 | Peroxiredoxin-1 | 3 | 4 | 4 | 4 | 1 | 0.89 | 2.47E-01 |
| IPI00604590|IPI00795292 | Nucleoside diphosphate kinase | 4 | 4 | 4 | 6 | 3 | 1.07 | 4.19E-01 |
| IPI00456887|IPI00929107 | Heterogeneous nuclear ribonucleoprotein U-like protein 2 | 0 | 1 | 0 | 2 | 0 | 1.11 | 1.00E+00 |
| IPI00012772 | 60S ribosomal protein L8 | 1 | 3 | 1 | 2 | 3 | 1.00 | 7.14E-01 |
| IPI00413324|IPI00977661 | 60S ribosomal protein L17 | 0 | 3 | 2 | 2 | 2 | 1.20 | 4.00E-01 |
| IPI00026199 | Glutathione peroxidase 3 | 1 | 0 | 2 | 0 | 0 | 1.11 | 1.00E+00 |
| IPI00328113 | Fibrillin-1 | 1 | 0 | 0 | 0 | 2 | 1.11 | 3.33E-01 |
| IPI00299573 | 60S ribosomal protein L7a | 2 | 1 | 4 | 1 | 1 | 1.20 | 6.43E-01 |
| IPI00024067|IPI00455383 | Isoform 1 of Clathrin heavy chain 1 | 8 | 5 | 4 | 10 | 6 | 1.02 | 5.70E-01 |
| IPI00013068 | Eukaryotic translation initiation factor 3 subunit E | 1 | 0 | 0 | 2 | 0 | 1.11 | 1.00E+00 |
| IPI00218493|IPI00873466 | Hypoxanthine-guanine phosphoribosyltransferase | 0 | 2 | 2 | 1 | 0 | 1.00 | 3.33E-01 |
| IPI00215780 | 40S ribosomal protein S19 | 2 | 3 | 1 | 3 | 3 | 0.95 | 5.95E-01 |
| IPI00218918 | Annexin A1 | 2 | 2 | 3 | 2 | 1 | 1.00 | 5.00E-01 |
| IPI00219219 | Galectin-1 | 2 | 1 | 0 | 2 | 2 | 0.93 | 4.00E-01 |
| IPI00022228|IPI00894287 | Vigilin | 0 | 1 | 0 | 0 | 2 | 1.11 | 1.00E+00 |
| IPI00169383 | Phosphoglycerate kinase 1 | 10 | 11 | 11 | 11 | 9 | 0.99 | 4.37E-01 |
| IPI00300725|IPI00909059 | Keratin| type II cytoskeletal 6A | 14 | 17 | 12 | 17 | 17 | 0.99 | 4.86E-01 |
| IPI00216236|IPI00657955 | Troponin I| fast skeletal muscle | 2 | 2 | 3 | 2 | 1 | 1.00 | 5.00E-01 |
| IPI00026314|IPI00646773|IPI00647556|IPI00796316|IPI01009415|IPI01015050 | Isoform 1 of Gelsolin | 2 | 4 | 4 | 3 | 2 | 1.00 | 3.92E-01 |
| IPI00410693 | SERPINE1 mRNA binding protein 1| isoform CRA_d | 3 | 2 | 2 | 5 | 2 | 1.14 | 5.76E-01 |
| IPI00295857|IPI00646493 | Isoform 1 of Coatomer subunit alpha | 1 | 0 | 0 | 2 | 0 | 1.11 | 1.00E+00 |
| IPI00554737|IPI01014074 | Serine/threonine-protein phosphatase 2A 65 kDa regulatory subunit A alpha isoform | 6 | 3 | 4 | 5 | 4 | 0.97 | 6.05E-01 |
| IPI00292695 | Long-chain specific acyl-CoA dehydrogenase| mitochondrial | 1 | 0 | 2 | 0 | 0 | 1.11 | 1.00E+00 |
| IPI00003918 | 60S ribosomal protein L4 | 0 | 3 | 0 | 3 | 2 | 1.07 | 1.00E+00 |
| IPI00012011|IPI00978796|IPI00984795 | Cofilin-1 | 3 | 2 | 2 | 4 | 2 | 1.05 | 6.52E-01 |
| IPI00465256 | GTP:AMP phosphotransferase| mitochondrial | 1 | 0 | 2 | 0 | 0 | 1.11 | 1.00E+00 |
| IPI00216587 | 40S ribosomal protein S8 | 0 | 1 | 0 | 0 | 2 | 1.11 | 1.00E+00 |
| IPI01019113|IPI01020686|IPI01022164 | Tubulin beta chain | 19 | 16 | 18 | 17 | 17 | 0.99 | 5.49E-01 |
| IPI00021439|IPI00021440 | Actin| cytoplasmic 1 | 20 | 19 | 19 | 21 | 19 | 1.01 | 5.46E-01 |
| IPI00026185 | Isoform 1 of F-actin-capping protein subunit beta | 3 | 3 | 4 | 2 | 3 | 1.00 | 6.89E-01 |
| IPI00099550 | Isoform 1 of Ubiquilin-1 | 2 | 0 | 0 | 3 | 0 | 1.00 | 1.00E+00 |
| IPI00016786|IPI00909484 | Isoform 2 of Cell division control protein 42 homolog | 0 | 2 | 2 | 0 | 1 | 1.00 | 1.00E+00 |
| IPI00746165|IPI00939854 | Isoform 1 of WD repeat-containing protein 1 | 0 | 1 | 0 | 2 | 0 | 1.11 | 1.00E+00 |
| IPI00010720|IPI00940257|IPI00964079|IPI00964855|IPI00965269|IPI00965722 | T-complex protein 1 subunit epsilon | 2 | 0 | 2 | 1 | 0 | 1.00 | 1.00E+00 |
| IPI00302927|IPI00921414|IPI01015038 | T-complex protein 1 subunit delta | 0 | 2 | 0 | 1 | 2 | 1.00 | 1.00E+00 |
| IPI00797126 | Uncharacterized protein | 2 | 2 | 2 | 2 | 2 | 1.00 | 7.57E-01 |
| IPI00410714 | Hemoglobin subunit alpha | 2 | 2 | 2 | 2 | 2 | 1.00 | 7.57E-01 |
| IPI00298547 | Protein DJ-1 | 2 | 2 | 2 | 2 | 2 | 1.00 | 7.57E-01 |
| IPI00473011|IPI00654755|IPI00657660|IPI00796636|IPI00829896|IPI01018020 | Hemoglobin subunit delta | 2 | 2 | 2 | 2 | 2 | 1.00 | 7.57E-01 |

**Table D**

| **Protein accession numbers** | **Protein name** | **Control1** | **Control2** | **Tumor1** | **Tumor2** | **Tumor3** | **Fold Change** | **Fisher** |
| --- | --- | --- | --- | --- | --- | --- | --- | --- |
| IPI00756257 | Isoform 1 of Titin | 150 | 166 | 69 | 33 | 57 | 0.34 | 5.66E-28 |
| IPI00553454 | myosin heavy chain IIa | 86 | 93 | 0 | 61 | 66 | 0.48 | 2.58E-25 |
| IPI00465786 | Talin-1 | 4 | 4 | 33 | 42 | 38 | 7.73 | 2.09E-14 |
| IPI00720238 | Uncharacterized protein | 47 | 46 | 24 | 10 | 15 | 0.36 | 3.40E-08 |
| IPI00379424 | Uncharacterized protein | 32 | 30 | 14 | 6 | 0 | 0.24 | 1.49E-06 |
| IPI00468696 | Keratin| type I cytoskeletal 42 | 0 | 0 | 8 | 12 | 9 | 10.67 | 7.94E-06 |
| IPI00311654 | Sarcoplasmic/endoplasmic reticulum calcium ATPase 1 | 25 | 26 | 7 | 11 | 11 | 0.40 | 6.08E-05 |
| IPI00127841 | ADP/ATP translocase 2 | 11 | 6 | 0 | 0 | 0 | 0.11 | 8.08E-05 |
| IPI00308162 | Calcium-binding mitochondrial carrier protein Aralar1 | 12 | 9 | 1 | 1 | 2 | 0.20 | 1.15E-04 |
| IPI00331546 | Heat shock-related 70 kDa protein 2 | 0 | 0 | 8 | 7 | 0 | 6.00 | 1.55E-04 |
| IPI00131138|IPI00875567 | Isoform 1 of Filamin-A | 6 | 5 | 13 | 23 | 22 | 3.13 | 6.39E-04 |
| IPI00622235 | Transitional endoplasmic reticulum ATPase | 14 | 11 | 24 | 31 | 29 | 2.15 | 8.76E-04 |
| IPI00346834 | Keratin| type II cytoskeletal 2 oral | 9 | 10 | 0 | 5 | 6 | 0.44 | 1.53E-03 |
| IPI00110588 | Moesin | 1 | 1 | 6 | 11 | 7 | 4.50 | 1.69E-03 |
| IPI00227392 | 14-3-3 protein eta | 0 | 0 | 6 | 5 | 2 | 5.33 | 2.16E-03 |
| IPI00130460|IPI00928416 | NADH dehydrogenase [ubiquinone] flavoprotein 1| mitochondrial | 11 | 9 | 4 | 1 | 4 | 0.36 | 2.71E-03 |
| IPI00380896 | Myosin-1 | 104 | 112 | 87 | 74 | 84 | 0.76 | 2.96E-03 |
| IPI00420569|IPI00762871 | Sodium/potassium-transporting ATPase subunit alpha-2 | 7 | 4 | 0 | 0 | 4 | 0.36 | 3.03E-03 |
| IPI00663627 | Filamin-B | 0 | 0 | 4 | 7 | 6 | 6.67 | 3.03E-03 |
| IPI00421271 | plectin isoform 1f | 12 | 8 | 16 | 28 | 22 | 2.09 | 4.50E-03 |
| IPI00938530 | myosin-11 isoform 1 | 8 | 8 | 0 | 5 | 10 | 0.67 | 6.32E-03 |
| IPI00553777|IPI00817004 | Putative uncharacterized protein | 1 | 1 | 5 | 9 | 2 | 3.17 | 7.62E-03 |
| IPI00676959 | Keratin| type I cuticular Ha6 | 4 | 5 | 0 | 0 | 0 | 0.18 | 7.94E-03 |
| IPI00353563 | Fascin | 0 | 0 | 3 | 7 | 2 | 5.00 | 8.33E-03 |
| IPI00228548 | Beta-enolase | 19 | 17 | 10 | 7 | 14 | 0.60 | 8.71E-03 |
| IPI00115607 | Trifunctional enzyme subunit beta| mitochondrial | 7 | 6 | 1 | 1 | 0 | 0.22 | 8.86E-03 |
| IPI00123181 | Myosin-9 | 14 | 14 | 20 | 32 | 30 | 1.89 | 1.03E-02 |
| IPI00122928 | Tubulin beta-6 chain | 0 | 9 | 8 | 10 | 9 | 1.82 | 1.10E-02 |
| IPI00115564 | ADP/ATP translocase 1 | 8 | 8 | 2 | 2 | 4 | 0.41 | 1.15E-02 |
| IPI00404837 | Myosin-4 | 102 | 110 | 93 | 75 | 82 | 0.79 | 1.40E-02 |
| IPI00115528|IPI00776023 | Plastin-3 | 0 | 0 | 2 | 10 | 4 | 6.33 | 1.52E-02 |
| IPI00317794 | Nucleolin | 0 | 0 | 3 | 5 | 2 | 4.33 | 1.79E-02 |
| IPI00130127|IPI00856819 | Glycogen [starch] synthase| muscle | 5 | 3 | 0 | 0 | 1 | 0.27 | 1.79E-02 |
| IPI00323179 | Rab GDP dissociation inhibitor alpha | 0 | 0 | 3 | 5 | 4 | 5.00 | 1.79E-02 |
| IPI00315893 | Alpha-amylase 1 | 3 | 5 | 0 | 0 | 0 | 0.20 | 1.79E-02 |
| IPI00387557 | alpha-actinin-2 | 40 | 39 | 27 | 26 | 30 | 0.71 | 1.81E-02 |
| IPI00225275 | Glycogen phosphorylase| muscle form | 18 | 17 | 13 | 6 | 11 | 0.59 | 1.88E-02 |
| IPI00330862 | Ezrin | 0 | 0 | 2 | 8 | 7 | 6.67 | 2.22E-02 |
| IPI00468665 | Uncharacterized protein | 32 | 38 | 28 | 19 | 26 | 0.70 | 2.28E-02 |
| IPI00109169|IPI00988842 | Isocitrate dehydrogenase [NAD] subunit gamma 1| mitochondrial | 8 | 8 | 5 | 1 | 3 | 0.44 | 2.47E-02 |
| IPI00118892 | Plastin-2 | 0 | 0 | 4 | 3 | 0 | 3.33 | 2.86E-02 |
| IPI00625729 | Keratin| type II cytoskeletal 1 | 0 | 0 | 4 | 3 | 2 | 4.00 | 2.86E-02 |
| IPI00114945 | Septin-2 | 0 | 0 | 3 | 4 | 2 | 4.00 | 2.86E-02 |
| IPI00221613|IPI00221614 | ADP-ribosylation factor 1 | 3 | 4 | 0 | 0 | 0 | 0.22 | 2.86E-02 |
| IPI00230754 | Mitochondrial 2-oxoglutarate/malate carrier protein | 4 | 3 | 0 | 0 | 0 | 0.22 | 2.86E-02 |
| IPI00112963 | Catenin alpha-1 | 0 | 0 | 2 | 6 | 3 | 4.67 | 3.57E-02 |
| IPI00331541 | 6-phosphofructokinase| muscle type | 9 | 10 | 5 | 3 | 5 | 0.51 | 3.77E-02 |
| IPI00118676 | Eukaryotic initiation factor 4A-I | 5 | 2 | 7 | 10 | 9 | 2.15 | 4.47E-02 |
| IPI00122528|IPI00991018 | Transforming growth factor-beta-induced protein ig-h3 | 0 | 0 | 2 | 5 | 2 | 4.00 | 4.76E-02 |
| IPI00122312|IPI00990997 | Uncharacterized protein | 0 | 0 | 3 | 3 | 0 | 3.00 | 5.00E-02 |
| IPI00467841|IPI00761696 | Putative uncharacterized protein | 0 | 0 | 3 | 3 | 4 | 4.33 | 5.00E-02 |
| IPI00131459 | Nucleoside diphosphate kinase A | 3 | 3 | 0 | 0 | 0 | 0.25 | 5.00E-02 |
| IPI00230264 | Histone H2A.x | 5 | 6 | 0 | 5 | 4 | 0.62 | 5.77E-02 |
| IPI00115627 | Actin-related protein 3 | 1 | 2 | 4 | 7 | 7 | 2.80 | 6.29E-02 |
| IPI00880839 | stress-70 protein| mitochondrial | 11 | 13 | 5 | 8 | 7 | 0.59 | 6.41E-02 |
| IPI00622847|IPI00828488|IPI01023279 | Isoform 2 of Heterogeneous nuclear ribonucleoproteins A2/B1 | 4 | 5 | 7 | 13 | 6 | 1.76 | 6.51E-02 |
| IPI00420656 | Isoform 2 of Tenascin | 0 | 0 | 2 | 4 | 2 | 3.67 | 6.67E-02 |
| IPI00121149|IPI00755120 | Isoform 3 of Ribosome-binding protein 1 | 0 | 0 | 2 | 4 | 2 | 3.67 | 6.67E-02 |
| IPI00111560|IPI00347814|IPI00410883|IPI00755843|IPI00988216 | Isoform 1 of Protein SET | 0 | 0 | 2 | 4 | 3 | 4.00 | 6.67E-02 |
| IPI00311369 | Ubiquitin carboxyl-terminal hydrolase isozyme L3 | 0 | 0 | 2 | 4 | 3 | 4.00 | 6.67E-02 |
| IPI00409462 | Spliceosome RNA helicase Ddx39b | 0 | 0 | 2 | 4 | 3 | 4.00 | 6.67E-02 |
| IPI00153660 | Dihydrolipoyllysine-residue acetyltransferase component of pyruvate dehydrogenase complex| mitochondrial | 4 | 4 | 2 | 0 | 2 | 0.47 | 7.14E-02 |
| IPI00129430 | Splicing factor| proline- and glutamine-rich | 1 | 0 | 4 | 3 | 1 | 2.44 | 7.14E-02 |
| IPI00662244 | glycogen debranching enzyme | 4 | 3 | 1 | 0 | 0 | 0.30 | 7.14E-02 |
| IPI00117312 | Aspartate aminotransferase| mitochondrial | 9 | 8 | 5 | 3 | 5 | 0.56 | 7.49E-02 |
| IPI00656173 | Isoform 4 of LIM domain-binding protein 3 | 8 | 9 | 5 | 3 | 2 | 0.46 | 7.49E-02 |
| IPI00122565 | Isoform 1 of Rab GDP dissociation inhibitor beta | 2 | 1 | 5 | 5 | 5 | 2.40 | 7.75E-02 |
| IPI00225378|IPI00876179 | keratin| type I cytoskeletal 15 | 11 | 10 | 6 | 5 | 6 | 0.58 | 7.78E-02 |
| IPI00408378|IPI00656269|IPI00853924 | Isoform 1 of 14-3-3 protein theta | 2 | 4 | 7 | 7 | 7 | 2.00 | 7.99E-02 |
| IPI00467447 | Ras GTPase-activating-like protein IQGAP1 | 0 | 1 | 2 | 6 | 5 | 3.56 | 8.33E-02 |
| IPI00124287 | Polyadenylate-binding protein 1 | 2 | 0 | 5 | 3 | 4 | 2.50 | 8.33E-02 |
| IPI00230440 | Adenosylhomocysteinase | 1 | 1 | 3 | 6 | 2 | 2.33 | 8.79E-02 |
| IPI00755181|IPI00798492|IPI00828744|IPI01008564 | keratin| type I cytoskeletal 10 | 5 | 4 | 0 | 4 | 3 | 0.61 | 9.79E-02 |
| IPI00915477 | Uncharacterized protein | 3 | 2 | 0 | 0 | 1 | 0.38 | 1.00E-01 |
| IPI00331436|IPI00828469 | Isoform 1 of Cytosol aminopeptidase | 2 | 3 | 0 | 0 | 1 | 0.38 | 1.00E-01 |
| IPI00467004|IPI01016187 | signal transducer and activator of transcription 1 isoform 1 | 0 | 0 | 3 | 2 | 1 | 3.00 | 1.00E-01 |
| IPI00785343|IPI00986082 | Histone H3.3 | 0 | 0 | 3 | 2 | 1 | 3.00 | 1.00E-01 |
| IPI00323819|IPI00987717 | 40S ribosomal protein S20 | 0 | 0 | 2 | 3 | 2 | 3.33 | 1.00E-01 |
| IPI00122562|IPI00131407 | Proteasome subunit alpha type | 0 | 0 | 3 | 2 | 3 | 3.67 | 1.00E-01 |
| IPI00136134 | Isoform 1 of Protein NDRG2 | 2 | 3 | 0 | 0 | 0 | 0.29 | 1.00E-01 |
| IPI00461964 | Methylmalonate-semialdehyde dehydrogenase [acylating]| mitochondrial | 2 | 3 | 0 | 0 | 0 | 0.29 | 1.00E-01 |
| IPI00626655 | Isoform 1 of Myomesin-1 | 5 | 6 | 2 | 2 | 2 | 0.46 | 1.00E-01 |
| IPI00122293 | Prolargin | 4 | 4 | 3 | 0 | 2 | 0.53 | 1.06E-01 |
| IPI00224456 | Isoform 1 of Sarcalumenin | 9 | 7 | 5 | 3 | 6 | 0.63 | 1.07E-01 |
| IPI00123190 | Isoform 1 of Adenylosuccinate synthetase isozyme 1 | 5 | 2 | 1 | 0 | 1 | 0.37 | 1.07E-01 |
| IPI00420312 | Keratin| type II cytoskeletal 4 | 11 | 10 | 5 | 7 | 6 | 0.61 | 1.07E-01 |
| IPI00323357 | Heat shock cognate 71 kDa protein | 18 | 16 | 24 | 23 | 23 | 1.35 | 1.10E-01 |
| IPI00230394 | Lamin-B1 | 0 | 1 | 3 | 3 | 1 | 2.22 | 1.14E-01 |
| IPI00128023 | NADH dehydrogenase [ubiquinone] iron-sulfur protein 2| mitochondrial | 3 | 3 | 0 | 1 | 1 | 0.42 | 1.14E-01 |
| IPI00226430 | 3-ketoacyl-CoA thiolase| mitochondrial | 4 | 3 | 2 | 0 | 3 | 0.59 | 1.19E-01 |
| IPI00128296 | Creatine kinase U-type| mitochondrial | 3 | 4 | 0 | 2 | 0 | 0.37 | 1.19E-01 |
| IPI00620362|IPI00653643|IPI00985815 | Heterogeneous nuclear ribonucleoprotein L | 1 | 2 | 4 | 5 | 3 | 2.00 | 1.21E-01 |
| IPI00121309 | NADH dehydrogenase [ubiquinone] iron-sulfur protein 3| mitochondrial | 5 | 4 | 2 | 1 | 2 | 0.48 | 1.21E-01 |
| IPI00130102 | Desmin | 21 | 18 | 17 | 11 | 15 | 0.75 | 1.23E-01 |
| IPI00230044 | Isoform 2 of Tropomyosin alpha-3 chain | 8 | 7 | 12 | 12 | 9 | 1.41 | 1.30E-01 |
| IPI00129526 | Endoplasmin | 5 | 5 | 8 | 10 | 8 | 1.61 | 1.33E-01 |
| IPI00406117|IPI00406118 | Isoform 1 of Heterogeneous nuclear ribonucleoprotein Q | 0 | 2 | 2 | 6 | 5 | 2.67 | 1.33E-01 |
| IPI00466919 | 6-phosphogluconate dehydrogenase| decarboxylating | 1 | 0 | 2 | 4 | 2 | 2.44 | 1.43E-01 |
| IPI00137409 | Transketolase | 1 | 0 | 2 | 4 | 4 | 2.89 | 1.43E-01 |
| IPI00396774 | myosin binding protein C| slow type | 10 | 14 | 11 | 5 | 8 | 0.69 | 1.44E-01 |
| IPI00459725 | Isoform 1 of Isocitrate dehydrogenase [NAD] subunit alpha| mitochondrial | 10 | 10 | 9 | 4 | 7 | 0.70 | 1.53E-01 |
| IPI00133206|IPI00405114 | 26S protease regulatory subunit 6A | 3 | 2 | 5 | 6 | 4 | 1.71 | 1.57E-01 |
| IPI00554894 | Annexin A6 | 6 | 5 | 3 | 2 | 3 | 0.56 | 1.57E-01 |
| IPI00127707|IPI00221796|IPI00221799 | Isoform 1 of Poly(rC)-binding protein 2 | 4 | 0 | 3 | 5 | 4 | 1.67 | 1.59E-01 |
| IPI00119667 | Elongation factor 1-alpha 2 | 12 | 11 | 7 | 8 | 9 | 0.72 | 1.65E-01 |
| IPI00138274 | Alpha-crystallin B chain | 3 | 4 | 1 | 1 | 2 | 0.52 | 1.67E-01 |
| IPI00133916|IPI00224729 | Heterogeneous nuclear ribonucleoprotein H | 0 | 3 | 3 | 4 | 3 | 1.73 | 1.67E-01 |
| IPI00135686 | Peptidyl-prolyl cis-trans isomerase B | 2 | 0 | 2 | 5 | 4 | 2.33 | 1.67E-01 |
| IPI00223377|IPI00223378|IPI00223593 | Isoform 10 of Myelin basic protein | 0 | 0 | 2 | 2 | 1 | 2.67 | 1.67E-01 |
| IPI00322312 | Rho GDP-dissociation inhibitor 1 | 0 | 0 | 2 | 2 | 1 | 2.67 | 1.67E-01 |
| IPI00128818|IPI00775838 | Putative pre-mRNA-splicing factor ATP-dependent RNA helicase DHX15 | 0 | 0 | 2 | 2 | 1 | 2.67 | 1.67E-01 |
| IPI00113240 | Metallothionein-2 | 0 | 0 | 2 | 2 | 2 | 3.00 | 1.67E-01 |
| IPI00119202|IPI00988820 | Protein S100-A11 | 0 | 0 | 2 | 2 | 2 | 3.00 | 1.67E-01 |
| IPI00224505 | 60S ribosomal protein L13 | 0 | 0 | 2 | 2 | 3 | 3.33 | 1.67E-01 |
| IPI00622912 | ryanodine receptor 1| skeletal muscle | 2 | 2 | 0 | 0 | 0 | 0.33 | 1.67E-01 |
| IPI00776084 | ATP synthase gamma chain | 2 | 2 | 0 | 0 | 0 | 0.33 | 1.67E-01 |
| IPI00132217|IPI00857192 | Mitochondrial fission 1 protein | 2 | 2 | 0 | 0 | 0 | 0.33 | 1.67E-01 |
| IPI00132653|IPI00858156 | Succinyl-CoA:3-ketoacid-coenzyme A transferase 1| mitochondrial | 2 | 2 | 0 | 0 | 0 | 0.33 | 1.67E-01 |
| IPI00116498 | 14-3-3 protein zeta/delta | 7 | 10 | 12 | 13 | 13 | 1.44 | 1.69E-01 |
| IPI00407339 | Histone H4 | 5 | 5 | 9 | 8 | 8 | 1.56 | 1.70E-01 |
| IPI00221402|IPI00856379 | Fructose-bisphosphate aldolase A | 16 | 17 | 13 | 11 | 11 | 0.72 | 1.75E-01 |
| IPI00459493 | Isoform 1 of T-complex protein 1 subunit alpha | 1 | 2 | 4 | 4 | 2 | 1.73 | 1.75E-01 |
| IPI00136056 | Isoform 1 of Keratin| type I cytoskeletal 13 | 12 | 13 | 9 | 8 | 11 | 0.77 | 1.77E-01 |
| IPI00330958|IPI00336873 | Isoform 1 of Heterogeneous nuclear ribonucleoprotein D0 | 0 | 2 | 3 | 3 | 2 | 1.83 | 1.79E-01 |
| IPI00321734 | Lactoylglutathione lyase | 2 | 0 | 3 | 3 | 0 | 1.50 | 1.79E-01 |
| IPI00136883|IPI00475378|IPI00749991 | Putative uncharacterized protein | 0 | 2 | 3 | 3 | 5 | 2.33 | 1.79E-01 |
| IPI00230108 | Protein disulfide-isomerase A3 | 3 | 2 | 7 | 4 | 6 | 1.90 | 1.82E-01 |
| IPI00318614 | Isocitrate dehydrogenase [NADP]| mitochondrial | 7 | 10 | 5 | 5 | 5 | 0.63 | 1.82E-01 |
| IPI00116074 | Aconitate hydratase| mitochondrial | 19 | 22 | 19 | 13 | 14 | 0.76 | 1.92E-01 |
| IPI00225307|IPI00469317 | seryl-tRNA synthetase| cytoplasmic isoform 1 | 0 | 1 | 2 | 3 | 0 | 1.78 | 2.00E-01 |
| IPI00758006 | Putative uncharacterized protein | 0 | 1 | 2 | 3 | 1 | 2.00 | 2.00E-01 |
| IPI00469918 | Rps16 protein | 1 | 0 | 3 | 2 | 1 | 2.00 | 2.00E-01 |
| IPI00308706|IPI00881253|IPI00988023 | 60S ribosomal protein L5 | 1 | 0 | 2 | 3 | 5 | 2.89 | 2.00E-01 |
| IPI00127415|IPI00849626 | Nucleophosmin | 0 | 0 | 1 | 4 | 1 | 3.00 | 2.00E-01 |
| IPI00352163|IPI00652813|IPI00974712|IPI00975193 | Putative uncharacterized protein | 0 | 0 | 1 | 4 | 2 | 3.33 | 2.00E-01 |
| IPI00626237 | Isoform 1 of 2-oxoglutarate dehydrogenase| mitochondrial | 10 | 8 | 8 | 4 | 8 | 0.77 | 2.06E-01 |
| IPI00118286 | 14-3-3 protein sigma | 4 | 6 | 8 | 8 | 7 | 1.44 | 2.07E-01 |
| IPI00664670|IPI00753917 | Uncharacterized protein | 18 | 14 | 10 | 14 | 16 | 0.84 | 2.09E-01 |
| IPI00139795 | 60S acidic ribosomal protein P2 | 2 | 0 | 2 | 4 | 3 | 2.00 | 2.14E-01 |
| IPI00229510 | L-lactate dehydrogenase B chain | 8 | 4 | 3 | 3 | 4 | 0.62 | 2.20E-01 |
| IPI00177038 | Actin-related protein 2 | 3 | 0 | 5 | 2 | 3 | 1.73 | 2.22E-01 |
| IPI00308882 | NADH-ubiquinone oxidoreductase 75 kDa subunit| mitochondrial | 8 | 9 | 6 | 5 | 4 | 0.63 | 2.25E-01 |
| IPI00120076 | Creatine kinase S-type| mitochondrial | 12 | 14 | 9 | 10 | 9 | 0.74 | 2.31E-01 |
| IPI00118384 | 14-3-3 protein epsilon | 5 | 7 | 10 | 8 | 4 | 1.19 | 2.31E-01 |
| IPI00122549|IPI00230540 | Isoform Pl-VDAC1 of Voltage-dependent anion-selective channel protein 1 | 8 | 10 | 7 | 5 | 5 | 0.67 | 2.31E-01 |
| IPI00122547 | Voltage-dependent anion-selective channel protein 2 | 3 | 3 | 1 | 1 | 3 | 0.67 | 2.43E-01 |
| IPI00114733 | Serpin H1 | 1 | 1 | 3 | 3 | 3 | 2.00 | 2.43E-01 |
| IPI00222515 | 26S proteasome non-ATPase regulatory subunit 11 | 0 | 0 | 1 | 3 | 1 | 2.67 | 2.50E-01 |
| IPI00420807|IPI00974806 | Isoform 1 of Serine/arginine-rich splicing factor 1 | 0 | 0 | 1 | 3 | 2 | 3.00 | 2.50E-01 |
| IPI00318901|IPI00331411|IPI00648431 | Uncharacterized protein | 1 | 3 | 0 | 0 | 0 | 0.33 | 2.50E-01 |
| IPI00115823 | myomesin 2 | 3 | 1 | 0 | 0 | 0 | 0.33 | 2.50E-01 |
| IPI00515360 | basement membrane-specific heparan sulfate proteoglycan core protein | 9 | 4 | 3 | 4 | 1 | 0.49 | 2.51E-01 |
| IPI00553798 | AHNAK nucleoprotein isoform 1 | 12 | 12 | 14 | 17 | 18 | 1.33 | 2.57E-01 |
| IPI00317740 | Guanine nucleotide-binding protein subunit beta-2-like 1 | 6 | 6 | 2 | 6 | 5 | 0.76 | 2.60E-01 |
| IPI00227299|IPI00988539 | Vimentin | 14 | 12 | 18 | 15 | 17 | 1.26 | 2.61E-01 |
| IPI00402968 | EH domain-containing protein 2 | 4 | 3 | 2 | 1 | 2 | 0.59 | 2.62E-01 |
| IPI00400016 | Laminin subunit gamma-1 | 4 | 3 | 2 | 1 | 2 | 0.59 | 2.62E-01 |
| IPI00307837 | Elongation factor 1-alpha 1 | 13 | 12 | 8 | 11 | 10 | 0.79 | 2.64E-01 |
| IPI00330804 | Heat shock protein HSP 90-alpha | 7 | 7 | 8 | 12 | 10 | 1.38 | 2.70E-01 |
| IPI00554929 | Heat shock protein HSP 90-beta | 18 | 15 | 19 | 21 | 19 | 1.18 | 2.76E-01 |
| IPI00230707 | 14-3-3 protein gamma | 5 | 5 | 7 | 8 | 6 | 1.33 | 2.77E-01 |
| IPI00130280 | ATP synthase subunit alpha| mitochondrial | 21 | 24 | 22 | 16 | 18 | 0.84 | 2.78E-01 |
| IPI00319992 | 78 kDa glucose-regulated protein | 12 | 13 | 13 | 19 | 16 | 1.26 | 2.78E-01 |
| IPI00116591 | Short-chain specific acyl-CoA dehydrogenase| mitochondrial | 4 | 4 | 2 | 2 | 2 | 0.60 | 2.84E-01 |
| IPI00420726 | 40S ribosomal protein S9 | 3 | 2 | 0 | 2 | 4 | 0.86 | 2.86E-01 |
| IPI00119305 | Proliferation-associated protein 2G4 | 1 | 1 | 2 | 4 | 2 | 1.83 | 2.86E-01 |
| IPI00119203 | Very long-chain specific acyl-CoA dehydrogenase| mitochondrial | 3 | 2 | 2 | 0 | 0 | 0.48 | 2.86E-01 |
| IPI00874585 | Keratin| type I cytoskeletal 16 | 6 | 7 | 9 | 9 | 8 | 1.29 | 2.93E-01 |
| IPI00114593|IPI00480406|IPI00653007 | Actin| alpha cardiac muscle 1 | 24 | 23 | 19 | 21 | 20 | 0.86 | 2.95E-01 |
| IPI00123319 | Isoform 1 of Tropomyosin beta chain | 21 | 24 | 19 | 19 | 19 | 0.85 | 2.96E-01 |
| IPI00114375 | Dihydropyrimidinase-related protein 2 | 4 | 5 | 2 | 3 | 3 | 0.67 | 2.96E-01 |
| IPI00261467 | Adenosine deaminase | 4 | 5 | 2 | 3 | 2 | 0.61 | 2.96E-01 |
| IPI00116753 | Electron transfer flavoprotein subunit alpha| mitochondrial | 4 | 5 | 2 | 3 | 2 | 0.61 | 2.96E-01 |
| IPI00121440 | Electron transfer flavoprotein subunit beta | 4 | 5 | 3 | 2 | 2 | 0.61 | 2.96E-01 |
| IPI00457898 | Phosphoglycerate mutase 1 | 9 | 6 | 9 | 11 | 7 | 1.18 | 2.96E-01 |
| IPI00137736 | 40S ribosomal protein S28 | 0 | 1 | 2 | 2 | 2 | 2.00 | 3.00E-01 |
| IPI00320217 | T-complex protein 1 subunit beta | 0 | 1 | 2 | 2 | 3 | 2.22 | 3.00E-01 |
| IPI00453826|IPI00990783 | Matrin-3 | 0 | 1 | 2 | 2 | 3 | 2.22 | 3.00E-01 |
| IPI00109109 | Superoxide dismutase [Mn]| mitochondrial | 2 | 2 | 0 | 1 | 1 | 0.56 | 3.00E-01 |
| IPI00124771|IPI00850430 | Phosphate carrier protein| mitochondrial | 2 | 2 | 0 | 1 | 1 | 0.56 | 3.00E-01 |
| IPI00117475 | Protein-glutamine gamma-glutamyltransferase E | 2 | 2 | 1 | 0 | 0 | 0.44 | 3.00E-01 |
| IPI00828338 | Uncharacterized protein | 2 | 2 | 1 | 0 | 0 | 0.44 | 3.00E-01 |
| IPI00222767 | Pyruvate dehydrogenase protein X component| mitochondrial | 2 | 2 | 0 | 1 | 0 | 0.44 | 3.00E-01 |
| IPI00462072|IPI00987541 | Alpha-enolase | 11 | 11 | 9 | 8 | 10 | 0.83 | 3.14E-01 |
| IPI00124954 | Kinesin-1 heavy chain | 1 | 4 | 4 | 4 | 5 | 1.52 | 3.15E-01 |
| IPI00230351 | Succinate dehydrogenase [ubiquinone] flavoprotein subunit| mitochondrial | 5 | 6 | 3 | 4 | 6 | 0.82 | 3.19E-01 |
| IPI00274407 | Isoform 1 of Elongation factor Tu| mitochondrial | 6 | 5 | 4 | 3 | 4 | 0.72 | 3.19E-01 |
| IPI00129928|IPI00759940 | Isoform Mitochondrial of Fumarate hydratase| mitochondrial | 6 | 5 | 3 | 4 | 4 | 0.72 | 3.19E-01 |
| IPI00553419 | Desmoplakin | 23 | 18 | 11 | 27 | 23 | 0.99 | 3.23E-01 |
| IPI00169463 | Tubulin beta-2C chain | 21 | 21 | 18 | 18 | 16 | 0.83 | 3.25E-01 |
| IPI00111218 | Aldehyde dehydrogenase| mitochondrial | 6 | 4 | 3 | 3 | 1 | 0.56 | 3.29E-01 |
| IPI00110827 | Actin| alpha skeletal muscle | 24 | 23 | 19 | 22 | 21 | 0.88 | 3.31E-01 |
| IPI00830749|IPI00877197|IPI00986243|IPI00990928 | LOW QUALITY PROTEIN: collagen alpha-3(VI) chain | 0 | 1 | 4 | 1 | 3 | 2.44 | 3.33E-01 |
| IPI00341282 | ATP synthase subunit b| mitochondrial | 2 | 1 | 0 | 0 | 1 | 0.53 | 3.33E-01 |
| IPI00132728|IPI00845772 | Isoform 1 of Cytochrome c1| heme protein| mitochondrial | 2 | 1 | 0 | 0 | 1 | 0.53 | 3.33E-01 |
| IPI00312128 | Isoform 1 of Transcription intermediary factor 1-beta | 0 | 0 | 1 | 2 | 0 | 2.00 | 3.33E-01 |
| IPI00128760 | Ubiquitin-conjugating enzyme E2 L3 | 0 | 0 | 1 | 2 | 0 | 2.00 | 3.33E-01 |
| IPI00127358 | SH3 domain-binding glutamic acid-rich-like protein 3 | 0 | 0 | 2 | 1 | 0 | 2.00 | 3.33E-01 |
| IPI00116850 | tRNA-splicing ligase RtcB homolog | 0 | 0 | 1 | 2 | 0 | 2.00 | 3.33E-01 |
| IPI00113870 | Proliferating cell nuclear antigen | 0 | 0 | 1 | 2 | 0 | 2.00 | 3.33E-01 |
| IPI00474450 | Dystrophin | 2 | 1 | 0 | 0 | 0 | 0.40 | 3.33E-01 |
| IPI00223757|IPI00988228 | Aldose reductase | 1 | 2 | 0 | 0 | 0 | 0.40 | 3.33E-01 |
| IPI00869462|IPI00988161 | Calsequestrin-2 | 1 | 2 | 0 | 0 | 0 | 0.40 | 3.33E-01 |
| IPI00122349|IPI00406447|IPI00881485 | Dihydropyrimidinase-related protein 3 | 1 | 2 | 0 | 0 | 0 | 0.40 | 3.33E-01 |
| IPI00131845 | Proteasome subunit alpha type-6 | 0 | 0 | 1 | 2 | 1 | 2.33 | 3.33E-01 |
| IPI00880644 | Uncharacterized protein | 0 | 0 | 1 | 2 | 1 | 2.33 | 3.33E-01 |
| IPI00320016 | Isoform 1 of Non-POU domain-containing octamer-binding protein | 0 | 0 | 2 | 1 | 1 | 2.33 | 3.33E-01 |
| IPI00224626|IPI00874440|IPI00985894 | septin-7 | 0 | 0 | 1 | 2 | 1 | 2.33 | 3.33E-01 |
| IPI00308222|IPI00378015|IPI00458127 | Isoform 2 of Drebrin-like protein | 0 | 0 | 1 | 2 | 1 | 2.33 | 3.33E-01 |
| IPI00230682|IPI00760000 | Isoform Long of 14-3-3 protein beta/alpha | 5 | 5 | 7 | 7 | 7 | 1.33 | 3.42E-01 |
| IPI00555140 | Phosphoglucomutase-1 | 3 | 5 | 1 | 4 | 1 | 0.60 | 3.43E-01 |
| IPI00651782 | protein NipSnap homolog 2 | 3 | 3 | 1 | 2 | 3 | 0.75 | 3.57E-01 |
| IPI00465880 | 40S ribosomal protein S17 | 2 | 1 | 3 | 3 | 2 | 1.47 | 3.57E-01 |
| IPI00467833|IPI00988063 | triosephosphate isomerase | 8 | 10 | 8 | 6 | 8 | 0.83 | 3.61E-01 |
| IPI00317590|IPI00620156|IPI00990188|IPI01019245 | 40S ribosomal protein S18 | 1 | 1 | 2 | 3 | 1 | 1.50 | 3.71E-01 |
| IPI00986015 | protein disulfide-isomerase A6-like | 1 | 1 | 3 | 2 | 2 | 1.67 | 3.71E-01 |
| IPI00223092 | Trifunctional enzyme subunit alpha| mitochondrial | 2 | 3 | 1 | 1 | 1 | 0.57 | 3.71E-01 |
| IPI00131204 | Isoform 1 of UTP--glucose-1-phosphate uridylyltransferase | 5 | 4 | 4 | 2 | 2 | 0.67 | 3.78E-01 |
| IPI00319994|IPI00751369 | L-lactate dehydrogenase A chain | 5 | 4 | 2 | 4 | 2 | 0.67 | 3.78E-01 |
| IPI00229475 | Junction plakoglobin | 13 | 13 | 7 | 17 | 9 | 0.86 | 3.86E-01 |
| IPI00224549 | Myosin regulatory light chain 2| skeletal muscle isoform | 12 | 14 | 12 | 10 | 11 | 0.86 | 3.86E-01 |
| IPI00131368 | Keratin| type II cytoskeletal 6A | 16 | 19 | 13 | 18 | 18 | 0.94 | 3.91E-01 |
| IPI00420745|IPI00989734 | Proteasome subunit alpha type | 2 | 2 | 3 | 4 | 0 | 1.11 | 3.92E-01 |
| IPI00309207 | Isoform 1 of Platelet-activating factor acetylhydrolase IB subunit alpha | 2 | 2 | 3 | 4 | 0 | 1.11 | 3.92E-01 |
| IPI00113141 | Citrate synthase| mitochondrial | 4 | 3 | 2 | 2 | 2 | 0.67 | 3.92E-01 |
| IPI00128209|IPI00750256 | Isoform 1 of Adenylate kinase isoenzyme 1 | 4 | 3 | 2 | 2 | 1 | 0.59 | 3.92E-01 |
| IPI00133006|IPI00918079|IPI00918301|IPI00987889 | Acyl carrier protein| mitochondrial | 0 | 2 | 2 | 2 | 0 | 1.17 | 4.00E-01 |
| IPI00111981 | Isoform 1 of Obg-like ATPase 1 | 0 | 2 | 2 | 2 | 2 | 1.50 | 4.00E-01 |
| IPI00118447 | Transcriptional activator protein Pur-alpha | 2 | 2 | 2 | 0 | 2 | 0.78 | 4.00E-01 |
| IPI00114560|IPI00467910|IPI00989100 | Ras-related protein Rab-1A | 2 | 2 | 0 | 2 | 2 | 0.78 | 4.00E-01 |
| IPI00128522 | Isoform A of Heat shock protein beta-1 | 0 | 1 | 1 | 3 | 2 | 2.00 | 4.00E-01 |
| IPI00420363|IPI01023287 | Probable ATP-dependent RNA helicase DDX5 | 0 | 1 | 1 | 3 | 3 | 2.22 | 4.00E-01 |
| IPI00136703 | Creatine kinase B-type | 2 | 2 | 0 | 2 | 1 | 0.67 | 4.00E-01 |
| IPI00347019 | Keratin| type II cuticular Hb4 | 2 | 2 | 0 | 2 | 1 | 0.67 | 4.00E-01 |
| IPI00121319 | Cysteine-rich protein 2 | 2 | 2 | 2 | 0 | 1 | 0.67 | 4.00E-01 |
| IPI00116283|IPI00988286 | T-complex protein 1 subunit gamma | 1 | 0 | 1 | 3 | 5 | 2.67 | 4.00E-01 |
| IPI00986042|IPI01023191 | Calsequestrin-1 | 2 | 0 | 2 | 2 | 3 | 1.67 | 4.00E-01 |
| IPI00135231|IPI00762452 | Isocitrate dehydrogenase | 2 | 0 | 2 | 2 | 1 | 1.33 | 4.00E-01 |
| IPI00132443|IPI00480357|IPI00918137|IPI00919164 | Isoform 1 of Heterogeneous nuclear ribonucleoprotein M | 1 | 0 | 1 | 3 | 2 | 2.00 | 4.00E-01 |
| IPI00136984|IPI00850934 | 40S ribosomal protein S7 | 1 | 0 | 1 | 3 | 2 | 2.00 | 4.00E-01 |
| IPI00273803|IPI00473532|IPI00667252|IPI00762221|IPI00874692 | 60S ribosomal protein L15 | 1 | 3 | 0 | 1 | 0 | 0.44 | 4.00E-01 |
| IPI00322492|IPI00515199|IPI00742310 | Uncharacterized protein | 2 | 2 | 0 | 2 | 0 | 0.56 | 4.00E-01 |
| IPI00224784 | Prothymosin alpha | 2 | 2 | 0 | 2 | 0 | 0.56 | 4.00E-01 |
| IPI00170093 | NADH dehydrogenase [ubiquinone] iron-sulfur protein 8| mitochondrial | 2 | 2 | 2 | 0 | 0 | 0.56 | 4.00E-01 |
| IPI00122554 | Isoform 1 of Chaperone activity of bc1 complex-like| mitochondrial | 2 | 2 | 2 | 0 | 0 | 0.56 | 4.00E-01 |
| IPI00230365 | Keratin| type I cytoskeletal 17 | 12 | 13 | 7 | 16 | 15 | 1.01 | 4.03E-01 |
| IPI00162780|IPI00775863 | Guanine nucleotide-binding protein G(I)/G(S)/G(T) subunit beta-2 | 1 | 2 | 2 | 4 | 2 | 1.47 | 4.05E-01 |
| IPI00283862 | Proteasome subunit alpha type-1 | 2 | 1 | 2 | 4 | 2 | 1.47 | 4.05E-01 |
| IPI00849793|IPI00989404 | 60S ribosomal protein L12 | 3 | 3 | 5 | 4 | 3 | 1.25 | 4.05E-01 |
| IPI00123604|IPI00622968 | 40S ribosomal protein SA | 3 | 3 | 5 | 4 | 4 | 1.33 | 4.05E-01 |
| IPI00125778 | Transgelin-2 | 4 | 6 | 6 | 7 | 4 | 1.11 | 4.07E-01 |
| IPI00309997|IPI00775790|IPI00776352 | Isoform 1 of Four and a half LIM domains protein 1 | 7 | 6 | 6 | 4 | 4 | 0.76 | 4.07E-01 |
| IPI00123316 | Isoform 1 of Tropomyosin alpha-1 chain | 21 | 19 | 17 | 19 | 16 | 0.87 | 4.09E-01 |
| IPI00308885 | Isoform 1 of 60 kDa heat shock protein| mitochondrial | 9 | 5 | 7 | 10 | 7 | 1.13 | 4.11E-01 |
| IPI00458583 | Heterogeneous nuclear ribonucleoprotein U | 2 | 3 | 3 | 5 | 1 | 1.14 | 4.13E-01 |
| IPI00223713|IPI00331597 | Histone H1.2 | 4 | 4 | 6 | 5 | 4 | 1.20 | 4.14E-01 |
| IPI00319830 | Isoform 1 of Spectrin beta chain| brain 1 | 7 | 5 | 7 | 8 | 5 | 1.10 | 4.15E-01 |
| IPI00133440 | Prohibitin | 2 | 3 | 0 | 4 | 3 | 0.95 | 4.17E-01 |
| IPI00346073 | Heat shock 70 kDa protein 1B | 5 | 4 | 5 | 7 | 6 | 1.27 | 4.25E-01 |
| IPI00874456 | Dihydrolipoyl dehydrogenase| mitochondrial | 8 | 6 | 5 | 6 | 7 | 0.88 | 4.30E-01 |
| IPI00407130 | Isoform M2 of Pyruvate kinase isozymes M1/M2 | 17 | 17 | 13 | 18 | 19 | 0.98 | 4.32E-01 |
| IPI00337893 | Pyruvate dehydrogenase E1 component subunit alpha| somatic form| mitochondrial | 9 | 7 | 6 | 7 | 6 | 0.81 | 4.34E-01 |
| IPI00131366 | Keratin| type II cytoskeletal 6B | 13 | 16 | 11 | 15 | 14 | 0.92 | 4.39E-01 |
| IPI00118899 | Alpha-actinin-4 | 17 | 16 | 10 | 22 | 16 | 0.97 | 4.40E-01 |
| IPI00117350 | Tubulin alpha-4A chain | 11 | 13 | 13 | 14 | 12 | 1.08 | 4.41E-01 |
| IPI00169925 | Isoform 1 of NADH dehydrogenase [ubiquinone] flavoprotein 2| mitochondrial | 5 | 4 | 5 | 2 | 6 | 0.97 | 4.51E-01 |
| IPI00987580 | Peptidyl-prolyl cis-trans isomerase | 5 | 2 | 5 | 4 | 4 | 1.19 | 4.51E-01 |
| IPI00468481 | ATP synthase subunit beta| mitochondrial | 22 | 24 | 23 | 20 | 22 | 0.94 | 4.55E-01 |
| IPI00753793|IPI00753815 | Isoform 2 of Spectrin alpha chain| brain | 12 | 9 | 5 | 16 | 6 | 0.87 | 4.59E-01 |
| IPI00127596 | Creatine kinase M-type | 10 | 11 | 12 | 7 | 10 | 0.93 | 4.62E-01 |
| IPI00554989 | Peptidyl-prolyl cis-trans isomerase | 6 | 3 | 6 | 5 | 5 | 1.15 | 4.65E-01 |
| IPI00453768|IPI00849652|IPI00971257|IPI00974834|IPI00975020|IPI00975135|IPI00975198|IPI00989704|IPI01007744 | 60S ribosomal protein L17 | 0 | 3 | 2 | 2 | 2 | 1.20 | 4.76E-01 |
| IPI00229055|IPI00229058|IPI00318623|IPI00336780|IPI00465745|IPI00466353|IPI00466881|IPI00469990|IPI00753801|IPI00830453|IPI00830536|IPI00830654|IPI00831350 | Isoform A5e17 of Troponin T| fast skeletal muscle | 5 | 5 | 5 | 3 | 4 | 0.83 | 4.80E-01 |
| IPI00321718 | Prohibitin-2 | 3 | 4 | 1 | 5 | 3 | 0.89 | 4.90E-01 |
| IPI00336324 | Malate dehydrogenase| cytoplasmic | 3 | 4 | 4 | 5 | 3 | 1.11 | 5.00E-01 |
| IPI00114352|IPI00282748|IPI00458777 | Isoform 1 of Myc box-dependent-interacting protein 1 | 5 | 4 | 4 | 3 | 3 | 0.79 | 5.00E-01 |
| IPI00132042 | Pyruvate dehydrogenase E1 component subunit beta| mitochondrial | 4 | 5 | 4 | 3 | 3 | 0.79 | 5.00E-01 |
| IPI00462157 | Tripartite motif-containing protein 72 | 3 | 3 | 2 | 2 | 2 | 0.75 | 5.00E-01 |
| IPI00222430|IPI00667117|IPI00831604 | acyl-CoA-binding protein isoform 1 | 3 | 3 | 2 | 2 | 2 | 0.75 | 5.00E-01 |
| IPI00118153 | Cysteine and glycine-rich protein 3 | 2 | 2 | 3 | 3 | 4 | 1.44 | 5.00E-01 |
| IPI00420261|IPI00665601 | High mobility group protein B1 | 2 | 3 | 4 | 3 | 1 | 1.05 | 5.00E-01 |
| IPI00226073 | Isoform 1 of Heterogeneous nuclear ribonucleoprotein F | 3 | 2 | 3 | 4 | 1 | 1.05 | 5.00E-01 |
| IPI00312700 | Isoform MLC1 of Myosin light chain 1/3| skeletal muscle isoform | 6 | 6 | 5 | 5 | 5 | 0.86 | 5.00E-01 |
| IPI00120719 | Cytochrome c oxidase subunit 5A| mitochondrial | 4 | 3 | 3 | 2 | 1 | 0.67 | 5.00E-01 |
| IPI00273646|IPI00850779 | Glyceraldehyde-3-phosphate dehydrogenase | 3 | 5 | 4 | 2 | 3 | 0.80 | 5.00E-01 |
| IPI00470152 | Ubiquitin-40S ribosomal protein S27a | 1 | 2 | 3 | 2 | 2 | 1.33 | 5.00E-01 |
| IPI00134621 | GTP-binding nuclear protein Ran | 2 | 3 | 1 | 2 | 2 | 0.76 | 5.00E-01 |
| IPI00338536 | Succinate dehydrogenase [ubiquinone] iron-sulfur subunit| mitochondrial | 2 | 3 | 1 | 2 | 1 | 0.67 | 5.00E-01 |
| IPI00331345|IPI00473521|IPI00679159 | 40S ribosomal protein S3a | 0 | 2 | 1 | 3 | 1 | 1.33 | 5.00E-01 |
| IPI00224740 | Profilin-1 | 0 | 2 | 1 | 3 | 2 | 1.50 | 5.00E-01 |
| IPI00312058 | Catalase | 2 | 1 | 0 | 1 | 2 | 0.80 | 5.00E-01 |
| IPI00119876 | Cytoplasmic dynein 1 heavy chain 1 | 2 | 1 | 0 | 1 | 2 | 0.80 | 5.00E-01 |
| IPI00116705 | Fatty acid-binding protein| adipocyte | 2 | 1 | 0 | 1 | 2 | 0.80 | 5.00E-01 |
| IPI00331461|IPI00340036|IPI00474856|IPI00624735 | 60S ribosomal protein L11 | 0 | 1 | 2 | 1 | 0 | 1.33 | 5.00E-01 |
| IPI00228757|IPI00853896 | Isoform Short of Thymosin beta-4 | 2 | 1 | 1 | 0 | 1 | 0.67 | 5.00E-01 |
| IPI00133284 | ES1 protein homolog| mitochondrial | 1 | 0 | 2 | 1 | 2 | 1.78 | 5.00E-01 |
| IPI00112448 | 40S ribosomal protein S10 | 1 | 0 | 2 | 1 | 2 | 1.78 | 5.00E-01 |
| IPI00136563 | Carnitine O-palmitoyltransferase 1| muscle isoform | 1 | 1 | 0 | 0 | 0 | 0.50 | 5.00E-01 |
| IPI00131896 | Brain protein 44 | 1 | 1 | 0 | 0 | 0 | 0.50 | 5.00E-01 |
| IPI00121462 | Keratin| type I cytoskeletal 23 | 1 | 1 | 0 | 0 | 0 | 0.50 | 5.00E-01 |
| IPI00469392 | Isoform 1 of Reticulon-4 | 1 | 1 | 0 | 0 | 0 | 0.50 | 5.00E-01 |
| IPI00113271|IPI00808203|IPI00831354|IPI00831676|IPI00915517 | Isoform 2 of Dysferlin | 1 | 1 | 0 | 0 | 0 | 0.50 | 5.00E-01 |
| IPI00230124 | Fatty acid-binding protein| heart | 0 | 0 | 1 | 1 | 1 | 2.00 | 5.00E-01 |
| IPI00125929 | NADH dehydrogenase [ubiquinone] 1 alpha subcomplex subunit 4 | 1 | 1 | 0 | 0 | 0 | 0.50 | 5.00E-01 |
| IPI00116222|IPI00986304 | 3-hydroxyisobutyrate dehydrogenase| mitochondrial | 1 | 1 | 0 | 0 | 0 | 0.50 | 5.00E-01 |
| IPI00117896 | Microtubule-associated protein RP/EB family member 1 | 0 | 0 | 1 | 1 | 1 | 2.00 | 5.00E-01 |
| IPI00344004 | NADH dehydrogenase (Ubiquinone) 1 alpha subcomplex| 12 | 1 | 1 | 0 | 0 | 0 | 0.50 | 5.00E-01 |
| IPI00127021 | NEDD8 | 1 | 1 | 0 | 0 | 0 | 0.50 | 5.00E-01 |
| IPI00116281 | T-complex protein 1 subunit zeta | 0 | 0 | 1 | 1 | 1 | 2.00 | 5.00E-01 |
| IPI00387379 | 2|4-dienoyl-CoA reductase| mitochondrial | 1 | 1 | 0 | 0 | 0 | 0.50 | 5.00E-01 |
| IPI00130883 | Putative RNA-binding protein 3 | 0 | 0 | 1 | 1 | 1 | 2.00 | 5.00E-01 |
| IPI00111013|IPI00404551 | Cathepsin D | 0 | 0 | 1 | 1 | 1 | 2.00 | 5.00E-01 |
| IPI00136119 | Uncharacterized protein | 2 | 1 | 1 | 0 | 0 | 0.53 | 5.00E-01 |
| IPI00474637|IPI00775915|IPI00849598|IPI00915054 | 60S ribosomal protein L10 | 1 | 2 | 1 | 0 | 0 | 0.53 | 5.00E-01 |
| IPI00316740 | DNA damage-binding protein 1 | 0 | 0 | 1 | 1 | 0 | 1.67 | 5.00E-01 |
| IPI00309768 | PDZ and LIM domain protein 1 | 0 | 0 | 1 | 1 | 0 | 1.67 | 5.00E-01 |
| IPI00339468|IPI00875791 | Uncharacterized protein | 0 | 0 | 1 | 1 | 0 | 1.67 | 5.00E-01 |
| IPI00109588 | Collagen alpha-1(IV) chain | 0 | 0 | 1 | 1 | 0 | 1.67 | 5.00E-01 |
| IPI00125143|IPI00462975 | Arpc1b protein | 0 | 0 | 1 | 1 | 0 | 1.67 | 5.00E-01 |
| IPI00266281 | Thioredoxin-like protein 1 | 0 | 0 | 1 | 1 | 0 | 1.67 | 5.00E-01 |
| IPI00556871 | Uncharacterized protein | 0 | 0 | 1 | 1 | 0 | 1.67 | 5.00E-01 |
| IPI00661414|IPI00775844 | Actin-related protein 2/3 complex subunit 2 | 0 | 0 | 1 | 1 | 0 | 1.67 | 5.00E-01 |
| IPI00756745|IPI00874362|IPI00989412 | laminin subunit alpha-2 | 0 | 0 | 1 | 1 | 2 | 2.33 | 5.00E-01 |
| IPI00111885 | Cytochrome b-c1 complex subunit 1| mitochondrial | 4 | 4 | 3 | 3 | 4 | 0.87 | 5.00E-01 |
| IPI00123639 | Calreticulin | 3 | 5 | 4 | 6 | 4 | 1.13 | 5.00E-01 |
| IPI00338039 | Tubulin beta-2A chain | 13 | 12 | 14 | 13 | 14 | 1.09 | 5.00E-01 |
| IPI00117352 | Tubulin beta-5 chain | 19 | 16 | 16 | 17 | 18 | 0.97 | 5.06E-01 |
| IPI00466069 | Elongation factor 2 | 17 | 19 | 17 | 21 | 19 | 1.05 | 5.07E-01 |
| IPI00123313 | Ubiquitin-like modifier-activating enzyme 1 | 3 | 2 | 2 | 6 | 1 | 1.14 | 5.10E-01 |
| IPI00990246 | Nucleoside diphosphate kinase | 4 | 4 | 4 | 6 | 3 | 1.07 | 5.20E-01 |
| IPI00318841 | Elongation factor 1-gamma | 4 | 6 | 4 | 4 | 5 | 0.89 | 5.20E-01 |
| IPI00108125 | Eukaryotic translation initiation factor 5A-1 | 3 | 3 | 3 | 5 | 3 | 1.17 | 5.29E-01 |
| IPI00229963 | Serpin B5 | 5 | 3 | 3 | 3 | 1 | 0.67 | 5.29E-01 |
| IPI00314950|IPI00626366 | 60S acidic ribosomal protein P0 | 2 | 4 | 2 | 2 | 4 | 0.92 | 5.48E-01 |
| IPI00139301 | Keratin| type II cytoskeletal 5 | 13 | 15 | 12 | 15 | 15 | 1.00 | 5.49E-01 |
| IPI00110850|IPI00874482 | Actin| cytoplasmic 1 | 18 | 17 | 17 | 19 | 17 | 1.01 | 5.49E-01 |
| IPI00380436|IPI00989903 | Alpha-actinin-1 | 14 | 13 | 11 | 21 | 20 | 1.26 | 5.52E-01 |
| IPI00117348 | Tubulin alpha-1B chain | 13 | 14 | 14 | 14 | 13 | 1.01 | 5.53E-01 |
| IPI00136929 | Gamma actin-like protein | 16 | 14 | 14 | 17 | 15 | 1.02 | 5.55E-01 |
| IPI00555069 | Phosphoglycerate kinase 1 | 10 | 11 | 11 | 11 | 9 | 0.99 | 5.59E-01 |
| IPI00323592 | Malate dehydrogenase| mitochondrial | 11 | 11 | 11 | 10 | 10 | 0.94 | 5.59E-01 |
| IPI00224575|IPI00890005 | Isoform 2 of Heterogeneous nuclear ribonucleoprotein K | 10 | 8 | 9 | 10 | 7 | 0.97 | 5.60E-01 |
| IPI00134599 | 40S ribosomal protein S3 | 5 | 3 | 3 | 7 | 2 | 1.00 | 5.63E-01 |
| IPI00405227 | Vinculin | 7 | 7 | 7 | 6 | 6 | 0.92 | 5.73E-01 |
| IPI00133708 | Putative ATP-dependent RNA helicase Pl10 | 3 | 2 | 2 | 5 | 5 | 1.43 | 5.76E-01 |
| IPI00471475|IPI00471476|IPI00471477|IPI00884509 | Isoform 1 of Plasminogen activator inhibitor 1 RNA-binding protein | 3 | 2 | 2 | 5 | 2 | 1.14 | 5.76E-01 |
| IPI00128904 | Poly(rC)-binding protein 1 | 2 | 5 | 3 | 2 | 4 | 0.89 | 5.76E-01 |
| IPI00331092|IPI00990327 | 40S ribosomal protein S4| X isoform | 2 | 3 | 2 | 5 | 4 | 1.33 | 5.76E-01 |
| IPI00269661|IPI00269662|IPI00459722|IPI00466185|IPI00986859|IPI00987145 | Isoform 1 of Heterogeneous nuclear ribonucleoprotein A3 | 6 | 5 | 6 | 6 | 6 | 1.08 | 5.79E-01 |
| IPI00114209 | Glutamate dehydrogenase 1| mitochondrial | 5 | 4 | 5 | 3 | 2 | 0.79 | 5.81E-01 |
| IPI00133522 | Protein disulfide-isomerase | 6 | 6 | 7 | 6 | 6 | 1.05 | 5.82E-01 |
| IPI00320459 | Epiplakin | 2 | 2 | 0 | 5 | 3 | 1.22 | 5.83E-01 |
| IPI00317309 | Annexin A5 | 5 | 5 | 4 | 5 | 5 | 0.94 | 5.86E-01 |
| IPI00117167 | Isoform 1 of Gelsolin | 2 | 4 | 4 | 3 | 2 | 1.00 | 5.87E-01 |
| IPI00123194 | Biglycan | 4 | 2 | 4 | 3 | 5 | 1.25 | 5.87E-01 |
| IPI00354819|IPI00409817 | Isoform Smooth muscle of Myosin light polypeptide 6 | 5 | 5 | 5 | 6 | 7 | 1.17 | 5.90E-01 |
| IPI00230204 | Aspartate aminotransferase| cytoplasmic | 4 | 4 | 4 | 3 | 3 | 0.87 | 5.95E-01 |
| IPI00113241|IPI00875584|IPI00990110 | 40S ribosomal protein S19 | 2 | 3 | 1 | 3 | 3 | 0.95 | 5.95E-01 |
| IPI00228633 | Glucose-6-phosphate isomerase | 2 | 3 | 3 | 1 | 2 | 0.86 | 5.95E-01 |
| IPI00323483|IPI00453818|IPI00944688 | Isoform 3 of Programmed cell death 6-interacting protein | 2 | 1 | 0 | 2 | 3 | 1.07 | 6.00E-01 |
| IPI00284806 | Hypoxanthine-guanine phosphoribosyltransferase | 0 | 2 | 2 | 1 | 0 | 1.00 | 6.00E-01 |
| IPI00229517 | Galectin-1 | 2 | 1 | 0 | 2 | 2 | 0.93 | 6.00E-01 |
| IPI00172221|IPI00556723|IPI00556781|IPI00874710 | Isoform 2 of Dynamin-1-like protein | 1 | 2 | 0 | 2 | 3 | 1.07 | 6.00E-01 |
| IPI00331174 | T-complex protein 1 subunit eta | 0 | 2 | 1 | 2 | 2 | 1.33 | 6.00E-01 |
| IPI00380130|IPI00987518|IPI00988545 | Putative uncharacterized protein | 2 | 1 | 0 | 2 | 1 | 0.80 | 6.00E-01 |
| IPI00468203 | Annexin A2 | 13 | 10 | 11 | 12 | 14 | 1.07 | 6.07E-01 |
| IPI00227930|IPI00282266|IPI00282269|IPI00348270 | Histone H2B type 1-H | 3 | 3 | 2 | 3 | 3 | 0.92 | 6.08E-01 |
| IPI00121788 | Peroxiredoxin-1 | 3 | 4 | 3 | 3 | 1 | 0.74 | 6.17E-01 |
| IPI00129517|IPI00759999 | Isoform Mitochondrial of Peroxiredoxin-5| mitochondrial | 3 | 4 | 3 | 3 | 2 | 0.81 | 6.17E-01 |
| IPI00230706 | Phosphoglycerate mutase 2 | 4 | 3 | 3 | 3 | 3 | 0.89 | 6.17E-01 |
| IPI00117689 | Polymerase I and transcript release factor | 3 | 3 | 4 | 3 | 3 | 1.08 | 6.17E-01 |
| IPI00127942 | Destrin | 2 | 2 | 2 | 1 | 0 | 0.67 | 6.29E-01 |
| IPI00121105 | Hydroxyacyl-coenzyme A dehydrogenase| mitochondrial | 2 | 2 | 2 | 1 | 0 | 0.67 | 6.29E-01 |
| IPI00117957 | Asporin | 2 | 2 | 2 | 1 | 0 | 0.67 | 6.29E-01 |
| IPI00118986 | ATP synthase subunit O| mitochondrial | 2 | 2 | 2 | 1 | 2 | 0.89 | 6.29E-01 |
| IPI00406442|IPI00986302 | Succinyl-CoA ligase [GDP-forming] subunit alpha| mitochondrial | 2 | 2 | 2 | 1 | 2 | 0.89 | 6.29E-01 |
| IPI00113214|IPI00881918 | Ubiquitin carboxyl-terminal hydrolase 5 | 2 | 2 | 1 | 2 | 2 | 0.89 | 6.29E-01 |
| IPI00227140 | Keratin| type I cytoskeletal 14 | 13 | 12 | 10 | 16 | 12 | 1.01 | 6.29E-01 |
| IPI00620256 | Isoform A of Prelamin-A/C | 18 | 18 | 20 | 17 | 20 | 1.05 | 6.32E-01 |
| IPI00134809 | Isoform 1 of Dihydrolipoyllysine-residue succinyltransferase component of 2-oxoglutarate dehydrogenase complex| mitochondrial | 3 | 2 | 2 | 2 | 0 | 0.67 | 6.43E-01 |
| IPI00230395 | Annexin A1 | 2 | 2 | 3 | 2 | 1 | 1.00 | 6.43E-01 |
| IPI00223196 | Troponin I| fast skeletal muscle | 2 | 2 | 3 | 2 | 1 | 1.00 | 6.43E-01 |
| IPI00848816 | cofilin-1-like | 2 | 2 | 2 | 3 | 1 | 1.00 | 6.43E-01 |
| IPI00985626 | Uncharacterized protein | 2 | 3 | 2 | 2 | 2 | 0.86 | 6.43E-01 |
| IPI00330363|IPI00354363|IPI00988887|IPI01018378 | 60S ribosomal protein L7a | 2 | 1 | 4 | 1 | 1 | 1.20 | 6.43E-01 |
| IPI00310091 | Serine/threonine-protein phosphatase 2A 65 kDa regulatory subunit A alpha isoform | 6 | 3 | 4 | 5 | 4 | 0.97 | 6.48E-01 |
| IPI00225634|IPI00473415 | 40S ribosomal protein S12 | 2 | 4 | 2 | 3 | 2 | 0.83 | 6.52E-01 |
| IPI00120886 | Nuclease-sensitive element-binding protein 1 | 2 | 3 | 4 | 2 | 4 | 1.24 | 6.52E-01 |
| IPI00169916|IPI00648173 | Clathrin heavy chain 1 | 8 | 5 | 4 | 10 | 6 | 1.02 | 6.60E-01 |
| IPI00459945 | Myozenin-1 | 1 | 4 | 2 | 3 | 2 | 0.95 | 6.67E-01 |
| IPI00266188 | Cofilin-2 | 3 | 2 | 1 | 4 | 2 | 0.95 | 6.67E-01 |
| IPI00461456|IPI00752639|IPI00752985|IPI00754976|IPI00849847|IPI00874935 | 60S ribosomal protein L23a | 0 | 1 | 1 | 1 | 0 | 1.11 | 6.67E-01 |
| IPI00114162 | Fatty acid-binding protein| epidermal | 0 | 1 | 1 | 1 | 1 | 1.33 | 6.67E-01 |
| IPI00263863 | 10 kDa heat shock protein| mitochondrial | 1 | 1 | 1 | 0 | 1 | 0.83 | 6.67E-01 |
| IPI00111222 | Aldehyde dehydrogenase| dimeric NADP-preferring | 1 | 1 | 1 | 0 | 0 | 0.67 | 6.67E-01 |
| IPI00117910 | Peroxiredoxin-2 | 1 | 0 | 1 | 1 | 0 | 1.11 | 6.67E-01 |
| IPI00120503|IPI00989020 | Coatomer subunit beta | 1 | 0 | 1 | 1 | 1 | 1.33 | 6.67E-01 |
| IPI00272033|IPI00330000|IPI00974916|IPI00989397 | Histone H2A type 2-C | 4 | 5 | 4 | 5 | 7 | 1.15 | 6.81E-01 |
| IPI00124692 | Transaldolase | 2 | 4 | 3 | 3 | 2 | 0.92 | 6.89E-01 |
| IPI00406800 | Isoform 1 of F-actin-capping protein subunit beta | 3 | 3 | 4 | 2 | 3 | 1.00 | 6.89E-01 |
| IPI00467374 | PDZ and LIM domain protein 5 isoform ENH4 | 1 | 2 | 1 | 1 | 0 | 0.67 | 7.00E-01 |
| IPI00123494 | 26S proteasome non-ATPase regulatory subunit 2 | 1 | 1 | 2 | 1 | 1 | 1.17 | 7.00E-01 |
| IPI00124111 | Plakophilin-1 | 7 | 7 | 5 | 11 | 5 | 1.00 | 7.04E-01 |
| IPI00109142 | S-formylglutathione hydrolase | 2 | 1 | 1 | 3 | 0 | 0.93 | 7.14E-01 |
| IPI00137787 | 60S ribosomal protein L8 | 1 | 3 | 1 | 2 | 3 | 1.00 | 7.14E-01 |
| IPI00130344 | Chloride intracellular channel protein 1 | 1 | 3 | 2 | 2 | 1 | 0.89 | 7.14E-01 |
| IPI00222419|IPI00987305 | Cytochrome c| somatic | 2 | 2 | 3 | 1 | 1 | 0.89 | 7.14E-01 |
| IPI00277001|IPI00987554 | Proteasome subunit alpha type-4 | 3 | 3 | 3 | 3 | 2 | 0.92 | 7.16E-01 |
| IPI00311873 | Serine/threonine-protein phosphatase PP1-beta catalytic subunit | 4 | 2 | 2 | 4 | 5 | 1.17 | 7.27E-01 |
| IPI00137331 | Adenylyl cyclase-associated protein 1 | 3 | 2 | 2 | 3 | 3 | 1.05 | 7.38E-01 |
| IPI00284119 | Troponin C| skeletal muscle | 3 | 2 | 3 | 2 | 2 | 0.95 | 7.38E-01 |
| IPI00119065 | Laminin subunit beta-2 | 1 | 1 | 2 | 0 | 0 | 0.83 | 7.50E-01 |
| IPI00109823 | 1|4-alpha-glucan-branching enzyme | 1 | 1 | 0 | 2 | 0 | 0.83 | 7.50E-01 |
| IPI00315100 | Transforming protein RhoA | 1 | 1 | 2 | 0 | 0 | 0.83 | 7.50E-01 |
| IPI00124820 | Coronin-1C | 1 | 1 | 0 | 2 | 0 | 0.83 | 7.50E-01 |
| IPI00421223 | Tropomyosin alpha-4 chain | 5 | 2 | 3 | 3 | 2 | 0.81 | 7.51E-01 |
| IPI00165854|IPI00850217 | Ubiquitin-conjugating enzyme E2 N | 2 | 2 | 2 | 2 | 0 | 0.78 | 7.57E-01 |
| IPI00313900 | Lumican | 2 | 2 | 2 | 2 | 0 | 0.78 | 7.57E-01 |
| IPI00555059 | Peroxiredoxin-6 | 2 | 2 | 2 | 2 | 1 | 0.89 | 7.57E-01 |
| IPI00126635 | isocitrate dehydrogenase 3| beta subunit | 2 | 2 | 2 | 2 | 1 | 0.89 | 7.57E-01 |
| IPI00316491 | Hemoglobin subunit beta-2 | 2 | 2 | 2 | 2 | 2 | 1.00 | 7.57E-01 |
| IPI00111831 | Nascent polypeptide-associated complex subunit alpha| muscle-specific form | 2 | 2 | 2 | 2 | 2 | 1.00 | 7.57E-01 |
| IPI00762198 | Beta-globin | 2 | 2 | 2 | 2 | 2 | 1.00 | 7.57E-01 |
| IPI00985596 | hemoglobin subunit beta-1-like isoform 5 | 2 | 2 | 2 | 2 | 2 | 1.00 | 7.57E-01 |
| IPI00117264|IPI00895414 | Protein DJ-1 | 2 | 2 | 2 | 2 | 2 | 1.00 | 7.57E-01 |
| IPI00469114 | Hemoglobin subunit alpha | 2 | 2 | 2 | 2 | 2 | 1.00 | 7.57E-01 |
| IPI00131695 | Serum albumin | 1 | 2 | 2 | 1 | 0 | 0.80 | 8.00E-01 |
| IPI00116279 | T-complex protein 1 subunit epsilon | 2 | 1 | 2 | 1 | 0 | 0.80 | 8.00E-01 |
| IPI00322562 | 40S ribosomal protein S14 | 2 | 1 | 2 | 1 | 1 | 0.93 | 8.00E-01 |
| IPI00130445 | Thrombospondin-4 | 1 | 2 | 1 | 2 | 2 | 1.07 | 8.00E-01 |
| IPI00129685 | Translationally-controlled tumor protein | 1 | 1 | 1 | 1 | 0 | 0.83 | 8.33E-01 |
| IPI00322209 | Keratin| type II cytoskeletal 8 | 0 | 4 | 0 | 6 | 7 | 1.78 | 1.00E+00 |
| IPI00114342|IPI00989451 | Hexokinase-2 | 0 | 5 | 0 | 2 | 2 | 0.67 | 1.00E+00 |
| IPI00261627 | Succinyl-CoA ligase [ADP-forming] subunit beta| mitochondrial | 3 | 0 | 2 | 0 | 2 | 0.93 | 1.00E+00 |
| IPI00120870|IPI00338018|IPI00339003|IPI00400421 | Isoform 2 of Periostin | 2 | 0 | 0 | 3 | 3 | 1.50 | 1.00E+00 |
| IPI00135640|IPI00462789 | 26S protease regulatory subunit 8 | 0 | 1 | 0 | 3 | 1 | 1.56 | 1.00E+00 |
| IPI00280207 | Isoform 2 of Ubiquilin-1 | 2 | 0 | 0 | 3 | 0 | 1.00 | 1.00E+00 |
| IPI00108454|IPI00113655|IPI00474622 | 40S ribosomal protein S6 | 2 | 0 | 0 | 3 | 2 | 1.33 | 1.00E+00 |
| IPI00112414|IPI00985755 | Exportin-2 | 2 | 0 | 0 | 0 | 1 | 0.67 | 1.00E+00 |
| IPI00122438 | Fibrillin-1 | 1 | 0 | 0 | 0 | 2 | 1.11 | 1.00E+00 |
| IPI00109073 | Tubulin beta-4 chain | 0 | 0 | 0 | 17 | 15 | 11.67 | 1.00E+00 |
| IPI00130185 | Serine/threonine-protein phosphatase PP1-alpha catalytic subunit | 0 | 0 | 0 | 6 | 6 | 5.00 | 1.00E+00 |
| IPI00331556 | Heat shock 70 kDa protein 4 | 1 | 0 | 0 | 4 | 2 | 2.00 | 1.00E+00 |
| IPI00117063|IPI00830623 | RNA-binding protein FUS | 0 | 0 | 0 | 3 | 1 | 2.33 | 1.00E+00 |
| IPI00124223 | Proteasome activator complex subunit 1 | 0 | 0 | 3 | 0 | 2 | 2.67 | 1.00E+00 |
| IPI00115117|IPI00648577|IPI00649391 | Stomatin-like protein 2 | 0 | 0 | 0 | 3 | 1 | 2.33 | 1.00E+00 |
| IPI00128441 | Putative uncharacterized protein | 0 | 0 | 0 | 2 | 3 | 2.67 | 1.00E+00 |
| IPI00126716 | Eukaryotic initiation factor 4A-III | 0 | 0 | 0 | 3 | 2 | 2.67 | 1.00E+00 |
| IPI00132756 | Annexin A8 | 0 | 0 | 0 | 3 | 2 | 2.67 | 1.00E+00 |
| IPI00316623|IPI00403823|IPI00658280|IPI00660602|IPI00663949|IPI00676225|IPI00676489|IPI00750243|IPI00751207|IPI00752108|IPI00752593|IPI00753220|IPI00754401|IPI00754980|IPI00755935|IPI00756875|IPI00757459 | Isoform 3 of Catenin delta-1 | 0 | 0 | 0 | 2 | 3 | 2.67 | 1.00E+00 |
| IPI00339885 | Collagen alpha-1(VI) chain | 1 | 0 | 0 | 2 | 2 | 1.56 | 1.00E+00 |
| IPI00896727 | Cullin-associated NEDD8-dissociated protein 1 | 0 | 0 | 0 | 1 | 1 | 1.67 | 1.00E+00 |
| IPI01008333 | myelin protein P0 precursor | 0 | 0 | 1 | 0 | 1 | 1.67 | 1.00E+00 |
| IPI00269613 | Eukaryotic translation initiation factor 3 subunit I | 0 | 0 | 0 | 1 | 1 | 1.67 | 1.00E+00 |
| IPI00274739|IPI00753114 | Isoform 2 of DNA-binding protein A | 0 | 0 | 1 | 0 | 1 | 1.67 | 1.00E+00 |
| IPI00126191 | Isoform B2 of Lamin-B2 | 0 | 0 | 0 | 1 | 1 | 1.67 | 1.00E+00 |
| IPI00115580 | Eukaryotic translation initiation factor 3 subunit M | 0 | 0 | 0 | 1 | 1 | 1.67 | 1.00E+00 |
| IPI00123199|IPI00929813|IPI00990323 | Nucleosome assembly protein 1-like 1 | 0 | 0 | 0 | 1 | 1 | 1.67 | 1.00E+00 |
| IPI00311236|IPI01023242 | 60S ribosomal protein L7 | 0 | 0 | 0 | 1 | 1 | 1.67 | 1.00E+00 |
| IPI00267295 | 26S proteasome non-ATPase regulatory subunit 1 | 0 | 0 | 0 | 1 | 1 | 1.67 | 1.00E+00 |
| IPI00112251 | Tubulin beta-3 chain | 0 | 0 | 0 | 11 | 11 | 8.33 | 1.00E+00 |
| IPI00626132|IPI00830581 | Uncharacterized protein | 0 | 0 | 0 | 2 | 1 | 2.00 | 1.00E+00 |
| IPI00222546 | 60S ribosomal protein L22 | 0 | 0 | 0 | 2 | 1 | 2.00 | 1.00E+00 |
| IPI00323881 | Importin subunit beta-1 | 0 | 0 | 0 | 2 | 1 | 2.00 | 1.00E+00 |
| IPI00555004 | Alcohol dehydrogenase class-3 | 0 | 0 | 0 | 2 | 1 | 2.00 | 1.00E+00 |
| IPI00154004|IPI00928020 | Ubiquitin thioesterase OTUB1 | 0 | 0 | 0 | 2 | 1 | 2.00 | 1.00E+00 |
| IPI00308938 | Calpain-2 catalytic subunit | 0 | 0 | 0 | 1 | 2 | 2.00 | 1.00E+00 |
| IPI00121430|IPI00776316|IPI00830892|IPI00881909 | Isoform 1 of Collagen alpha-1(XII) chain | 0 | 0 | 0 | 4 | 2 | 3.00 | 1.00E+00 |
| IPI00652919 | Isoform 1 of Poly(U)-binding-splicing factor PUF60 | 0 | 0 | 0 | 4 | 2 | 3.00 | 1.00E+00 |
| IPI00762774 | Eukaryotic translation initiation factor 3 subunit D | 0 | 0 | 0 | 2 | 1 | 2.00 | 1.00E+00 |
| IPI00126248|IPI00762047 | ATP-citrate synthase isoform 1 | 0 | 0 | 0 | 2 | 1 | 2.00 | 1.00E+00 |
| IPI00120162|IPI00850570 | Casein kinase II subunit alpha | 0 | 0 | 0 | 1 | 2 | 2.00 | 1.00E+00 |
| IPI00407517|IPI00759952|IPI00760078|IPI01007768|IPI01007844 | Uncharacterized protein | 0 | 0 | 0 | 1 | 2 | 2.00 | 1.00E+00 |
| IPI00987113|IPI00990575 | 60S ribosomal protein L30-like | 0 | 0 | 0 | 4 | 3 | 3.33 | 1.00E+00 |
| IPI00336913 | Tripartite motif-containing protein 29 | 0 | 0 | 0 | 4 | 3 | 3.33 | 1.00E+00 |
| IPI00315135 | Mitochondrial import receptor subunit TOM22 homolog | 0 | 0 | 0 | 2 | 1 | 2.00 | 1.00E+00 |
| IPI00230133 | Histone H1.5 | 0 | 0 | 2 | 0 | 1 | 2.00 | 1.00E+00 |
| IPI00122011|IPI00625759 | Isoform 1 of Splicing factor 3B subunit 3 | 0 | 0 | 0 | 3 | 4 | 3.33 | 1.00E+00 |
| IPI00130992|IPI00830335|IPI00957112 | Calpain small subunit 1 | 0 | 0 | 0 | 2 | 1 | 2.00 | 1.00E+00 |
| IPI00469268 | T-complex protein 1 subunit theta | 0 | 0 | 0 | 2 | 1 | 2.00 | 1.00E+00 |
| IPI00134135|IPI00556850|IPI00828470 | Isoform 1 of Low molecular weight phosphotyrosine protein phosphatase | 0 | 1 | 0 | 0 | 0 | 0.67 | 1.00E+00 |
| IPI00113824 | Basement membrane-specific heparan sulfate proteoglycan core protein | 9 | 0 | 0 | 0 | 0 | 0.18 | 1.00E+00 |
| IPI00121534 | Carbonic anhydrase 2 | 0 | 1 | 0 | 0 | 0 | 0.67 | 1.00E+00 |
| IPI00331334 | BAG family molecular chaperone regulator 3 | 1 | 0 | 0 | 0 | 0 | 0.67 | 1.00E+00 |
| IPI00623845 | Selenium-binding protein 1 | 0 | 2 | 0 | 0 | 0 | 0.50 | 1.00E+00 |
| IPI00471246 | Isovaleryl-CoA dehydrogenase| mitochondrial | 1 | 0 | 0 | 0 | 0 | 0.67 | 1.00E+00 |
| IPI00230013|IPI00319970|IPI00407868|IPI00410982|IPI00626793 | Isoform 2B of Voltage-dependent calcium channel subunit alpha-2/delta-1 | 1 | 0 | 0 | 0 | 0 | 0.67 | 1.00E+00 |
| IPI00453996|IPI00608077|IPI00900442 | Isoform 1 of Myosin-14 | 0 | 2 | 0 | 0 | 0 | 0.50 | 1.00E+00 |
| IPI00226515 | Transgelin | 0 | 3 | 0 | 0 | 0 | 0.40 | 1.00E+00 |
| IPI00403589|IPI00988949 | Enhancer of rudimentary homolog | 0 | 2 | 0 | 0 | 0 | 0.50 | 1.00E+00 |
| IPI00115866|IPI00880581|IPI00988643 | Isoform 1 of Hydroxyacylglutathione hydrolase| mitochondrial | 2 | 0 | 0 | 0 | 0 | 0.50 | 1.00E+00 |
| IPI00311493 | Keratin| type I cytoskeletal 18 | 0 | 2 | 0 | 0 | 0 | 0.50 | 1.00E+00 |
| IPI00626860|IPI00874436 | Isoform 2 of Malignant T cell-amplified sequence 1 | 0 | 1 | 0 | 0 | 0 | 0.67 | 1.00E+00 |
| IPI00113347|IPI01016108 | Carnitine O-acetyltransferase | 2 | 0 | 0 | 0 | 0 | 0.50 | 1.00E+00 |
| IPI00378974 | Myomesin-3 | 1 | 0 | 0 | 0 | 0 | 0.67 | 1.00E+00 |
| IPI00131406 | Proteasome subunit alpha type-7 | 0 | 1 | 0 | 0 | 0 | 0.67 | 1.00E+00 |
| IPI00230185 | Glycerol-3-phosphate dehydrogenase [NAD+]| cytoplasmic | 0 | 2 | 0 | 0 | 0 | 0.50 | 1.00E+00 |
| IPI00112549|IPI00857226 | Long-chain-fatty-acid--CoA ligase 1 | 0 | 2 | 0 | 0 | 0 | 0.50 | 1.00E+00 |
| IPI00129857 | Anterior gradient protein 2 homolog | 0 | 1 | 0 | 0 | 0 | 0.67 | 1.00E+00 |
| IPI00121135|IPI00474430 | Serine/arginine-rich splicing factor 2 | 1 | 0 | 0 | 0 | 0 | 0.67 | 1.00E+00 |
| IPI00459487|IPI00665996 | Isoform 1 of Succinyl-CoA ligase [GDP-forming] subunit beta| mitochondrial | 0 | 1 | 0 | 0 | 0 | 0.67 | 1.00E+00 |
| IPI00123342 | Hypoxia up-regulated protein 1 | 0 | 1 | 0 | 0 | 0 | 0.67 | 1.00E+00 |
| IPI00274222 | Isobutyryl-CoA dehydrogenase| mitochondrial | 0 | 2 | 0 | 0 | 0 | 0.50 | 1.00E+00 |
| IPI00308976 | NADP-dependent malic enzyme| mitochondrial | 0 | 1 | 0 | 0 | 0 | 0.67 | 1.00E+00 |
| IPI00380195 | Phosphoglycolate phosphatase | 1 | 0 | 0 | 0 | 0 | 0.67 | 1.00E+00 |
| IPI00122548|IPI00991021 | Voltage-dependent anion-selective channel protein 3 | 0 | 2 | 0 | 0 | 0 | 0.50 | 1.00E+00 |
| IPI00313222|IPI00626312 | 60S ribosomal protein L6 | 0 | 1 | 0 | 0 | 0 | 0.67 | 1.00E+00 |
| IPI00125971 | 26S protease regulatory subunit 10B | 0 | 1 | 0 | 0 | 0 | 0.67 | 1.00E+00 |
| IPI00462445 | E3 ubiquitin-protein ligase NEDD4 | 2 | 0 | 0 | 0 | 0 | 0.50 | 1.00E+00 |
| IPI00121623 | Dynein light chain 1| cytoplasmic | 0 | 3 | 0 | 0 | 0 | 0.40 | 1.00E+00 |
| IPI00137658 | Keratin| type I cytoskeletal 24 | 0 | 1 | 0 | 0 | 0 | 0.67 | 1.00E+00 |
| IPI00336889|IPI00944111|IPI00988797 | Isoform SERCA3B of Sarcoplasmic/endoplasmic reticulum calcium ATPase 3 | 5 | 0 | 0 | 0 | 0 | 0.29 | 1.00E+00 |
| IPI00132734 | Dynein light chain 2| cytoplasmic | 0 | 2 | 0 | 0 | 0 | 0.50 | 1.00E+00 |
| IPI00122696|IPI00828412 | Uncharacterized protein | 1 | 0 | 0 | 0 | 0 | 0.67 | 1.00E+00 |
| IPI00226140 | Amine oxidase [flavin-containing] B | 0 | 1 | 0 | 0 | 0 | 0.67 | 1.00E+00 |
| IPI00845840 | Isoform M1 of Pyruvate kinase isozymes M1/M2 | 0 | 16 | 0 | 0 | 0 | 0.11 | 1.00E+00 |
| IPI00309964|IPI00323166|IPI00762127|IPI00874685|IPI00875011 | Uncharacterized protein | 0 | 1 | 0 | 0 | 0 | 0.67 | 1.00E+00 |
| IPI00125592|IPI00653062 | Ubiquinone biosynthesis protein COQ7 homolog | 2 | 0 | 0 | 0 | 0 | 0.50 | 1.00E+00 |
| IPI00828796 | insulin-degrading enzyme | 1 | 0 | 0 | 0 | 0 | 0.67 | 1.00E+00 |
| IPI00229527 | Leukotriene A-4 hydrolase | 0 | 2 | 0 | 0 | 0 | 0.50 | 1.00E+00 |
| IPI00311175 | Tubulin alpha-8 chain | 0 | 7 | 0 | 0 | 0 | 0.22 | 1.00E+00 |
| IPI00454008 | Serine hydroxymethyltransferase | 0 | 2 | 0 | 0 | 0 | 0.50 | 1.00E+00 |
| IPI00138251 | Dermatopontin | 0 | 2 | 0 | 0 | 0 | 0.50 | 1.00E+00 |
| IPI00116535|IPI00119220|IPI00125265|IPI00605628|IPI00989118 | Uncharacterized protein | 0 | 1 | 0 | 0 | 0 | 0.67 | 1.00E+00 |
| IPI00132605|IPI00844770 | Isoform 1 of Thioesterase superfamily member 5 | 0 | 1 | 0 | 0 | 0 | 0.67 | 1.00E+00 |
| IPI00131357|IPI00972920|IPI00990390 | Uncharacterized protein | 1 | 0 | 0 | 0 | 0 | 0.67 | 1.00E+00 |
| IPI00108189 | Histidine triad nucleotide-binding protein 1 | 1 | 0 | 0 | 0 | 0 | 0.67 | 1.00E+00 |
| IPI00857345|IPI00944141 | Uncharacterized protein | 0 | 2 | 0 | 0 | 0 | 0.50 | 1.00E+00 |
| IPI00308691 | Solute carrier family 2| facilitated glucose transporter member 1 | 0 | 0 | 0 | 3 | 0 | 2.00 | 1.00E+00 |
| IPI00136483 | Proteasome subunit beta type-7 | 0 | 0 | 0 | 0 | 1 | 1.33 | 1.00E+00 |
| IPI00462291 | High mobility group protein B2 | 0 | 0 | 1 | 0 | 0 | 1.33 | 1.00E+00 |
| IPI00323624 | Isoform Long of Complement C3 (Fragment) | 0 | 0 | 1 | 0 | 0 | 1.33 | 1.00E+00 |
| IPI00108143 | Heterogeneous nuclear ribonucleoprotein H2 | 0 | 0 | 3 | 0 | 0 | 2.00 | 1.00E+00 |
| IPI00828528 | Type I epidermal keratin (Fragment) | 0 | 0 | 0 | 5 | 0 | 2.67 | 1.00E+00 |
| IPI00279079 | Fibrinogen beta chain | 0 | 0 | 0 | 6 | 0 | 3.00 | 1.00E+00 |
| IPI00110807|IPI00850057|IPI00853743|IPI00986055|IPI00990637 | MCG129835| isoform CRA_b | 0 | 0 | 1 | 0 | 0 | 1.33 | 1.00E+00 |
| IPI00387370|IPI00608037|IPI00662657|IPI00750471|IPI00857499 | UDP-N-acetylhexosamine pyrophosphorylase-like | 0 | 0 | 0 | 0 | 1 | 1.33 | 1.00E+00 |
| IPI00621272|IPI00986795|IPI00987184|IPI00990554 | NHP2-like protein 1 | 0 | 0 | 2 | 0 | 0 | 1.67 | 1.00E+00 |
| IPI00223714 | Histone H1.4 | 0 | 0 | 4 | 0 | 0 | 2.33 | 1.00E+00 |
| IPI00130095 | Ras GTPase-activating protein-binding protein 1 | 0 | 0 | 0 | 2 | 0 | 1.67 | 1.00E+00 |
| IPI00228820 | Glutathione S-transferase Mu 2 | 0 | 0 | 1 | 0 | 0 | 1.33 | 1.00E+00 |
| IPI00663587 | Heterogeneous nuclear ribonucleoprotein G-like 1 | 0 | 0 | 0 | 2 | 0 | 1.67 | 1.00E+00 |
| IPI00133985 | RuvB-like 1 | 0 | 0 | 0 | 1 | 0 | 1.33 | 1.00E+00 |
| IPI00139364|IPI00606760|IPI00607076 | Serine/arginine-rich splicing factor 4 | 0 | 0 | 0 | 1 | 0 | 1.33 | 1.00E+00 |
| IPI00329913|IPI00757109|IPI00970652 | Isoform 1 of Protein-L-isoaspartate(D-aspartate) O-methyltransferase | 0 | 0 | 1 | 0 | 0 | 1.33 | 1.00E+00 |
| IPI00331644 | Proteasome subunit alpha type-3 | 0 | 0 | 1 | 0 | 0 | 1.33 | 1.00E+00 |
| IPI00269076|IPI00648318 | Isoform 2 of Adenylate kinase 2| mitochondrial | 0 | 0 | 0 | 2 | 0 | 1.67 | 1.00E+00 |
| IPI00162790|IPI00880213 | 60S ribosomal protein L18a | 0 | 0 | 0 | 1 | 0 | 1.33 | 1.00E+00 |
| IPI00457611|IPI00515330|IPI00648786 | Isoform 1 of Septin-9 | 0 | 0 | 0 | 2 | 0 | 1.67 | 1.00E+00 |
| IPI00136936|IPI00230513|IPI00881629|IPI00881968 | Isoform 1 of Vacuolar protein sorting-associated protein 29 | 0 | 0 | 0 | 1 | 0 | 1.33 | 1.00E+00 |
| IPI00321190|IPI00928070|IPI00928204|IPI00928284|IPI00928320|IPI00928581 | Sulfated glycoprotein 1 | 0 | 0 | 1 | 0 | 0 | 1.33 | 1.00E+00 |
| IPI00123458 | Cornifin-A | 0 | 0 | 0 | 1 | 0 | 1.33 | 1.00E+00 |
| IPI00266899 | Peptidyl-prolyl cis-trans isomerase FKBP1A | 0 | 0 | 2 | 0 | 0 | 1.67 | 1.00E+00 |
| IPI00114052 | Small nuclear ribonucleoprotein-associated protein B | 0 | 0 | 0 | 1 | 0 | 1.33 | 1.00E+00 |
| IPI00108774|IPI00975107 | UV excision repair protein RAD23 homolog B | 0 | 0 | 0 | 1 | 0 | 1.33 | 1.00E+00 |
| IPI00396797|IPI00653307 | Isoform 1 of Probable ATP-dependent RNA helicase DDX17 | 0 | 0 | 0 | 2 | 0 | 1.67 | 1.00E+00 |
| IPI00113394|IPI00230660|IPI00857457|IPI00985517|IPI00988085 | 40S ribosomal protein S15a-like | 0 | 0 | 1 | 0 | 0 | 1.33 | 1.00E+00 |
| IPI00120914 | Eukaryotic translation initiation factor 3 subunit F | 0 | 0 | 0 | 4 | 0 | 2.33 | 1.00E+00 |
| IPI00116356|IPI00622811 | AP-2 complex subunit mu | 0 | 0 | 1 | 0 | 0 | 1.33 | 1.00E+00 |
| IPI00399943 | Actin-related protein 2/3 complex subunit 5 | 0 | 0 | 1 | 0 | 0 | 1.33 | 1.00E+00 |
| IPI00918973 | Serine-threonine kinase receptor-associated protein | 0 | 0 | 0 | 0 | 2 | 1.67 | 1.00E+00 |
| IPI00352984 | Xanthine dehydrogenase/oxidase | 0 | 0 | 1 | 0 | 0 | 1.33 | 1.00E+00 |
| IPI00323800 | Neurofilament medium polypeptide | 0 | 0 | 1 | 0 | 0 | 1.33 | 1.00E+00 |
| IPI00468688 | Threonyl-tRNA synthetase| cytoplasmic | 0 | 0 | 0 | 3 | 0 | 2.00 | 1.00E+00 |
| IPI00122743 | Aspartyl-tRNA synthetase| cytoplasmic | 0 | 0 | 0 | 1 | 0 | 1.33 | 1.00E+00 |
| IPI00874728 | Isoform 2 of Tropomyosin beta chain | 0 | 0 | 0 | 0 | 16 | 6.33 | 1.00E+00 |
| IPI00109368 | Calmodulin-like protein 3 | 0 | 0 | 1 | 0 | 0 | 1.33 | 1.00E+00 |
| IPI00320399|IPI00466738|IPI00466817|IPI00623133 | Isoform Beta of Lamina-associated polypeptide 2| isoforms beta/delta/epsilon/gamma | 0 | 0 | 0 | 2 | 0 | 1.67 | 1.00E+00 |
| IPI00118499 | Ubiquitin carboxyl-terminal hydrolase isozyme L4 | 0 | 0 | 0 | 4 | 0 | 2.33 | 1.00E+00 |
| IPI00323235 | Lactotransferrin | 0 | 0 | 0 | 1 | 0 | 1.33 | 1.00E+00 |
| IPI00463468 | Nucleolar protein 58 | 0 | 0 | 0 | 1 | 0 | 1.33 | 1.00E+00 |
| IPI00118344 | UDP-glucose 6-dehydrogenase | 0 | 0 | 0 | 2 | 0 | 1.67 | 1.00E+00 |
| IPI00125899 | Catenin beta-1 | 0 | 0 | 0 | 2 | 0 | 1.67 | 1.00E+00 |
| IPI00990529 | 23 kDa protein | 0 | 0 | 0 | 1 | 0 | 1.33 | 1.00E+00 |
| IPI00223047 | Cytoskeleton-associated protein 4 | 0 | 0 | 0 | 1 | 0 | 1.33 | 1.00E+00 |
| IPI00621027 | Collagen alpha-2(VI) chain | 0 | 0 | 2 | 0 | 0 | 1.67 | 1.00E+00 |
| IPI00474446 | Eukaryotic translation initiation factor 2 subunit 1 | 0 | 0 | 0 | 2 | 0 | 1.67 | 1.00E+00 |
| IPI00132456|IPI00987013 | UPF0568 protein C14orf166 homolog | 0 | 0 | 1 | 0 | 0 | 1.33 | 1.00E+00 |
| IPI00123557 | RuvB-like 2 | 0 | 0 | 0 | 1 | 0 | 1.33 | 1.00E+00 |
| IPI00132460|IPI00880492 | 60S ribosomal protein L26 | 0 | 0 | 0 | 1 | 0 | 1.33 | 1.00E+00 |
| IPI00130381|IPI00277446|IPI00988370 | Large proline-rich protein BAG6 | 0 | 0 | 0 | 0 | 1 | 1.33 | 1.00E+00 |
| IPI00131259|IPI00224486|IPI00989544 | Galectin-3 | 0 | 0 | 0 | 2 | 0 | 1.67 | 1.00E+00 |
| IPI00323600 | Coronin-1A | 0 | 0 | 2 | 0 | 0 | 1.67 | 1.00E+00 |
| IPI00270326 | Putative uncharacterized protein | 0 | 0 | 0 | 2 | 0 | 1.67 | 1.00E+00 |
| IPI00125901|IPI00751092|IPI00989181 | 40S ribosomal protein S13 | 0 | 0 | 0 | 1 | 0 | 1.33 | 1.00E+00 |
| IPI00117569|IPI00762542 | Putative uncharacterized protein | 0 | 0 | 0 | 1 | 0 | 1.33 | 1.00E+00 |
| IPI00127172 | ATP-dependent RNA helicase DDX1 | 0 | 0 | 0 | 2 | 0 | 1.67 | 1.00E+00 |
| IPI00330063|IPI00653841|IPI00877182|IPI00987970 | F-actin-capping protein subunit alpha-1 | 0 | 0 | 0 | 1 | 0 | 1.33 | 1.00E+00 |
| IPI00469260|IPI00649950 | 116 kDa U5 small nuclear ribonucleoprotein component | 0 | 0 | 0 | 1 | 0 | 1.33 | 1.00E+00 |
| IPI00223415|IPI00918997 | Asparaginyl-tRNA synthetase| cytoplasmic | 0 | 0 | 0 | 2 | 0 | 1.67 | 1.00E+00 |
| IPI00331444 | Importin-7 | 0 | 0 | 0 | 1 | 0 | 1.33 | 1.00E+00 |
| IPI00116192 | Thioredoxin-dependent peroxide reductase| mitochondrial | 0 | 0 | 0 | 1 | 0 | 1.33 | 1.00E+00 |
| IPI00230145 | Ferritin heavy chain | 0 | 0 | 0 | 1 | 0 | 1.33 | 1.00E+00 |
| IPI00113845 | Proteasome subunit beta type-1 | 0 | 0 | 0 | 3 | 0 | 2.00 | 1.00E+00 |
| IPI00110684|IPI00988694 | Inorganic pyrophosphatase | 0 | 0 | 0 | 2 | 0 | 1.67 | 1.00E+00 |
| IPI00463573 | Eukaryotic translation initiation factor 3 subunit L | 0 | 0 | 0 | 1 | 0 | 1.33 | 1.00E+00 |
| IPI00123746 | Cadherin-13 | 0 | 0 | 3 | 0 | 0 | 2.00 | 1.00E+00 |
| IPI00224128 | Glycylpeptide N-tetradecanoyltransferase 1 | 0 | 0 | 0 | 2 | 0 | 1.67 | 1.00E+00 |
| IPI00850580|IPI00894870 | Isoform 2 of tRNA (cytosine(34)-C(5))-methyltransferase | 0 | 0 | 0 | 1 | 0 | 1.33 | 1.00E+00 |
| IPI00111265 | F-actin-capping protein subunit alpha-2 | 0 | 0 | 1 | 0 | 0 | 1.33 | 1.00E+00 |
| IPI00121515|IPI00757359|IPI00881332 | caprin-1 isoform c | 0 | 0 | 0 | 1 | 0 | 1.33 | 1.00E+00 |
| IPI00421206 | BTB/POZ domain-containing protein KCTD12 | 0 | 0 | 3 | 0 | 0 | 2.00 | 1.00E+00 |
| IPI00626752 | Mitotic checkpoint protein BUB3 | 0 | 0 | 0 | 0 | 1 | 1.33 | 1.00E+00 |
| IPI00113362 | Crk-like protein | 0 | 0 | 0 | 0 | 1 | 1.33 | 1.00E+00 |
| IPI00132089|IPI00989777 | Putative uncharacterized protein | 0 | 0 | 0 | 0 | 2 | 1.67 | 1.00E+00 |
| IPI00228583 | Myotrophin | 0 | 0 | 0 | 0 | 1 | 1.33 | 1.00E+00 |
| IPI00458105 | 60S ribosomal protein L3-like | 0 | 0 | 0 | 3 | 0 | 2.00 | 1.00E+00 |
| IPI00115862 | Eukaryotic translation initiation factor 6 | 0 | 0 | 0 | 2 | 0 | 1.67 | 1.00E+00 |
| IPI00943994 | Small nuclear ribonucleoprotein F | 0 | 0 | 0 | 1 | 0 | 1.33 | 1.00E+00 |
| IPI00126072 | Synaptic vesicle membrane protein VAT-1 homolog | 0 | 0 | 0 | 3 | 0 | 2.00 | 1.00E+00 |
| IPI00119945 | Omega-amidase NIT2 | 0 | 0 | 0 | 1 | 0 | 1.33 | 1.00E+00 |
| IPI00317902|IPI00875197 | Proteasome subunit beta type-5 | 0 | 0 | 0 | 0 | 1 | 1.33 | 1.00E+00 |
| IPI00623284 | Splicing factor 3B subunit 1 | 0 | 0 | 0 | 1 | 0 | 1.33 | 1.00E+00 |
| IPI00121013|IPI00830525 | Isoform 1 of Astrocytic phosphoprotein PEA-15 | 0 | 0 | 0 | 0 | 1 | 1.33 | 1.00E+00 |
| IPI00227814|IPI00228955|IPI00753792 | Isoform Stat3B of Signal transducer and activator of transcription 3 | 0 | 0 | 0 | 3 | 0 | 2.00 | 1.00E+00 |
| IPI00315548|IPI00404653|IPI00461504|IPI00462005|IPI00468385|IPI00555045|IPI00749628|IPI00875087|IPI00989154|IPI00989581|IPI00989761|IPI00990217|IPI00990363|IPI01023145 | 60S ribosomal protein L21 | 0 | 0 | 0 | 0 | 1 | 1.33 | 1.00E+00 |
| IPI00117087 | Hsp90 co-chaperone Cdc37 | 0 | 0 | 0 | 1 | 0 | 1.33 | 1.00E+00 |
| IPI00230507|IPI00623553 | ATP synthase subunit d| mitochondrial | 0 | 0 | 0 | 1 | 0 | 1.33 | 1.00E+00 |
| IPI00131086|IPI00653072|IPI00775798|IPI00776018|IPI00776053|IPI00986913|IPI00988761|IPI00990730 | Cytoplasmic dynein 1 intermediate chain 2 | 0 | 0 | 0 | 2 | 0 | 1.67 | 1.00E+00 |
| IPI00225390 | Cytochrome c oxidase subunit 6B1 | 0 | 0 | 0 | 1 | 0 | 1.33 | 1.00E+00 |
| IPI00134961 | Medium-chain specific acyl-CoA dehydrogenase| mitochondrial | 0 | 0 | 0 | 0 | 1 | 1.33 | 1.00E+00 |
| IPI00229859|IPI01008667 | Eif3b protein | 0 | 0 | 0 | 2 | 0 | 1.67 | 1.00E+00 |
| IPI00130591|IPI00403996|IPI00555078|IPI00776384 | Isoform 3 of Interleukin enhancer-binding factor 3 | 0 | 0 | 0 | 4 | 0 | 2.33 | 1.00E+00 |
| IPI00985680 | 17 kDa protein | 0 | 0 | 0 | 1 | 0 | 1.33 | 1.00E+00 |
| IPI00462140 | Keratin| type II cytoskeletal 1b | 0 | 0 | 3 | 0 | 0 | 2.00 | 1.00E+00 |
| IPI00330303 | Bifunctional purine biosynthesis protein PURH | 0 | 0 | 0 | 1 | 0 | 1.33 | 1.00E+00 |
| IPI00463297|IPI00474487|IPI00849174|IPI00869475|IPI00956939|IPI00986515|IPI00986933 | 60S ribosomal protein L36 | 0 | 0 | 2 | 0 | 0 | 1.67 | 1.00E+00 |
| IPI00353727 | Annexin A4 | 0 | 0 | 1 | 0 | 0 | 1.33 | 1.00E+00 |
| IPI00118143|IPI00121977 | Src substrate cortactin | 0 | 0 | 0 | 1 | 0 | 1.33 | 1.00E+00 |
| IPI00129178 | Ornithine aminotransferase| mitochondrial | 0 | 0 | 0 | 2 | 0 | 1.67 | 1.00E+00 |
| IPI00124709|IPI00173160|IPI00985620 | 40S ribosomal protein S27-like | 0 | 0 | 0 | 0 | 1 | 1.33 | 1.00E+00 |
| IPI00309035 | Dolichyl-diphosphooligosaccharide--protein glycosyltransferase subunit 1 | 0 | 0 | 0 | 1 | 0 | 1.33 | 1.00E+00 |
| IPI00313479|IPI00480458|IPI00648396|IPI00649251 | Isoform 2 of Integrin beta-4 | 0 | 0 | 0 | 0 | 3 | 2.00 | 1.00E+00 |
| IPI00123292|IPI00467635|IPI00874931 | Putative uncharacterized protein | 0 | 0 | 0 | 0 | 2 | 1.67 | 1.00E+00 |
| IPI00113696 | Metallothionein-1 | 0 | 0 | 0 | 1 | 0 | 1.33 | 1.00E+00 |
| IPI00556768|IPI00649283 | Thyroid hormone receptor-associated protein 3 | 0 | 0 | 0 | 1 | 0 | 1.33 | 1.00E+00 |
| IPI00132575 | Coactosin-like protein | 0 | 0 | 1 | 0 | 0 | 1.33 | 1.00E+00 |
| IPI00132347|IPI00555000 | Uncharacterized protein | 0 | 0 | 0 | 1 | 0 | 1.33 | 1.00E+00 |
| IPI00380895 | Myosin-3 | 0 | 0 | 30 | 0 | 0 | 11.00 | 1.00E+00 |
| IPI00124225|IPI00969956 | Proteasome activator complex subunit 2 | 0 | 0 | 0 | 2 | 0 | 1.67 | 1.00E+00 |
| IPI00125960 | Protein NDRG1 | 0 | 0 | 0 | 3 | 0 | 2.00 | 1.00E+00 |
| IPI00227516 | Protein FAM83H | 0 | 0 | 0 | 2 | 0 | 1.67 | 1.00E+00 |
| IPI00271951 | protein disulfide-isomerase A4 | 0 | 0 | 0 | 0 | 1 | 1.33 | 1.00E+00 |
| IPI00135464|IPI00850034 | Isoform 2 of Plakophilin-3 | 0 | 0 | 0 | 5 | 0 | 2.67 | 1.00E+00 |
| IPI00323806|IPI00762051|IPI00880689 | Putative uncharacterized protein | 0 | 0 | 0 | 1 | 0 | 1.33 | 1.00E+00 |
| IPI00828969|IPI00970046|IPI00970258|IPI00974601|IPI00988107 | Isoform 1 of PDZ and LIM domain protein 5 | 0 | 0 | 0 | 2 | 0 | 1.67 | 1.00E+00 |
| IPI00387232|IPI00399449|IPI00756894 | Isoform 3 of NSFL1 cofactor p47 | 0 | 0 | 0 | 2 | 0 | 1.67 | 1.00E+00 |
| IPI00132474 | Integrin beta-1 | 0 | 0 | 0 | 0 | 1 | 1.33 | 1.00E+00 |
| IPI00129276|IPI00986325 | Eukaryotic translation initiation factor 3 subunit A | 0 | 0 | 0 | 0 | 2 | 1.67 | 1.00E+00 |
| IPI00134017 | Cysteine and histidine-rich domain-containing protein 1 | 0 | 0 | 0 | 1 | 0 | 1.33 | 1.00E+00 |
| IPI00331321|IPI00555071|IPI00755220 | Uncharacterized protein | 0 | 0 | 0 | 1 | 0 | 1.33 | 1.00E+00 |
| IPI00266752 | Copine-3 | 0 | 0 | 0 | 2 | 0 | 1.67 | 1.00E+00 |
| IPI00314439 | 26S proteasome non-ATPase regulatory subunit 3 | 0 | 0 | 0 | 0 | 2 | 1.67 | 1.00E+00 |
| IPI00118766|IPI00465884 | Isoform 2 of Nuclear protein localization protein 4 homolog | 0 | 0 | 0 | 1 | 0 | 1.33 | 1.00E+00 |
| IPI00129323|IPI00221826 | Isoform Long of Serine/arginine-rich splicing factor 3 | 0 | 0 | 0 | 1 | 0 | 1.33 | 1.00E+00 |
| IPI00122399|IPI00990160 | Golgi apparatus protein 1 | 0 | 0 | 0 | 0 | 2 | 1.67 | 1.00E+00 |
| IPI00137229|IPI00330767|IPI00454151|IPI00869371 | Isoform 2 of Cellular nucleic acid-binding protein | 0 | 0 | 2 | 0 | 0 | 1.67 | 1.00E+00 |
| IPI00134131|IPI00648476 | Isoform SCPx of Non-specific lipid-transfer protein | 0 | 0 | 1 | 0 | 0 | 1.33 | 1.00E+00 |
| IPI00420329 | Uncharacterized protein | 0 | 0 | 0 | 2 | 0 | 1.67 | 1.00E+00 |
| IPI00338650|IPI00650026|IPI00918033|IPI00986711|IPI00987783 | Uncharacterized protein | 0 | 0 | 1 | 0 | 0 | 1.33 | 1.00E+00 |
| IPI00988328 | tubulin beta-3 chain-like | 0 | 0 | 0 | 0 | 2 | 1.67 | 1.00E+00 |
| IPI00119224 | Small nuclear ribonucleoprotein Sm D3 | 0 | 0 | 2 | 0 | 0 | 1.67 | 1.00E+00 |
| IPI00132208 | DnaJ homolog subfamily A member 1 | 0 | 0 | 0 | 0 | 1 | 1.33 | 1.00E+00 |
| IPI00315452|IPI00607023 | Purine nucleoside phosphorylase | 0 | 0 | 0 | 1 | 0 | 1.33 | 1.00E+00 |
| IPI00130343|IPI00223443|IPI00223444|IPI00759870|IPI00759886|IPI00874321 | Putative uncharacterized protein | 0 | 0 | 0 | 2 | 0 | 1.67 | 1.00E+00 |
| IPI00454049 | Enoyl-CoA hydratase| mitochondrial | 0 | 0 | 0 | 0 | 1 | 1.33 | 1.00E+00 |
| IPI00112223 | EF-hand domain-containing protein D2 | 0 | 0 | 2 | 0 | 0 | 1.67 | 1.00E+00 |
| IPI00122568 | Rho GDP-dissociation inhibitor 2 | 0 | 0 | 1 | 0 | 0 | 1.33 | 1.00E+00 |
| IPI00351252 | GMP synthase [glutamine-hydrolyzing] | 0 | 0 | 0 | 0 | 2 | 1.67 | 1.00E+00 |
| IPI00314467 | Proteasome subunit beta type-3 | 0 | 0 | 0 | 1 | 0 | 1.33 | 1.00E+00 |
| IPI00113746|IPI00990842 | Splicing factor U2AF 65 kDa subunit | 0 | 0 | 0 | 2 | 0 | 1.67 | 1.00E+00 |
| IPI00116134 | Dipeptidyl peptidase 3 | 0 | 0 | 0 | 1 | 0 | 1.33 | 1.00E+00 |
| IPI00113660 | Proteasome activator complex subunit 3 | 0 | 0 | 0 | 2 | 0 | 1.67 | 1.00E+00 |
| IPI00230212 | Glutathione S-transferase Mu 1 | 0 | 0 | 1 | 0 | 0 | 1.33 | 1.00E+00 |
| IPI00131224 | Transcription elongation factor B polypeptide 2 | 0 | 0 | 0 | 0 | 1 | 1.33 | 1.00E+00 |
| IPI00331734|IPI00555055|IPI00919098 | Histone H2A.Z | 0 | 0 | 0 | 4 | 0 | 2.33 | 1.00E+00 |
| IPI00322150 | Thimet oligopeptidase | 0 | 0 | 1 | 0 | 0 | 1.33 | 1.00E+00 |
| IPI00139788 | Serotransferrin | 0 | 0 | 1 | 0 | 0 | 1.33 | 1.00E+00 |
| IPI00404590|IPI00467914 | Putative uncharacterized protein | 0 | 0 | 0 | 0 | 2 | 1.67 | 1.00E+00 |
| IPI00322869 | ATP-binding cassette sub-family E member 1 | 0 | 0 | 0 | 0 | 1 | 1.33 | 1.00E+00 |
| IPI00113536|IPI00831258|IPI01008670 | Isoform 2 of Acidic leucine-rich nuclear phosphoprotein 32 family member B | 0 | 0 | 0 | 1 | 0 | 1.33 | 1.00E+00 |
| IPI00459443 | 182 kDa tankyrase-1-binding protein | 0 | 0 | 0 | 1 | 0 | 1.33 | 1.00E+00 |
| IPI00116308|IPI00989477 | Hsc70-interacting protein | 0 | 0 | 0 | 2 | 0 | 1.67 | 1.00E+00 |
| IPI00130794|IPI00458413 | Uncharacterized protein | 2 | 0 | 1 | 0 | 0 | 0.67 | 1.00E+00 |
| IPI00123891 | Cysteine and glycine-rich protein 1 | 2 | 0 | 1 | 0 | 0 | 0.67 | 1.00E+00 |
| IPI00338964|IPI00468900 | Isoform SERCA2B of Sarcoplasmic/endoplasmic reticulum calcium ATPase 2 | 0 | 13 | 0 | 0 | 8 | 0.49 | 1.00E+00 |
| IPI00116277 | T-complex protein 1 subunit delta | 0 | 1 | 0 | 1 | 2 | 1.33 | 1.00E+00 |
| IPI00127408|IPI00761613 | Uncharacterized protein | 1 | 0 | 1 | 0 | 2 | 1.33 | 1.00E+00 |
| IPI00111258|IPI00986977 | major vault protein | 0 | 1 | 0 | 2 | 1 | 1.33 | 1.00E+00 |
| IPI00121514 | Stress-induced-phosphoprotein 1 | 0 | 1 | 0 | 1 | 2 | 1.33 | 1.00E+00 |
| IPI00122426|IPI00648315 | 60S ribosomal protein L19 | 1 | 0 | 1 | 0 | 2 | 1.33 | 1.00E+00 |
| IPI00131186|IPI00515257 | Isoform 2 of Transcription factor BTF3 | 1 | 0 | 0 | 1 | 2 | 1.33 | 1.00E+00 |
| IPI00137730 | Phosphatidylethanolamine-binding protein 1 | 0 | 1 | 0 | 1 | 1 | 1.11 | 1.00E+00 |
| IPI00136251 | DnaJ homolog subfamily A member 2 | 1 | 0 | 1 | 0 | 0 | 0.89 | 1.00E+00 |
| IPI00124328 | Tropomodulin-4 | 0 | 1 | 0 | 0 | 1 | 0.89 | 1.00E+00 |
| IPI00116154|IPI00785410|IPI01008140 | cytochrome c oxidase subunit 5B| mitochondrial | 1 | 0 | 1 | 0 | 0 | 0.89 | 1.00E+00 |
| IPI00123129 | Staphylococcal nuclease domain-containing protein 1 | 1 | 0 | 0 | 1 | 1 | 1.11 | 1.00E+00 |
| IPI00118875|IPI00515654|IPI00928390|IPI00944009 | Isoform 1 of Elongation factor 1-delta | 0 | 1 | 1 | 0 | 0 | 0.89 | 1.00E+00 |
| IPI00127989|IPI00985716 | Prostaglandin E synthase 3 | 0 | 2 | 0 | 0 | 2 | 0.83 | 1.00E+00 |
| IPI00349401 | splicing factor 3b| subunit 2 | 1 | 0 | 0 | 1 | 0 | 0.89 | 1.00E+00 |
| IPI00121322|IPI00882287 | Electron transfer flavoprotein-ubiquinone oxidoreductase| mitochondrial | 1 | 0 | 1 | 0 | 0 | 0.89 | 1.00E+00 |
| IPI00127085|IPI00849927 | 60S ribosomal protein L10a | 1 | 0 | 0 | 0 | 1 | 0.89 | 1.00E+00 |
| IPI00133621 | Protein S100-A14 | 0 | 2 | 0 | 0 | 2 | 0.83 | 1.00E+00 |
| IPI00338295 | DNA polymerase epsilon catalytic subunit A | 0 | 1 | 1 | 0 | 0 | 0.89 | 1.00E+00 |
| IPI00114285 | Glutathione S-transferase omega-1 | 1 | 0 | 1 | 0 | 1 | 1.11 | 1.00E+00 |
| IPI00124149 | Nuclear transport factor 2 | 1 | 0 | 0 | 1 | 0 | 0.89 | 1.00E+00 |
| IPI00228385 | Glucose-6-phosphate 1-dehydrogenase X | 0 | 1 | 0 | 1 | 0 | 0.89 | 1.00E+00 |
| IPI00129512 | Proteasome subunit beta type-4 | 1 | 0 | 0 | 1 | 0 | 0.89 | 1.00E+00 |
| IPI00348328 | keratin Kb40 | 1 | 0 | 0 | 1 | 0 | 0.89 | 1.00E+00 |
| IPI00120508 | Myotilin | 2 | 0 | 1 | 0 | 1 | 0.83 | 1.00E+00 |
| IPI00466820|IPI00475203|IPI00971244 | 40S ribosomal protein S8 | 0 | 1 | 0 | 0 | 2 | 1.11 | 1.00E+00 |
| IPI00222208 | Heterogeneous nuclear ribonucleoprotein U-like protein 2 | 0 | 1 | 0 | 2 | 0 | 1.11 | 1.00E+00 |
| IPI00229834|IPI00928004|IPI00989407 | Coatomer subunit alpha | 1 | 0 | 0 | 2 | 0 | 1.11 | 1.00E+00 |
| IPI00221769 | GTP:AMP phosphotransferase| mitochondrial | 1 | 0 | 2 | 0 | 0 | 1.11 | 1.00E+00 |
| IPI00132250 | Eukaryotic translation initiation factor 3 subunit E | 1 | 0 | 0 | 2 | 0 | 1.11 | 1.00E+00 |
| IPI00133536|IPI00845541 | Glutathione peroxidase 3 | 1 | 0 | 2 | 0 | 0 | 1.11 | 1.00E+00 |
| IPI00894588 | long-chain specific acyl-CoA dehydrogenase| mitochondrial precursor | 1 | 0 | 2 | 0 | 0 | 1.11 | 1.00E+00 |
| IPI00314748|IPI00975201 | WD repeat-containing protein 1 | 0 | 1 | 0 | 2 | 0 | 1.11 | 1.00E+00 |
| IPI00123379 | Vigilin | 0 | 1 | 0 | 0 | 2 | 1.11 | 1.00E+00 |
| IPI00119581 | rRNA 2'-O-methyltransferase fibrillarin | 0 | 0 | 0 | 2 | 2 | 2.33 | 1.00E+00 |
| IPI00108895 | 26S protease regulatory subunit 6B | 0 | 0 | 0 | 2 | 2 | 2.33 | 1.00E+00 |
| IPI00132276 | Vesicle-associated membrane protein 3 | 0 | 0 | 0 | 2 | 2 | 2.33 | 1.00E+00 |
| IPI00321308 | Alanyl-tRNA synthetase| cytoplasmic | 0 | 0 | 0 | 2 | 2 | 2.33 | 1.00E+00 |
| IPI00138406|IPI00407954 | Ras-related protein Rap-1A | 0 | 0 | 0 | 2 | 2 | 2.33 | 1.00E+00 |
| IPI00466570 | Isoform 1 of Transmembrane emp24 domain-containing protein 10 | 0 | 0 | 0 | 2 | 2 | 2.33 | 1.00E+00 |
| IPI00135977 | Chloride intracellular channel protein 4 | 0 | 0 | 0 | 2 | 2 | 2.33 | 1.00E+00 |
| IPI00113257|IPI00402913|IPI00464127|IPI00670546|IPI00672748|IPI00918685|IPI00918921|IPI00919055 | Isoform 1 of Ubiquitin-conjugating enzyme E2 variant 1 | 0 | 0 | 0 | 2 | 2 | 2.33 | 1.00E+00 |
| IPI00227969|IPI00420835 | Isoform Alpha-6X1A of Integrin alpha-6 | 0 | 0 | 0 | 2 | 2 | 2.33 | 1.00E+00 |
| IPI00277930 | Putative uncharacterized protein | 0 | 0 | 0 | 2 | 2 | 2.33 | 1.00E+00 |
| IPI00125091|IPI00648086|IPI00648767|IPI00649088|IPI00649463|IPI00649913 | LIM and SH3 domain protein 1 | 0 | 0 | 0 | 2 | 2 | 2.33 | 1.00E+00 |
| IPI00139780|IPI00849782 | 60S ribosomal protein L23 | 0 | 1 | 0 | 2 | 2 | 1.56 | 1.00E+00 |
| IPI00462934 | Far upstream element-binding protein 2 | 0 | 0 | 0 | 3 | 3 | 3.00 | 1.00E+00 |
| IPI00130000 | Puromycin-sensitive aminopeptidase | 0 | 0 | 0 | 3 | 3 | 3.00 | 1.00E+00 |
| IPI00133428 | 26S protease regulatory subunit 4 | 2 | 0 | 0 | 2 | 3 | 1.33 | 1.00E+00 |
| IPI00119138 | Cytochrome b-c1 complex subunit 2| mitochondrial | 2 | 0 | 2 | 0 | 2 | 1.17 | 1.00E+00 |
| IPI00120374 | Serine/threonine-protein phosphatase 2A catalytic subunit alpha isoform | 2 | 0 | 0 | 2 | 2 | 1.17 | 1.00E+00 |
| IPI00230415|IPI00851014|IPI00988004 | Eukaryotic translation initiation factor 2 subunit 3| X-linked | 2 | 0 | 0 | 2 | 2 | 1.17 | 1.00E+00 |
| IPI00117288|IPI00277066|IPI00648228 | Heterogeneous nuclear ribonucleoprotein A/B | 0 | 2 | 0 | 2 | 0 | 0.83 | 1.00E+00 |
| IPI00331146 | UMP-CMP kinase | 0 | 2 | 0 | 2 | 0 | 0.83 | 1.00E+00 |
| IPI00113895 | Alpha-centractin | 0 | 2 | 0 | 2 | 0 | 0.83 | 1.00E+00 |
| IPI00113849 | Isoform 2 of Cell division control protein 42 homolog | 0 | 2 | 2 | 0 | 1 | 1.00 | 1.00E+00 |
| IPI00111412 | 60S ribosomal protein L4 | 0 | 3 | 0 | 3 | 2 | 1.07 | 1.00E+00 |
| IPI00229796 | Glycogen phosphorylase| brain form | 0 | 8 | 0 | 4 | 0 | 0.47 | 1.00E+00 |

**Table E**

| **Protein accession numbers** | **Protein name** | **Control1** | **Control2** | **Control3** | **Tumor1** | **Tumor2** | **Fold Change** | **Fisher** |
| --- | --- | --- | --- | --- | --- | --- | --- | --- |
| **List of proteins with the number of unique endogenous peptides found in both Human and Mouse Databases after search against Human database: CID** | | | | | | | | |
| IPI00217469 | Histone H1.1 | 12 | 10 | 6 | 5 | 0 | 0.34 | 3.56E-04 |
| IPI00418471 | Vimentin | 9 | 7 | 6 | 0 | 2 | 0.24 | 1.13E-03 |
| IPI00453473 | Histone H4 | 10 | 7 | 10 | 2 | 1 | 0.25 | 3.22E-03 |
| IPI00020101 | Histone H2B type 1-C/E/F/G/I | 11 | 8 | 8 | 4 | 1 | 0.35 | 4.71E-03 |
| IPI00002352 | Myosin regulatory light chain 2| skeletal muscle isoform | 9 | 7 | 1 | 2 | 1 | 0.38 | 4.88E-03 |
| IPI00217465 | Histone H1.2 | 10 | 15 | 10 | 4 | 4 | 0.39 | 5.19E-03 |
| IPI00216457 | Histone H2A type 2-A | 4 | 8 | 1 | 1 | 0 | 0.28 | 6.99E-03 |
| IPI00255316 | Histone H2A type 1-D | 6 | 4 | 3 | 1 | 0 | 0.28 | 1.52E-02 |
| IPI00550363|IPI00647915 | Transgelin-2 | 9 | 2 | 6 | 0 | 0 | 0.15 | 1.82E-02 |
| IPI00021439|IPI00021440 | Actin| cytoplasmic 1 | 4 | 3 | 0 | 0 | 0 | 0.30 | 2.86E-02 |
| IPI00013415 | 40S ribosomal protein S7 | 4 | 3 | 2 | 0 | 0 | 0.25 | 2.86E-02 |
| IPI00000494 | 60S ribosomal protein L5 | 3 | 4 | 2 | 0 | 0 | 0.25 | 2.86E-02 |
| IPI00012750 | 40S ribosomal protein S25 | 3 | 3 | 3 | 0 | 0 | 0.25 | 5.00E-02 |
| IPI00021266|IPI00789159|IPI00793523|IPI00794894 | Protein | 2 | 4 | 3 | 0 | 0 | 0.25 | 6.67E-02 |
| IPI00395998|IPI00927658 | 60S ribosomal protein L32 | 2 | 3 | 2 | 0 | 0 | 0.30 | 1.00E-01 |
| IPI00479217|IPI00644079|IPI00644224|IPI00883857 | cDNA FLJ44920 fis| clone BRAMY3011501| highly similar to Heterogeneous nuclear ribonucleoprotein U | 3 | 2 | 2 | 0 | 0 | 0.30 | 1.00E-01 |
| IPI00655650 | 40S ribosomal protein S26 | 4 | 4 | 0 | 1 | 1 | 0.55 | 1.03E-01 |
| IPI00021840 | 40S ribosomal protein S6 | 3 | 3 | 1 | 1 | 0 | 0.45 | 1.14E-01 |
| IPI00003918|IPI00795303 | 60S ribosomal protein L4 | 3 | 3 | 4 | 1 | 0 | 0.35 | 1.14E-01 |
| IPI00215965 | Isoform A1-B of Heterogeneous nuclear ribonucleoprotein A1 | 6 | 1 | 3 | 0 | 0 | 0.23 | 1.43E-01 |
| IPI00550239 | Histone H1.0 | 2 | 2 | 1 | 0 | 0 | 0.38 | 1.67E-01 |
| IPI00410714 | Hemoglobin subunit alpha | 2 | 2 | 3 | 0 | 0 | 0.30 | 1.67E-01 |
| IPI00026302 | 60S ribosomal protein L31 | 6 | 5 | 6 | 4 | 2 | 0.60 | 2.18E-01 |
| IPI00180240|IPI00220828|IPI00719405|IPI00815642|IPI00816288|IPI01011913|IPI01012854|IPI01013265|IPI01014272|IPI01015569 | Thymosin beta-4-like protein 3 | 3 | 1 | 1 | 0 | 0 | 0.38 | 2.50E-01 |
| IPI00414676|IPI00514027|IPI00555614 | Heat shock protein HSP 90-beta | 2 | 2 | 2 | 1 | 0 | 0.50 | 3.00E-01 |
| IPI00021428 | Actin| alpha skeletal muscle | 4 | 2 | 2 | 4 | 0 | 0.82 | 3.33E-01 |
| IPI00007611|IPI00878218|IPI00893479 | ATP synthase subunit O| mitochondrial | 1 | 2 | 0 | 0 | 0 | 0.50 | 3.33E-01 |
| IPI00334627|IPI00418169|IPI00455315|IPI00909509 | Putative annexin A2-like protein | 1 | 2 | 0 | 0 | 0 | 0.50 | 3.33E-01 |
| IPI00645948 | Uncharacterized protein | 2 | 1 | 1 | 0 | 0 | 0.43 | 3.33E-01 |
| IPI00306413 | Tubulin polymerization-promoting protein family member 3 | 1 | 2 | 2 | 1 | 0 | 0.56 | 5.00E-01 |
| IPI00031812|IPI00450235|IPI00643351 | Nuclease-sensitive element-binding protein 1 | 1 | 1 | 1 | 0 | 0 | 0.50 | 5.00E-01 |
| IPI00026964|IPI00889196 | Cytochrome b-c1 complex subunit Rieske| mitochondrial | 1 | 1 | 1 | 0 | 0 | 0.50 | 5.00E-01 |
| IPI00021263|IPI00789337|IPI00979946|IPI00981067|IPI00981450 | 14-3-3 protein zeta/delta | 1 | 1 | 1 | 0 | 0 | 0.50 | 5.00E-01 |
| IPI00300990|IPI00470438|IPI00644245|IPI00719040 | Isoform 1 of Chromatin target of PRMT1 protein | 1 | 1 | 1 | 0 | 0 | 0.50 | 5.00E-01 |
| IPI00382804|IPI00396485|IPI00472724|IPI00940393 | EEF1A protein (Fragment) | 1 | 1 | 1 | 0 | 0 | 0.50 | 5.00E-01 |
| IPI00023048|IPI00064086|IPI00642971|IPI00795920|IPI00985353|IPI01009986|IPI01013278|IPI01015965 | Isoform 2 of Elongation factor 1-delta | 1 | 1 | 1 | 0 | 0 | 0.50 | 5.00E-01 |
| IPI00025329 | 60S ribosomal protein L19 | 1 | 1 | 1 | 0 | 0 | 0.50 | 5.00E-01 |
| IPI00654777|IPI00941255|IPI01013021|IPI01013988 | cDNA FLJ36192 fis| clone TESTI2027450| highly similar to Eukaryotic translation initiation factor 3 subunit 5 | 1 | 1 | 1 | 0 | 0 | 0.50 | 5.00E-01 |
| IPI00221092 | 40S ribosomal protein S16 | 1 | 1 | 0 | 0 | 0 | 0.60 | 5.00E-01 |
| IPI00216318|IPI00759832 | Isoform Long of 14-3-3 protein beta/alpha | 1 | 1 | 0 | 0 | 0 | 0.60 | 5.00E-01 |
| IPI00479058 | 40S ribosomal protein S15 | 1 | 1 | 0 | 0 | 0 | 0.60 | 5.00E-01 |
| IPI00221093 | 40S ribosomal protein S17 | 1 | 1 | 0 | 0 | 0 | 0.60 | 5.00E-01 |
| IPI00025091 | 40S ribosomal protein S11 | 1 | 1 | 0 | 0 | 0 | 0.60 | 5.00E-01 |
| IPI00219155 | 60S ribosomal protein L27 | 1 | 1 | 0 | 0 | 0 | 0.60 | 5.00E-01 |
| IPI00006663|IPI00792207|IPI01021497|IPI01022431 | Aldehyde dehydrogenase| mitochondrial | 1 | 1 | 0 | 0 | 0 | 0.60 | 5.00E-01 |
| IPI00009550|IPI00983345 | Probable C->U-editing enzyme APOBEC-2 | 1 | 1 | 0 | 0 | 0 | 0.60 | 5.00E-01 |
| IPI00013485|IPI00979595 | 40S ribosomal protein S2 | 1 | 2 | 1 | 1 | 0 | 0.64 | 5.00E-01 |
| IPI00012772|IPI00976256|IPI00976899 | 60S ribosomal protein L8 | 1 | 1 | 0 | 1 | 0 | 0.90 | 6.67E-01 |
| IPI00973736|IPI00982652 | Uncharacterized protein | 1 | 1 | 1 | 1 | 0 | 0.75 | 6.67E-01 |
| IPI00017855|IPI00382844|IPI00790739|IPI00909879|IPI01009581|IPI01015355 | Aconitate hydratase| mitochondrial | 1 | 1 | 1 | 1 | 0 | 0.75 | 6.67E-01 |
| IPI00376005|IPI00411704|IPI00855924 | Isoform 2 of Eukaryotic translation initiation factor 5A-1 | 1 | 1 | 1 | 1 | 0 | 0.75 | 6.67E-01 |
| IPI00376798|IPI00746438 | Isoform 1 of 60S ribosomal protein L11 | 1 | 1 | 1 | 1 | 0 | 0.75 | 6.67E-01 |
| IPI00383598 | Cytochrome c oxidase polypeptide VIa | 1 | 1 | 1 | 1 | 0 | 0.75 | 6.67E-01 |
| IPI00219153 | 60S ribosomal protein L22 | 1 | 1 | 1 | 1 | 0 | 0.75 | 6.67E-01 |
| IPI00021405|IPI00216953 | Isoform A of Prelamin-A/C | 3 | 0 | 4 | 1 | 1 | 0.60 | 8.00E-01 |
| IPI00019884|IPI01010507 | Alpha-actinin-2 | 1 | 1 | 0 | 1 | 1 | 1.20 | 8.33E-01 |
| IPI00031691|IPI00967876|IPI00968128 | 60S ribosomal protein L9 | 1 | 1 | 1 | 1 | 1 | 1.00 | 8.33E-01 |
| IPI00396378|IPI00414696 | Isoform B1 of Heterogeneous nuclear ribonucleoproteins A2/B1 | 0 | 0 | 3 | 2 | 0 | 1.00 | 1.00E+00 |
| IPI00456429 | Ubiquitin-60S ribosomal protein L40 | 1 | 0 | 1 | 0 | 0 | 0.60 | 1.00E+00 |
| IPI00033494|IPI00220573|IPI00719669 | Myosin regulatory light chain 12B | 1 | 0 | 1 | 0 | 0 | 0.60 | 1.00E+00 |
| IPI00180956|IPI00240503|IPI00450768 | Uncharacterized protein | 1 | 0 | 1 | 0 | 0 | 0.60 | 1.00E+00 |
| IPI00221222|IPI00930562 | Activated RNA polymerase II transcriptional coactivator p15 | 1 | 0 | 1 | 0 | 0 | 0.60 | 1.00E+00 |
| IPI00218988|IPI00922165|IPI01021158 | Isoform 2 of Adenylate kinase 2| mitochondrial | 1 | 0 | 1 | 0 | 0 | 0.60 | 1.00E+00 |
| IPI00013296 | 40S ribosomal protein S18 | 1 | 0 | 1 | 0 | 0 | 0.60 | 1.00E+00 |
| IPI00414860 | 60S ribosomal protein L37a | 1 | 0 | 1 | 0 | 0 | 0.60 | 1.00E+00 |
| IPI00217030|IPI00646114 | 40S ribosomal protein S4| X isoform | 1 | 0 | 1 | 0 | 0 | 0.60 | 1.00E+00 |
| IPI00219160 | 60S ribosomal protein L34 | 1 | 0 | 2 | 0 | 0 | 0.50 | 1.00E+00 |
| IPI00299573 | 60S ribosomal protein L7a | 1 | 0 | 2 | 0 | 0 | 0.50 | 1.00E+00 |
| IPI00008438|IPI00749512 | 40S ribosomal protein S10 | 1 | 0 | 2 | 0 | 0 | 0.50 | 1.00E+00 |
| IPI00304612 | 60S ribosomal protein L13a | 2 | 0 | 1 | 0 | 0 | 0.50 | 1.00E+00 |
| IPI00217950 | Non-histone chromosomal protein HMG-17 | 1 | 0 | 2 | 0 | 0 | 0.50 | 1.00E+00 |
| IPI00220827 | Thymosin beta-10 | 2 | 0 | 1 | 0 | 0 | 0.50 | 1.00E+00 |
| IPI00465439|IPI00796333 | Fructose-bisphosphate aldolase A | 0 | 0 | 0 | 1 | 0 | 1.50 | 1.00E+00 |
| IPI00102685|IPI00385137|IPI00657979|IPI00658209|IPI00878612|IPI00910385 | Myeloid-associated differentiation marker | 0 | 0 | 0 | 1 | 0 | 1.50 | 1.00E+00 |
| IPI00440493|IPI00471928|IPI00908963 | ATP synthase subunit alpha| mitochondrial | 0 | 0 | 0 | 3 | 0 | 2.50 | 1.00E+00 |
| IPI00044631|IPI00477017 | Histone H2A-Bbd type 2/3 | 0 | 0 | 0 | 1 | 0 | 1.50 | 1.00E+00 |
| IPI00293276 | Macrophage migration inhibitory factor | 0 | 0 | 0 | 1 | 0 | 1.50 | 1.00E+00 |
| IPI00470528 | 60S ribosomal protein L15 | 0 | 0 | 0 | 1 | 0 | 1.50 | 1.00E+00 |
| IPI00018140|IPI00402183|IPI00402185|IPI00930205 | Isoform 1 of Heterogeneous nuclear ribonucleoprotein Q | 0 | 0 | 0 | 1 | 0 | 1.50 | 1.00E+00 |
| IPI00216456 | Histone H2A type 1-C | 0 | 0 | 0 | 0 | 1 | 1.50 | 1.00E+00 |
| IPI00009893|IPI00640755|IPI00978380|IPI00978830 | Gastric triacylglycerol lipase | 0 | 0 | 0 | 1 | 0 | 1.50 | 1.00E+00 |
| IPI00747053|IPI00917823 | TFIIH basal transcription factor complex helicase XPB subunit | 0 | 0 | 0 | 0 | 1 | 1.50 | 1.00E+00 |
| IPI00027397|IPI00607796|IPI00909195 | Isoform 1 of Hematological and neurological expressed 1-like protein | 0 | 0 | 1 | 0 | 0 | 0.75 | 1.00E+00 |
| IPI00014263 | Isoform Long of Eukaryotic translation initiation factor 4H | 0 | 0 | 1 | 0 | 0 | 0.75 | 1.00E+00 |
| IPI00216319 | 14-3-3 protein eta | 0 | 1 | 0 | 0 | 0 | 0.75 | 1.00E+00 |
| IPI00014587|IPI00216393|IPI00790571|IPI00945731|IPI00946844|IPI00975889|IPI00977178 | Isoform Brain of Clathrin light chain A | 0 | 0 | 1 | 0 | 0 | 0.75 | 1.00E+00 |
| IPI00220994|IPI01014852 | Core histone macro-H2A.2 | 0 | 0 | 1 | 0 | 0 | 0.75 | 1.00E+00 |
| IPI00014424 | Elongation factor 1-alpha 2 | 0 | 1 | 0 | 0 | 0 | 0.75 | 1.00E+00 |
| IPI00456887 | Heterogeneous nuclear ribonucleoprotein U-like protein 2 | 0 | 1 | 0 | 0 | 0 | 0.75 | 1.00E+00 |
| IPI00020557 | Prolow-density lipoprotein receptor-related protein 1 | 0 | 1 | 0 | 0 | 0 | 0.75 | 1.00E+00 |
| IPI00303335|IPI00743654|IPI00914847|IPI00917728|IPI01015244 | Nebulin | 0 | 0 | 1 | 0 | 0 | 0.75 | 1.00E+00 |
| IPI00032164 | Cysteine and glycine-rich protein 3 | 1 | 0 | 0 | 0 | 0 | 0.75 | 1.00E+00 |
| IPI00419373|IPI00455134|IPI00927677|IPI00939124 | Isoform 1 of Heterogeneous nuclear ribonucleoprotein A3 | 0 | 0 | 1 | 0 | 0 | 0.75 | 1.00E+00 |
| IPI00872947 | Similar to Plexin A2 | 0 | 0 | 1 | 0 | 0 | 0.75 | 1.00E+00 |
| IPI00023004|IPI00232533 | Eukaryotic translation initiation factor 1A| Y-chromosomal | 2 | 0 | 0 | 0 | 0 | 0.60 | 1.00E+00 |
| IPI00465361|IPI00978971 | 60S ribosomal protein L13 | 0 | 0 | 1 | 0 | 0 | 0.75 | 1.00E+00 |
| IPI00328840|IPI01010794 | THO complex subunit 4 | 1 | 0 | 0 | 0 | 0 | 0.75 | 1.00E+00 |
| IPI00384444 | Keratin| type I cytoskeletal 14 | 0 | 0 | 2 | 0 | 0 | 0.60 | 1.00E+00 |
| IPI00215611|IPI01021392|IPI01022132 | Cysteine-rich protein 1 | 0 | 1 | 0 | 0 | 0 | 0.75 | 1.00E+00 |
| IPI00013881|IPI00479191|IPI00980668 | Heterogeneous nuclear ribonucleoprotein H | 1 | 0 | 0 | 0 | 0 | 0.75 | 1.00E+00 |
| IPI00216322|IPI00216323|IPI00216324|IPI00216325|IPI00216326|IPI00216327|IPI00216328|IPI00216329|IPI00218951|IPI00218952|IPI00921926|IPI00939274|IPI01011552|IPI01011959 | Isoform 2 of Regulator of G-protein signaling 6 | 0 | 1 | 0 | 0 | 0 | 0.75 | 1.00E+00 |
| IPI00186290|IPI00909570|IPI01010856 | Elongation factor 2 | 0 | 1 | 0 | 0 | 0 | 0.75 | 1.00E+00 |
| IPI00216236|IPI00657955 | Troponin I| fast skeletal muscle | 0 | 0 | 1 | 0 | 0 | 0.75 | 1.00E+00 |
| IPI00022442 | Acyl carrier protein| mitochondrial | 0 | 1 | 0 | 0 | 0 | 0.75 | 1.00E+00 |
| IPI00030179|IPI00790291|IPI00794746 | 60S ribosomal protein L7 | 0 | 0 | 1 | 0 | 0 | 0.75 | 1.00E+00 |
| IPI00027463 | Protein S100-A6 | 0 | 0 | 1 | 0 | 0 | 0.75 | 1.00E+00 |
| IPI00220871 | 60S ribosomal protein L37 | 0 | 1 | 0 | 0 | 0 | 0.75 | 1.00E+00 |
| IPI00013991 | Isoform 1 of Tropomyosin beta chain | 0 | 1 | 0 | 0 | 0 | 0.75 | 1.00E+00 |
| IPI00013508|IPI00013808|IPI00759776|IPI00845465|IPI00909239|IPI00921118|IPI00942539|IPI01009456|IPI01011340|IPI01015738 | alpha-actinin-1 isoform a | 1 | 0 | 0 | 0 | 0 | 0.75 | 1.00E+00 |
| IPI00291764 | Histone H2A type 1 | 0 | 5 | 0 | 0 | 0 | 0.38 | 1.00E+00 |
| IPI00026260|IPI00029091|IPI00604590|IPI00795292|IPI00797082 | Isoform 1 of Nucleoside diphosphate kinase B | 0 | 1 | 0 | 0 | 0 | 0.75 | 1.00E+00 |
| IPI00018206|IPI00910267 | Aspartate aminotransferase| mitochondrial | 0 | 1 | 0 | 0 | 0 | 0.75 | 1.00E+00 |
| IPI00218606 | 40S ribosomal protein S23 | 1 | 0 | 0 | 0 | 0 | 0.75 | 1.00E+00 |
| IPI00411979|IPI00418767|IPI00847544|IPI01021503 | Isoform 3 of Formin-like protein 3 | 1 | 0 | 0 | 0 | 0 | 0.75 | 1.00E+00 |
| IPI00031523|IPI00382470|IPI00784295|IPI00910046 | Isoform 2 of Heat shock protein HSP 90-alpha | 0 | 0 | 1 | 0 | 0 | 0.75 | 1.00E+00 |
| IPI00215780 | 40S ribosomal protein S19 | 0 | 1 | 0 | 0 | 0 | 0.75 | 1.00E+00 |
| IPI00465294 | Cell division cycle 5-like protein | 0 | 0 | 1 | 0 | 0 | 0.75 | 1.00E+00 |
| IPI00030131|IPI00181409|IPI00216230|IPI00830089 | Isoform Beta of Lamina-associated polypeptide 2| isoforms beta/gamma | 1 | 0 | 0 | 0 | 0 | 0.75 | 1.00E+00 |
| IPI00410693|IPI00412714|IPI00470497|IPI00470498 | SERPINE1 mRNA binding protein 1| isoform CRA_d | 1 | 0 | 2 | 0 | 1 | 0.75 | 1.00E+00 |
| IPI00025092|IPI00384170|IPI00410261|IPI00412087|IPI00789615|IPI00797657|IPI00939358|IPI01015161|IPI01018657|IPI01020911|IPI01021665|IPI01022241|IPI01022367|IPI01022579|IPI01022710 | Isoform 1 of Myosin-binding protein C| slow-type | 0 | 0 | 1 | 1 | 0 | 1.13 | 1.00E+00 |
| IPI00015077 | Eukaryotic translation initiation factor 1 | 1 | 0 | 0 | 1 | 0 | 1.13 | 1.00E+00 |
| IPI00465084 | Desmin | 0 | 1 | 1 | 1 | 0 | 0.90 | 1.00E+00 |
| IPI00293434 | Signal recognition particle 14 kDa protein | 1 | 0 | 0 | 1 | 0 | 1.13 | 1.00E+00 |
| IPI00217468 | Histone H1.5 | 3 | 0 | 3 | 0 | 0 | 0.33 | 1.00E+00 |
| **List of proteins with the number of unique endogenous peptides found in both Human and Mouse Databases after search against Mouse database: CID** | | | | | | | | |
| IPI00223713 | Histone H1.2 | 11 | 14 | 0 | 4 | 0 | 0.32 | 3.95E-05 |
| IPI00227299 | Vimentin | 9 | 7 | 6 | 0 | 2 | 0.24 | 1.13E-03 |
| IPI00331597 | Histone H1.3 | 14 | 17 | 11 | 6 | 4 | 0.40 | 1.25E-03 |
| IPI00407339 | Histone H4 | 10 | 7 | 10 | 2 | 1 | 0.25 | 3.22E-03 |
| IPI00282266 | Histone H2B type 1-C/E/G | 11 | 8 | 8 | 4 | 1 | 0.35 | 4.71E-03 |
| IPI00224549 | Myosin regulatory light chain 2, skeletal muscle isoform | 9 | 7 | 1 | 2 | 1 | 0.38 | 4.88E-03 |
| IPI00330000,IPI00974916,IPI00989397 | Histone H2A type 2-A | 4 | 8 | 1 | 1 | 0 | 0.28 | 6.99E-03 |
| IPI00125778 | Transgelin-2 | 9 | 2 | 6 | 0 | 0 | 0.15 | 1.82E-02 |
| IPI00136984,IPI00850934 | 40S ribosomal protein S7 | 4 | 3 | 2 | 0 | 0 | 0.25 | 2.86E-02 |
| IPI00308706,IPI00988023 | 60S ribosomal protein L5 | 3 | 4 | 2 | 0 | 0 | 0.25 | 2.86E-02 |
| IPI00110850,IPI00874482 | Actin, cytoplasmic 1 | 4 | 3 | 0 | 0 | 0 | 0.30 | 2.86E-02 |
| IPI00137735 | 40S ribosomal protein S25 | 3 | 3 | 3 | 0 | 0 | 0.25 | 5.00E-02 |
| IPI00461456,IPI00849847 | 60S ribosomal protein L23a | 2 | 4 | 3 | 0 | 0 | 0.25 | 6.67E-02 |
| IPI00230623 | 60S ribosomal protein L32 | 2 | 3 | 2 | 0 | 0 | 0.30 | 1.00E-01 |
| IPI00458583 | Heterogeneous nuclear ribonucleoprotein U | 3 | 2 | 2 | 0 | 0 | 0.30 | 1.00E-01 |
| IPI00228616 | Histone H1.1 | 2 | 3 | 2 | 0 | 0 | 0.30 | 1.00E-01 |
| IPI00377441 | 40S ribosomal protein S26 | 4 | 4 | 0 | 1 | 1 | 0.55 | 1.03E-01 |
| IPI00108454,IPI00113655,IPI00990093 | 40S ribosomal protein S6 | 3 | 3 | 1 | 1 | 0 | 0.45 | 1.14E-01 |
| IPI00111412 | 60S ribosomal protein L4 | 3 | 3 | 4 | 1 | 0 | 0.35 | 1.14E-01 |
| IPI00553777,IPI00817004 | Putative uncharacterized protein | 6 | 1 | 3 | 0 | 0 | 0.23 | 1.43E-01 |
| IPI00404590,IPI00467914 | Putative uncharacterized protein | 2 | 2 | 1 | 0 | 0 | 0.38 | 1.67E-01 |
| IPI00469114 | Hemoglobin subunit alpha | 2 | 2 | 3 | 0 | 0 | 0.30 | 1.67E-01 |
| IPI00123007 | 60S ribosomal protein L31 | 6 | 5 | 6 | 4 | 2 | 0.60 | 2.18E-01 |
| IPI00228757,IPI00853896 | Isoform Short of Thymosin beta-4 | 3 | 1 | 1 | 0 | 0 | 0.38 | 2.50E-01 |
| IPI00221463,IPI00265761 | Histone H2A type 3 | 4 | 3 | 2 | 2 | 1 | 0.63 | 2.62E-01 |
| IPI00554929,IPI00830562,IPI00989376,IPI01016123 | Heat shock protein HSP 90-beta | 2 | 2 | 2 | 1 | 0 | 0.50 | 3.00E-01 |
| IPI00110827 | Actin, alpha skeletal muscle | 4 | 2 | 2 | 4 | 0 | 0.82 | 3.33E-01 |
| IPI00468203,IPI00885455 | Annexin A2 | 1 | 2 | 0 | 0 | 0 | 0.50 | 3.33E-01 |
| IPI00118986 | ATP synthase subunit O, mitochondrial | 1 | 2 | 0 | 0 | 0 | 0.50 | 3.33E-01 |
| IPI00420261,IPI00665601 | High mobility group protein B1 | 2 | 1 | 1 | 0 | 0 | 0.43 | 3.33E-01 |
| IPI00307837 | Elongation factor 1-alpha 1 | 1 | 1 | 1 | 0 | 0 | 0.50 | 5.00E-01 |
| IPI00230682,IPI00760000 | Isoform Long of 14-3-3 protein beta/alpha | 1 | 1 | 0 | 0 | 0 | 0.60 | 5.00E-01 |
| IPI00120886,IPI00647981 | Nuclease-sensitive element-binding protein 1 | 1 | 1 | 1 | 0 | 0 | 0.50 | 5.00E-01 |
| IPI00468771,IPI00515654,IPI00928179,IPI01023296 | Uncharacterized protein | 1 | 1 | 1 | 0 | 0 | 0.50 | 5.00E-01 |
| IPI00380130,IPI00605141,IPI00606508,IPI00987518,IPI00988101,IPI00988545 | Putative uncharacterized protein | 1 | 2 | 1 | 1 | 0 | 0.64 | 5.00E-01 |
| IPI00319231,IPI00850964 | 40S ribosomal protein S15 | 1 | 1 | 0 | 0 | 0 | 0.60 | 5.00E-01 |
| IPI00120914 | Eukaryotic translation initiation factor 3 subunit F | 1 | 1 | 1 | 0 | 0 | 0.50 | 5.00E-01 |
| IPI00111218 | Aldehyde dehydrogenase, mitochondrial | 1 | 1 | 0 | 0 | 0 | 0.60 | 5.00E-01 |
| IPI00122426,IPI00648315 | 60S ribosomal protein L19 | 1 | 1 | 1 | 0 | 0 | 0.50 | 5.00E-01 |
| IPI00120174,IPI00283671,IPI00469107,IPI00471466,IPI00471467,IPI00830185 | Isoform 2 of Friend of PRMT1 protein | 1 | 1 | 1 | 0 | 0 | 0.50 | 5.00E-01 |
| IPI00122421 | 60S ribosomal protein L27 | 1 | 1 | 0 | 0 | 0 | 0.60 | 5.00E-01 |
| IPI00465880,IPI00985790 | 40S ribosomal protein S17 | 1 | 1 | 0 | 0 | 0 | 0.60 | 5.00E-01 |
| IPI00125150 | Probable C->U-editing enzyme APOBEC-2 | 1 | 1 | 0 | 0 | 0 | 0.60 | 5.00E-01 |
| IPI00762542 | 40S ribosomal protein S11 | 1 | 1 | 0 | 0 | 0 | 0.60 | 5.00E-01 |
| IPI00469918,IPI00985889 | Rps16 protein | 1 | 1 | 0 | 0 | 0 | 0.60 | 5.00E-01 |
| IPI00133557 | Tubulin polymerization-promoting protein family member 3 | 1 | 2 | 2 | 1 | 0 | 0.56 | 5.00E-01 |
| IPI00116498 | 14-3-3 protein zeta/delta | 1 | 1 | 1 | 0 | 0 | 0.50 | 5.00E-01 |
| IPI00133240 | Cytochrome b-c1 complex subunit Rieske, mitochondrial | 1 | 1 | 1 | 0 | 0 | 0.50 | 5.00E-01 |
| IPI00108125 | Eukaryotic translation initiation factor 5A-1 | 1 | 1 | 1 | 1 | 0 | 0.75 | 6.67E-01 |
| IPI00116074 | Aconitate hydratase, mitochondrial | 1 | 1 | 1 | 1 | 0 | 0.75 | 6.67E-01 |
| IPI00121443 | Cytochrome c oxidase polypeptide VIa | 1 | 1 | 1 | 1 | 0 | 0.75 | 6.67E-01 |
| IPI00118707,IPI00331461,IPI00340036,IPI00474856,IPI00624735,IPI00989506 | Uncharacterized protein | 1 | 1 | 1 | 1 | 0 | 0.75 | 6.67E-01 |
| IPI00222546 | 60S ribosomal protein L22 | 1 | 1 | 1 | 1 | 0 | 0.75 | 6.67E-01 |
| IPI00399483 | 40S ribosomal protein S30 | 1 | 1 | 1 | 1 | 0 | 0.75 | 6.67E-01 |
| IPI00137787 | 60S ribosomal protein L8 | 1 | 1 | 0 | 1 | 0 | 0.90 | 6.67E-01 |
| IPI00620256 | Isoform A of Prelamin-A/C | 3 | 0 | 4 | 1 | 1 | 0.60 | 8.00E-01 |
| IPI00331664,IPI00387557 | Alpha-actinin-2 | 1 | 1 | 0 | 1 | 1 | 1.20 | 8.33E-01 |
| IPI00122413,IPI00407917,IPI00990485 | 60S ribosomal protein L9 | 1 | 1 | 1 | 1 | 1 | 1.00 | 8.33E-01 |
| IPI00652934,IPI00987032,IPI00987386 | Histone H2A | 0 | 0 | 1 | 0 | 0 | 0.75 | 1.00E+00 |
| IPI00107958,IPI00225969,IPI00396784 | Hematological and neurological expressed 1-like protein | 0 | 0 | 1 | 0 | 0 | 0.75 | 1.00E+00 |
| IPI00137313 | Plexin-A2 | 0 | 0 | 1 | 0 | 0 | 0.75 | 1.00E+00 |
| IPI00284444,IPI00988498 | Cell division cycle 5-related protein | 0 | 0 | 1 | 0 | 0 | 0.75 | 1.00E+00 |
| IPI00269661,IPI00269662,IPI00626666,IPI00667787,IPI00751584,IPI00988230,IPI00990072 | Isoform 1 of Heterogeneous nuclear ribonucleoprotein A3 | 0 | 0 | 1 | 0 | 0 | 0.75 | 1.00E+00 |
| IPI00330363,IPI00462453,IPI01018378 | 60S ribosomal protein L7a | 1 | 0 | 2 | 0 | 0 | 0.50 | 1.00E+00 |
| IPI00113070 | TFIIH basal transcription factor complex helicase XPB subunit | 0 | 0 | 0 | 0 | 1 | 1.50 | 1.00E+00 |
| IPI00124742,IPI00222560 | Isoform Long of Eukaryotic translation initiation factor 4H | 0 | 0 | 1 | 0 | 0 | 0.75 | 1.00E+00 |
| IPI00269076 | Isoform 2 of Adenylate kinase 2, mitochondrial | 1 | 0 | 1 | 0 | 0 | 0.60 | 1.00E+00 |
| IPI00265467,IPI00653348 | Regulator of G-protein signaling 6 | 0 | 1 | 0 | 0 | 0 | 0.75 | 1.00E+00 |
| IPI00153400 | Histone H2A.J | 0 | 4 | 0 | 0 | 0 | 0.43 | 1.00E+00 |
| IPI00230133 | Histone H1.5 | 5 | 0 | 3 | 0 | 0 | 0.27 | 1.00E+00 |
| IPI00117583 | Thymosin beta-10 | 2 | 0 | 1 | 0 | 0 | 0.50 | 1.00E+00 |
| IPI00121427 | Protein S100-A6 | 0 | 0 | 1 | 0 | 0 | 0.75 | 1.00E+00 |
| IPI00112448 | 40S ribosomal protein S10 | 1 | 0 | 2 | 0 | 0 | 0.50 | 1.00E+00 |
| IPI00123319 | Isoform 1 of Tropomyosin beta chain | 0 | 1 | 0 | 0 | 0 | 0.75 | 1.00E+00 |
| IPI00130102 | Desmin | 0 | 1 | 1 | 1 | 0 | 0.90 | 1.00E+00 |
| IPI00114409,IPI00648658,IPI00648681,IPI00648927,IPI00649453,IPI00828932,IPI00876257 | clathrin light chain A isoform c | 0 | 0 | 1 | 0 | 0 | 0.75 | 1.00E+00 |
| IPI00118899,IPI00380436,IPI00989903 | Alpha-actinin-4 | 1 | 0 | 0 | 0 | 0 | 0.75 | 1.00E+00 |
| IPI00330804 | Heat shock protein HSP 90-alpha | 0 | 0 | 1 | 0 | 0 | 0.75 | 1.00E+00 |
| IPI00227140 | Keratin, type I cytoskeletal 14 | 0 | 0 | 2 | 0 | 0 | 0.60 | 1.00E+00 |
| IPI00230427 | Macrophage migration inhibitory factor | 0 | 0 | 0 | 1 | 0 | 1.50 | 1.00E+00 |
| IPI00131357,IPI00972920 | 40S ribosomal protein S23 | 1 | 0 | 0 | 0 | 0 | 0.75 | 1.00E+00 |
| IPI00137930,IPI00677780,IPI00761998,IPI00850655 | eukaryotic translation initiation factor 1 | 1 | 0 | 0 | 1 | 0 | 1.13 | 1.00E+00 |
| IPI00134097,IPI00224505 | 60S ribosomal protein L13 | 0 | 0 | 1 | 0 | 0 | 0.75 | 1.00E+00 |
| IPI00460103,IPI00461652,IPI00466153,IPI00621625 | 60S ribosomal protein L34 | 1 | 0 | 2 | 0 | 0 | 0.50 | 1.00E+00 |
| IPI00132418,IPI00756504 | Signal recognition particle 14 kDa protein | 1 | 0 | 0 | 1 | 0 | 1.13 | 1.00E+00 |
| IPI00127417,IPI00408489,IPI00990246 | Down syndrome cell adhesion molecule-like protein (Fragment) | 0 | 1 | 0 | 0 | 0 | 0.75 | 1.00E+00 |
| IPI00775948 | Uncharacterized protein | 0 | 0 | 1 | 0 | 0 | 0.75 | 1.00E+00 |
| IPI00471475,IPI00471476,IPI00471477,IPI00884509 | Isoform 1 of Plasminogen activator inhibitor 1 RNA-binding protein | 1 | 0 | 2 | 0 | 1 | 0.75 | 1.00E+00 |
| IPI00222208 | Heterogeneous nuclear ribonucleoprotein U-like protein 2 | 0 | 1 | 0 | 0 | 0 | 0.75 | 1.00E+00 |
| IPI00130280,IPI00857439 | ATP synthase subunit alpha, mitochondrial | 0 | 0 | 0 | 3 | 0 | 2.50 | 1.00E+00 |
| IPI00113241,IPI00665513,IPI00856352,IPI00856927,IPI00857038,IPI00857391,IPI00875584,IPI00875929,IPI00990110 | 40S ribosomal protein S19 | 0 | 1 | 0 | 0 | 0 | 0.75 | 1.00E+00 |
| IPI00119063 | Prolow-density lipoprotein receptor-related protein 1 | 0 | 1 | 0 | 0 | 0 | 0.75 | 1.00E+00 |
| IPI00109044,IPI00132705,IPI00758006 | myosin light chain, regulatory B-like | 1 | 0 | 1 | 0 | 0 | 0.60 | 1.00E+00 |
| IPI00466069 | Elongation factor 2 | 0 | 1 | 0 | 0 | 0 | 0.75 | 1.00E+00 |
| IPI00126338,IPI00320399,IPI00466738,IPI00466817,IPI00623133,IPI00798595 | Isoform Alpha of Lamina-associated polypeptide 2, isoforms alpha/zeta | 1 | 0 | 0 | 0 | 0 | 0.75 | 1.00E+00 |
| IPI00273803,IPI00473532,IPI00850320 | 60S ribosomal protein L15 | 0 | 0 | 0 | 1 | 0 | 1.50 | 1.00E+00 |
| IPI00153608,IPI00308984 | Eukaryotic translation initiation factor 1A | 2 | 0 | 0 | 0 | 0 | 0.60 | 1.00E+00 |
| IPI00133006,IPI00918079,IPI00918301,IPI00987889 | Acyl carrier protein, mitochondrial | 0 | 1 | 0 | 0 | 0 | 0.75 | 1.00E+00 |
| IPI00396774,IPI00895129 | myosin binding protein C, slow type | 0 | 0 | 1 | 1 | 0 | 1.13 | 1.00E+00 |
| IPI00230365 | Keratin, type I cytoskeletal 17 | 1 | 0 | 1 | 0 | 0 | 0.60 | 1.00E+00 |
| IPI00338650,IPI00987783,IPI00990954 | Non-histone chromosomal protein HMG-17 | 1 | 0 | 2 | 0 | 0 | 0.50 | 1.00E+00 |
| IPI00379424,IPI00751023 | Uncharacterized protein | 0 | 0 | 1 | 0 | 0 | 0.75 | 1.00E+00 |
| IPI00227392 | 14-3-3 protein eta | 0 | 1 | 0 | 0 | 0 | 0.75 | 1.00E+00 |
| IPI00420509,IPI00467225,IPI00652582,IPI00882164 | Isoform 1 of Formin-like protein 3 | 1 | 0 | 0 | 0 | 0 | 0.75 | 1.00E+00 |
| IPI00331092,IPI00990327 | 40S ribosomal protein S4, X isoform | 1 | 0 | 1 | 0 | 0 | 0.60 | 1.00E+00 |
| IPI00117312 | Aspartate aminotransferase, mitochondrial | 0 | 1 | 0 | 0 | 0 | 0.75 | 1.00E+00 |
| IPI00133916,IPI00224729 | Uncharacterized protein | 1 | 0 | 0 | 0 | 0 | 0.75 | 1.00E+00 |
| IPI00262488 | Cysteine-rich protein 1 | 0 | 1 | 0 | 0 | 0 | 0.75 | 1.00E+00 |
| IPI00453924 | 60S ribosomal protein L37 | 0 | 1 | 0 | 0 | 0 | 0.75 | 1.00E+00 |
| IPI00114407 | Isoform 1 of THO complex subunit 4 | 1 | 0 | 0 | 0 | 0 | 0.75 | 1.00E+00 |
| IPI00222550 | Putative uncharacterized protein | 1 | 0 | 1 | 0 | 0 | 0.60 | 1.00E+00 |
| IPI00223196,IPI00830423 | Troponin I, fast skeletal muscle | 0 | 0 | 1 | 0 | 0 | 0.75 | 1.00E+00 |
| IPI00406117,IPI00406118 | Isoform 1 of Heterogeneous nuclear ribonucleoprotein Q | 0 | 0 | 0 | 1 | 0 | 1.50 | 1.00E+00 |
| IPI00225633 | Activated RNA polymerase II transcriptional coactivator p15 | 1 | 0 | 1 | 0 | 0 | 0.60 | 1.00E+00 |
| IPI00132938 | Myeloid-associated differentiation marker | 0 | 0 | 0 | 1 | 0 | 1.50 | 1.00E+00 |
| IPI00118153 | Cysteine and glycine-rich protein 3 | 1 | 0 | 0 | 0 | 0 | 0.75 | 1.00E+00 |
| IPI00221402,IPI00856379 | Fructose-bisphosphate aldolase A | 0 | 0 | 0 | 1 | 0 | 1.50 | 1.00E+00 |
| IPI00405058,IPI00622847,IPI00828488,IPI01023279 | Isoform 3 of Heterogeneous nuclear ribonucleoproteins A2/B1 | 0 | 0 | 3 | 2 | 0 | 1.00 | 1.00E+00 |
| IPI00317590,IPI00620156,IPI00990188,IPI01019245 | 40S ribosomal protein S18 | 1 | 0 | 1 | 0 | 0 | 0.60 | 1.00E+00 |
| IPI00131735 | Gastric triacylglycerol lipase | 0 | 0 | 0 | 1 | 0 | 1.50 | 1.00E+00 |
| IPI00138892,IPI00621468,IPI00895319,IPI00895479,IPI00918763,IPI00987848 | ubiquitin-like protein ISG15-like | 1 | 0 | 1 | 0 | 0 | 0.60 | 1.00E+00 |
| IPI00919200 | Uncharacterized protein | 2 | 0 | 1 | 0 | 0 | 0.50 | 1.00E+00 |
| IPI00119667 | Elongation factor 1-alpha 2 | 0 | 1 | 0 | 0 | 0 | 0.75 | 1.00E+00 |

**Table F**

| **Protein accession numbers** | **Protein name** | **Control1** | **Control2** | **Control3** | **Tumor1** | **Tumor2** | **Fold Change** | **Fisher** |
| --- | --- | --- | --- | --- | --- | --- | --- | --- |
| **List of proteins with the number of unique endogenous peptides found in both Human and Mouse Databases after search against Human database: HCD** | | | | | | | | |
| IPI00217465|IPI00217466|IPI00217467 | Histone H1.2 | 11 | 17 | 10 | 1 | 1 | 0.15 | 2.51E-06 |
| IPI00453473 | Histone H4 | 12 | 11 | 6 | 2 | 2 | 0.28 | 3.63E-04 |
| IPI00217469 | Histone H1.1 | 7 | 10 | 7 | 2 | 1 | 0.28 | 3.22E-03 |
| IPI00003918|IPI00795303 | 60S ribosomal protein L4 | 4 | 6 | 2 | 0 | 0 | 0.20 | 4.76E-03 |
| IPI00217468 | Histone H1.5 | 4 | 5 | 0 | 0 | 0 | 0.25 | 7.94E-03 |
| IPI00419833 | Histone H2B type 1-K | 5 | 7 | 1 | 2 | 1 | 0.47 | 3.50E-02 |
| IPI00026272|IPI00031562 | Histone H2A type 1-B/E | 0 | 0 | 0 | 4 | 2 | 4.00 | 6.67E-02 |
| IPI00021439|IPI00021440 | Actin| cytoplasmic 1 | 3 | 2 | 0 | 0 | 0 | 0.38 | 1.00E-01 |
| IPI00215965|IPI00465365|IPI00797148|IPI01021093|IPI01021324|IPI01022060|IPI01022801 | Isoform A1-B of Heterogeneous nuclear ribonucleoprotein A1 | 2 | 3 | 0 | 0 | 0 | 0.38 | 1.00E-01 |
| IPI00029750|IPI00847986|IPI00903204|IPI00915363|IPI00915463 | Isoform 1 of 40S ribosomal protein S24 | 2 | 3 | 1 | 0 | 0 | 0.33 | 1.00E-01 |
| IPI00410714|IPI00853068 | Hemoglobin subunit alpha | 3 | 2 | 1 | 0 | 0 | 0.33 | 1.00E-01 |
| IPI00021266|IPI00789159|IPI00793523|IPI00794894 | 60S ribosomal protein L23a | 2 | 3 | 1 | 0 | 0 | 0.33 | 1.00E-01 |
| IPI00021428|IPI00414057 | Actin| alpha skeletal muscle | 2 | 2 | 0 | 0 | 0 | 0.43 | 1.67E-01 |
| IPI00013415 | 40S ribosomal protein S7 | 2 | 2 | 1 | 0 | 0 | 0.38 | 1.67E-01 |
| IPI00020101 | Histone H2B type 1-C/E/F/G/I | 0 | 0 | 0 | 3 | 1 | 3.00 | 2.50E-01 |
| IPI00025329 | 60S ribosomal protein L19 | 1 | 2 | 0 | 0 | 0 | 0.50 | 3.33E-01 |
| IPI00025091 | 40S ribosomal protein S11 | 1 | 2 | 0 | 0 | 0 | 0.50 | 3.33E-01 |
| IPI00255316 | Histone H2A type 1-D | 4 | 4 | 2 | 4 | 2 | 0.92 | 4.71E-01 |
| IPI00304612 | 60S ribosomal protein L13a | 1 | 1 | 0 | 0 | 0 | 0.60 | 5.00E-01 |
| IPI00218606 | 40S ribosomal protein S23 | 1 | 1 | 0 | 0 | 0 | 0.60 | 5.00E-01 |
| IPI00021840 | 40S ribosomal protein S6 | 1 | 2 | 1 | 1 | 0 | 0.64 | 5.00E-01 |
| IPI00026302 | 60S ribosomal protein L31 | 1 | 1 | 1 | 0 | 1 | 0.75 | 6.67E-01 |
| IPI00550239 | Histone H1.0 | 1 | 1 | 1 | 1 | 1 | 1.00 | 8.33E-01 |
| IPI00012772|IPI00909884 | 60S ribosomal protein L8 | 0 | 1 | 1 | 0 | 0 | 0.60 | 1.00E+00 |
| IPI00000494 | 60S ribosomal protein L5 | 0 | 1 | 1 | 0 | 0 | 0.60 | 1.00E+00 |
| IPI00219155 | 60S ribosomal protein L27 | 0 | 1 | 1 | 0 | 0 | 0.60 | 1.00E+00 |
| IPI00299573 | 60S ribosomal protein L7a | 0 | 2 | 1 | 0 | 0 | 0.50 | 1.00E+00 |
| IPI00216457 | Histone H2A type 2-A | 0 | 4 | 2 | 0 | 0 | 0.33 | 1.00E+00 |
| IPI00013485|IPI00871956|IPI00979595 | 40S ribosomal protein S2 | 0 | 0 | 0 | 1 | 0 | 1.50 | 1.00E+00 |
| IPI00550363|IPI00647915 | Transgelin-2 | 0 | 2 | 0 | 0 | 0 | 0.60 | 1.00E+00 |
| IPI00030179|IPI00794746 | 60S ribosomal protein L7 | 0 | 1 | 0 | 0 | 0 | 0.75 | 1.00E+00 |
| IPI00217950 | Non-histone chromosomal protein HMG-17 | 0 | 3 | 0 | 0 | 0 | 0.50 | 1.00E+00 |
| IPI00002352 | Myosin regulatory light chain 2| skeletal muscle isoform | 0 | 5 | 0 | 0 | 0 | 0.38 | 1.00E+00 |
| IPI00021405|IPI00216953|IPI00514320|IPI00910241|IPI00979923 | Isoform A of Prelamin-A/C | 2 | 0 | 0 | 0 | 0 | 0.60 | 1.00E+00 |
| IPI00479217|IPI00644079|IPI00644224|IPI00883857 | Isoform Short of Heterogeneous nuclear ribonucleoprotein U | 0 | 0 | 1 | 0 | 0 | 0.75 | 1.00E+00 |
| IPI00220827 | Thymosin beta-10 | 0 | 1 | 0 | 0 | 0 | 0.75 | 1.00E+00 |
| IPI00032220|IPI00908365 | Angiotensinogen | 0 | 0 | 1 | 0 | 0 | 0.75 | 1.00E+00 |
| IPI00465361|IPI00978971 | 60S ribosomal protein L13 | 1 | 0 | 0 | 0 | 0 | 0.75 | 1.00E+00 |
| IPI00221093 | 40S ribosomal protein S17 | 0 | 1 | 0 | 0 | 0 | 0.75 | 1.00E+00 |
| IPI00396378|IPI00414696 | Isoform B1 of Heterogeneous nuclear ribonucleoproteins A2/B1 | 1 | 0 | 0 | 0 | 0 | 0.75 | 1.00E+00 |
| IPI00013296|IPI00984608 | 40S ribosomal protein S18 | 0 | 1 | 0 | 0 | 0 | 0.75 | 1.00E+00 |
| IPI00303476|IPI01021250|IPI01021986 | ATP synthase subunit beta| mitochondrial | 0 | 0 | 1 | 0 | 0 | 0.75 | 1.00E+00 |
| IPI00395998|IPI00927658 | 60S ribosomal protein L32 | 0 | 0 | 1 | 0 | 0 | 0.75 | 1.00E+00 |
| IPI00973736|IPI00982652 | Uncharacterized protein | 0 | 0 | 1 | 0 | 0 | 0.75 | 1.00E+00 |
| IPI00376005|IPI00411704|IPI00855924 | Isoform 2 of Eukaryotic translation initiation factor 5A-1 | 0 | 0 | 1 | 0 | 0 | 0.75 | 1.00E+00 |
| IPI00221089|IPI00977844 | 40S ribosomal protein S13 | 0 | 1 | 0 | 0 | 0 | 0.75 | 1.00E+00 |
| IPI00033494|IPI00220573|IPI00719669 | Myosin regulatory light chain 12B | 0 | 1 | 0 | 0 | 0 | 0.75 | 1.00E+00 |
| IPI00414676|IPI00514027|IPI00555614 | Heat shock protein HSP 90-beta | 0 | 0 | 1 | 0 | 0 | 0.75 | 1.00E+00 |
| IPI00022796|IPI00419258|IPI00644653|IPI00645948 | Putative high mobility group protein B1-like 1 | 0 | 2 | 0 | 0 | 0 | 0.60 | 1.00E+00 |
| IPI00414860 | 60S ribosomal protein L37a | 0 | 2 | 0 | 0 | 0 | 0.60 | 1.00E+00 |
| IPI00655650 | 40S ribosomal protein S26 | 0 | 1 | 0 | 0 | 0 | 0.75 | 1.00E+00 |
| IPI00219160 | 60S ribosomal protein L34 | 2 | 0 | 0 | 0 | 0 | 0.60 | 1.00E+00 |
| IPI00418471 | Vimentin | 1 | 0 | 0 | 0 | 0 | 0.75 | 1.00E+00 |
| IPI00215719|IPI01020905|IPI01021193|IPI01022344|IPI01022981 | 60S ribosomal protein L18 | 0 | 1 | 0 | 0 | 0 | 0.75 | 1.00E+00 |
| IPI00007611|IPI00878218|IPI00893479 | ATP synthase subunit O| mitochondrial | 0 | 0 | 1 | 1 | 0 | 1.13 | 1.00E+00 |
| IPI00382804|IPI00396485|IPI00472724|IPI00940393 | EEF1A protein (Fragment) | 0 | 2 | 0 | 1 | 0 | 0.90 | 1.00E+00 |
| **List of proteins with the number of unique endogenous peptides found in both Human and Mouse Databases after search against Mouse database: HCD** | | | | | | | | |
| IPI00223714|IPI00331597 | Histone H1.4 | 11 | 17 | 11 | 2 | 1 | 0.18 | 1.00E-05 |
| IPI00407339 | Histone H4 | 12 | 11 | 6 | 2 | 2 | 0.28 | 3.63E-04 |
| IPI00111412 | 60S ribosomal protein L4 | 4 | 6 | 2 | 0 | 0 | 0.20 | 4.76E-03 |
| IPI00282266 | Histone H2B type 1-C/E/G | 5 | 7 | 1 | 3 | 1 | 0.56 | 5.94E-02 |
| IPI00110850|IPI00652436|IPI00874482 | Actin| cytoplasmic 1 | 3 | 2 | 0 | 0 | 0 | 0.38 | 1.00E-01 |
| IPI00553777|IPI00817004 | Putative uncharacterized protein | 2 | 3 | 0 | 0 | 0 | 0.38 | 1.00E-01 |
| IPI00461456|IPI00849847|IPI00874935 | 60S ribosomal protein L23a | 2 | 3 | 1 | 0 | 0 | 0.33 | 1.00E-01 |
| IPI00469114|IPI00845802 | Hemoglobin subunit alpha | 3 | 2 | 1 | 0 | 0 | 0.33 | 1.00E-01 |
| IPI00402981|IPI00465568|IPI00753456|IPI00988773|IPI00990583 | Isoform 2 of 40S ribosomal protein S24 | 2 | 3 | 1 | 0 | 0 | 0.33 | 1.00E-01 |
| IPI00110827 | Actin| alpha skeletal muscle | 2 | 2 | 0 | 0 | 0 | 0.43 | 1.67E-01 |
| IPI00136984|IPI00850934 | 40S ribosomal protein S7 | 2 | 2 | 1 | 0 | 0 | 0.38 | 1.67E-01 |
| IPI00625021|IPI00762542 | Uncharacterized protein | 1 | 2 | 0 | 0 | 0 | 0.50 | 3.33E-01 |
| IPI00122426|IPI00648315 | 60S ribosomal protein L19 | 1 | 2 | 0 | 0 | 0 | 0.50 | 3.33E-01 |
| IPI00330000|IPI00974916|IPI00989397 | Histone H2A type 2-A | 3 | 4 | 2 | 4 | 1 | 0.88 | 4.24E-01 |
| IPI00972920 | 40S ribosomal protein S23 | 1 | 1 | 0 | 0 | 0 | 0.60 | 5.00E-01 |
| IPI00223217|IPI00919200 | 60S ribosomal protein L13a | 1 | 1 | 0 | 0 | 0 | 0.60 | 5.00E-01 |
| IPI00108454|IPI00113655|IPI00990093 | 40S ribosomal protein S6 | 1 | 2 | 1 | 1 | 0 | 0.64 | 5.00E-01 |
| IPI00221463|IPI00265761 | Histone H2A type 3 | 2 | 3 | 2 | 4 | 2 | 1.20 | 6.52E-01 |
| IPI00123007|IPI00625397|IPI00677102|IPI00990512 | 60S ribosomal protein L31 | 1 | 1 | 1 | 0 | 1 | 0.75 | 6.67E-01 |
| IPI00404590|IPI00467914 | Putative uncharacterized protein | 1 | 1 | 1 | 1 | 1 | 1.00 | 8.33E-01 |
| IPI00137787 | 60S ribosomal protein L8 | 0 | 1 | 1 | 0 | 0 | 0.60 | 1.00E+00 |
| IPI00122421|IPI00990717 | 60S ribosomal protein L27 | 0 | 1 | 1 | 0 | 0 | 0.60 | 1.00E+00 |
| IPI00308706|IPI00988023 | 60S ribosomal protein L5 | 0 | 1 | 1 | 0 | 0 | 0.60 | 1.00E+00 |
| IPI00265107|IPI00330363|IPI00354363|IPI00462453|IPI01018378 | Uncharacterized protein | 0 | 2 | 1 | 0 | 0 | 0.50 | 1.00E+00 |
| IPI00604967|IPI00606379|IPI00606508|IPI00987179|IPI00987518|IPI00988101|IPI00988545 | Uncharacterized protein | 0 | 0 | 0 | 1 | 0 | 1.50 | 1.00E+00 |
| IPI00222550 | Putative uncharacterized protein | 0 | 2 | 0 | 0 | 0 | 0.60 | 1.00E+00 |
| IPI00227299 | Vimentin | 1 | 0 | 0 | 0 | 0 | 0.75 | 1.00E+00 |
| IPI00134097|IPI00224505|IPI00988790 | 60S ribosomal protein L13 | 1 | 0 | 0 | 0 | 0 | 0.75 | 1.00E+00 |
| IPI00317590|IPI00620156|IPI00990188|IPI01019245 | 40S ribosomal protein S18 | 0 | 1 | 0 | 0 | 0 | 0.75 | 1.00E+00 |
| IPI00338650|IPI00650026|IPI00918033|IPI00986711|IPI00987783|IPI00990954 | Non-histone chromosomal protein HMG-17 | 0 | 3 | 0 | 0 | 0 | 0.50 | 1.00E+00 |
| IPI00460103|IPI00461652|IPI00466153|IPI00621625 | 60S ribosomal protein L34 | 2 | 0 | 0 | 0 | 0 | 0.60 | 1.00E+00 |
| IPI00311236|IPI01023242 | 60S ribosomal protein L7 | 0 | 1 | 0 | 0 | 0 | 0.75 | 1.00E+00 |
| IPI00405058|IPI00622847|IPI00828488|IPI01023279 | Isoform 3 of Heterogeneous nuclear ribonucleoproteins A2/B1 | 1 | 0 | 0 | 0 | 0 | 0.75 | 1.00E+00 |
| IPI00458583|IPI00970121 | Heterogeneous nuclear ribonucleoprotein U | 0 | 0 | 1 | 0 | 0 | 0.75 | 1.00E+00 |
| IPI00108125 | Eukaryotic translation initiation factor 5A-1 | 0 | 0 | 1 | 0 | 0 | 0.75 | 1.00E+00 |
| IPI00654069 | angiotensinogen | 0 | 0 | 1 | 0 | 0 | 0.75 | 1.00E+00 |
| IPI00230133 | Histone H1.5 | 4 | 0 | 0 | 0 | 0 | 0.43 | 1.00E+00 |
| IPI00125901|IPI00989181|IPI00990510 | 40S ribosomal protein S13 | 0 | 1 | 0 | 0 | 0 | 0.75 | 1.00E+00 |
| IPI00620256 | Isoform A of Prelamin-A/C | 2 | 0 | 0 | 0 | 0 | 0.60 | 1.00E+00 |
| IPI00377441 | 40S ribosomal protein S26 | 0 | 1 | 0 | 0 | 0 | 0.75 | 1.00E+00 |
| IPI00109044|IPI00132705|IPI00758006 | myosin light chain| regulatory B-like | 0 | 1 | 0 | 0 | 0 | 0.75 | 1.00E+00 |
| IPI00125778 | Transgelin-2 | 0 | 2 | 0 | 0 | 0 | 0.60 | 1.00E+00 |
| IPI00420261|IPI00665601|IPI00853656 | High mobility group protein B1 | 0 | 2 | 0 | 0 | 0 | 0.60 | 1.00E+00 |
| IPI00465880|IPI00985790 | 40S ribosomal protein S17 | 0 | 1 | 0 | 0 | 0 | 0.75 | 1.00E+00 |
| IPI00399483 | 40S ribosomal protein S30 | 0 | 0 | 1 | 0 | 0 | 0.75 | 1.00E+00 |
| IPI00224549 | Myosin regulatory light chain 2| skeletal muscle isoform | 0 | 5 | 0 | 0 | 0 | 0.38 | 1.00E+00 |
| IPI00554929|IPI00830562|IPI00989376|IPI01016123 | Heat shock protein HSP 90-beta | 0 | 0 | 1 | 0 | 0 | 0.75 | 1.00E+00 |
| IPI00468481 | ATP synthase subunit beta| mitochondrial | 0 | 0 | 1 | 0 | 0 | 0.75 | 1.00E+00 |
| IPI00555113 | 60S ribosomal protein L18 | 0 | 1 | 0 | 0 | 0 | 0.75 | 1.00E+00 |
| IPI00117583 | Thymosin beta-10 | 0 | 1 | 0 | 0 | 0 | 0.75 | 1.00E+00 |
| IPI00230623|IPI00889220 | 60S ribosomal protein L32 | 0 | 0 | 1 | 0 | 0 | 0.75 | 1.00E+00 |
| IPI00461407 | Uncharacterized protein | 0 | 0 | 1 | 1 | 0 | 1.13 | 1.00E+00 |
| IPI00307837 | Elongation factor 1-alpha 1 | 0 | 2 | 0 | 1 | 0 | 0.90 | 1.00E+00 |

**Table G**

| **Protein accession numbers** | **Protein name** | **Control1** | **Control2** | **Control3** | **Tumor1** | **Tumor2** | **Fold Change** | **Fisher** |
| --- | --- | --- | --- | --- | --- | --- | --- | --- |
| **List of proteins with the number of unique endogenous peptides found in both Human and Mouse Databases after search against Human database: ETD** | | | | | | | | |
| IPI00453473 | Histone H4 | 4 | 3 | 2 | 1 | 1 | 0.50 | 1.67E-01 |
| IPI00217468 | Histone H1.5 | 2 | 2 | 0 | 0 | 0 | 0.43 | 1.67E-01 |
| IPI00221089|IPI00977844 | 40S ribosomal protein S13 | 0 | 0 | 0 | 2 | 2 | 3.00 | 1.67E-01 |
| IPI00217950 | Non-histone chromosomal protein HMG-17 | 1 | 2 | 0 | 0 | 0 | 0.50 | 3.33E-01 |
| IPI00002352 | Myosin regulatory light chain 2| skeletal muscle isoform | 0 | 0 | 0 | 1 | 2 | 2.50 | 3.33E-01 |
| IPI00337455|IPI00477427|IPI00641544|IPI00658138|IPI00852975|IPI00853392|IPI00872734|IPI00937352|IPI00943329 | Isoform 5 of Troponin T| fast skeletal muscle | 0 | 0 | 0 | 2 | 1 | 2.50 | 3.33E-01 |
| IPI00020101 | Histone H2B type 1-C/E/F/G/I | 4 | 5 | 5 | 8 | 5 | 1.32 | 3.61E-01 |
| IPI00216457 | Histone H2A type 2-A | 3 | 1 | 1 | 0 | 1 | 0.56 | 4.00E-01 |
| IPI00395998|IPI00927658 | 60S ribosomal protein L32 | 0 | 0 | 1 | 1 | 1 | 1.50 | 5.00E-01 |
| IPI00176903|IPI00513773|IPI00514023|IPI01014936 | Isoform 1 of Polymerase I and transcript release factor | 0 | 0 | 0 | 1 | 1 | 2.00 | 5.00E-01 |
| IPI00217469 | Histone H1.1 | 4 | 6 | 4 | 5 | 6 | 1.15 | 5.75E-01 |
| IPI00217465|IPI00217466|IPI00217467 | Histone H1.2 | 7 | 5 | 8 | 6 | 5 | 0.85 | 5.93E-01 |
| IPI00025091 | 40S ribosomal protein S11 | 1 | 2 | 2 | 2 | 2 | 1.13 | 6.29E-01 |
| IPI00550239 | Histone H1.0 | 1 | 1 | 2 | 2 | 1 | 1.07 | 7.00E-01 |
| IPI00013415 | 40S ribosomal protein S7 | 1 | 1 | 1 | 0 | 2 | 1.00 | 7.50E-01 |
| IPI00012772|IPI00909884 | 60S ribosomal protein L8 | 1 | 1 | 0 | 2 | 0 | 1.20 | 7.50E-01 |
| IPI00218606 | 40S ribosomal protein S23 | 1 | 1 | 2 | 1 | 1 | 0.86 | 8.33E-01 |
| IPI00003918 | 60S ribosomal protein L4 | 1 | 1 | 1 | 1 | 1 | 1.00 | 8.33E-01 |
| IPI00414860 | 60S ribosomal protein L37a | 0 | 0 | 3 | 3 | 0 | 1.25 | 1.00E+00 |
| IPI00000494 | 60S ribosomal protein L5 | 0 | 0 | 3 | 2 | 0 | 1.00 | 1.00E+00 |
| IPI00306332 | 60S ribosomal protein L24 | 1 | 0 | 1 | 0 | 0 | 0.60 | 1.00E+00 |
| IPI00021428 | Actin| alpha skeletal muscle | 0 | 0 | 0 | 1 | 0 | 1.50 | 1.00E+00 |
| IPI00410714|IPI00853068 | Hemoglobin subunit alpha | 0 | 0 | 0 | 0 | 1 | 1.50 | 1.00E+00 |
| IPI00027487|IPI00908787|IPI00909684 | Creatine kinase M-type | 0 | 0 | 0 | 0 | 1 | 1.50 | 1.00E+00 |
| IPI00007611|IPI00878218|IPI00893479 | ATP synthase subunit O| mitochondrial | 0 | 0 | 0 | 0 | 1 | 1.50 | 1.00E+00 |
| IPI00008438|IPI00749512 | 40S ribosomal protein S10 | 0 | 0 | 0 | 0 | 1 | 1.50 | 1.00E+00 |
| IPI00219155 | 60S ribosomal protein L27 | 0 | 0 | 0 | 1 | 0 | 1.50 | 1.00E+00 |
| IPI00440493|IPI00471928|IPI00908963 | ATP synthase subunit alpha| mitochondrial | 0 | 0 | 0 | 0 | 1 | 1.50 | 1.00E+00 |
| IPI00029750 | Isoform 1 of 40S ribosomal protein S24 | 0 | 1 | 0 | 0 | 0 | 0.75 | 1.00E+00 |
| IPI00025329 | 60S ribosomal protein L19 | 1 | 0 | 0 | 0 | 0 | 0.75 | 1.00E+00 |
| IPI00550021|IPI00651660|IPI00877635 | 60S ribosomal protein L3 | 1 | 0 | 0 | 0 | 0 | 0.75 | 1.00E+00 |
| IPI00973736|IPI00982652 | Uncharacterized protein | 0 | 1 | 0 | 0 | 0 | 0.75 | 1.00E+00 |
| IPI00022796|IPI00419258|IPI00644653|IPI00645948 | Putative high mobility group protein B1-like 1 | 0 | 0 | 1 | 0 | 0 | 0.75 | 1.00E+00 |
| IPI00219160 | 60S ribosomal protein L34 | 2 | 0 | 0 | 0 | 0 | 0.60 | 1.00E+00 |
| IPI00220871 | 60S ribosomal protein L37 | 0 | 1 | 0 | 2 | 0 | 1.50 | 1.00E+00 |
| IPI00013296|IPI00984608 | 40S ribosomal protein S18 | 0 | 1 | 2 | 0 | 1 | 0.75 | 1.00E+00 |
| IPI00021266|IPI00789159|IPI00793523|IPI00794894 | 60S ribosomal protein L23a | 0 | 1 | 1 | 0 | 1 | 0.90 | 1.00E+00 |
| IPI00026302 | 60S ribosomal protein L31 | 0 | 1 | 0 | 1 | 0 | 1.13 | 1.00E+00 |
| IPI00030179|IPI00794746 | 60S ribosomal protein L7 | 0 | 0 | 1 | 1 | 0 | 1.13 | 1.00E+00 |
| **List of proteins with the number of unique endogenous peptides found in both Human and Mouse Databases after search against Mouse database: ETD** | | | | | | | | |
| IPI00407339 | Histone H4 | 4 | 3 | 2 | 1 | 1 | 0.50 | 1.67E-01 |
| IPI00230133 | Histone H1.5 | 2 | 2 | 0 | 0 | 0 | 0.43 | 1.67E-01 |
| IPI00125901|IPI00989181|IPI00990510 | 40S ribosomal protein S13 | 0 | 0 | 0 | 2 | 2 | 3.00 | 1.67E-01 |
| IPI00338650|IPI00987783|IPI00990954 | Non-histone chromosomal protein HMG-17 | 1 | 2 | 0 | 0 | 0 | 0.50 | 3.33E-01 |
| IPI00229055|IPI00229058|IPI00318623|IPI00336780|IPI00465745|IPI00466353|IPI00466881|IPI00469990|IPI00753801|IPI00830453|IPI00830536|IPI00830654|IPI00831350 | Isoform A5e17 of Troponin T| fast skeletal muscle | 0 | 0 | 0 | 2 | 1 | 2.50 | 3.33E-01 |
| IPI00224549 | Myosin regulatory light chain 2| skeletal muscle isoform | 0 | 0 | 0 | 1 | 2 | 2.50 | 3.33E-01 |
| IPI00282266 | Histone H2B type 1-C/E/G | 4 | 5 | 5 | 8 | 5 | 1.32 | 3.61E-01 |
| IPI00330000|IPI00974916|IPI00989397 | Histone H2A type 2-A | 3 | 1 | 1 | 0 | 1 | 0.56 | 4.00E-01 |
| IPI00230623 | 60S ribosomal protein L32 | 0 | 0 | 1 | 1 | 1 | 1.50 | 5.00E-01 |
| IPI00117689 | Polymerase I and transcript release factor | 0 | 0 | 0 | 1 | 1 | 2.00 | 5.00E-01 |
| IPI00625021|IPI00762542 | Uncharacterized protein | 1 | 2 | 2 | 2 | 2 | 1.13 | 6.29E-01 |
| IPI00223714|IPI00331597 | Histone H1.4 | 7 | 7 | 8 | 7 | 7 | 0.96 | 6.47E-01 |
| IPI00404590|IPI00467914 | Putative uncharacterized protein | 1 | 1 | 2 | 2 | 1 | 1.07 | 7.00E-01 |
| IPI00136984|IPI00850934 | 40S ribosomal protein S7 | 1 | 1 | 1 | 0 | 2 | 1.00 | 7.50E-01 |
| IPI00137787 | 60S ribosomal protein L8 | 1 | 1 | 0 | 2 | 0 | 1.20 | 7.50E-01 |
| IPI00972920 | 40S ribosomal protein S23 | 1 | 1 | 2 | 1 | 1 | 0.86 | 8.33E-01 |
| IPI00111412 | 60S ribosomal protein L4 | 1 | 1 | 1 | 1 | 1 | 1.00 | 8.33E-01 |
| IPI00222550 | Putative uncharacterized protein | 0 | 0 | 3 | 3 | 0 | 1.25 | 1.00E+00 |
| IPI00308706|IPI00988023 | 60S ribosomal protein L5 | 0 | 0 | 3 | 2 | 0 | 1.00 | 1.00E+00 |
| IPI00762051 | 60S ribosomal protein L24 | 1 | 0 | 1 | 0 | 0 | 0.60 | 1.00E+00 |
| IPI00469114 | Hemoglobin subunit alpha | 0 | 0 | 0 | 0 | 1 | 1.50 | 1.00E+00 |
| IPI00127596 | Creatine kinase M-type | 0 | 0 | 0 | 0 | 1 | 1.50 | 1.00E+00 |
| IPI00122421 | 60S ribosomal protein L27 | 0 | 0 | 0 | 1 | 0 | 1.50 | 1.00E+00 |
| IPI00130280|IPI00857439 | ATP synthase subunit alpha| mitochondrial | 0 | 0 | 0 | 0 | 1 | 1.50 | 1.00E+00 |
| IPI00112448 | 40S ribosomal protein S10 | 0 | 0 | 0 | 0 | 1 | 1.50 | 1.00E+00 |
| IPI00110827 | Actin| alpha skeletal muscle | 0 | 0 | 0 | 1 | 0 | 1.50 | 1.00E+00 |
| IPI00118986 | ATP synthase subunit O| mitochondrial | 0 | 0 | 0 | 0 | 1 | 1.50 | 1.00E+00 |
| IPI00321170|IPI00753623 | 60S ribosomal protein L3 | 1 | 0 | 0 | 0 | 0 | 0.75 | 1.00E+00 |
| IPI00122426|IPI00648315 | 60S ribosomal protein L19 | 1 | 0 | 0 | 0 | 0 | 0.75 | 1.00E+00 |
| IPI00466153|IPI00621625 | 60S ribosomal protein L34 | 2 | 0 | 0 | 0 | 0 | 0.60 | 1.00E+00 |
| IPI00402981|IPI00465568|IPI00753456|IPI00988773|IPI00990583 | Isoform 2 of 40S ribosomal protein S24 | 0 | 1 | 0 | 0 | 0 | 0.75 | 1.00E+00 |
| IPI00399483 | 40S ribosomal protein S30 | 0 | 1 | 0 | 0 | 0 | 0.75 | 1.00E+00 |
| IPI00420261|IPI00665601|IPI00853656 | High mobility group protein B1 | 0 | 0 | 1 | 0 | 0 | 0.75 | 1.00E+00 |
| IPI00453924 | 60S ribosomal protein L37 | 0 | 1 | 0 | 2 | 0 | 1.50 | 1.00E+00 |
| IPI00317590|IPI00620156|IPI00990188|IPI01019245 | 40S ribosomal protein S18 | 0 | 1 | 2 | 0 | 1 | 0.75 | 1.00E+00 |
| IPI00461456|IPI00849847|IPI00874935|IPI00986856 | 60S ribosomal protein L23a | 0 | 1 | 1 | 0 | 1 | 0.90 | 1.00E+00 |
| IPI00123007|IPI00625397|IPI00677102|IPI00990512 | 60S ribosomal protein L31 | 0 | 1 | 0 | 1 | 0 | 1.13 | 1.00E+00 |
| IPI00311236|IPI01023242 | 60S ribosomal protein L7 | 0 | 0 | 1 | 1 | 0 | 1.13 | 1.00E+00 |

**Table H**

| **Category** | **Term** | **Count** | **%** | **PValue** | **Genes** |
| --- | --- | --- | --- | --- | --- |
| GOTERM_BP_FAT | GO:0030036~actin cytoskeleton organization | 6 | 14.29 | 8.93E-06 | IPI00843975, IPI00216694, IPI00298994, IPI00163187, IPI00289334, IPI00302592 |
| GOTERM_BP_FAT | GO:0030029~actin filament-based process | 6 | 14.29 | 1.22E-05 | IPI00843975, IPI00216694, IPI00298994, IPI00163187, IPI00289334, IPI00302592 |
| GOTERM_BP_FAT | GO:0032507~maintenance of protein location in cell | 4 | 9.52 | 2.04E-05 | IPI00843975, IPI00298994, IPI00289334, IPI00302592 |
| GOTERM_BP_FAT | GO:0045185~maintenance of protein location | 4 | 9.52 | 3.30E-05 | IPI00843975, IPI00298994, IPI00289334, IPI00302592 |
| GOTERM_BP_FAT | GO:0051651~maintenance of location in cell | 4 | 9.52 | 3.30E-05 | IPI00843975, IPI00298994, IPI00289334, IPI00302592 |
| GOTERM_BP_FAT | GO:0007016~cytoskeletal anchoring at plasma membrane | 3 | 7.14 | 4.66E-05 | IPI00843975, IPI00298994, IPI00289334 |
| GOTERM_BP_FAT | GO:0051235~maintenance of location | 4 | 9.52 | 7.83E-05 | IPI00843975, IPI00298994, IPI00289334, IPI00302592 |
| GOTERM_BP_FAT | GO:0007015~actin filament organization | 4 | 9.52 | 1.11E-04 | IPI00843975, IPI00216694, IPI00163187, IPI00302592 |
| GOTERM_BP_FAT | GO:0007010~cytoskeleton organization | 6 | 14.29 | 2.06E-04 | IPI00843975, IPI00216694, IPI00298994, IPI00163187, IPI00289334, IPI00302592 |
| GOTERM_BP_FAT | GO:0008104~protein localization | 7 | 16.67 | 7.10E-04 | IPI00031461, IPI00843975, IPI00022774, IPI00298994, IPI00216319, IPI00289334, IPI00302592 |
| GOTERM_BP_FAT | GO:0022614~membrane to membrane docking | 2 | 4.76 | 6.64E-03 | IPI00843975, IPI00219365 |
| GOTERM_BP_FAT | GO:0007043~cell-cell junction assembly | 2 | 4.76 | 2.37E-02 | IPI00215948, IPI00298994 |
| GOTERM_BP_FAT | GO:0051017~actin filament bundle formation | 2 | 4.76 | 2.76E-02 | IPI00843975, IPI00163187 |
| GOTERM_BP_FAT | GO:0007159~leukocyte adhesion | 2 | 4.76 | 3.66E-02 | IPI00843975, IPI00219365 |
| GOTERM_BP_FAT | GO:0022406~membrane docking | 2 | 4.76 | 4.05E-02 | IPI00843975, IPI00219365 |
| GOTERM_BP_FAT | GO:0045216~cell-cell junction organization | 2 | 4.76 | 4.56E-02 | IPI00215948, IPI00298994 |
| GOTERM_BP_FAT | GO:0034329~cell junction assembly | 2 | 4.76 | 5.32E-02 | IPI00215948, IPI00298994 |
| GOTERM_BP_FAT | GO:0046907~intracellular transport | 4 | 9.52 | 5.40E-02 | IPI00022774, IPI00215965, IPI00216319, IPI00302592 |
| GOTERM_BP_FAT | GO:0007155~cell adhesion | 4 | 9.52 | 6.31E-02 | IPI00843975, IPI00018219, IPI00215948, IPI00219365 |
| GOTERM_BP_FAT | GO:0022610~biological adhesion | 4 | 9.52 | 6.33E-02 | IPI00843975, IPI00018219, IPI00215948, IPI00219365 |
| GOTERM_BP_FAT | GO:0034330~cell junction organization | 2 | 4.76 | 7.32E-02 | IPI00215948, IPI00298994 |
| GOTERM_BP_FAT | GO:0045184~establishment of protein localization | 4 | 9.52 | 7.90E-02 | IPI00031461, IPI00022774, IPI00216319, IPI00302592 |
| GOTERM_BP_FAT | GO:0032386~regulation of intracellular transport | 2 | 4.76 | 9.04E-02 | IPI00075248, IPI00302592 |
| KEGG_PATHWAY | hsa05130:Pathogenic Escherichia coli infection | 3 | 7.14 | 5.24E-03 | IPI00843975, IPI00013683, IPI00444262 |
| KEGG_PATHWAY | hsa04670:Leukocyte transendothelial migration | 3 | 7.14 | 2.13E-02 | IPI00843975, IPI00215948, IPI00219365 |
| KEGG_PATHWAY | hsa04510:Focal adhesion | 3 | 7.14 | 5.68E-02 | IPI00298994, IPI00289334, IPI00302592 |

**Table I**

| **Category** | **Term** | **Count** | **%** | **PValue** | **Genes** |
| --- | --- | --- | --- | --- | --- |
| GOTERM_BP_FAT | GO:0015980~energy derivation by oxidation of organic compounds | 4 | 13.333 | 2.42E-04 | IPI00220150, IPI00028520, IPI00157144, IPI00386271 |
| GOTERM_BP_FAT | GO:0006091~generation of precursor metabolites and energy | 4 | 13.333 | 2.31E-03 | IPI00220150, IPI00028520, IPI00157144, IPI00386271 |
| GOTERM_BP_FAT | GO:0045333~cellular respiration | 3 | 10.000 | 3.20E-03 | IPI00220150, IPI00028520, IPI00386271 |
| GOTERM_BP_FAT | GO:0055085~transmembrane transport | 4 | 13.333 | 1.23E-02 | IPI00024804, IPI00007188, IPI00219729, IPI00386271 |
| GOTERM_BP_FAT | GO:0022904~respiratory electron transport chain | 2 | 6.667 | 5.53E-02 | IPI00028520, IPI00386271 |
| GOTERM_BP_FAT | GO:0006754~ATP biosynthetic process | 2 | 6.667 | 7.62E-02 | IPI00024804, IPI00177817 |
| GOTERM_BP_FAT | GO:0051640~organelle localization | 2 | 6.667 | 7.87E-02 | IPI00215914, IPI00024804 |
| GOTERM_BP_FAT | GO:0009206~purine ribonucleoside triphosphate biosynthetic process | 2 | 6.667 | 8.36E-02 | IPI00024804, IPI00177817 |
| GOTERM_BP_FAT | GO:0009201~ribonucleoside triphosphate biosynthetic process | 2 | 6.667 | 8.44E-02 | IPI00024804, IPI00177817 |
| GOTERM_BP_FAT | GO:0009145~purine nucleoside triphosphate biosynthetic process | 2 | 6.667 | 8.44E-02 | IPI00024804, IPI00177817 |
| GOTERM_BP_FAT | GO:0009142~nucleoside triphosphate biosynthetic process | 2 | 6.667 | 8.69E-02 | IPI00024804, IPI00177817 |
| GOTERM_BP_FAT | GO:0046034~ATP metabolic process | 2 | 6.667 | 8.93E-02 | IPI00024804, IPI00177817 |
| GOTERM_BP_FAT | GO:0022900~electron transport chain | 2 | 6.667 | 9.66E-02 | IPI00028520, IPI00386271 |
| GOTERM_BP_FAT | GO:0009205~purine ribonucleoside triphosphate metabolic process | 2 | 6.667 | 9.90E-02 | IPI00024804, IPI00177817 |
| GOTERM_BP_FAT | GO:0009152~purine ribonucleotide biosynthetic process | 2 | 6.667 | 9.90E-02 | IPI00024804, IPI00177817 |
| GOTERM_BP_FAT | GO:0009199~ribonucleoside triphosphate metabolic process | 2 | 6.667 | 9.98E-02 | IPI00024804, IPI00177817 |
| KEGG_PATHWAY | hsa05010:Alzheimer's disease | 3 | 10.000 | 1.93E-02 | IPI00028520, IPI00024804, IPI00177817 |
| KEGG_PATHWAY | hsa04020:Calcium signaling pathway | 3 | 10.000 | 2.23E-02 | IPI00024804, IPI00007188, IPI00177817 |

**Table J**

| *Clinicopathological*  *parameter* | *Patients* | |
| --- | --- | --- |
|  | **Sample %**    **N=12 100** | |
| ***Age of diagnosis (yrs)*** | | |
| 45-70 | | |
| ***Sex*** |  |  |
| Male | 9 | 75 |
| Female | 3 | 25 |
| ***Smoking habit*** |  |  |
| Yes | 9 | 75 |
| No | 0 | 0 |
| Ex | 3 | 25 |
| ***Alcohol consumption*** |  |  |
| Yes | 6 | 50 |
| No | 3 | 25 |
| Ex | 3 | 25 |
| ***Site of location*** |  |  |
| Tongue | 6 | 50 |
| Soft palate | 3 | 25 |
| Bucal floor | 3 | 25 |
| ***Histological differentiation**** |  |  |
| Well differentiated | 3 | 25 |
| Moderately differentiated | 6 | 50 |
| Poorly differentiated | 3 | 25 |

*** Histological differentiation according to guidelines given by** Barnes L, Eveson J, Reichart P, Sidransky D. World Health Organization Classification of Tumours:Pathology and Genetics of Head and Neck Tumours. Lyon: IARC Press; 2005.

***Supporting Information: FIGURES***

**Figure A**

**
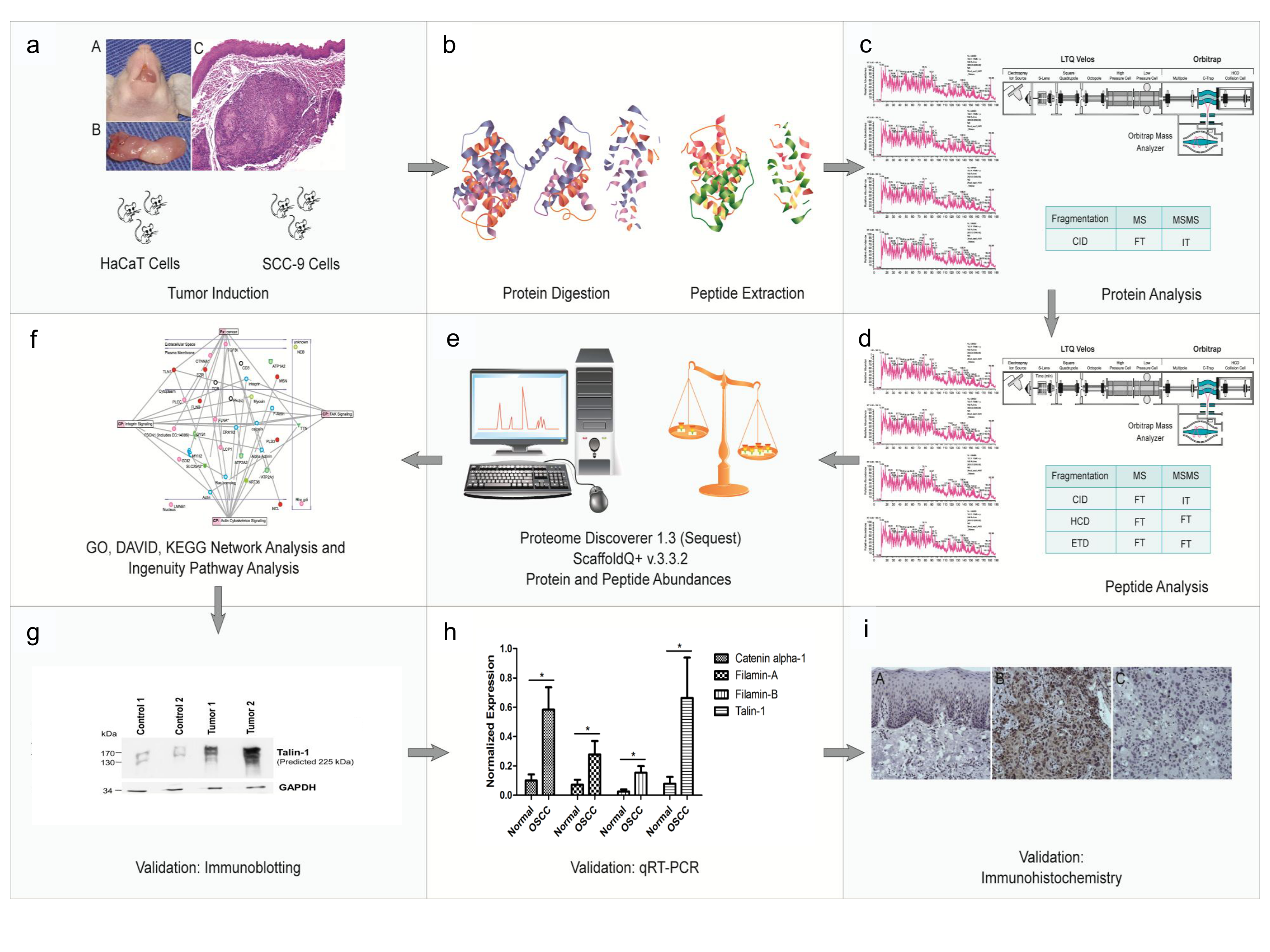
**

**Figure B**

**a**

**
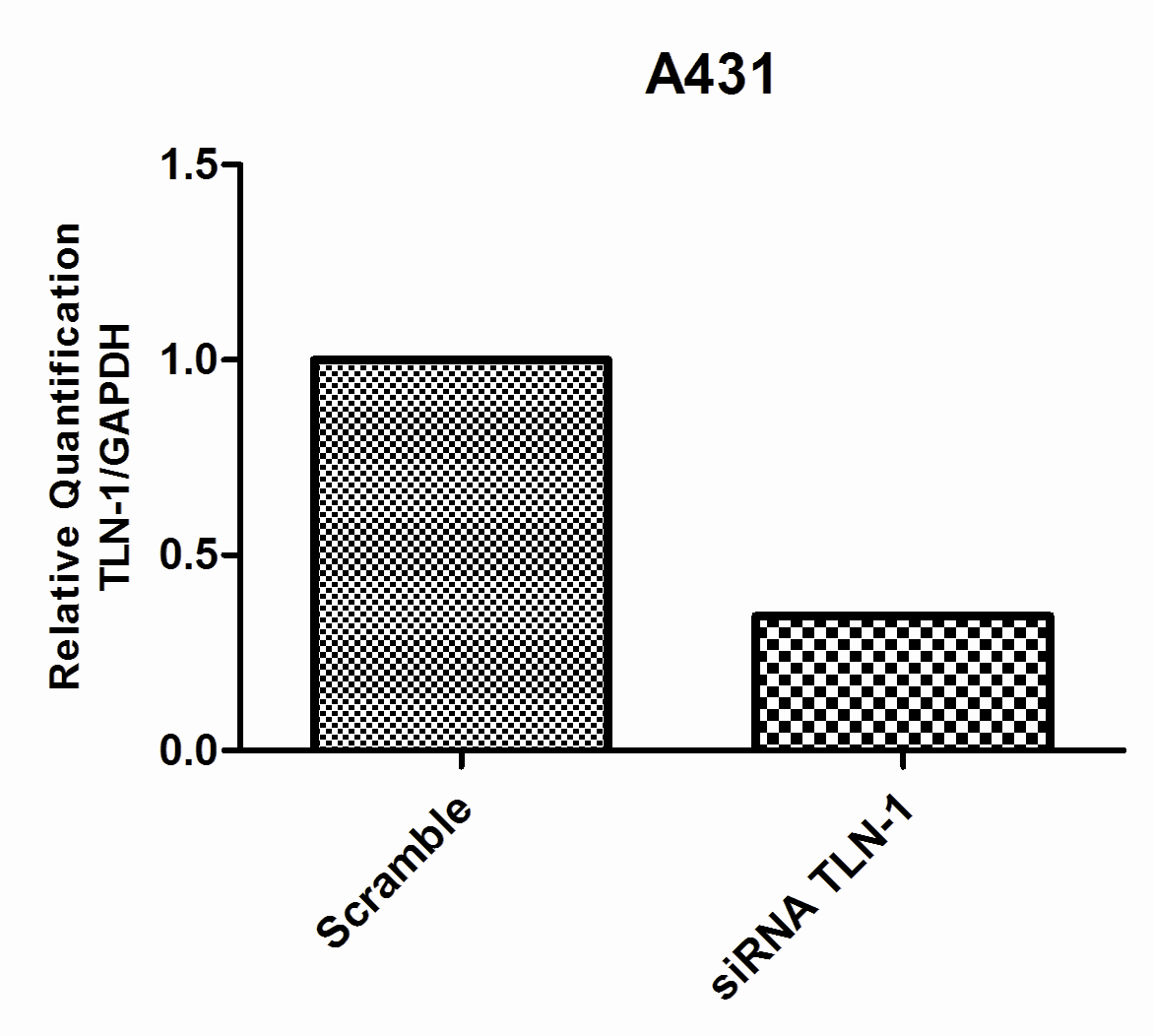
**

**b**

**
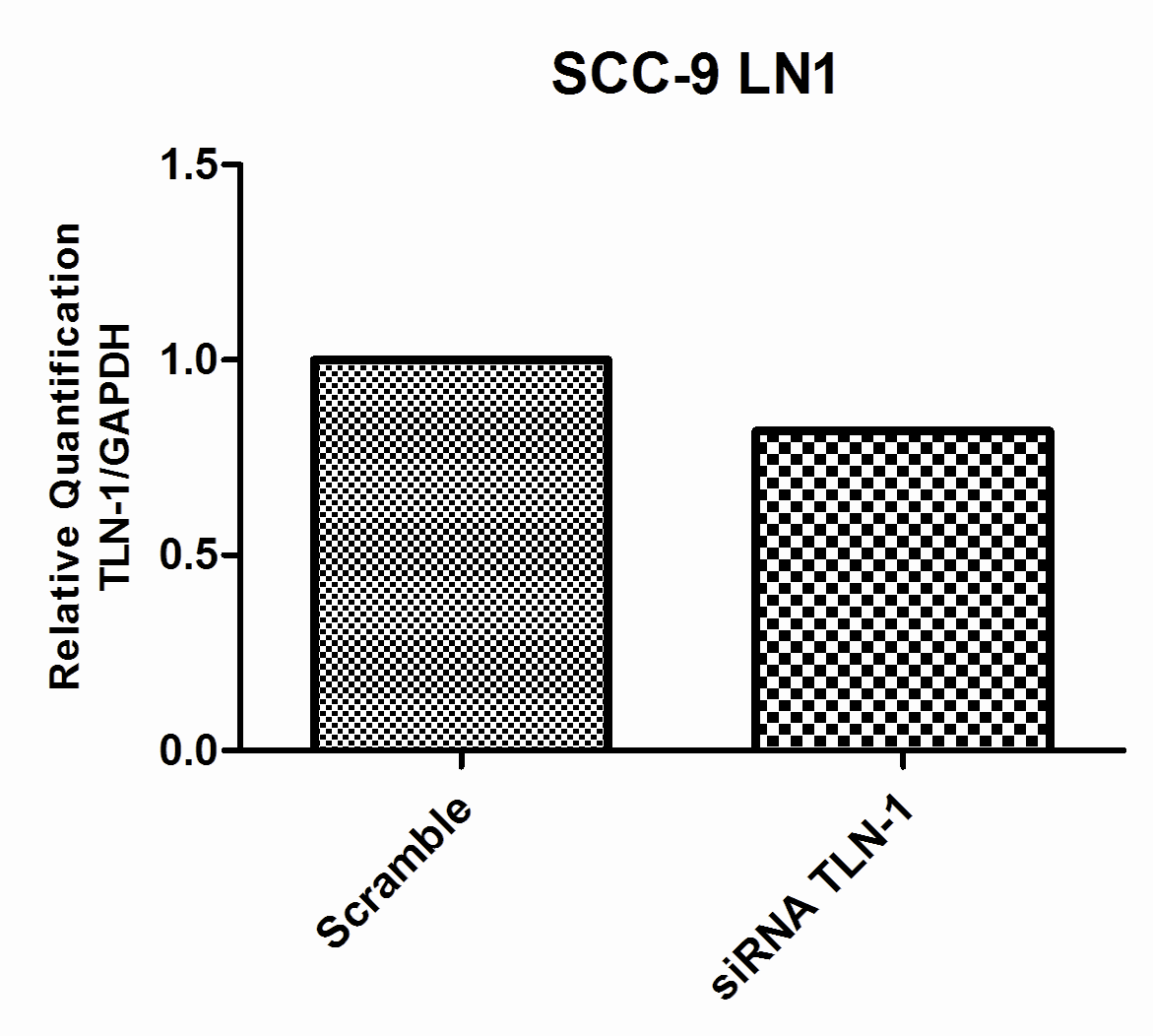
**
